# Depression, anxiety, and stress in oral lichen planus: a systematic review and meta-analysis

**DOI:** 10.1007/s00784-021-04114-0

**Published:** 2021-08-30

**Authors:** Teresa De Porras-Carrique, Miguel Ángel González-Moles, Saman Warnakulasuriya, Pablo Ramos-García

**Affiliations:** 1 School of Dentistry, University of Granada, Granada, Spain; 2 Biohealth Research Institute (IBS), Granada, Spain; 3 WHO Collaborating Centre for Oral Cancer, London, UK; 4 Faculty of Dentistry, Oral and Craniofacial Sciences, King’s College London, London, UK

**Keywords:** Oral lichen planus, Depression, Anxiety, Stress, Systematic review, Meta-analysis

## Abstract

**Objectives:**

We present this systematic review and meta-analyses to evaluate current evidence on the prevalence of depression, anxiety, and stress in patients with oral lichen planus and their magnitude of association.

**Material and methods:**

We searched PubMed, Embase, Web of Science, Scopus, PsycInfo, and Google Scholar for studies published before January 2021. We evaluated the quality of studies using a specific method for systematic reviews addressing prevalence questions, designed by the Joanna Briggs Institute. We carried out meta-analyses and performed heterogeneity, subgroups, meta-regression, and small-study effects analyses.

**Results:**

Fifty-one studies (which recruited 6,815 patients) met the inclusion criteria. Our results reveal a high prevalence of depression (31.19%), anxiety (54.76%), and stress (41.10%) in oral lichen planus. Furthermore, OLP patients presented a significantly higher relative frequency than control group without OLP for depression (OR = 6.15, 95% CI = 2.73–13.89, *p* < 0.001), anxiety (OR = 3.51, 95% CI = 2.10–5.85, *p* < 0.001), and stress (OR = 3.64, 95% CI = 1.48–8.94, *p* = 0.005), showing large effect sizes. Subgroups meta-analyses showed the relevance of the participation of psychologists and psychiatrists in the diagnosis of depression, anxiety, and stress in patients with OLP. Multivariable meta-regression analysis showed the importance of the comorbidity of depression-anxiety in patients with OLP.

**Conclusions:**

Our systematic review and meta-analysis show that patients with OLP suffer a higher prevalence of depression, anxiety, and stress, being more frequent than in general population.

Clinical relevance

In the dental clinic, especially dentists should be aware of depression, anxiety, and stress in OLP patients to achieve a correct referral.

**Supplementary Information:**

The online version contains supplementary material available at 10.1007/s00784-021-04114-0.

## Introduction

Oral lichen planus (OLP) is a chronic inflammatory autoimmune disease that presents with white reticular lesions accompanied or not by erythematous, erosive, plaque, bullous, or papular lesions [1]. The importance of the disease lies in its frequency, affecting 1% of the general population as recently has been documented, with a higher prevalence in Europe (1.38%) [2]. Furthermore, OLP is now considered undoubtedly an oral potentially malignant disorder with a risk of progression to cancer in 2.28% of the affected population [1, 3–5].

A widely recognized and generally accepted feature of OLP is related to its possible association with some psychological disorders [6, 7] among which are essentially anxiety, depression, and stress [8–10]. A systematic review has reported the presence of psychological disorders in patients suffering from OLP [11], and more recently, a meta-analysis corroborates the association between cutaneous and oral lichen planus with depression and anxiety [12]. The aforementioned meta-analysis [12], the only one published to date, even being the work that provides the greatest scientific evidence on the subject, presents critically low methodological quality. As will be discussed later, there is significant bias in the selection of included papers that impacts on the strength of this review.

Encountering cases with OLP is not uncommon in clinical dental practice. The management of OLP has multiple aspects, all of which are important and complex, such as its chronic nature and consequently the frequent need to prescribe prolonged treatments with immunosuppressants, i.e., topical corticosteroids; its potential to evolve into oral cancer, requiring lifelong follow-up; its association with systemic diseases, among which are diabetes mellitus, hypertension, hepatitis C, and some autoimmune diseases Hashimoto’s thyroiditis and thymoma [13–17]; and also its association with psychological disorders. The recognition of psychological disorders in patients with OLP is especially complex due to the difficulty to exploring this aspect in the dental clinic. As a consequence of the reticence of many patients to reveal or recognize their psychiatric diseases, particularly if this topic is not specifically investigated, the patient will probably keep it hidden. Furthermore, many patients with OLP, even admitting to being subjected to an altered emotional state, have not previously been diagnosed by a psychologist or a psychiatrist. In addition, probably, it is likely that patients, due to fear of the adverse effects of the treatment or even embarrassment, do not make the decision to ask for medical advice. Finally, it must be recognized that many dentists may not feel authorized or qualified, or even not knowing how to refer a patient for a psychological evaluation. Another relevant dimension concerns the extent to which it could affect the emotional state of the patient with OLP to be informed of the risk of developing oral cancer.

All these questions justify carrying out a thorough investigation on the subject with the aim of knowing, based on scientific evidence, what is the real magnitude of the problem, what are the clinical aspects of a patient with OLP that should make the dentist suspect the presence of an associated psychological disorder, and what should be the attitude in the management of these patients in the dental clinic. To achieve these objectives, a systematic review and meta-analysis have been carried out to qualitatively and quantitatively evaluate the prevalence and magnitude of the association between OLP and psychological disorders, as well as the associated factors, following strict criteria validated in international consensus that guarantee obtaining of results based on scientific methodology leading to a high quality of evidence.

## Material and methods

### Framework design

This systematic review and meta-analysis closely followed the criteria of *Cochrane Handbook for Systematic Reviews of Interventions* [18] and Joanna Briggs Institute (University of Adelaide, Australia) for systematic reviews formulating focused questions of prevalence and for proportion meta-analyses. It was also designed, conducted, and validated according to *A MeaSurement Tool to Assess systematic Reviews* (AMSTAR2) high standards [19], and reporting complied with MOOSE and PRISMA guidelines [20, 21].

To assess the prevalence of mental disorders among OLP patients, Condition, Context and Population (*CoCoPop) framework* was designed: condition, proportion of cases with depression, anxiety, and/or stress, expressed as percentage; context, their associated characteristics (i.e., geographical area, suspicion method for depression, anxiety, and stress, specialist implied in the diagnosis of metal disorders, publication language, sex, age, tobacco, alcohol, type of OLP, year of publication, risk of bias, and human development index); population, participants with OLP diagnosed by clinical and/or histopathological criteria.

To assess the magnitude of association between mental disorders and OLP, *PECOTS framework* was designed: population, participants with OLP diagnosed by clinical and/or histopathological criteria; exposure, cases with depression, anxiety, and/or stress; comparison, healthy controls (i.e., non-affected by the precedent mental disorders); outcome, magnitude of association using odds ratios as effect size measure, with 95% confidence intervals; timing, no restrictions by follow-up period or publication date; setting, observational studies published in any language.

### Protocol

In order to minimize risk of bias and improve the transparency, precision, and integrity of our systematic review and meta-analysis, a protocol on its methodology has been a priori designed and submitted in *PROSPERO International prospective register of systematic reviews* (www.crd.york.ac.uk/PROSPERO; registration code CRD42020222371). Our protocol also complied with PRISMA-P statement in order to ensure scientific rigor [22].

### Search strategy

We searched MEDLINE (through PubMed), Embase, PsycInfo, Web of Science, and Scopus databases for studies published before the search date (upper limit, January 2021), with no lower date limit. Searches were built to maximize sensitivity and combined thesaurus terms used by the databases (i.e., MeSH and Emtree) with free terms (Table 1, Appendix p.5). Only keywords synonyms or related to oral lichen planus were included, to retrieve the maximum number of possible registers. An additional screening was performed handsearching the reference lists of retrieved included studies and using Google. All references were managed using Mendeley v.1.19.4 (Elsevier, Amsterdam, The Netherlands); duplicates were also removed via this software.

### Eligibility criteria

The inclusion criteria were the following: (1) original studies, without publication language (studies published in English [*n* = 47], Chinese [*n* = 1], French [*n* = 1], Italian [*n* = 1], and Spanish [*n* = 1] were identified and included) or date restrictions; (2) studies analyzing the prevalence of depression, anxiety, or stress in patients with OLP (with or without a control group), and/or the magnitude of association (control group needed); (3) observational study design; (4) when results derived from the same study population, we included the most recently reported or those providing more data; the use of the same population in different studies was determined by verifying the name and affiliation of authors, location of the study, source of patients, and recruitment period.

The exclusion criteria were the following: (1) retractions, reviews, meta-analyses, case reports, editorials, letters, meeting abstracts, personal comments, or book chapters; (2) animal research or in vitro studies; (3) absence of healthy control group for the magnitude of association analysis; (4) lack of essential data for statistical analyses; (5) presence of aggregated data for OLP and cutaneous or genital lichen planus.

### Study selection process

Eligibility criteria were applied independently by two authors (TDPC and PRG). Any discrepancies were resolved by consensus with a third author (MAGM). Evaluators were first trained and calibrated for the process of identification and selection of studies, performing several screening rounds (50 papers each). The reliability of the study selection process was estimated calculating inter-agreement scores and Cohen’s kappa (κ) values. Articles were selected in two stages: screening titles and abstracts for those apparently meeting inclusion criteria (stage I, 100% of agreement; *κ* = 1.00), and reading the full-text of previously selected articles, excluding those not meeting eligibility criteria (stage II, 99.70% of agreement; *κ* = 0.95).

### Data extraction

One author (TDPC) independently extracted data from the selected articles. A standardized full-text analysis was performed using Excel v.16.46 spreadsheets (Microsoft, Redmond, WA, USA). Datasets were crosschecked by a second author (PRG). All discrepancies were also solved by consensus. Data were gathered on the first, last, and corresponding author; publication year; country and continent; source of patient recruitment; recruitment and follow-up periods; sample size; absolute and relative frequencies of mental disorders; study design; location and clinical appearance of lesions; diagnostic criteria for OLP; suspicion method for mental disorders; specialists implied; sex; age; and tobacco and alcohol consumption.

### Evaluation of quality and risk of bias of primary-level studies

Two authors (TDPC and PRG) evaluated the quality and risk of using a specific method for systematic reviews addressing prevalence questions (Joanna Briggs Institute, University of Adelaide, Australia) [23]. The following items were critically appraised: (1) Was the sample representative of the target population?; (2) Were study participants recruited in an appropriate way?; (3) Was the sample size adequate?; (4) Were the study subjects and the setting described in detail?; (5) Was the data analysis conducted with sufficient coverage of the identified sample?; (6) Were objective, standard criteria used for the measurement of the condition?; (7) Was the condition measured reliably?; (8) Was the statistical analysis appropriate?; (9) Were all important confounding factors/subgroups/differences identified and accounted for?; (10) Were subpopulations identified using objective criteria?. Each domain was categorized as “Yes” (low RoB), “Unclear” (moderate RoB), and “No” (High RoB). Furthermore, a specific score was attributed to individual items (low RoB = 3; moderate RoB = 2; high RoB = 1) to obtain an overall RoB estimate.

### Statistical analysis

The prevalence of mental disorders among patients with OLP was calculated extracting the raw numerators (number of cases with depression, anxiety, and stress) and denominators (patients with OLP). These proportions and their corresponding 95% confidence intervals (95%CI), constructed using the score method [24], were meta-analyzed to obtain pooled proportions (PP) expressed as percentage. The influence of studies with extreme values (0, 100, or close to 0 or 100) was minimized by using Freeman-Tukey double-arcsine transformation, to stabilize the variance of the study-specific prevalence [25]. The magnitude of association between OLP and mental disorders (i.e., depression, anxiety, and stress) was also separately explored estimating and combining odds ratios (OR) with 95% CI. All meta-analyses were performed using random-effects models, weighed by the inverse-variance based on the DerSimonian and Laird method [26], to account for the possibility that there are different underlying results among study subpopulations (e.g., differences in geographic areas, sex, age, suspicion method, etc.). Forest plots were constructed to graphically represent the overall effect and for subsequent visual inspection analyses (*p* < 0.05 was considered significant).

Heterogeneity between studies was assessed applying the χ^2^-based Cochran’s Q test (given its low statistical power, *p* < 0.10 was considered significant). *I*^2^ statistic was also quantified (values of 50–75% were interpreted as moderate-to-high degree of inconsistency across the studies) to estimate what proportion of the variance in observed effects reflects variation in true effects, rather than sampling error [27, 28]. Preplanned stratified meta-analyses were performed to identify potential sources of heterogeneity and to determine subgroups-specific prevalence [29]. The potential effect of study covariates on the prevalence of mental disorders in OLP was also explored using meta-regression [30]. We performed univariable and multivariable random-effects meta-regression analyses using the restricted maximum likelihood (REML) method [31]. The covariates identified to be statistically significant (*p* < 0.05) in a first-step univariable analysis were included in a multivariable meta-regression model. Considering the low number of studies with data available for some meta-regression analyses, the *p* values were calculated using a permutation test based on Monte Carlo simulations (1,000 permutations) [32]. Weighted bubble plots were also constructed to graphically represent the fitted meta-regression lines.

Finally, secondary analyses were carried out to test the stability and reliability of meta-analysis results. Therefore, sensitivity analyses were carried out to explore the influence individual primary-level studies on the pooled estimates [33]. For this, the meta-analyses were repeated sequentially, omitting one study at a time (“leave-one-out” method). Furthermore, funnel plots were constructed to evaluate small-study effects, such as publication bias [34]. In addition, the Egger [35] regression test was applied performing a linear regression of the effect estimates on their standard errors, weighting by 1/(variance of the effect estimate), considering a *p*_Egger_ value of < 0.10 as significant. In addition, trying to confirm the absence of small-study effects, a nonparametric “trim and fill” method was used to identify and potentially correct the funnel plot asymmetry [36]. The statistical analysis was designed by PRG and executed by TDPC, using Stata software (version 16.1, Stata Corp, USA).

### Validation of methodological quality

Two independent authors (PRG and TDPC) critically designed and validated the methodology followed in this systematic review and meta-analysis using AMSTAR2 tool [19], created as an instrument to develop, evaluate, and validate high-quality systematic reviews through 16 items (the 16-item checklist is listed in the Appendix, pp. 62–65). An overall rating is obtained based on weaknesses in critical domains (i.e., items 2, 4, 7, 9, 11, 13, and 15) and noncritical domains. The overall confidence on the methodology of the systematic review is rated in one of the four levels: “High,” “Moderate,” “Low,” and “Critically low” (the full explanation is also listed in the Appendix, p. 66).

## Results

### Literature search

**Fig. 1:**
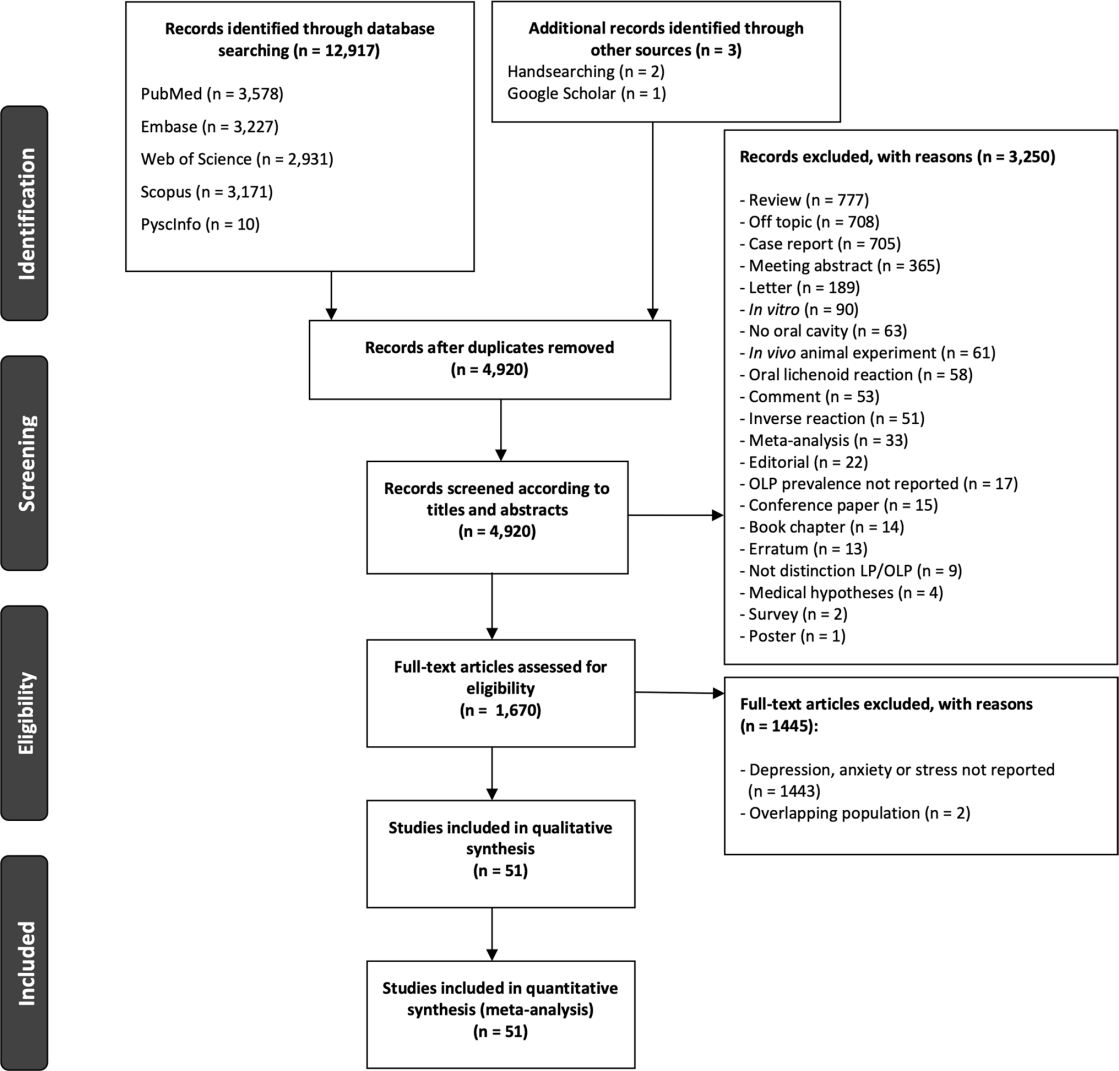
Flow diagram showing the identification and selection process of studies that address the prevalence of psychological disorders among OLP patients

The flow diagram (Fig. 1) illustrates the results of the study selection process. We identified a total of 12,917 records published before January 2021 (Appendix Table 1, p. 5): 3,578 from PubMed, 3,227 from Embase, 2,931 from Web of Science, 3,171 from Scopus, 10 from PsycInfo, and 3 from handsearching methods (2 from the bibliographic reference lists [37, 38] and one from Google Scholar [6]). After removal of duplicate records, 4,925 were potentially eligible. Once the titles and abstracts had been screened, 1,670 studies were evaluated in full-text, of which 1,445 studies did not comply with the inclusion criteria. Finally, 51 studies were included in the qualitative and quantitative analysis (references for included and excluded studies—with their reasons for exclusion reasons—are listed in the Appendix, pp. 69–73).

### Study characteristics

Table 1 summarizes the general characteristics of the 51 meta-analyzed studies, which recruited 6,815 patients. Supplementary Table 2 displays in more detail the characteristics and variables collected (Appendix Table 2, pp. 6–8).

**Table 1:** Characteristics of the studies included in the meta-analysis

	All studies	Depression	Anxiety	Stress
Total studies	51 studies	33 studies	31 studies	24 studies
Sample size				
Total no. of patients	6,815	4,031	3,336	3,450
Range	9–803	9–803	9–600	9–723
Publication year	1992–2021	1995–2021	1993–2021	1992–2020
Geographic area				
Asia	15 studies (4 countries)	10 studies (3 countries)	9 studies (2 countries)	9 studies (2 countries)
Europe	25 studies (13 countries)	16 studies (10 countries)	16 studies (9 countries)	9 studies (8 countries)
North America	4 studies (1 country)	3 studies (1 country)	1 studies (1 country)	2 studies (1 country)
South America	5 studies (2 countries)	3 studies (2 countries)	4 studies (2 countries)	2 studies (2 countries)
Global	2 studies (2 countries)	1 studies (1 country)	1 studies (1 country)	2 studies (2 countries)
Total	3 continents (22 countries)	3 continents (17 countries)	3 continents (15 countries)	3 continents (15 countries)
Specialist implied in diagnosis				
Psychologist	2 studies (161 patients)	2 studies (161 patients)	2 studies (161 patients)	2 studies (161 patients)
Psychiatrist	3 studies (169 patients)	3 studies (169 patients)	2 studies (102 patients)	——
Oral medicine-pathologist/dentist/dermatologist	46 studies (6,485 patients)	28 studies (3,701 patients)	27 studies (3,073 patients)	22 studies (3,289 patients)
Suspicion methods				
Anamnesis	8 studies (1,551 patients)	4 studies (948 patients)	2 studies (117 patients)	4 studies (558 patients)
BDI-II	2 studies (161 patients)	2 studies (161 patients)	——	——
CES-D	1 study (91 patients)	1 study (91 patients)	——	——
DASS-21	5 studies (163 patients)	5 studies (163 patients)	5 studies (163 patients)	5 studies (163 patients)
HADS	6 studies (503 patients)	6 studies (503 patients)	5 studies (458 patients)	1 study (49 patients)
HAM-A	5 studies (703 patients)	——	5 studies (703 patients)	——
HAM-D	4 studies (670 patients)	4 studies (670 patients)	——	——
PGWBI	1 study (67 patients)	——	1 study (67 patients)	——
PSQ	1 study (49 patients)	——	——	1 study (49 patients)
PSS-10	2 studies (302 patients)	——	——	2 studies (302 patients)
SAS	2 studies (274 patients)	——	2 studies (274 patients)	——
SDS	6 studies (348 patients)	1 study (100 patients)	—	——
STAI	6 studies (348 patients)	——	6 studies (348 patients)	——
Test of recent experience	1 study (9 patients)	——	——	1 study (9 patients)
WCQ	1 study (112 patients)	——	——	1 study (112 patients)
Multiple	3 studies (160 patients)	2 studies (115 patients)	1 study (45 patients)	——
Not described	16 studies (3,118 patients)	8 studies (1,280 patients)	4 studies (1,101 patients)	9 studies (2,208 patients)

Thirty-three studies (4,031 patients) reported data on the prevalence of depression in OLP patients. Regarding the prevalence by continents, 10 studies (441 patients) took place in Asia, 16 (2,902 patients) in Europe, 3 (170 patients) in North America, 3 (148 patients) in South America, and only one multicentric across various continents. Besides, prevalence by depression suspicion method was also performed: 6 studies (503 patients) diagnosed this disorder using *hospital and anxiety depression scale* (HADS), 5 studies (163 patients) by *depression, anxiety and stress scale-21 items* (DASS-21), 4 studies (948 patients) by anamnesis, 4 studies (670 patients) by *Hamilton Depression Rating Scale* (HAM-D), 2 studies (161 patients) by *Beck depression inventory II* (BDI-II), 1 study (100 patients) by *Zung Self-Rating Depression Scale* (SDS), and another study (91 patients) by *Center for Epidemiological Studies–Depression Scale* (CES-D). However, 8 studies (1,280 patients) did not describe how the suspicion was made and 2 studies (115 patients) used multiple tests. Depression was diagnosed in collaboration with a psychologist in 2 studies (161 patients), with a psychiatrist in 3 studies (169 patients), and with the rest of specialists—including dentists, dermatologists, and/or oral medicine/pathologists—in 28 studies (3,701 patients).

Thirty-one studies (3,336 patients) reported data on the prevalence of anxiety in OLP patients. With regard to the prevalence by continents, 9 studies (535 patients) took place in Asia, 16 (2,236 patients) in Europe, 1 (10 patients) in North America, 4 (185 patients) in South America, and only one multicentric across various continents. In addition, prevalence by anxiety suspicion method was also performed: 6 studies (348 patients) by *State-Trait Anxiety Inventory* (STAI), 5 (703 patients) by *Hamilton Anxiety Rating Scale* (HAM-A), another 5 (458 patients) by HADS, and further 5 (163 patients) by DASS-21 test. Moreover, 2 studies (117 studies) diagnosed this disorder by anamnesis and 2 more (274 patients) by Zung Self-Rating Anxiety Scale (SAS). However, 4 studies (1,101 patients) did not describe how the suspicion was made and one study (45 patients) used multiple tests. Anxiety was diagnosed in collaboration with a psychologist in 2 studies (161 patients), by a psychiatrist in another 2 studies (102 patients), and by the rest of specialists—including dentists, dermatologists, and/or oral medicine/pathologists—in 27 studies (3,073 patients).

Twenty-four studies (3,450 patients) reported data on the prevalence of stress in OLP patients. Regarding the prevalence by continents, 9 studies (527 patients) took place in Asia, another 9 (1,691 patients) in Europe, 2 (768 patients) in North America, further 2 (30 patients) in South America, and two multicentric across various continents. Moreover, prevalence by stress suspicion method was also performed: 5 studies (163 patients) by DASS-21 test, 4 (558 patients) by anamnesis, 2 (302 patients) by *Perceived Stress Scale* (PSS-10) and *Ways of Coping Questionnaire* (WCQ), HADS, *General Perceived Stress Questionnaire* (PSQ), and Test of Recent Experience were used in one study (112, 49, 49 and 9 patients) respectively. Stress was diagnosed in collaboration with a psychologist in 2 studies (161 patients) and by the rest of specialists—including dentists, dermatologists, and/or oral medicine/pathologists—in 22 studies (3,289 patients).

### Qualitative analysis

**Fig. 2:**
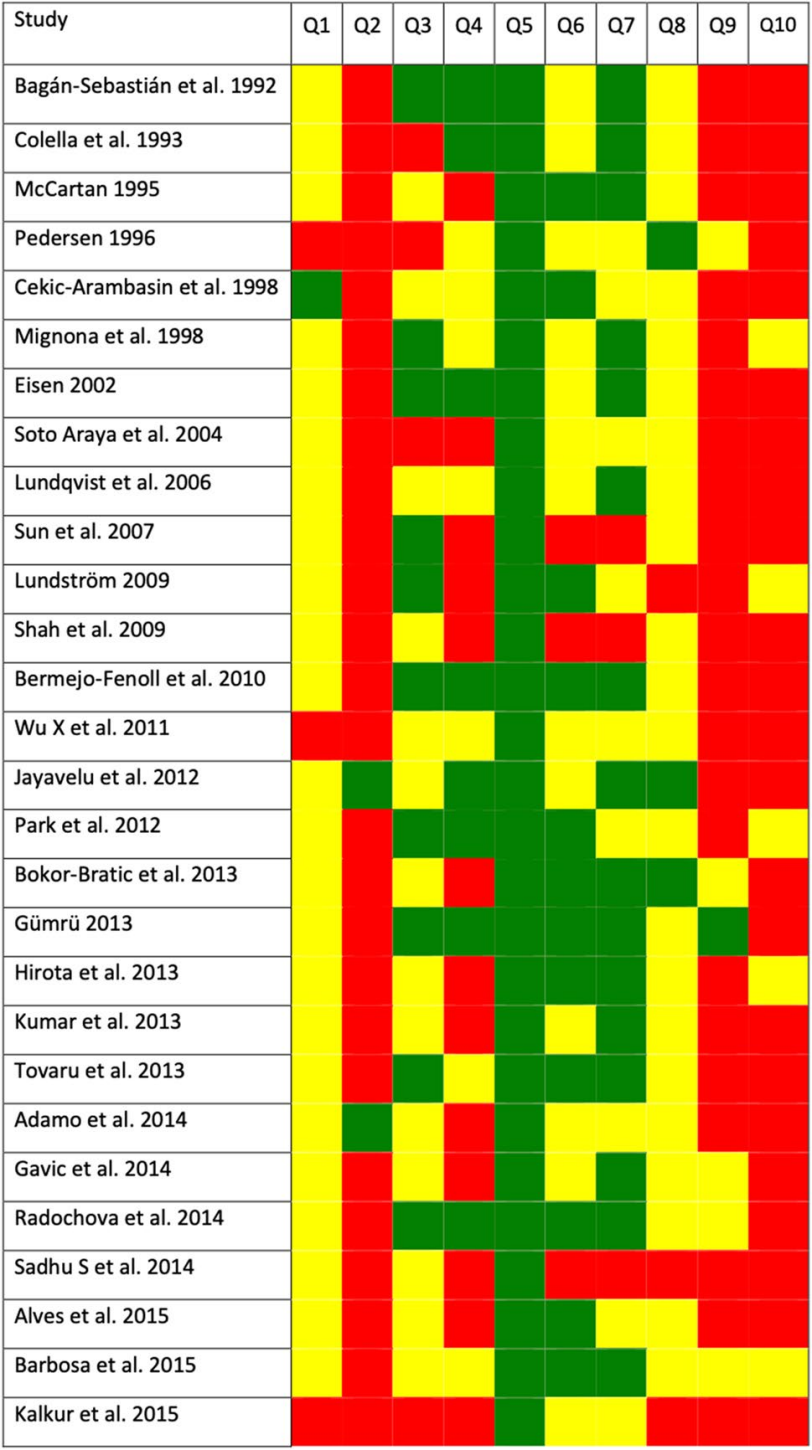
Quality plot graphically representing the risk of bias in individual studies, critically appraising ten domains, using a method specifically designed for systematic reviews addressing questions of prevalence (developed by the Joanna Briggs Institute, University of Adelaide, South Australia). Green, low risk of potential bias; yellow, moderate; red, high

According to our risk of bias (RoB) analysis, all the studies were not conducted with the same scrupulousness, being the items Q2, Q9, and Q10, and those with the highest risk of bias (Fig. 2). The Q2 item investigates whether the studies recruited patients adequately, not reporting most of them random sampling methods from the study population. The Q9 item targets biases due to the lack of control of potentially confounding factors in the studies (design, measurement, and/or communication). The Q10 item assesses whether the relevant data from the study subpopulations (sex, age, alcohol and tobacco consumption) were reported appropriately.

### Quantitative analysis (meta-analysis)

**Fig. 3:**
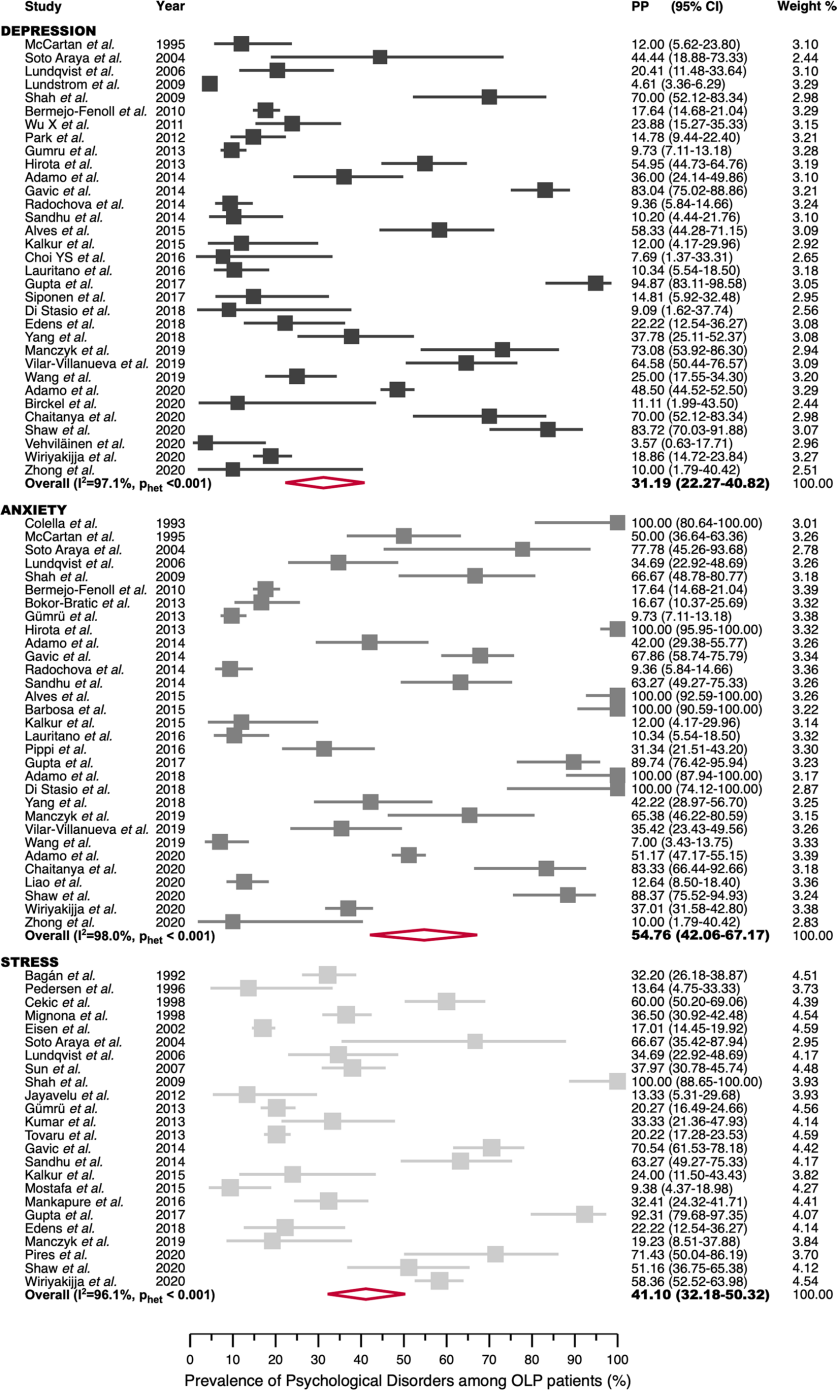
Forest plot graphically representing the prevalence of depression, anxiety, and stress among OLP patients

The results of the meta-analyses were graphically depicted in forest plots (Fig. 3, Appendix) and detailed in Table 2.

**Table 2:** Prevalence and magnitude of association of depression in patients with OLP and associated factors

	Sample size (n)		Statistical		Pooled data		Heterogeneity		
Meta-analyses	Studies	Patients	Model	Method	ES (95% CI)	*P value*	*P*_*het*_	*I*^*2*^ (%)	*Appendix*^*a*^
Magnitude of association^a^	16	1,833	REM	D-L	OR = 6.150(2.723–13.891)	< 0.001	< 0.001	85.40	Figure S1,p9
Prevalence^c^	33	4,031	REM	D-L	PP = 31.19%(22.27–40.82)	──	< 0.001	97.14	
Prevalence by continents^d^						< 0.001^e^			Figure S2,p10
Asia	10	441	REM	D-L	PP = 43.35%(22.91–64.97)		< 0.001	95.02	
Europe	16	2,902	REM	D-L	PP = 25.19%(13.79–38.52)		< 0.001	97.97	
North America	3	170	REM	D-L	PP = 15.51%(10.09–21.70)		──	──	
South America	3	148	REM	D-L	PP = 55.58%(47.20–63.81)		──	──	
Global	1	370	REM	D-L	PP = 9.73%(7.11–13.18)		──	──	
Prevalence by depression suspicion methods^d^						< 0.001^e^			Figure S3,p11
Anamnesis	4	948	REM	D-L	PP = 9.73%(2.81–19.56)		< 0.001	82.60	
BDI-II	2	161	REM	D-L	PP = 65.32%(57.72–72.55)		──	──	
CES-D	1	91	REM	D-L	PP = 54.95%(44.73–64.76)		──	──	
DASS-21	5	163	REM	D-L	PP = 68.75%(38.76–92.32)		< 0.001	93.28	
HADS	6	503	REM	D-L	PP = 33.14%(15.85–18.62)		< 0.001	93.71	
HAM-D	4	670	REM	D-L	PP = 37.13%(22.83–52.55)		0.02	70.77	
SDS	1	100	REM	D-L	PP = 25.00%(17.55–34.30)		──	──	
Multiple	2	115	REM	D-L	PP = 37.60%(28.88–46.74)		──	──	
Not described	8	1,280	REM	D-L	PP = 11.18%(7.61–15.24)		0.01	60.61	
Prevalence by specialist implied in diagnosis of depression^d^						< 0.001^e^			Figure S4,p12
Psychologist	2	161	REM	D-L	PP = 65.32%(57.72–72.55)		──	──	
Psychiatrist	3	169	REM	D-L	PP = 30.20%(8.17–58.05)		──	──	
Oral medicine-pathologist/dentist/dermatologist	28	3,701	REM	D-L	PP = 29.77%(20.84–39.50)		< 0.001	96.92	
Prevalence by publication language^d^						0.39^e^			Figure S5,p13
English	30	3,946	REM	D-L	PP = 31.72%(22.34–41.88)		< 0.001	97.40	
Other	3	85	REM	D-L	PP = 23.74%(12.46–36.90)		──	──	
Prevalence by sex^d^						0.92^e^			Figure S6,p14
Females	9	1,122	REM	D-L	PP = 18.96%(4.96–37.93)		< 0.001	93.79	
Males	9	1,122	REM	D-L	PP = 14.32%(0.00–41.75)		0.06	86.27	
Prevalence. Univariable meta-regression^f^									
Sex (% OLP females)	32	4,006	Random-effects meta-regression		Coef = -0.0029(-0.0109 to 0.0050)	0.47 ± 0.016^ g^	het_explained_ = -2.52%^h^		Figure S7,p.15
Age (mean age of OLP patients)	31	3,967	Random-effects meta-regression		Coef = -0.0133(-0.0275 to 0.0009)	0.07 ± 0.009^ g^	het_explained_ = 7.59%^h^		Figure S8,p.16
Tobacco (% OLP smokers)	12	2,534	Random-effects meta-regression		Coef = 0.0025(-0.0123 to 0.0174)	0.74 ± 0.014^ g^	het_explained_ = -9.56%^h^		Figure S9,p.17
Alcohol (% OLP drinkers)	6	1,447	Random-effects meta-regression		Coef = -0.0045(-0.0211 to 0.0120)	0.43 ± 0.016^ g^	het_explained_ = -13.92%^h^		Figure S10, p.18
Red lesions (%OLP patients)	17	2,074	Random-effects meta-regression		Coef = -0.0019(-0.0057 to 0.0019)	0.30 ± 0.015^ g^	het_explained_ = -0.59%^h^		Figure S11, p.19
Anxiety	25	2,924	Random-effects meta-regression		Coef = 0.0065(0.0040 to 0.0089)	< 0.001^ g^	het_explained_ = 75.25%^h^		Figure S12, p.20
Stress	13	1,671	Random-effects meta-regression		Coef = 0.0069(0.0000 to 0.0138)	0.05 ± 0.008^ g^	het_explained_ = 34.82%^h^		Figure S13, p.21
Year	33	4,031	Random-effects meta-regression		Coef = 0.0122(-0.0055 to 0.0300)	0.13 ± 0.011^ g^	het_explained_ = 7.17%^h^		Figure S14, p.22
HDI	33	4,031	Random-effects meta-regression		Coef = -1.4243(-0.2.2701 to -0.2.2701)	0.004 ± 0.002^ g^	het_explained_ = 33.02%^h^		Figure S15, p.23
RoB	33	4,031	Random-effects meta-regression		Coef = -0.1759(-0.5084 to 0.1565)	0.32 ± 0.015^ g^	het_explained_ = 2.65%^h^		Figure S16,p.24
Prevalence. Multivariable meta-regression^f^									
Anxiety	11	1,033	Random-effects meta-regression		Coef = 0.0107(0.0025 to 0.0188)	0.02 ± 0.008^ g^	het_explained_ = 72.59%^h^		Figure S12, p.20
Stress			Random-effects meta-regression		Coef = -0.0006(-0.0090 to 0.0079)	0.87 ± 0.002^ g^			Figure S13, p.21
HDI			Random-effects meta-regression		Coef = 0.0728(-0.1.437 to 1.1470)	0.90 ± 0.001^ g^			Figure S15, p.23

Abbreviations: *Stat*., statistical; *Wt*, method of weighting; *PP*, pooled proportion; *CI*, confidence intervals; *REM*, random-effects model; *D-L*, DerSimonian and Laird method; OLP, oral lichen planus; *BDI-II*, Beck depression inventory II; *CES-D*, Center for Epidemiological Studies–Depression Scale; *DASS-21*, depression, anxiety and stress scale-21 items; *HADS*, hospital and anxiety depression scale; *HAM-D*, Hamilton Depression Rating Scale; *SDS*, Zung Self-Rating Depression Scale

^a^Magnitude of association meta-analyses

^b^More information in the appendix

^c^Proportion meta-analyses

^d^Proportion meta-analyses (subgroup analyses)

^e^Test for between-subgroup differences

^f^Effect of study covariates on the prevalence of depression, anxiety, or stress among OLP patients. A meta-regression coefficient > 0 indicates a greater impact of covariates on the prevalence of mental disorders in patients with OLP

^g^*P* value ± standard error after 10,000 permutations based on Monte Carlo simulation

^h^Proportion of between-study variance explained (adjusted *R*^2^ statistic) using the residual maximum likelihood (REML) method. A negative number for proportion of heterogeneity explained reflects no heterogeneity explained

^i^Adjusted model for prevalence of depression in OLP (number of comparisons = 11); adjusted *R*^2^ statistic = 72.59%; joint test for all covariates *F* = 0.0225, *p* = 0.0107

### Depression

#### Prevalence of depression in OLP patients

The pooled proportion (PP) was 31.19% (95% CI = 22.27–40.82), with a high degree of heterogeneity (*I*^2^ = 97.14%, *p* < 0.001) (Fig. 3).

### Magnitude of association between depression and OLP

Patients with OLP showed a significantly higher frequency of depression than the general population control group (OR = 6.15, 95% CI = 2.72–13.89, *p* < 0.001; Appendix p. 9).

### Subgroup meta-analyses and meta-regressions

**Fig. 4:**
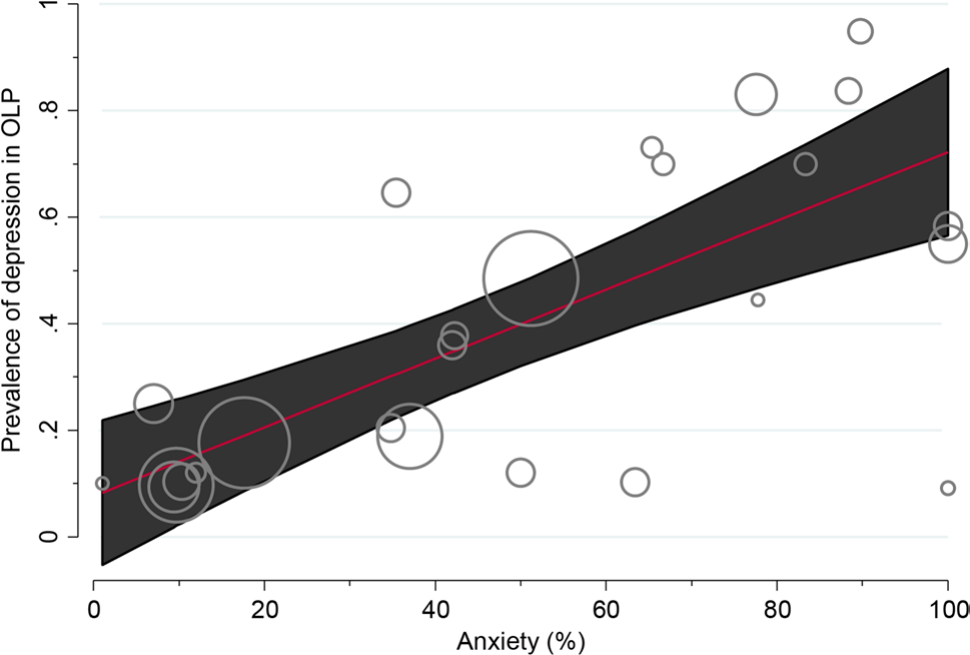
Bubble plot graphically representing the potential effect of the covariate anxiety (expressed as the percentage of patients with signs of anxiety, in x-axis) on the prevalence of depression among OLP patients (expressed as proportions, in y-axis). The fitted meta-regression line (red line) was depicted with their corresponding 95% confidence intervals (black area), together with bubbles (grey circles) representing the estimates from primary-level studies (sized according to the precision of each estimate, the inverse of its within-study variance, in a z-axis)

In the stratified analyses (Appendix pp. 10–14), we found significant differences between continents (*p* < 0.001), finding the highest prevalence in South America (PP = 55.58%, 95% CI = 47.20–63.81) and Asia (PP = 43.35%, 95% CI = 22.91–64.97). We also observed significant results between the tests used to diagnose depression. After adjustment in a multivariable meta-regression model, only anxiety maintained the statistical significance (*p* = 0.02), probably being the most influential covariate associated with the OLP depression comorbidity (Table 2; Fig. 4).

### Anxiety

#### Prevalence of anxiety in OLP patients

The estimated PP was 54.76% (95% CI = 42.06–67.17), with a high degree of heterogeneity (*I*^2^ = 98.00%, *p* < 0.001) (Fig. 3).

### Magnitude of association between anxiety and OLP

Patients with OLP showed a significantly higher frequency of anxiety than the general population control group (OR = 3.51, 95% CI = 2.10–5.85, *p* < 0.001; Appendix p. 25).

### Subgroup meta-analyses and meta-regressions

In the subgroup analyses (Appendix pp. 26–30), we found significant differences between continents (*p* < 0.001); South America outnumbered the rest of continents with the highest prevalence (PP = 99.88%, 95% CI = 95.71–100.00). Moreover, significant differences were observed between the tests used to diagnose anxiety. The prevalence did not vary significantly for the rest of the factors investigated (age, sex, tobacco and alcohol consumption) in the univariate meta-regression analyses (Appendix pp. 31–38) except for HDI (*p* = 0.03) (Table 3).

**Table 3:** Prevalence and magnitude of association of anxiety in patients with OLP and associated factors

	**Sample size (n)**		**Statistical**		**Pooled data**		**Heterogeneity**		
**Meta-analyses**	**Studies**	**Patients**	**Model**	**Method**	**ES (95% CI)**	***P-value***	***P***_***het***_	***I***^***2***^ **(%)**	***Appendix***^***a***^
Magnitude of association	17	1,941	REM	D-L	OR = 3.51(2.10–5.85)	< 0.001	< 0.001	63.30	Figure S17,p25
Prevalence^c^	31	3,336	REM	D-L	PP = 54.76% (42.06–67.17)	──	< 0.001	98.00	
Prevalence by continents^d^						< 0.001^e^			Figure S18,p26
Asia	9	535	REM	D-L	PP = 50.90% (25.26–76.29)		< 0.001	97.16	
Europe	16	2,236	REM	D-L	PP = 47.88% (35.02–60.88)		< 0.001	96.93	
North America	1	10	REM	D-L	PP = 10.00% (1.79–40.42)		──	──	
South America	4	185	REM	D-L	PP = 99.88% (95.71–100.00)		0.05	60.89	
Global	1	370	REM	D-L	PP = 9.73% (7.11–13.18)		──	──	
Prevalence by anxiety suspicion methods^d^						< 0.001^e^			Figure S19,p27
Anamnesis	2	117	REM	D-L	PP = 13.41% (8.70–18.91)		──	──	
DASS-21	5	163	REM	D-L	PP = 66.07% (36.74–90.17)		< 0.001	92.93	
HADS	5	458	REM	D-L	PP = 53.22% (36.90–69.20)		< 0.001	88.78	
HAM-A	5	703	REM	D-L	PP = 79.48% (50.76–98.13)		< 0.001	95.02	
PGWBI	1	67	REM	D-L	PP = 31.34% (21.51–43.20)		──	──	
SAS	2	274	REM	D-L	PP = 10.41% (7.01–14.36)		──	──	
STAI	6	348	REM	D-L	PP = 91.29% (66.16–100.00)		< 0.001	96.83	
Multiple	1	45	REM	D-L	PP = 42.22% (28.97–56.70)		──	──	
Not described	4	1,101	REM	D-L	PP = 11.40% (6.57–17.21)		< 0.001	79.76	
Prevalence by specialist implied in diagnosis of anxiety^d^						< 0.001^e^			Figure S20,p28
Psychologist	2	161	REM	D-L	PP = 57.99% (50.21–65.58)		──	──	
Psychiatrist	2	102	REM	D-L	PP = 100.00% (99.30–100.00)		──	──	
Oral medicine-pathologist/dentist/dermatologist	27	3,073	REM	D-L	PP = 50.18% (37.89–62.45)		< 0.001	97.62	
Prevalence by publication language^d^						< 0.001^e^			Figure S21,p29
English	29	3,311	REM	D-L	PP = 52.06% (39.22–64.78)		< 0.001	98.02	
Other	2	25	REM	D-L	PP = 96.13% (82.71–100.00)		──	──	
Prevalence by sex^d^						0.96^e^			Figure S22,p30
Females	5	192	REM	D-L	PP = 88.12% (59.09–100.00)		0.48	92.60	
Males	5	192	REM	D-L	PP = 93.29% (59.37–100.00)		0.37	76.08	
Prevalence. Univariable meta-regression.^f^									
Sex (% OLP females)	30	3,311	random-effects meta-regression		Coef = -.0006 (-.0130 to .0118)	0.92 ± 0.008^ g^	het_explained_ = -3.92%^h^		Figure S23, p.31
Age (mean age of OLP patients)	29	3,272	random-effects meta-regression		Coef = -.0028 (-.0224 to .0168)	0.77 ± 0.013^ g^	het_explained_ = -3.93%^h^		Figure S24, p.32
Tobacco (% OLP smokers)	10	1,803	random-effects meta-regression		Coef = -.0020 (-.0132 to .0093)	0.70 ± 0.015^ g^	het_explained_ = -13.86%^h^		Figure S25, p.33
Alcohol (% OLP drinkers)	7	1,520	random-effects meta-regression		Coef = -.0003 (-.0125 to .0120)	0.98 ± 0.004^ g^	het_explained_ = -25.77%^h^		Figure S26, p.34
Red lesions (%OLP patients)	17	2,162	random-effects meta-regression		Coef = -.0015 (-.0079 to .0049)	0.68 ± 0.015^ g^	het_explained_ = -6.66%^h^		Figure S27, p.35
Year	31	3,336	random-effects meta-regression		Coef = -.0060 (-.0258 to .0137)	0.62 ± 0.015^ g^	het_explained_ = -2.98%^h^		Figure S28, p.36
HDI	31	3,336	random-effects meta-regression		Coef = -1.2944 (-2.4707 to -.1180)	0.03 ± 0.006^ g^	het_explained_ = 18.00%^h^		Figure S29, p.37
RoB	31	3,336	random-effects meta-regression		Coef = -.3563 (-.7738 to .0611)	0.09 ± 0.009^ g^	het_explained_ = 9.31%^h^		Figure S30, p.38

Abbreviations: *Stat*., statistical; *Wt*, method of weighting; *PP*, pooled proportion; *CI*, confidence intervals; *REM*, random-effects model; *D-L*, DerSimonian and Laird method; *OLP*, oral lichen planus; *DASS-21*, depression, anxiety and stress scale-21 items; *HADS*, hospital and anxiety depression scale; *RoB*, Risk of Bias; *HAM-A*, Hamilton Anxiety Rating Scale; *PGWBI*, Psychologial General Well-Being Index; *SAS*, Zung Self-Rating Anxiety Scale; *STAI*, State-Trait Anxiety Inventory

^a^Magnitude of association meta-analyses

^b^More information in the appendix

^c^Proportion meta-analyses

^d^Proportion meta-analyses (Subgroup analyses)

^e^Test for between-subgroup differences

^f^Effect of study covariates on the prevalence of depression, anxiety or stress among OLP patients. A meta-regression coefficient > 0 indicates a greater impact of covariates on the prevalence of mental disorders in patients with OLP

^g^*P* value ± standard error after 10,000 permutations based on Monte Carlo simulation

^h^Proportion of between-study variance explained (adjusted R^2^ statistic) using the residual maximum likelihood (REML) method. A negative number for proportion of heterogeneity explained reflects no heterogeneity explained

### Stress

#### Prevalence of stress in OLP patients

The PP was 41.10% (95% CI = 32.18–50.32), with a significant degree of heterogeneity (*I*^2^ = 96.11%, *p* < 0.001) (Fig. 3).

### Magnitude of association between stress and OLP

Patients with OLP showed a significantly higher frequency of anxiety than the general population control group (OR = 3.64, 95% CI = 1.48–8.94, *p* = 0.005; Appendix p. 39).

### Subgroup meta-analyses and meta-regressions

In the stratified analyses (Appendix pp. 40–44), we found significant differences between continents (*p* < 0.001), finding the highest prevalence in South America. Prevalence did not vary significantly for the rest of the factors investigated (age, sex, tobacco, alcohol, and HDI) in the univariate meta-regression analyses (Appendix pp. 45–52) (Table 4).

**Table 4:** Prevalence and magnitude of association of stress in patients with OLP and associated factors

	**Sample size (n)**		**Statistical**		**Pooled data**		**Heterogeneity**		
**Meta-analyses**	**Studies**	**Patients**	**Model**	**Method**	**ES (95% CI)**	***P-value***	***P***_***het***_	***I***^***2***^ **(%)**	***Appendix***^***a***^
Magnitude of association	8	956	REM	D-L	OR = 3.64(1.48–8.94)	0.005	< 0.001	75.40	Figure S31,p39
Prevalence^c^	24	3,450	REM	D-L	PP = 41.10% (32.18–50.32)	──	< 0.001	96.11	
Prevalence by continents^d^						< 0.001^e^			Figure S32,p40
Asia	9	527	REM	D-L	PP = 52.15% (32.79–71.20)		< 0.001	94.72	
Europe	9	1,691	REM	D-L	PP = 38.52% (25.53–52.38)		< 0.001	96.45	
North America	2	768	REM	D-L	PP = 17.00% (14.38–19.79)		──	──	
South America	2	30	REM	D-L	PP = 70.28% (51.86–86.15)		──	──	
Global	2	434	REM	D-L	PP = 18.34% (14.80–22.17)		──	──	
Prevalence by stress suspicion methods^d^						< 0.001^e^			Figure S33,p41
Anamnesis	4	558	REM	D-L	PP = 13.41% (8.70–18.91)		──	──	
DASS-21	5	163	REM	D-L	PP = 66.07% (36.74–90.17)		< 0.001	92.93	
HADS	1	49	REM	D-L	PP = 53.22% (36.90–69.20)		< 0.001	88.78	
PSQ	1	49	REM	D-L	PP = 79.48% (50.76–98.13)		< 0.001	95.02	
PSS-10	2	302	REM	D-L	PP = 31.34% (21.51–43.20)		──	──	
Test of Recent Experience	1	9	REM	D-L	PP = 10.41% (7.01–14.36)		──	──	
WCQ	1	112	REM	D-L	PP = 91.29% (66.16–100.00)		< 0.001	96.83	
Not described	9	2,208	REM	D-L	PP = 11.40% (6.57–17.21)		< 0.001	79.76	
Prevalence by specialist implied in diagnosis of stress^d^						0.001^e^			Figure S34,p42
Psychologist	2	161	REM	D-L	PP = 59.98% (52.23–67.49)		──	──	
Oral medicine-pathologist/dentist/dermatologist	22	3,289	REM	D-L	PP = 39.90% (30.96–49.18)		< 0.001	95.92	
Prevalence by publication language^d^						0.142^e^			Figure S35,p43
English	23	3,441	REM	D-L	PP = 40.40% (31.43–49.70)		< 0.001	96.25	
Other	1	9	REM	D-L	PP = 66.67% (35.42–87.94)		──	──	
Prevalence by sex^d^						< 0.001^e^			Figure S36,p44
Females	2	289	REM	D-L	PP = 22.46% (16.34–29.19)		──	──	
Males	2	289	REM	D-L	PP = 53.22% (43.55–62.79)		──	──	
Prevalence. Univariable meta-regression.^f^									
Sex (% OLP females)	22	3,267	random-effects meta-regression		Coef = -.0004 (-.0078 to .0069)	0.89 ± 0.010^ g^	het_explained_ = -7.13%^h^		Figure S37, p.45
Age (mean age of OLP patients)	19	3,153	random-effects meta-regression		Coef = -.0065 (-.0247 to .0118)	0.49 ± 0.016^ g^	het_explained_ = -8.56%^h^		Figure S38, p.46
Tobacco (% OLP smokers)	13	1,210	random-effects meta-regression		Coef = -.0046 (-.0146 to .0054)	0.35 ± 0.015^ g^	het_explained_ = -5.86%^h^		Figure S39, p.47
Alcohol (% OLP drinkers)	5	847	random-effects meta-regression		Coef = -.0016 (-.0194 to .0163)	0.75 ± 0.014^ g^	het_explained_ = -40.88%^h^		Figure S40, p.48
Red lesions (%OLP patients)	15	2,839	random-effects meta-regression		Coef = -.0013 (-.0051 to .0024)	0.44 ± 0.016^ g^	het_explained_ = -9.05%^h^		Figure S41, p.49
Year	24	3,450	random-effects meta-regression		Coef = -.0044 (-.0082 to .0170)	0.48 ± 0.016^ g^	het_explained_ = -3.36%^h^		Figure S42, p.50
HDI	24	3,450	random-effects meta-regression		Coef = -.5188 (-1.4102 to .3726)	0.241 ± 0.014^ g^	het_explained_ = 0.68%^h^		Figure S43, p.51
RoB	24	3,450	random-effects meta-regression		Coef = -.2666 (-.5855 to .0522)	0.11 ± 0.001^ g^	het_explained_ = 10.71%^h^		Figure S44, p.52

Abbreviations: *Stat*., statistical; *Wt*, method of weighting; *PP*, pooled proportion; *CI*, confidence intervals; *REM*, random-effects model; *D-L*, DerSimonian and Laird method; *OLP*, oral lichen planus; *DASS-21*, depression, anxiety and stress scale-21 items; *HADS*, hospital and anxiety depression scale; *HDI*, Human Development Index; *RoB*, Risk of Bias; *PSQ*, General Perceived Stress Questionnaire; *PSS-10*, Perceived Stress Scale; *WCQ*, Ways of Coping Questionnaire

^a^Magnitude of association meta-analyses

^b^More information in the appendix

^c^Proportion meta-analyses

^d^Proportion meta-analyses (Subgroup analyses)

^e^Test for between-subgroup differences

^f^Effect of study covariates on the prevalence of depression, anxiety or stress among OLP patients. A meta-regression coefficient > 0 indicates a greater impact of covariates on the prevalence of mental disorders in patients with OLP

^g^*P* value ± standard error after 10,000 permutations based on Monte Carlo simulation

^h^Proportion of between-study variance explained (adjusted *R*^2^ statistic) using the residual maximum likelihood (REML) method. A negative number for proportion of heterogeneity explained reflects no heterogeneity explained

### Quantitative evaluation (secondary analyses)

#### Sensitivity analysis

The consecutive repetition of meta-analyses using the “leave-one-out” method (Appendix, pp. 56–61) did not vary the overall results considerably. Hence, the reported pooled estimations are not influenced by a specific primary-level study.

### Analysis of small‐study effects

Egger’s regression test indicated statistically significant asymmetry for the prevalence of depression, anxiety, and stress in OLP patients (*p*_Egger_ = 0.09, 0.01, and 0.02, respectively). Funnel plots (Appendix pp. 53–55) appeared to be slightly asymmetric for the studies plotted at the bottom, singularly for anxiety variable; however, due to a considerable degree of inter-study heterogeneity, the visual inspection analysis was complex. Nevertheless, the nonparametric trim and fill method did not detect the presence of unpublished studies, so the final estimates were not adjusted based on imputation techniques for missing studies. In summary, the presence of small-study effects was suspected, but publication bias was potentially ruled out.

### Validation of methodological quality

The methods applied in this systematic review and meta-analysis were implemented, critically appraised, and validated using AMSTAR2 [39], obtaining an overall rating of “high” (15 out of 16 points) (the checklist, explanation, and scoring table are included in the Appendix, pp. 62–66).

## Discussion

The results of our systematic review and meta-analysis show a strong association between OLP and psychological disorders, i.e., depression, anxiety, and stress. Patients with OLP present a risk of suffering from depression (*p* < 0.001), anxiety (*p* < 0.001), and stress (*p* < 0.005) significantly higher than the general population, with a prevalence of depression of 31.19%, anxiety of 54.76%, and stress of 41.10% among OLP patients. Our results were derived from the analysis of 51 studies that collected information from 6,815 patients with OLP. A meta-analysis on the subject that included patients with OLP [12] has recently been published, reporting a prevalence of depression and anxiety of 26% and 27% of the cases, respectively. It must be noted that this meta-analysis [12] presents critically low methodological quality, according to AMSTAR2, which is essentially due to a significant selection bias derived from having designed a low-sensitive search strategy that only identified 16 studies for analysis—a number of studies considerably lower than the 51 studies included in our present meta-analysis. Therefore, the results of Jalenque et al. [12] seem incomplete.

Our results also interestingly reveal that the studies reporting the higher prevalences of depression also report the higher frequencies of anxiety (*p* = 0.001), which seems to indicate a comorbidity among depression, anxiety, and OLP. Specialists involved in the diagnosis and treatment of OLP, especially dentists—as they are in the first line of care for patients with oral diseases—must be aware of these important comorbidities in order to implement appropriate measures that allow patients with OLP to receive the specialized care required for these emotional disorders. As previously mentioned, it may not be straightforward for a dentist to bring out psychological disorders in patients with OLP, whose main reason for consultation is the presence of oral mucosal lesions. In the experience of the authors (MAGM, SW), patients often do not disclose these conditions out of shame, feelings of stigmatization or fear of family incomprehension, and the adverse effects of psychotropic drugs. Occasionally, patients consider their emotional disorders as non-pathological situations derived from stress or everyday problems in life. Finally, sometimes dentists may not feel themselves authorized or trained to identify and refer patients to a psychiatrist or psychologist. The treatment of psychological disorders is a relevant issue since many of them considerably decrease the quality of life of the patient, which in itself can be notably deteriorated by OLP. Furthermore, although there is no scientific evidence on the subject, hypothetically, in some patients, the control of psychological disorders could also improve the OLP control, since it is frequent to note the worsening of OLP symptoms in periods in which the emotional symptoms increase. The training and insight of the dentist will make it possible to suspect the presence of emotional factors, and through an anamnesis carried out with subtlety, the patient will recognize the existence of these abnormalities.

According to our qualitative evaluation using a specific critical appraisal checklist designed by the Joanna Briggs Institute for systematic reviews addressing prevalence questions, although our included primary-level studies had similar study design, all were not conducted with the same rigor. Most potential biases were caused by the failure considering three specific items (i.e., Q2, Q9, and Q10). To meet Q2, future studies should recruit study participants in an appropriate way, always reporting how sampling was performed, and preferably using random sampling methods. On the other hand, Q9 and Q10, respectively, target biases due to potentially confounding factors and non-identified subpopulations, both items sharing similarities. Future studies should be better designed, correctly measuring and clearly reporting data related to age, sex, OLP type and location of lesions, medical history, and tobacco/alcohol habits. Furthermore, studies do not report treatment for these conditions or if OLP patient relapses are precipitated by worsening of the emotional status of patients. On the other hand, future studies should also focus on these issues. On the other hand, we tested the influence of risk of bias on the overall results using meta-regression, and no significant differences were observed. The overall results do not depend on the influence of the subset of studies with lowest quality, increasing the quality of evidence of the results reported in our meta-analysis. We strongly encourage future studies assessing the prevalence of psychological disorders in OLP, to consider the recommendations given in this systematic review and meta-analysis to improve and standardize future research (Table 5).

**Table 5:** Recommendations for conducting future studies on OLP and mental disorders

1) Samples must be representative of the target population (i.e., OLP). Primary-level studies must clearly inform about the origin of the sample (general population, hospitals or specialized centers, dental schools, private offices)
2) OLP patients should be recruited in an appropriate way. The methods section should report how sampling was performed; random sampling from a population is strongly encouraged
3) An adequate OLP sample size is imperative to guarantee the representativeness of the population with OLP and to ensure a precise final estimate. Preliminary sample size calculation should be conducted to determine an adequate sample size
4) OLP subjects should be described in detail. Their demographic and clinicopathological characteristics should be registered during follow-up. Studies should include data related to sex, age, clinical appearance and location of the lesions, medical history, habits, and histopathological data of OLP Depression, anxiety, and stress should be measured in an objective way, using standard criteria. Specialists (i.e., psychologists and psychiatrists) should collaborate with dentists in future studies to make an appropriate diagnosis
5) Studies should report comprehensive data on follow-up periods and dropout rates. Long follow-up periods are encouraged. Studies should describe attempts to gather information on patients who dropped out, their features, and follow-up reasons
6) Studies must clearly report the OLP diagnostic criteria used, which should be agreed upon by groups of experts and in any case derived from scientific publications, preferably systematic reviews and meta-analyses (e.g., Gonzalez-Moles et al. 2020, [1]). It is recommended to include clinical and histopathological criteria in the diagnosis
7) It should be reported how the diagnosis was conducted (trained or educated authors involved, inter-agreement scoring [e.g., Cohen’s kappa statistic], more than one data collector, and justifications for diagnosis methods chosen and explicit methods)
8) The statistical analysis must be appropriate to achieve the objectives and supported by clear presentation of data. The reporting of prevalence and incidence estimates on depression, anxiety and/or stress should be accompanied by their confidence intervals
9) It is important to identify all potentially confounding factors (exclusion and/or differentiation of oral lichenoid reactions [by drugs or contact with dental materials], clear definitions and characterization of tobacco and alcohol consumption, sex and age of patients)
10) Potential subpopulations should be comprehensively described in a transparently way, preferably reporting individual patient data (geographical area, ethnia, sex, age, noxious habits, and singularly the prevalence of mental disorders and other systemic comorbidities)

Our systematic review and meta-analysis also presents some limitations that should be discussed. First, an inherent limitation of the included studies, as previously commented, was the lack of reporting of relevant datasets that limited the number of observations in secondary analyses (e.g., influence of sex, age, alcohol, tobacco, etc.). Future studies should report datasets in a more rigorous way—preferably individual patient data—given the clinical and methodological relevance of these variables. Second, we observed considerable inter-study heterogeneity. As stated in our study protocol, it was expected, and planned random-effects models were applied in all meta-analyses to account for heterogeneity. In addition, we conducted several stratified meta-analyses by selecting more homogeneous subgroups, identifying that factors such as geographic areas, specific questionnaires, and the participation of a psychologist or psychiatrist to reach mental disorders’ diagnosis constitute important explanatory sources of heterogeneity. Finally, we performed random-effects meta-regression analyses and applied the REML method to produce an adjusted *R*^2^ statistic, which estimates the proportion of the inter-study variance explained by covariates. This analysis showed that anxiety is a very relevant source of heterogeneity (approximately explaining 75.25% of heterogeneity), significantly associated with an increased prevalence of depression among OLP patients. Despite the above limitations, the robust nature of our systematic review and meta-analysis is remarkable, as evidenced by our careful process of identification and selection of studies (see flow diagram), where more than 10,000 registers were screened and more than 1,500 papers subject to full-text reading; the absence of restrictions by publication language or date limits; robust qualitative recommendations for future studies on this topic; and potential translational opportunities derived from our comprehensive statistical analysis.

In conclusion, OLP patients suffer depression, anxiety, and stress more frequently than the general population. The physicians involved in the management of OLP, especially dentists, should be aware of these comorbidities in order to implement the appropriate measures for their referral.

## Supplementary Information

Below is the link to the electronic supplementary material.

Supplementary file1 (PDF 6411 KB)
