# Supplementary material for: Depression, anxiety, and stress in oral lichen planus: a systematic review and meta-analysis
Source: Clin Oral Investig. 2021 Aug 30;26(2):1391–408. doi: 10.1007/s00784-021-04114-0 (PMC8816610; doi:10.1007/s00784-021-04114-0)
Supplement: Supplementary file 1 — Supplementary file1 (PDF 6411 KB) [file 784_2021_4114_MOESM1_ESM.pdf]

Appendix to the manuscript

Depression, anxiety and stress in oral lichen planus: a systematic review and meta-analysis

De Porras-Carrique T[a], González-Moles MA[a*], Warnakulasuriya S[b], Ramos-García P[a]

a- School of Dentistry, University of Granada. Biohealth Research Institute (IBS), Granada, Spain

b- Faculty of Dentistry, Oral and Craniofacial Sciences, King's College London. WHO Collaborating Centre for Oral Cancer, London, UK.

Corresponding Author: *Miguel Ángel González Moles

Oral Medicine Department, School of Dentistry, University of Granada,Granada, Paseo de Cartuja s/n, 18071 Granada, Spain.

Tel.:+34958243804; fax:+34958240908; E-mail: magonzal@ugr.es

Table of contents

1. Search strategy....5

2. Characteristics of analyzed studies....6

3. Depression meta-analyses....9

3.1 Magnitude of association between OLP and depression....9

3.2 Prevalence of depression among OLP patients. Subgroup meta-analyses....10

3.2.1 Prevalence of depression among OLP patients by continent....10

3.2.2 Prevalence of depression among OLP patients by diagnostic depression test....11

3.2.3 Prevalence of depression among OLP patients by specialist implied in diagnosis of depression....12

3.2.4 Prevalence of depression among OLP patients by publication language....13

3.2.5 Prevalence of depression among OLP patients by sex....14

3.3 Prevalence of depression among OLP patients. Meta-regression analyses....15

3.3.1 Effect of sex on the prevalence of depression among OLP patients.....15

3.3.2 Effect of age on the prevalence of depression among OLP patients....16

3.3.3 Effect of tobacco on the prevalence of depression among OLP patients....17

3.3.4 Effect of alcohol on the prevalence of depression among OLP patients....18

3.3.5 Effect of OLP type on the prevalence of depression among OLP patients....19

3.3.6 Effect of anxiety on the prevalence of depression among OLP patients....20

3.3.7 Effect of stress on the prevalence of depression among OLP patients....21

3.3.8 Effect of publication year on the prevalence of depression among OLP patients....22

3.3.9 Effect of human development index on the prevalence of depression among OLP patients....23

3.3.10 Effect of risk of bias on the prevalence of depression among OLP patients....24

4. Anxiety meta-analyses....25

4.1 Magnitude of association between OLP and anxiety....25

4.2 Prevalence of anxiety among OLP patients. Subgroup meta-analyses....26

4.2.1 Prevalence of anxiety among OLP patients by continent....26

4.2.2 Prevalence of anxiety among OLP patients by diagnostic anxiety test....27

4.2.3 Prevalence of anxiety among OLP patients by specialist implied in diagnosis of anxiety....28

4.2.4 Prevalence of anxiety among OLP patients by publication language....29

4.2.5 Prevalence of anxiety among OLP patients by sex....30

4.3 Prevalence of anxiety among OLP patients. Meta-regression analyses....31

4.3. Effect of sex on the prevalence of anxiety among OLP patients. 31
4.3.2 Effect of age on the prevalence of anxiety among OLP patients 32
4.3.3 Effect of tobacco on the prevalence of anxiety among OLP patients 33
4.3.4 Effect of alcohol on the prevalence of anxiety among OLP patients...........34
4.3.5 Effect of OLP type on the prevalence of anxiety among OLP patients 35
4.3.6 Effect of publication year on the prevalence of anxiety among OLP patients 36
4.3.7 Effect of human development index on the prevalence of anxiety among OLP patients..37
4.3.8 Effect of risk of bias on the prevalence of anxiety among OLP patients......38
5. Stress meta-analyses 39
5.1 Magnitude of association between OLP and stress...39
5.2 Prevalence of stress among OLP patients. Subgroup meta-analyses 40
5.2.1 Prevalence of stress among OLP patients by continent 40
5.2.2 Prevalence of stress among OLP patients by diagnostic stress test 41
5.2.3 Prevalence of stress among OLP patients by specialist implied in diagnosis of stress..42
5.2.4 Prevalence of stress among OLP patients by publication language.............43
5.2.5 Prevalence of stress among OLP patients by sex...44
5.3 Prevalence of stress among OLP patients. Meta-regression analyses.............45
5.3.1 Effect of sex on the prevalence of stress among OLP patients. 45
5.3.2 Effect of age on the prevalence of stress among OLP patients.....................46
5.3.3 Effect of tobacco on the prevalence of stress among OLP patients 47
5.3.4 Effect of alcohol on the prevalence of stress among OLP patients.............48
5.3.5 Effect of OLP type on the prevalence of stress among OLP patients 49
5.3.6 Effect of publication year on the prevalence of stress among OLP patients.50
5.3.7 Effect of human development index on the prevalence of stress among OLP patients..51
5.3.8 Effect of risk of bias on the prevalence of stress among OLP patients.........52
6. Analysis of small-study effects...53
6.1 Prevalence of depression among OLP patients...53
6.2 Prevalence of anxiety among OLP patients 54
6.3 Prevalence of stress among OLP patients 55
7. Sensitivity analysis (leave-one-out method)...56
7.1 Prevalence of depression among OLP patients..56
7.2 Magnitude of association between OLP and depression.................................57
7.3 Prevalence of anxiety among OLP patients 58
7.4 Magnitude of association between OLP and depression.................................59

7.5 Prevalence of stress among OLP patients 60
7.6 Magnitude of association between OLP and stress..61
8. Validation of methodological quality. 62
8.1 List S1. AMSTAR2 checklist 62
8.1 Table S9. AMSTAR2 scoring system..66
9. Depression, Anxiety and Stress Scale (DASS-21 Questionnaire)...............................67
10. List of included studies. List S2 69
11. List of excluded studies with reasons...74
11.1 List S3. Mental disorder not reported 74
11.2 List S4. Overlapping population 184

1. Search strategy

Table S1. Search strategy for each database, number of results, and execution date.

Database	Query/Search Strategy	Items founds /Results	Search time limits
PubMed	"Lichen Planus, Oral"[MeSH] OR "oral lichen planus" [All Fields]	3,578	January-2021
Embase	'oral lichen planus'/exp OR 'oral lichen planus'	3,227	January-2021
Web of Science	TS=("oral lichen planus")	2,931	January-2021
Scopus	TITLE-ABS-KEY ("oral lichen planus")	3,171	January-2021
PyschInfo	"Oral Lichen Planus"	10	January-2021
**TOTAL**			**12,917**

2. Characteristics of analyzed studies

Author	Year	Last author	Correspondin g author	Place, Country	Continent	Source of patients (Pop/Cl)	Recruitment period	OLP sample size (n)	Psychological pathology (n)	Type of study	Study design	Follow-up period		Location of lesion	OLP clinical lesions									Diagnostic criteria								Treatment	
												Yes/No	Months (n)		Red (n)	White (n)	Papular (n)	Reticular (n)	Erosive (n)	Atrophic (n)	Bullous (n)	Plaque (n)	Other (n)	Oral medicine/ specialist	Criteria used for diagnosis of OLP	Differentiation or exclusion between OLP/OLL/LR (Yes/No)	Clinic and histophatologic criteria (Yes/No)	Dysplasia Exclusion (Yes/No)	Tobacco Exclusion (Yes/No)	Evaluation of depression, anxiety and/or stress	Criteria used for depression, anxiety and/or stress	OLP (n)	Depression, anxiety and/or stress (n)
Bagán-Sebastián	1992	Jimenez Y	Bagán-Sebastián	School of Dentistry, Valencia University, Spain	Europe	Cl	ND	205	Stress (66)	Retrospective	Cross-sectional	No	0	Buccal mucosa (184), tongue (103), gingiva (56), palate (16), floor of mouth (6)	148	57	0	0	0	0	0	0	0	Oral medicine	Own criteria	ND	Yes	ND	ND	Self-reported	Anamnesis	ND	ND
Colella	1993	Vito	Colella	Dental and Stomatological Clinic, Faculty of Medicine and Surgery Second University of Naples, Naples, Italy	Europe	Cl	1993	16	Anxiety (16)	Retrospective	Cross-sectional	No	0	ND	ND	ND	ND	ND	ND	ND	ND	ND	ND	ND	ND	ND	Yes	No	ND	Medical assessment	General Health Questionnaire (GHQ) and Hamilton Anxiety Scale (HAM-A)	ND	ND
McCartan	1995	-	McCartan	Department of Oral Surgery, Oral Medicine and Oral Pathology, School of Dental Science, Trinity College, Dublin, Ireland	Europe	Cl	ND	50	Depression (6), anxiety (25)	Prospective	Cross-sectional	No	0	ND	25	28	ND	ND	ND	ND	ND	ND	ND	Oral medicine	WHO 1978	ND	Yes	ND	ND	Medical assessment	Hospital Anxiety and Depression Scale (HADS) and the Cattell 16 PF Questionnaire, Form C.	ND	ND
Pedersen	1996	-	Pedersen	University of Copenhagen, Denmark	Europe	Cl	ND	22	Stress (3)	Retrospective	Cross-sectional	No	0	Buccal mucosa (22), tongue (10), gingiva (6)	-	-	0	22	5	18	3	12	0	Oral medicine	Own criteria	No (LR included)	Yes	ND	No	ND	ND	ND	ND
Cekic-Arambasin	1998	Lazic-Segula	Cekic-Arambasin	General Croatia	Europe	Pop	ND	100	Stress (60)	Retrospective	Cross-sectional	No	0	ND	-	-	0	62	33	0	0	0	5	Oral medicine	WHO 1978	ND	ND	ND	ND	Medical assessment	ND	ND	ND
Mignona	1998	Bucci	Mignona	Departments of Oral Medicine and Pathology at the University of Naples "Federico II" and the University of Bari, Italy	Europe	Cl	1994-1998	263	Stress (96)	Prospective	Cross-sectional	No	0	Buccal mucosa (242), tongue (63), gingiva (16), palate (10), floor of the mouth (6), lip (6)	-	-	0	54	72	14	1	33	Mixed (89*)	Oral medicine/pathologist	Own criteria	ND	Yes	ND	ND	Medical assessment and Self-reported	Anamnesis	ND	ND
Eisen	2002	-	Eisen	A dermatologic clinic, Cincinnati, Ohio, USA	North America	Cl	ND	723	Stress (123)	Retrospective	Longitudinal	Yes	6-96	Buccal mucosa (776), tongue (435), gingiva (401), lip (117), floor of mouth (77), palate (43)	-	-	0	267	290	0	0	0	Erytematous (166)	Oral pathologist/ dermatopathologist	Own criteria	Yes (OLL excluded)	Yes	Yes	No	Self-reported	ND	ND	ND
Soto Araya	2004	Esguep	Soto Araya	The Diagnosis Centre of the Faculty of Odontolgy of the University of Chile and the Maxillofacial Department of the San Juan de Dios, Barros Luco and Sótero del Rio Hospitals, Chile	South America	Cl	ND	9	Depression (4), anxiety (7), stress (6)	Prospective	Cross-sectional	No	0	ND	-	-	0	4	3	2	0	0	0	Oral medicine/pathologist	Own criteria	Yes (OLL excluded)	No	No	ND	Medical assessment	Depression and anxiety: Hamilton Anxiety and Depression Scale<br>Stress: Test of Recent Expectience (TRS)	ND	No
Lundqvist	2006	Bergdahl	Lundqvist	Vulva Clinic and the Department of Oral Medicine, Umeå University Hospital, Sweden	Europe	Cl	ND	49	Depression (19), anxiety (17), stress (17)	Prospective	Cross-sectional	No	0	ND	-	-	0	0	49	0	0	0	0	Oral medicine	Own criteria	ND	Yes	ND	ND	Medical assessment	Depression: Beck Depression Inventory (BDI) questionnaire<br>Anxiety: The scale from the State-Trait Anxiety Inventory (STAI-S)<br>Stress: The General Perceived Stress Questionnaire (PSQ)	ND	Antidepressants (1)
Sun	2007	Chiang	Chiang	National Taiwan University, Taipei, Taiwan	Asia	Cl	ND	158	Stress (60)	Prospective	Cross-sectional	No	0	ND	142	16	ND	ND	ND	ND	ND	ND	ND	ND	ND	ND	ND	ND	ND	Self-reported	ND	ND	ND
Lundström	2009	-	Lundström	Department of Oral Medicine at the University Hospital in Linköping, Sweden	Europe	Cl	ND	803	Depression (37)	Prospective	Cross-sectional	No	0	ND	ND	ND	ND	ND	ND	ND	ND	ND	ND	Oral medicine	WHO 1980	No (OLL included)	Yes	Yes	No	Self-reported	Anamnesis	ND	ND
Shah	2009	Sujatha	Shah	An outpatient deparment, India	Asia	Cl	ND	30	Depression (21), stress (30)	Prospective	Cross-sectional	No	0	ND	-	-	0	11	10	0	0	0	Linear (1), Annular (1), Combined (7)	ND	ND	ND	Yes	ND	Yes	Medical assessment	Depression Anxiety Stress Scale-21 (DASS-21)	ND	ND
Bermejo-Fenoll	2010	Salazar-Sánchez	Sánchez-Siles	Department of Oral Medicine of the University of Murcia, Spain; Private practice setting of one of the authors (ABF) in Elche, Alicante, Spain	Europe	Cl	1991-2007	550	Depression (97), anxiety (97)	Retrospective	Cross-sectional	No	0	Buccal mucosa (428), gingiva (428), tongue (299), lip (81), palate (74), floor of mouth (33)	359	191	ND	ND	ND	ND	ND	ND	ND	Oral medicine	WHO 1978	Yes (LR excluded)	Yes	Yes	No	Medical assessment	ND	ND	Antidepressants (8)
Wu X	2011	Xia	Wu X	Dental Clinic, Department of Stomatology, The Sixth Affiliated Hospital of Wenzhou Medical College, Lishui People's Hospital, Lishui, Zhejiang province, China	Asia	Cl	2008-2009	67	Depression (16)	Prospective	Cross-sectional	No	0	ND	25	42	ND	ND	ND	ND	ND	ND	ND	Oral medicine	Oral Mucosal Disease Professional Committee of the Medical Association Trial standards	ND	Yes	ND	ND	Medical assessment	Jiao Self-Rating Scale, self-evaluation of depression (Self-rating Depression Scale, SDS) and Hamilton Depression Scale for depression (HAM-D)	ND	ND
Jayavelu	2012	Sambandan	Jayavelu	Department of Oral Medicine and Radiology, Rama Dental College and Research Centre, Kanpur, India	Asia	Cl	ND	30	Stress (4)	Prospective	Cross-sectional	No	0	Buccal mucosa (29), gingiva (6), tongue (3), lip (3), palate (1), oropharynx (1)	7	23	ND	ND	ND	ND	ND	ND	ND	Oral medicine	Own criteria	Yes (LR excluded)	Yes	ND	No	Self-reported	ND	ND	ND
Park	2012	Woo	Park	Division of Oral Medicine and Dentistry, Brigham and Women's Hospital, Boston, USA	North America	Cl	2005-2011	115	Depression (17)	Retrospective	Longitudinal	Yes	0.5-11.27 (only 55 patients)	Buccal mucosa (109), gingiva (74), tongue (45), palate (21), floor of mouth (2)	92	23	ND	ND	ND	ND	ND	ND	ND	Oral medicine /pathologist	WHO-modified 2003	No	Yes	Yes	ND	Medical assessment	ND	Topical and/or systemic steroids: fluocinonide 0.05% gel, dexamethasone solution 0.1 mg/1 mL, clobetasol 0.05% gel, tacrolimus 0.1% ointment and prednisone 0.75 to 1 mg/kg<br>Iintralesional injection: triamcinolone 40 mg/mL for ulcers at a dose of 10 mg/cm2 of ulceration	ND
Bokor-Bratic	2013	Dragnic	Cankovic	Oral Medicine Section of the Dental Clinic, Faculty of Medicine, Novi Sad, Serbia	Europe	Cl	2007-2009	90	Anxiety (15)	Prospective	Cross-sectional	No	0	ND	ND	ND	ND	ND	ND	ND	ND	ND	ND	Oral medicine /pathologist	WHO-modified 2003	ND	Yes	Yes	No	Self-reported	Questionnaire	No	ND
Gümrü	2013	-	Gümrü	Oral Diagnosis and Radiology Department of Marmara University Faculty of Dentistry, Istanbul, Turkey	Europe, Asia	Cl	1990-2010	370	Depression (36), anxiety (36), stress (75)	Retrospective	Cross-sectional	No	0	Buccal mucosa (356), gingiva (116), tongue (102), palate (34), floor of mouth (11)	224	146	ND	ND	ND	ND	ND	ND	ND	Oral medicine/ pathologist	WHO-modified 2003	Yes (OLL and LR excluded)	Yes	Yes	No	Medical assessment	ND	Topical, systemic corticosteroids alone or combinated	Antidepressant/anxiolytic (31)
Hirota	2013	Migliari	Migliari	Clinic of Oral Medicine, University of São Paulo, Brazil	South America	Cl	2006-2010	91	Depression (50), anxiety (91)	Prospective	Cross-sectional	No	0	ND	ND	ND	ND	ND	ND	ND	ND	ND	ND	Oral medicine/ pathologist	WHO-modified 2003	Yes (LR excluded)	Yes	Yes	ND	Self-reported	Depression: Center for Epidemiologic Studies Depression Scale (CES-D)<br>Anxiety: Anxiety Scale (STAI-T)	ND	ND
Kumar	2013	Kale	Kumar	Outpatient block of departments of Oral Medicine and Radiology, KLEVK Institute of Dental Sciences, Belgaum and SDM College of Dental Sciences, Dharwad, India	Asia	Cl	ND	45	Stress (15)	Prospective	Cross-sectional	No	0	ND	-	-	1	29	2	4	0	7	Ulcerative (2)	ND	Own criteria	ND	Yes	ND	No	Self-reported	Anamnesis	ND	ND

Tovaru	2013	Sardella	Tovaru	Department of Oral Medicine and Oral Pathology, Faculty of Dental Medicine, Carol Davila University, Bucharest, Romania	Europe	Cl	1990-2010	633	Stress (128)	Retrospective	Cross-sectional	No	0	Buccal mucosa > tongue > gingiva > floor of mouth	323	310	ND	ND	ND	ND	ND	ND	ND	Oral medicine /pathologist	WHO-modified 2003	Yes (OLL excluded)	Yes	Yes	No	ND	ND	ND	ND
Adamo	2014	Mignona	Mignona	Oral Medicine Unit of the 'Federico II University of Naples', Italy	Europe	Cl	2011	50	Depression (18), anxiety (21)	Prospective	Cross-sectional	No	0	ND	0	50	ND	ND	ND	ND	ND	ND	ND	Oral medicine/ pathologist	Own criteria	ND	Yes	Yes	ND	Medical assessment	Depression: Hamilton Rating Scale for Depression (HAM-D)<br>Anxiety: Hamilton Rating Scale for Anxiety (HAM-A)	No	No
Gavic	2014	Gruden Pokupec	Gavic	Dental clinic at school of Medicine of University of Split, Croatia	Europe	Cl	2011-2012	112	Depression (79), anxiety (87), stress (90)	Prospective	Cross-sectional	No	0	ND	ND	ND	ND	ND	ND	ND	ND	ND	ND	Dentist	Own criteria	ND	Yes	ND	No	Medical assessment	Depression: Beck Depression Inventory (BDI)<br>Anxiety: The State-Trait Anxiety Inven- tory (STAI)<br>Stress: Ways of Coping Questionnaire (WCQ)	ND	ND
Radochova	2014	Slezák	Radochova	Oral Medicine Unit at Department of Dentistry Clinic Charles University in Prague, Faculty of Medicine and University Hospital in Hradec Králové, the Czech Republic	Europe	Cl	2003-2013	171	Depression (16), anxiety (16)	Retrospective	Cross-sectional	No	0	Buccal mucosa (197), tongue (93), gingiva (44), lip (32), palate (8), floor of mouth (6)	92	79	ND	ND	ND	ND	ND	ND	ND	Oral medicine /pathologist	WHO-modified 2003	Yes (OLL, LR excluded)	Yes	Yes	No	Medical assessment	ND	Topical steroids alone or combinated with systemic steroids, combination of dexamtehasone gel and depot form of corticosteroid intralesional	ND
Sandhu S	2014	Sandhu J	Sandhu S	Department of Oral and Maxillofacial Pathology, Genesis Institute of Dental Sciences and Research, Ferozepur Moga Road, Punjab, India	Europe	Cl	2009-2001	49	Depression (5), anxiety (31), stress (31)	Prospective	Cross-sectional	No	0	ND	ND	ND	ND	ND	ND	ND	ND	ND	ND	Maxillofacial and pathologist	ND	Yes (LR excluded)	Yes	Yes	ND	Medical assessment	Hospital Anxiety and Depression Scale (HADS)	ND	ND
Alves	2015	Almeida	Alves	Sao Jose, dos Campos Dental School, UNESP – University Estadual Paulista, Sao Jose dos Campos, Sao Paulo, Brazil	South America	Cl	2009-2011	48	Depression (28), anxiety (48)	Prospective	Cross-sectional	No	0	ND	-	-	0	0	48	0	0	0	0	Oral medicine/ pathologist	WHO-modified 2003, Eisemberg 2000	No	Yes	ND	ND	Self-reported	Depression: The Self Reporting Questionnaire-20 (SRQ-20) and the Medical Outcomes Study 36-Item Short Form Health Survey (SF-36)<br>Anxiety: State-Trait Anxiety Inventory (STAI-s and STAI-t)	ND	ND
Barbosa	2015	de Medeiros	de Medeiros	Stomatology Clinic of the Federal University of Rio Grande do Norte and Federal University of Paraib, Brazil	South America	Cl	2000-2010	37	Anxiety (37)	Prospective	Cross-sectional	No	0	Buccal mucosa (29), gingiva (16), tongue (10), lip (7), palate (5)	16	21	ND	ND	ND	ND	ND	ND	ND	Oral medicine/ pathologist	Own criteria	ND	Yes	ND	ND	Medical assessment	The State-Trait Anxiety Inventory (STAI, part II)	ND	ND
Kalkur	2015	Sattur	Kalkur	ND	Asia	Cl	ND	25	Depression (3), anxiety (3), stress (6)	Prospective	Cross-sectional	No	24	ND	ND	ND	ND	ND	ND	ND	ND	ND	ND	Oral medicine/ pathologist	Own criteria	Yes (LR excluded)	Yes	Yes	No	Medical assessment	Depression Anxiety Stress Scale (DASS-42)	No	ND
Mostafa	2015	Ahmed	Mostafa	Outpatient clinic at the Faculty of Oral and Dental Medicine, Cairo University, Egypt	Asia	Pop	2012-2014	64	Stress (6)	Retrospective	Longitudinal	Yes	24	Buccal mucosa (52), gingiva (37), lip (28), tongue (11), palate (2)	-	-	2	6	13	38	3	2	0	Oral medicine/ pathologist	WHO-modified 2003	Yes (OLL, LR excluded)	Yes (not all patients)	ND	No	Self-reported	ND	ND[a]	ND
Choi YS	2016	Choi Y	Yoon, Park H, Choi Y	Oral Medicine Clinic, Seoul National University Dental Hospital (SNUDH); Tissue bank at the Department of Oral Pathology, SNUDH, South Korea	Asia	Cl	ND	13	Depression (1)	Prospective	Cross-sectional	No	0	Buccal mucosa (13), gingiva (5), tongue (3), palate (3), lip (1)	ND	ND	ND	ND	ND	ND	ND	ND	ND	Oral medicine/ pathologist	Own criteria	Yes (differentiation between OLP/OLL)	Yes	ND	Yes	Self-reported	ND	No	ND
Lauritano	2016	Petruzzi	Petruzzi	Dental Clinic of University of Bari, Italy	Europe	Cl	2011-2014	87	Depression (9), anxiety (9)	Retrospective	Cross-sectional	No	0	Buccal mucosa (56), tongue (31), gingiva (20), palate (10), fornix (6), lip (6),	34	53	ND	ND	ND	ND	ND	ND	ND	ND	WHO-modified 2003	ND	Yes	Yes	No	Self-reported	Anamnesis	Topical corticosteroids: clobetasol propionate ointment in orabase.<br>Systemic corticosteroids and topical calcineurin inhibitors were used alone or in combination.	ND
Mankapure	2016	Bhavthankar	Mankapure	Department of Oral Pathology and Microbiology, Government Dental College and Hospital, Aurangabad, India	Asia	Pop	ND	108	Stress (35)	Retrospective	Cross-sectional	No	0	Buccal mucosa (111), gingiva (32), tongue (18), fornix (2)	-	-	0	87	21	0	0	0	0	ND	WHO-modified 2003	Yes (OLL, LR excluded)	Yes	Yes	No	Self-reported	ND	Topical steroids (as a mucosal adhesive paste or as intralesional injection) alone or in combination with systemic steroids	ND
Pippi	2016	Petti	Petti	Department of Oral and Maxillofacial Sciences of the 'Sapienza' University of Rome, Rome, Italy	Europe	Cl	2014-2015	67	Anxiety (21)	Prospective	No	0	No	ND	24	43	ND	ND	ND	ND	ND	ND	ND	Oral medicine/ pathologist	WHO 1978	No	Yes	ND	No	Self-reported	Psychological General Well-Being Index (PGWBI- S)	Excluded	ND
Gupta	2017	Kamarthi	Prakash S. Mohan	Department of Oral Medicine and Radiology, Subharti Dental College, Meerut, Uttar Pradesh, India	Asia	Cl	ND	39	Depression (37), anxiety (35), stress (36)	Prospective	Longitudinal	Yes	12	Buccal mucosa (34), buccal vestibule (39), floor of the mouth (13), tongue (8), gingiva (4), palate (1)	-	-	10	31	17	7	1	3	0	Oral medicine/ pathologist	Own criteria	No	No	ND	Yes	Medical assessment	Depression Anxiety Stress Scale-21 (DASS-21)	No	ND
Siponen	2017	Salo	Siponen	Department of Diagnostics and Oral Medicine, Institute of Dentistry, University of Oulu or Oulu University Hospital and at the Department of Oral and Maxillofacial Diseases at Kuopio University Hospital, Finland	Europe	Cl	2004-2014	27	Depression (4)	Prospective	Longitudinal, trial	Yes	6	Buccal mucosa (27), gingival (26), tongue (12), floor of mouth (7), palate (5), lip (4)	26	1	ND	ND	ND	ND	ND	ND	ND	Oral medicine/ pathologist	Pindborg et al. 1997	Yes (OLL, LR excluded)	Yes (25 patients)	ND	No	Medical assessment	ND	ND	ND
Adamo	2018	Fortuna	Adamo	Oral Medicine Unit of the "Federico II University of Naples", Italy	Europe	Cl	2013	28	Anxiety (28)	Prospective	Longitudinal	Yes	6	Buccal mucosa (23), gingival (12), tongue (2), palate (1)	-	-	0	28	0	0	0	0	0	Oral medicine/ pathologist	WHO-modified 2003	ND	Yes	Yes	ND	Medical assessment	Hamilton Anxiety Scale (HAM-A)	ND[a]	ND
Di Stasio	2018	Lucchese	Serpico	Multidisciplinary Department of Medical-Surgical and Dental Specialties, University of Campania Luigi Vanvitelli, Naples, Italy	Europe	Cl	ND	11	Depression (1), anxiety (11)	Prospective	Cross-sectional	No	0	ND	ND	ND	ND	ND	ND	ND	ND	ND	ND	Dentist	ND	ND	ND	ND	ND	Medical assessment	Depression: Hamilton Rating Scale for Depression (HAM-D)<br>Anxiety: State-Trait Anxiety Inventory (STAI 1-2)	ND	ND
Edens	2018	Brennan	Brennan	Outpatient Oral Medicine Department, Carolinas Medical Center, Charlotte, NC, USA	North America	Cl	ND	45	Depression (10), stress (10)	Retrospective	Longitudinal	Yes	1.25	ND	-	-	0	30	23	0	0	34	0	Oral medicine	Own criteria	Yes (LR excluded)	Yes (not all patients)	ND	No	Medical assessment	Anamnesis	Topical steroids: clobetasol, dexamethasone, fluocinonide, triamcinolone<br>Systemic corticosterois	ND
Yang	2018	Zhang	Zhang	Department of Oral Medicine, Stomatology Hospital of Henan province, China	Asia	Cl	2017	45	Depression (17), anxiety (19)	Prospective	Cross-sectional	No	0	ND	ND	ND	ND	ND	ND	ND	ND	ND	ND	Oral medicine/ pathologist	Own criteria	No	No	ND	ND	Medical assessment	Hospital Anxiety and Depression Scale (HADS)	ND	ND
Manczyk	2019	Oruba	Oruba	Outpatient Clinic of Periodontology and Clinical Oral Pathology, University Dental Clinic in Kraków, Poland	Europe	Cl	ND	26	Depression (19), anxiety (17), stress (5)	Prospective	Cross-sectional	No	0	ND	ND	ND	ND	ND	ND	ND	ND	ND	ND	Dentist	ND	No	Yes	No	No	Medical assessment	Depression Anxiety Stress Scale-21 (DASS-21)	ND	ND
Vilar-Villanueva	2019	Blanco-Carrión	Gándara-Vila	Clinic of the Oral Medicine Teaching Unit of the Faculty of Odontology of the University of Santiago de Compostela, Spain	Europe	Cl	2017	48	Depression (31), anxiety (17)	Prospective	Cross-sectional	No	0	Buccal mucosa > Tongue = Gingiva	25	23	ND	ND	ND	ND	ND	ND	ND	Oral medicine/ pathologist	WHO-modified 2003	No	Yes	ND	No	Medical assessment	Hospital Anxiety and Depression Scale (HADS) and Oral Health Impact Profile<br>(OHIP-14)	ND	ND
Wang	2019	Fan	Fan	Department of Oral Medicine, Affiliated Hospital of Stomatology, Nanjing Medical University, Nanjing, China	Asia	Cl	ND	100	Depression (25), anxiety (7)	Prospective	Cross-sectional	No	0	ND	-	-	0	75	25	0	0	0	0	Oral medicine/ pathologist	WHO 1978	ND	Yes	ND	ND	Medical assessment	Zung self-rating anxiety scale and self-rating depression scale (SAS and SDS)	No	ND
Birckel	2020	Cribier	Birckel	Dermatology service, Strasbourg University Hospital, France	Europe	Cl	ND	9	Depression (1)	Prospective	Longitudinal	Yes	5.5	ND	-	-	0	0	9	0	0	0	0	Dermatologist	ND	ND	Yes	ND	ND	Medical assessment	ND	ND	ND
Chaitanya	2020	Bontala	Chaitanya	Department of Oral Medicine and Radiology, Panineeya Mahavidyala Institute of Dental Sciences and Research Centre, Hyderabad, India	Asia	Cl	ND	30	Depression (21), anxiety (25)	Prospective	Cross-sectional	No	0	ND	ND	ND	ND	ND	ND	ND	ND	ND	ND	Oral medicine/ pathologist	ND	Yes (LR excluded)	Yes	ND	ND	Medical assessment	Hospital Anxiety and Depression Scale (HADS)	ND	ND
Pires	2020	Freitas	Pires	Reference Center for Oral Lesions in the Department of Health Sciences, State University of Feira de Santana, Brazil	South America	Cl	2016-2017	21	Stress (15)	Prospective	Cross-sectional	No	0	Buccal mucosa (11) > other sites	3	18	ND	ND	ND	ND	ND	ND	ND	ND	WHO 2003	ND	Yes	ND	Yes	Medical assessment	Perceived Stress Scale	No	ND
Shaw	2020	Bansal	Konidena	Department of Oral Medicine and Radiology of a dental college in Haryana, India	Asia	Cl	ND	43	Depression (36), anxiety (38), stress (22)	Prospective	Cross-sectional	No	0	ND	-	-	0	31	4	0	0	5	Ulcerative (1), Annular (1), Papular (1)	Oral medicine/ pathologist	WHO 2003	Yes (LR excluded)	Yes	Yes	Yes	Medical assessment	Depression Anxiety Stress Scale (DASS-42)	ND	ND
Vehviläinen	2020	Siponen	Siponen	Kuopio University Hospital, Department of Clinical Pathology, Kuopio, Finland	Europe	Cl	ND	28	Depression (1)	Retrospective	Cross-sectional	No	0	Buccal mucosa (22), tongue (5), gingiva (1)	ND	ND	ND	ND	ND	ND	ND	ND	ND	ND	the American academy of oral and maxillofacial pathology (AAOMP)	ND	Yes	ND	ND	Medical assessment	ND	ND	Escitalopram

Wiriyakijja	2020	Riordain	Wiriyakijja	Oral Medicine clinic, UCLH Eastman Dental Hospital, London, UK	Europe	Cl	2018-2019	281	Depression (53), anxiety (104), stress (164)	Prospective	Cross-sectional	Yes	0	ND	229	52	ND	ND	ND	ND	ND	ND	ND	Oral medicine	WHO 2003	Yes (OLL excluded)	Yes	Yes	Yes	Medical assessment	Depression and Anxiety: Hospital Anxiety and Depression Scale (HADS)<br>Stress: the 10-item Perceived Stress Scale (PSS- 10)	ND	ND
Zhong	2020	Sedghizadeh	Sedghizadeh	Herman Ostrow School of Dentistry, Oral Medicine Clinic, in Los Angeles, California, United States	North America	Cl	ND	10	Depression (1), anxiety (1)	Retrospective	Cross-sectional	No	0	ND	-	-	0	9	5	0	0	0	0	Oral medicine/ pathologist	WHO 2003	ND	Yes	ND	ND	ND	ND	Dexamethasone oral elixir 0.5 mg/mL rinse and spit tid (6 patients), Clobetasol gel 0.05% for topical use tid (1 patient), Fluocinonide gel 0.05% for topical use tid (1 patient), combined treatment (1 patient)	ND
Adamo	2021	Mignona	Calabria	Fifteen Italian Oral Medicine outpatients' departments of different Italian Universities (eight northern, three central and four southern universities) join with the Italian Society of Oral Pathology and Medicine (SIPMO- Società Italiana di Patologia e Medicina Orale).	Europe	Cl	2018-2020	600	Depression (291), anxiety (307)	Prospective	Cross-sectional	No	0	Palate (273), tongue (243), lips (160), buccal mucosa (138), gingiva (123), floor of the mouth (114)	300	300	ND	ND	ND	ND	ND	ND	ND	Oral medicine/ pathologist	WHO 2003	Yes	Yes	Yes	No	Medical assessment	Depression: Hamilton Rating Scale for Depression (HAM-D)<br>Anxiety: Hamilton Rating Scale for Anxiety (HAM-A)	No	ND
Liao	2021	Jiang	Jiang	National Clinical Research Center for Oral Diseases, Department of Oral Medicine, West China Hospital of Stomatology, Sichuan University, Chengdu, Sichuan, China	Asia	Cl	ND	174	Anxiety (22)	Prospective	Cross-sectional	No	0	ND	76	98	ND	ND	ND	ND	ND	ND	ND	Oral medicine/ pathologist	WHO 1978	ND	Yes	ND	ND	Self-reported	Self-rating Anxiety Scale (SAS)	ND	ND

Abbreviations: Cl, clinic; ND, not described; OLP, oral lichen planus; OLL, oral lichenoid lesions; OLR, oral lichenoid reactions; Pop, Population; WHO, World Health Organisation.

3. Depression meta-analyses.

3.1 Magnitude of association between OLP and depression.

Figure S1. Forest plot graphically representing the analysis of the magnitude of association -using OR as effect size measure- between depression and OLP.

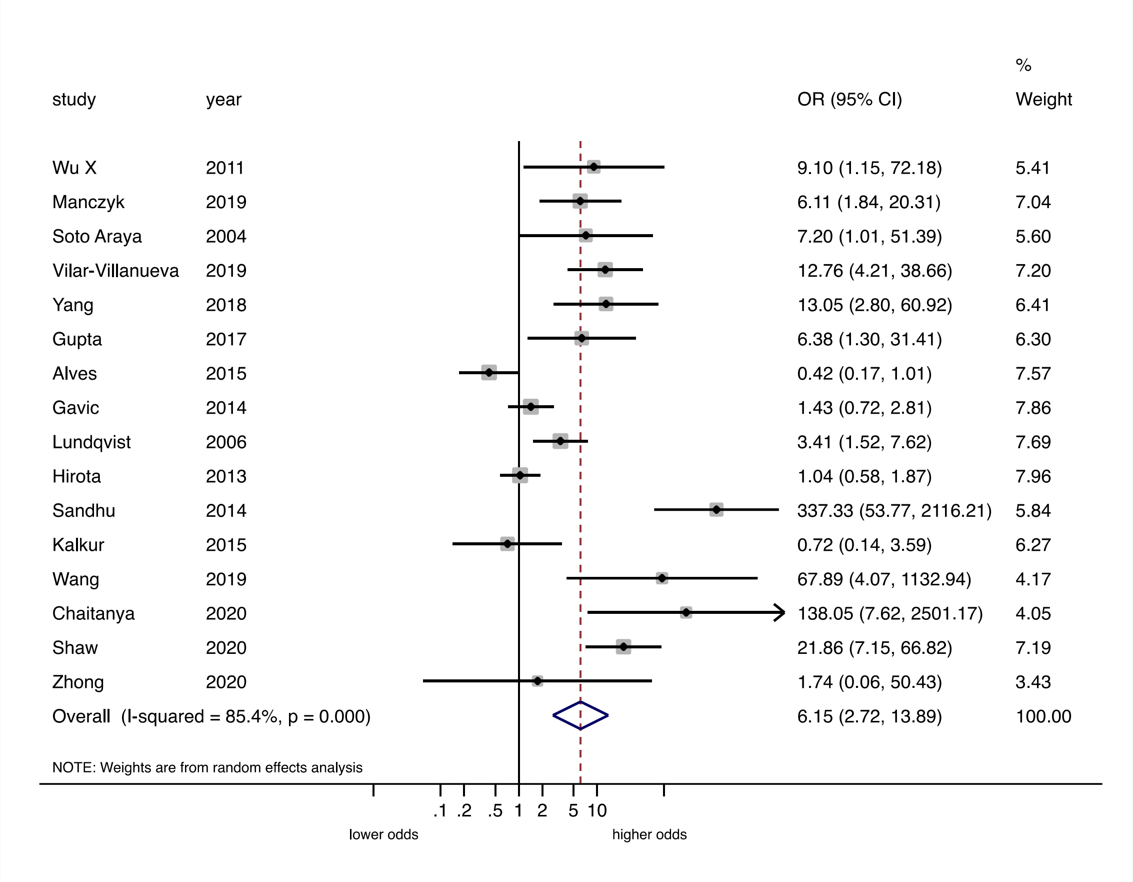

OR, odds ratio; CI, confidence interval; Random-effects model.

3.2 Prevalence of depression among OLP patients. Subgroup meta-analyses.

3.2.1 Prevalence of depression among OLP patients by continets.

Figure S2. Forest plot graphically representing the stratified analysis of the prevalence of depression -using pooled proportions as effect size measure- among OLP patients by continents.

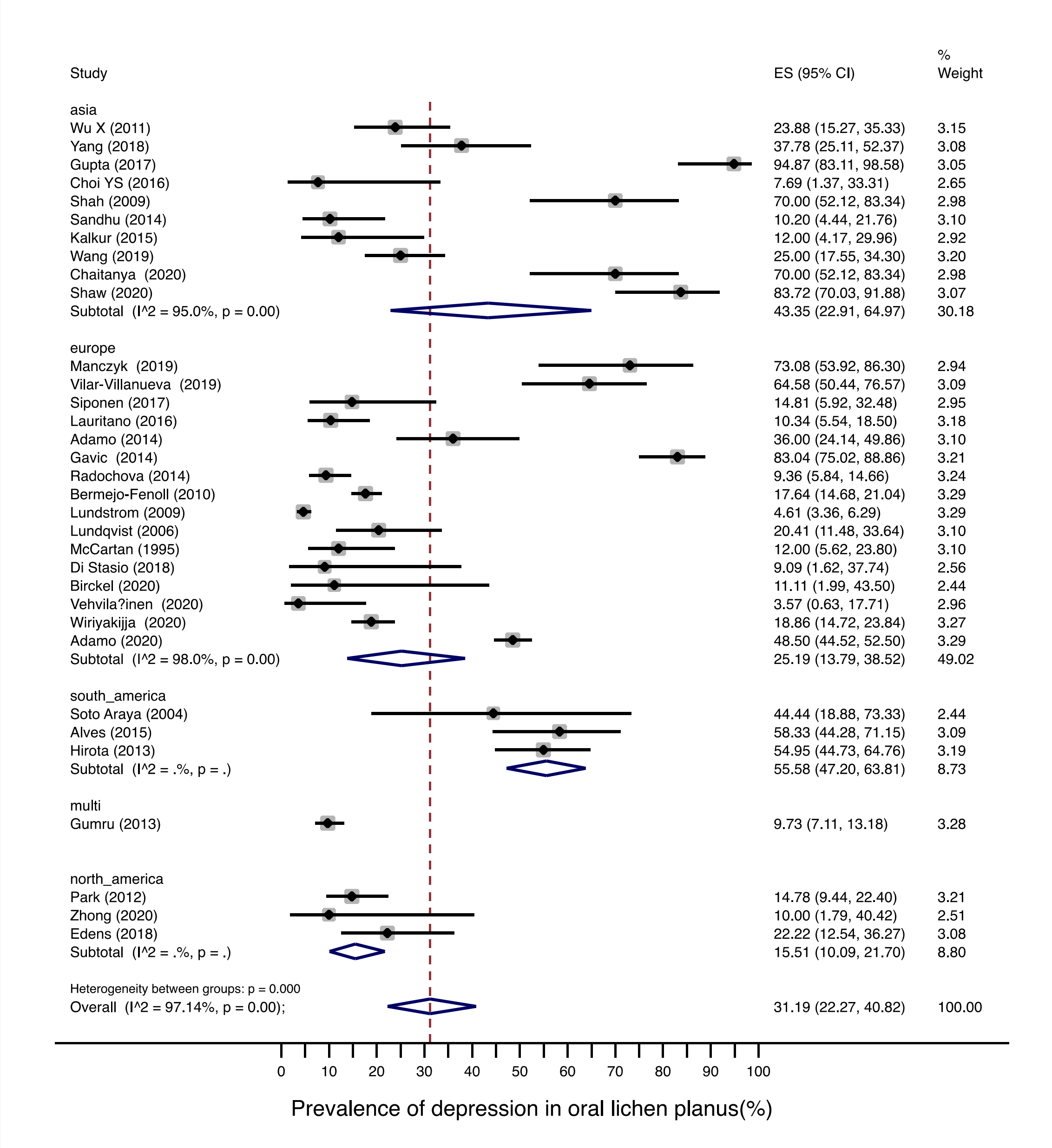

ES, effect size; CI, confidence interval; Random-effects model.

3.2.2 Prevalence of depression among OLP patients by dignostic depression test.

Figure S3. Forest plot graphically representing the stratified analysis of the prevalence of depression -using pooled proportions as effect size measure - among OLP patients by diagnostic depression test.

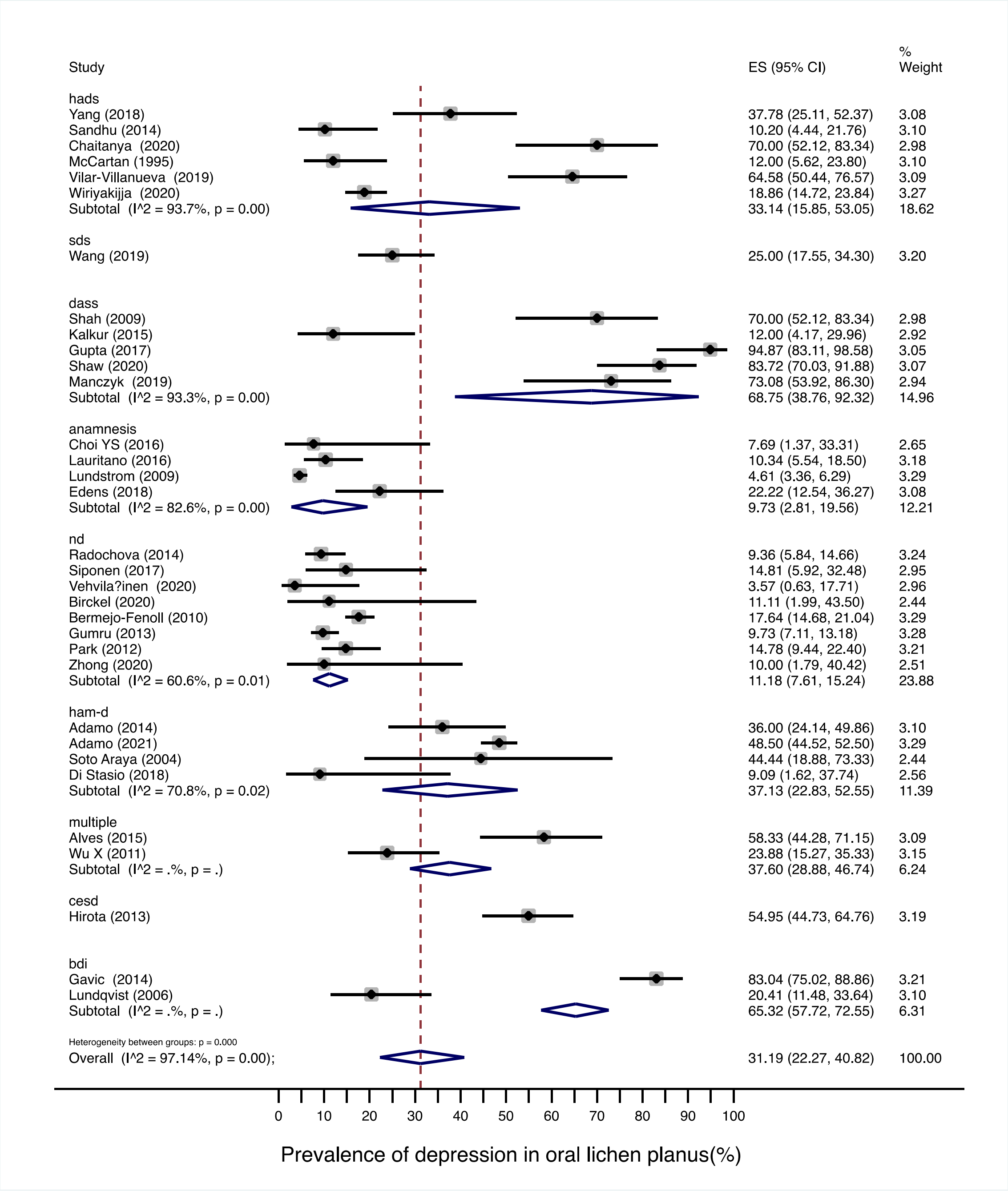

ES, estimation; CI, confidence interval; Random-effects model.

3.2.3 Prevalence of depression among OLP patients by specialist implied in diagnosis of depression.

Figure S4. Forest plot graphically representing the stratified analysis of the prevalence of depression -using pooled proportions as effect size measure - among OLP patients by specialist implied in diagnosis of depression.

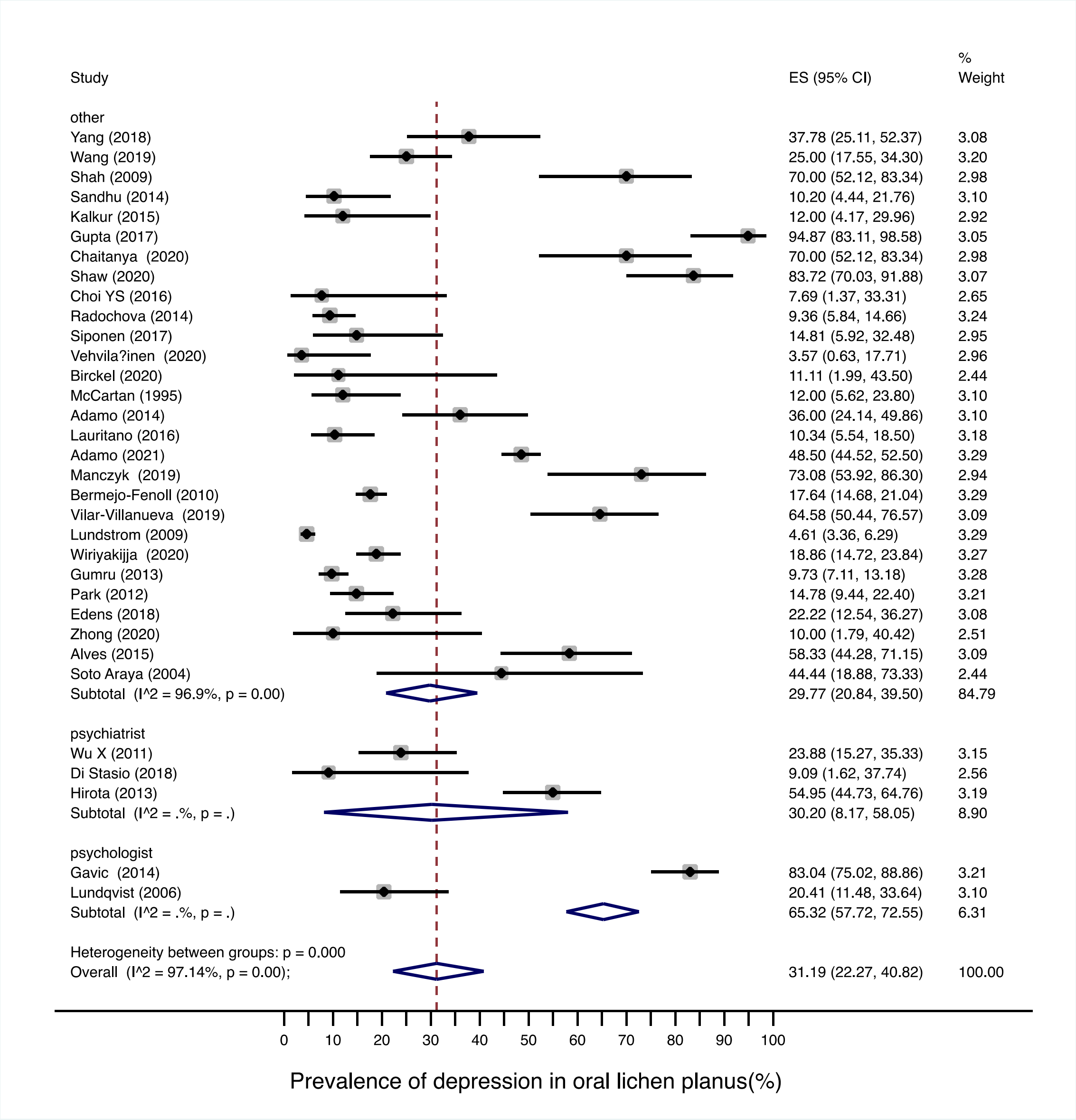

ES, estimation; CI, confidence interval; Random-effects model.

3.2.4 Prevalence of depression among OLP patients by publication language.

Figure S5. Forest plot graphically representing the stratified analysis of the prevalence of depression -using pooled proportions as effect size measure- among OLP patients by publication language.

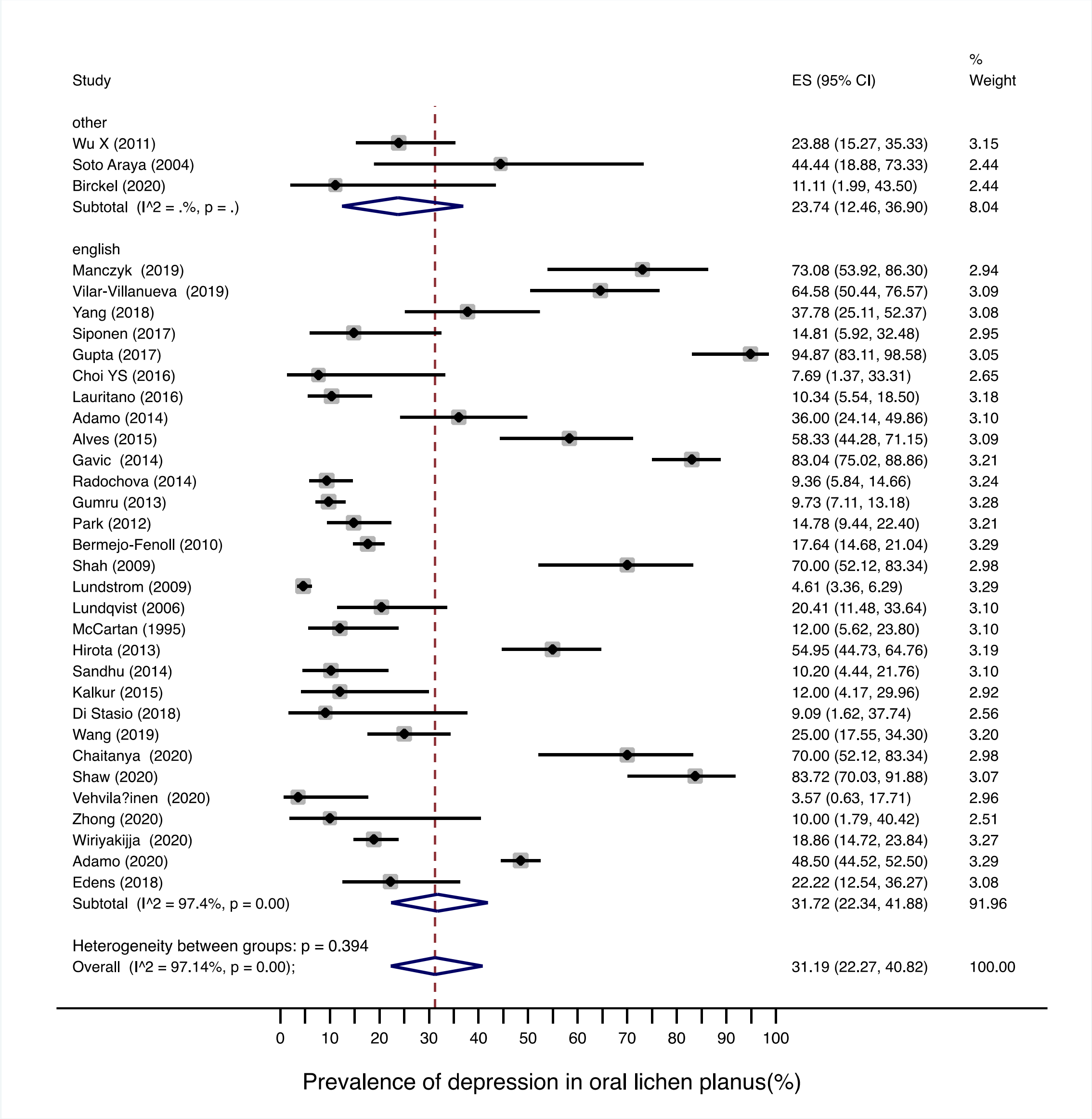

ES, estimation; CI, confidence interval; Random-effects model.

3.2.5 Prevalence of depression among OLP patients by sex.

Figure S6. Forest plot graphically representing the stratified analysis of the prevalence of depression -using pooled proportions as effect size measure- among OLP patients by sex.

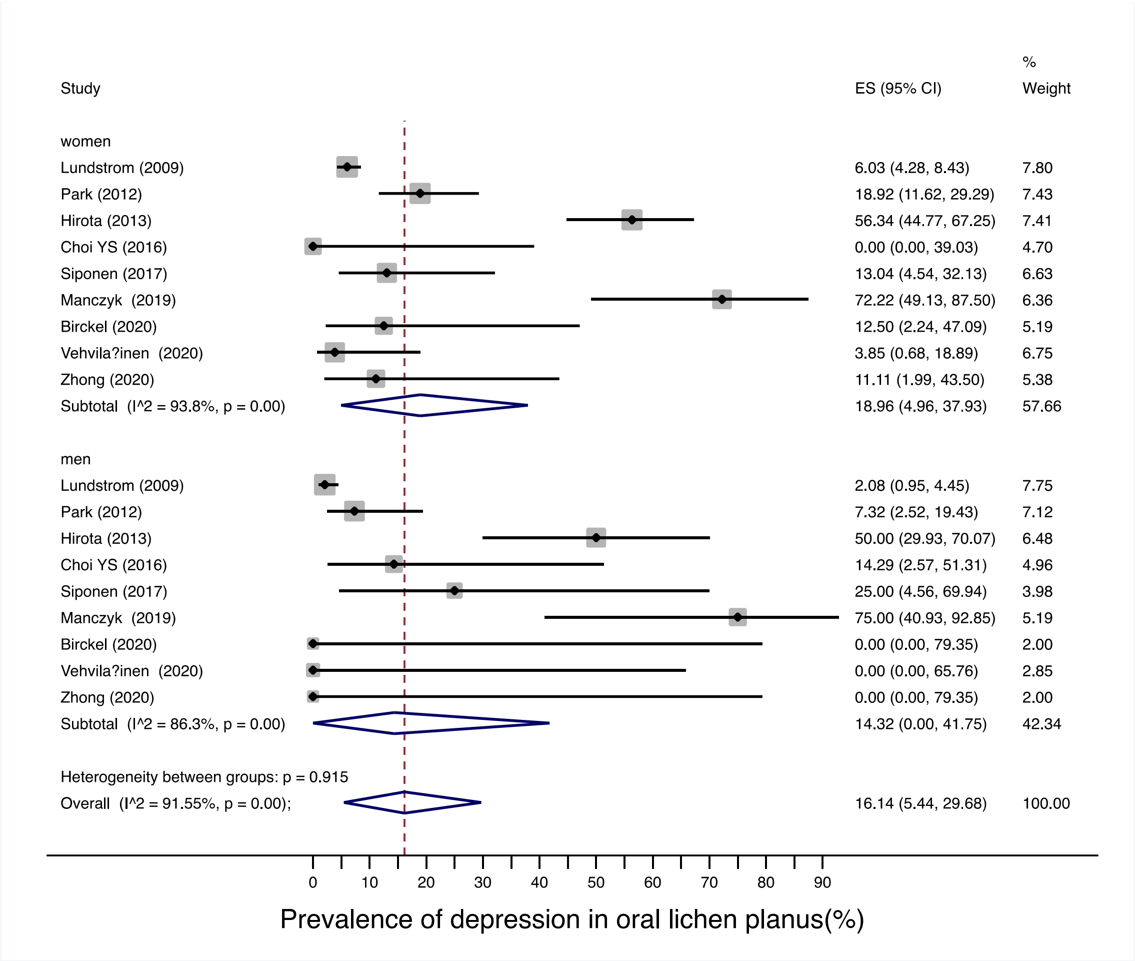

ES, estimation; CI, confidence interval; Random-effects model.

3.3 Prevalence of depression among OLP patients. Meta-regression analyses.

3.2.1 Effect of sex on the prevalence of depression among OLP patients.

Figure S7. Bubble plot graphically representing the potential effect of sex (% of female patients) on depression prevalence (derived from meta-regression analysis; see table 2).

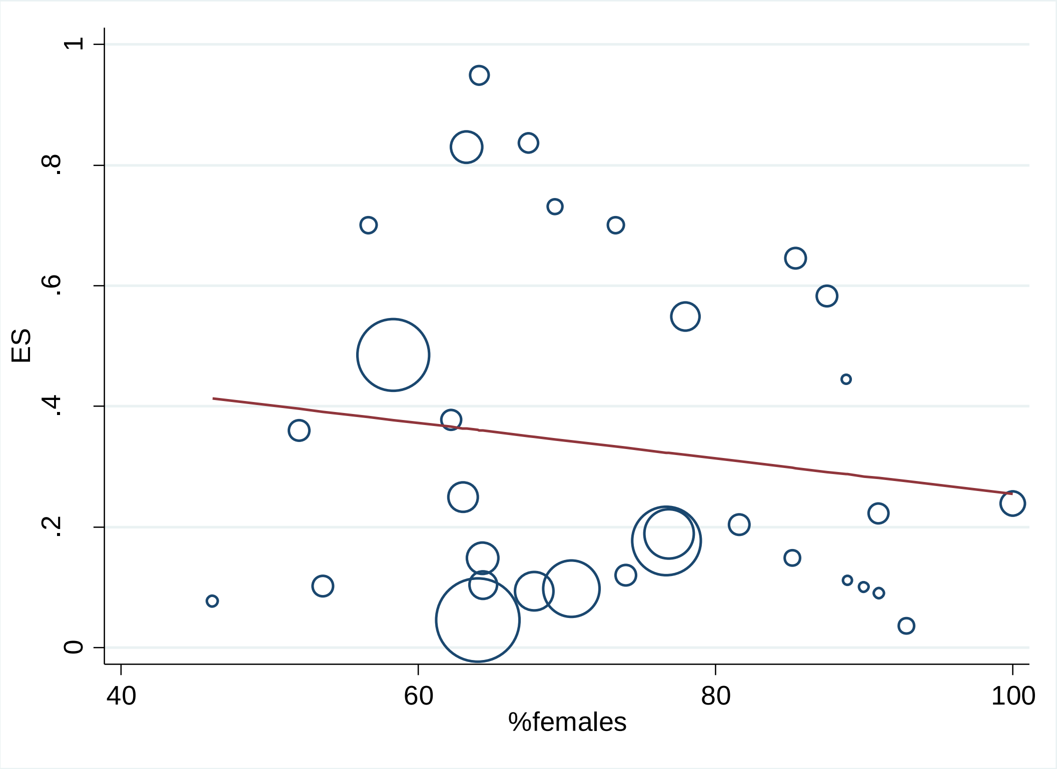

The red line exhibits the fitted regression line together with blue circles representing the estimates from each individual study, sized according to the precision of each estimate (the inverse of its within-study variance).

3.2.2 Effect of age on the prevalence of depression among OLP patients.

Figure S8. Bubble plot graphically representing the potential effect of age (expressed in years) of patients on depression prevalence (derived from meta-regression analysis; see table 2).

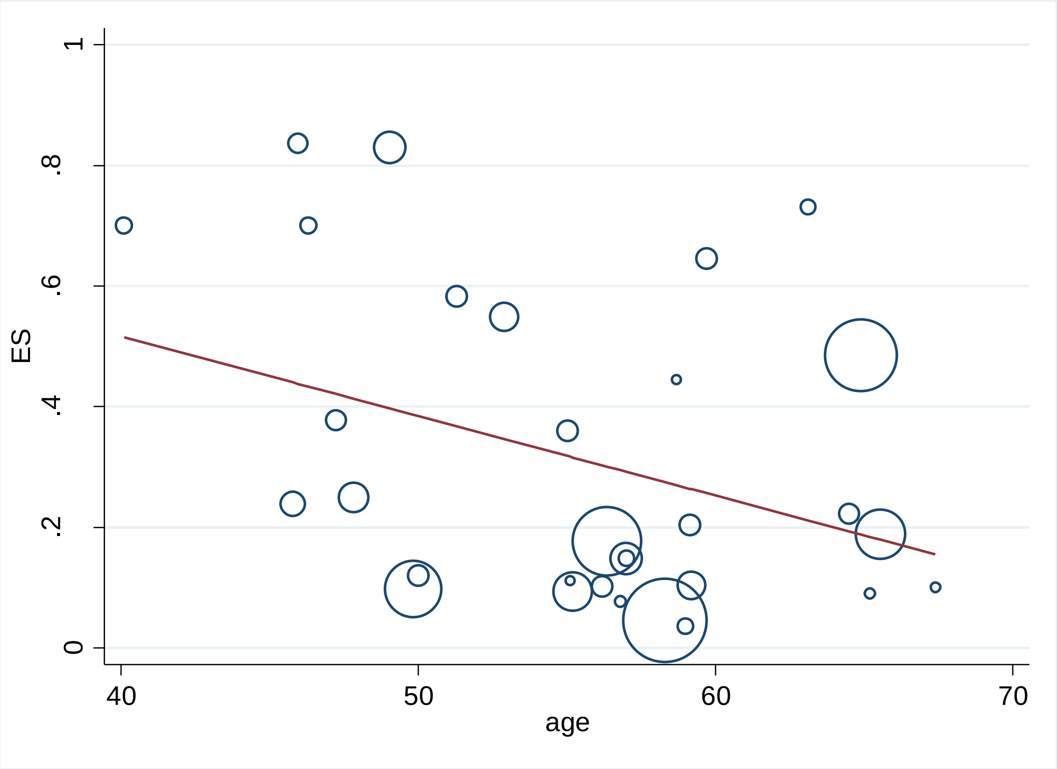

The red line exhibits the fitted regression line together with blue circles representing the estimates from each individual study, sized according to the precision of each estimate (the inverse of its within-study variance).

3.2.3 Effect of tobacco on the prevalence of depression among OLP patients.

Figure S9. Bubble plot graphically representing the potential effect of tobacco (% of smokers) on depression prevalence (derived from meta-regression analysis; see table 2).

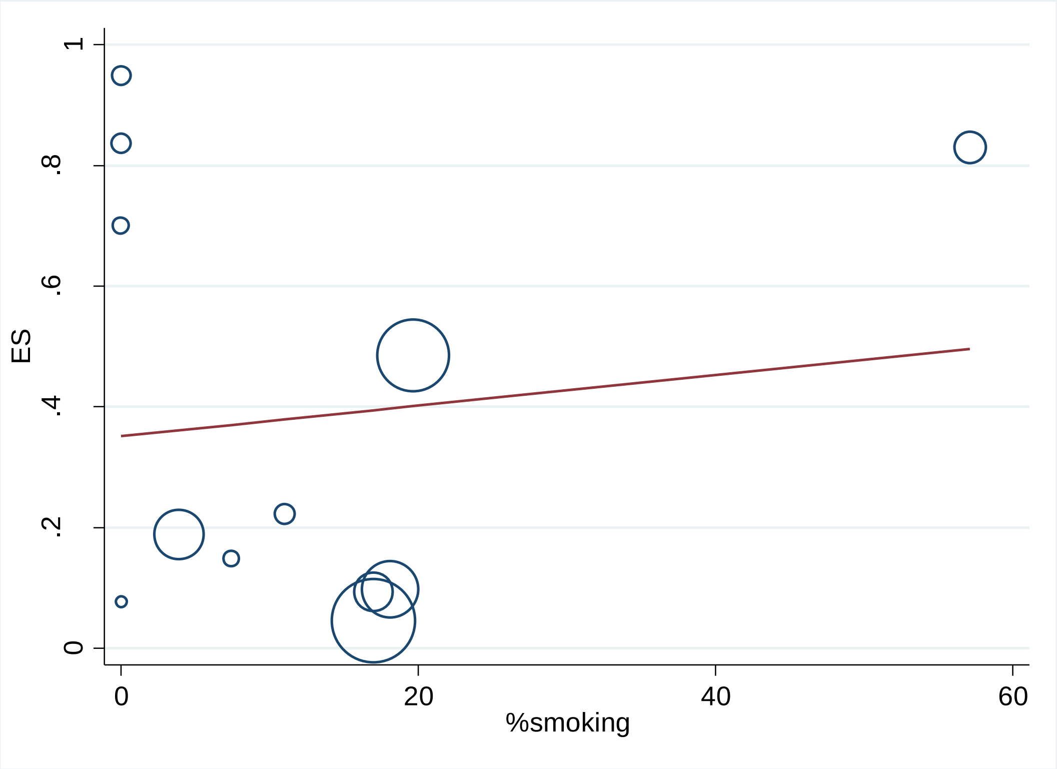

The red line exhibits the fitted regression line together with blue circles representing the estimates from each individual study, sized according to the precision of each estimate (the inverse of its within-study variance).

3.2.4 Effect of alcohol on the prevalence of depression among OLP patients.

Figure S10. Bubble plot graphically representing the potential effect of alcohol (% of drinkers) on depression prevalence (derived from meta-regression analysis; see table 2).

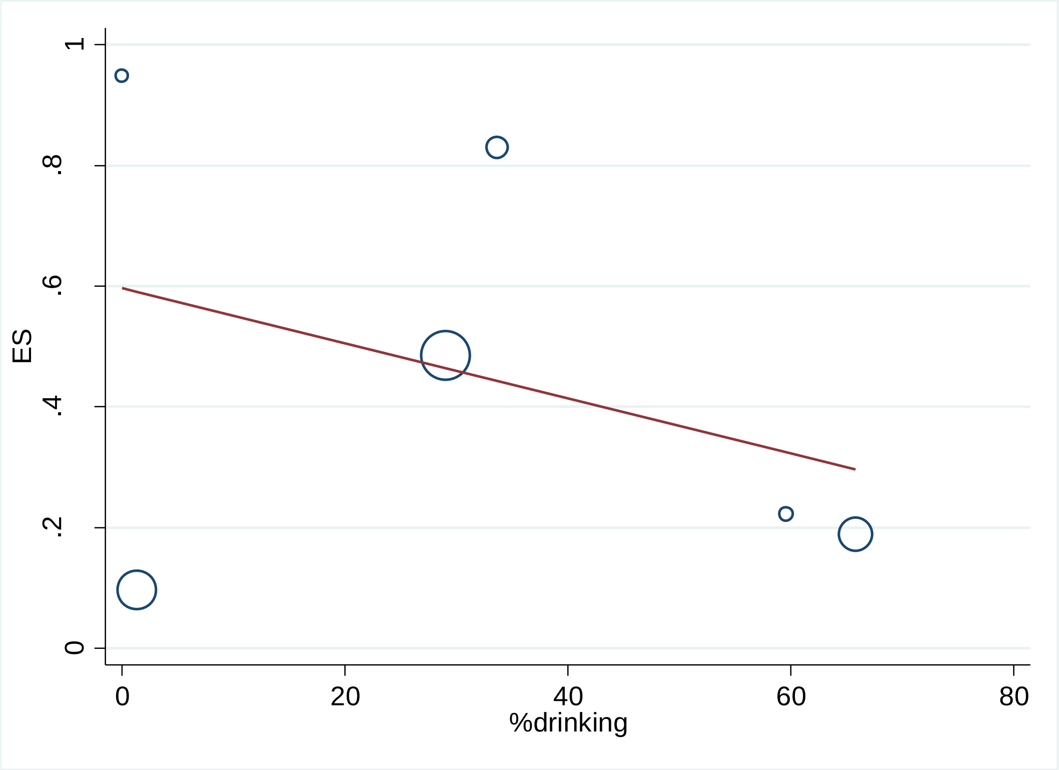

The red line exhibits the fitted regression line together with blue circles representing the estimates from each individual study, sized according to the precision of each estimate (the inverse of its within-study variance).

3.2.5 Effect of OLP type on the prevalence of depression among OLP patients.

Figure S11. Bubble plot graphically representing the potential effect of OLP type (% of patients with red lesions) on depression prevalence (derived from meta-regression analysis; see table 2).

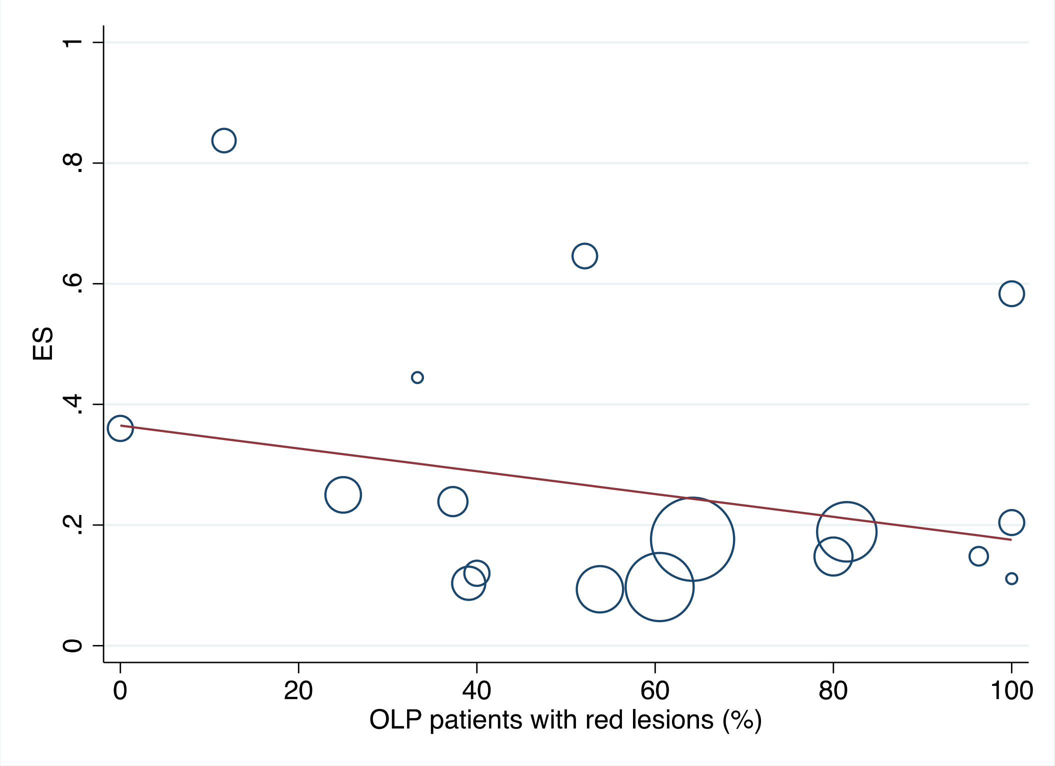

The red line exhibits the fitted regression line together with blue circles representing the estimates from each individual study, sized according to the precision of each estimate (the inverse of its within-study variance).

3.2.6 Effect of anxiety on the prevalence of depression among OLP patients.

Figure S12. Bubble plot graphically representing the potential effect of anxiety (% of patients affected by anxiety) on depression prevalence (derived from meta-regression analysis; see table 2).

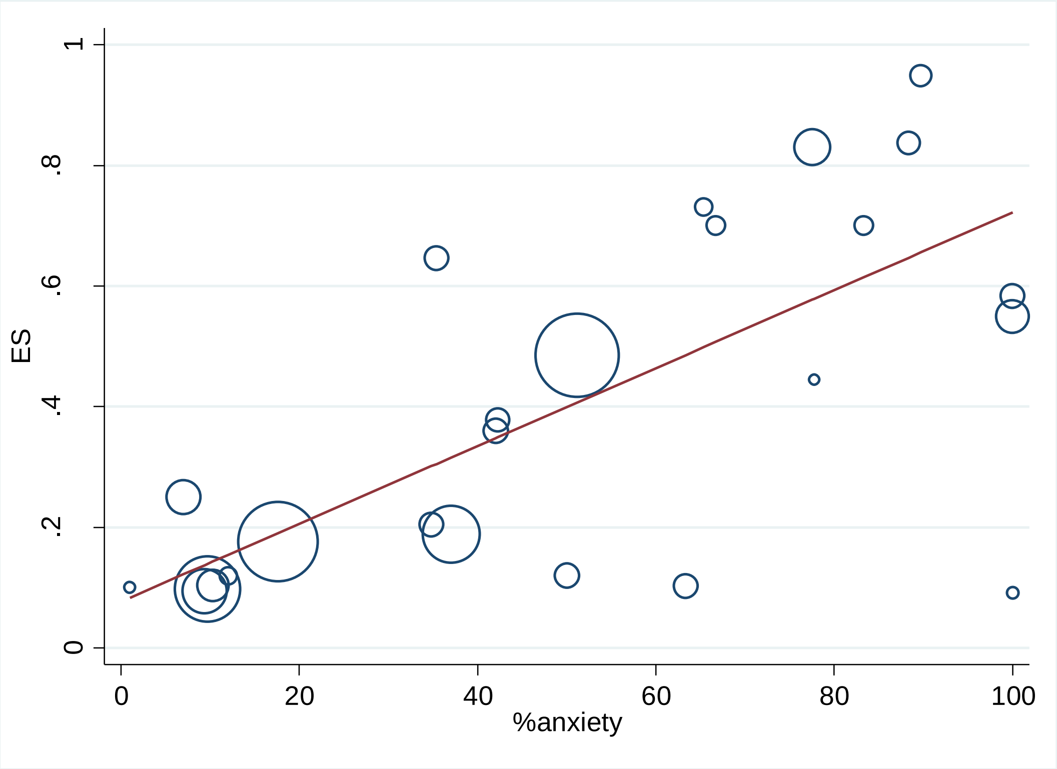

The red line exhibits the fitted regression line together with blue circles representing the estimates from each individual study, sized according to the precision of each estimate (the inverse of its within-study variance).

3.2.7 Effect of stress on the prevalence of depression among OLP patients.

Figure S13. Bubble plot graphically representing the potential effect of stress (% of patients affected by stress) on depression prevalence (derived from meta-regression analysis; see table 2).

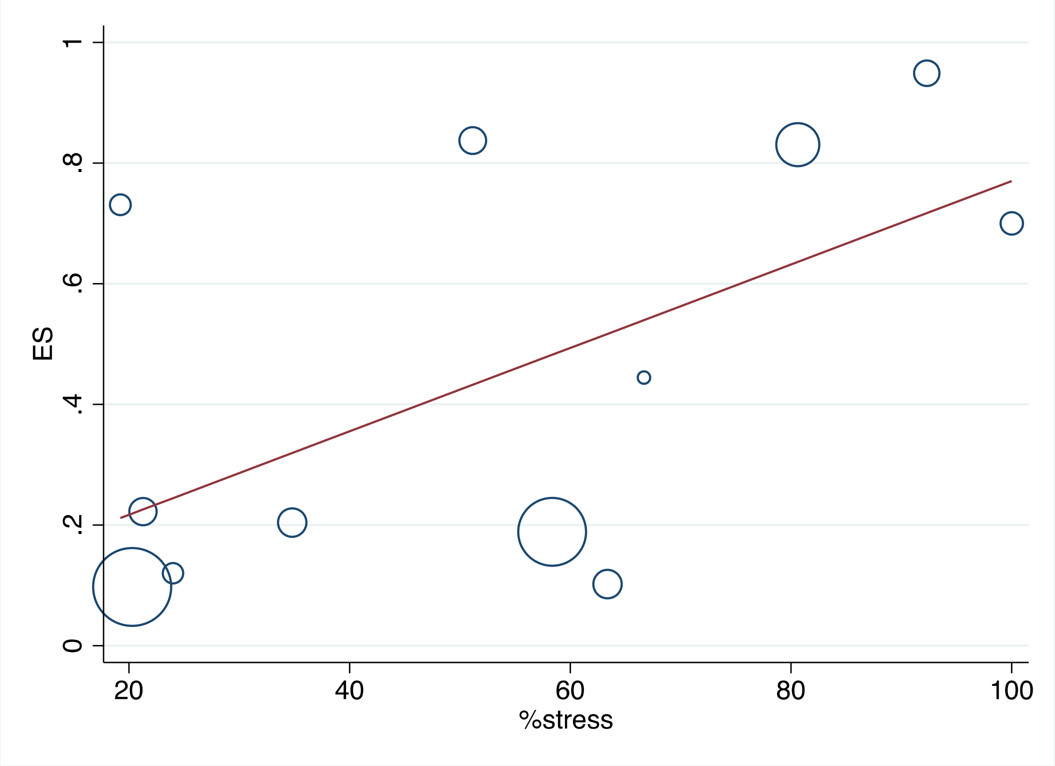

The red line exhibits the fitted regression line together with blue circles representing the estimates from each individual study, sized according to the precision of each estimate (the inverse of its within-study variance).

3.2.8 Effect of publication year on the prevalence of depression among OLP patients.

Figure S14. Bubble plot graphically representing the potential effect of publication year on depression prevalence (derived from meta-regression analysis; see table 2).

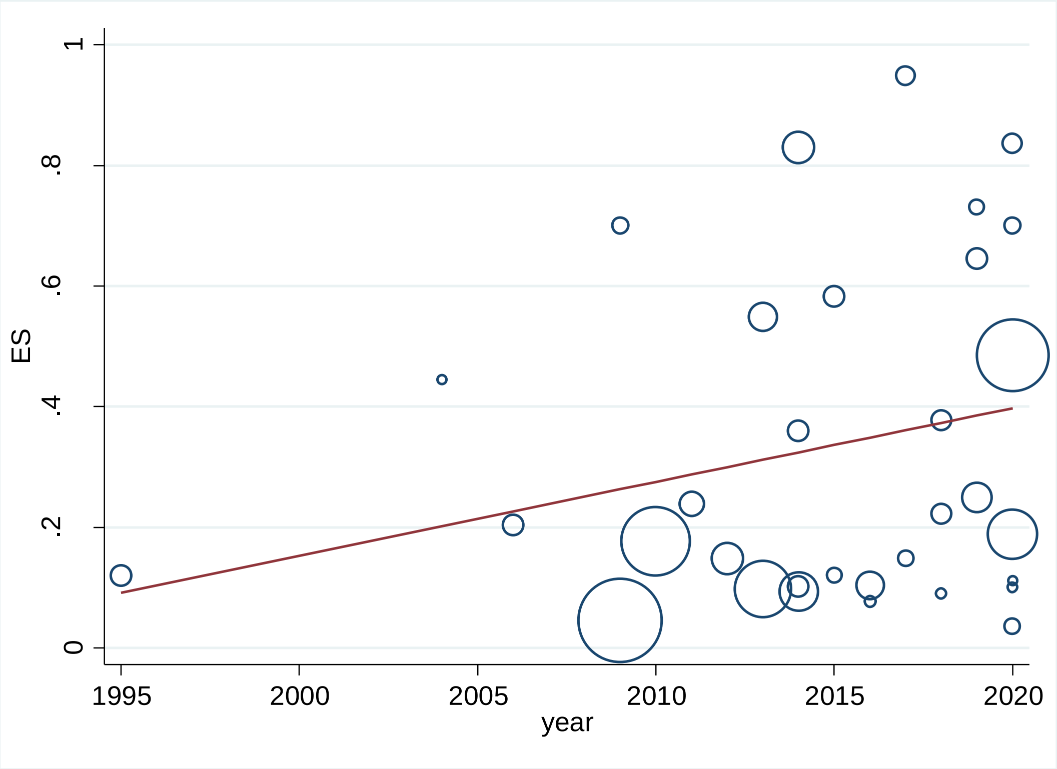

The red line exhibits the fitted regression line together with blue circles representing the estimates from each individual study, sized according to the precision of each estimate (the inverse of its within-study variance).

3.2.9 Effect of human development index on the prevalence of depression among OLP patients.

Figure S15. Bubble plot graphically representing the potential effect of human development index on depression prevalence (derived from meta-regression analysis; see table 2).

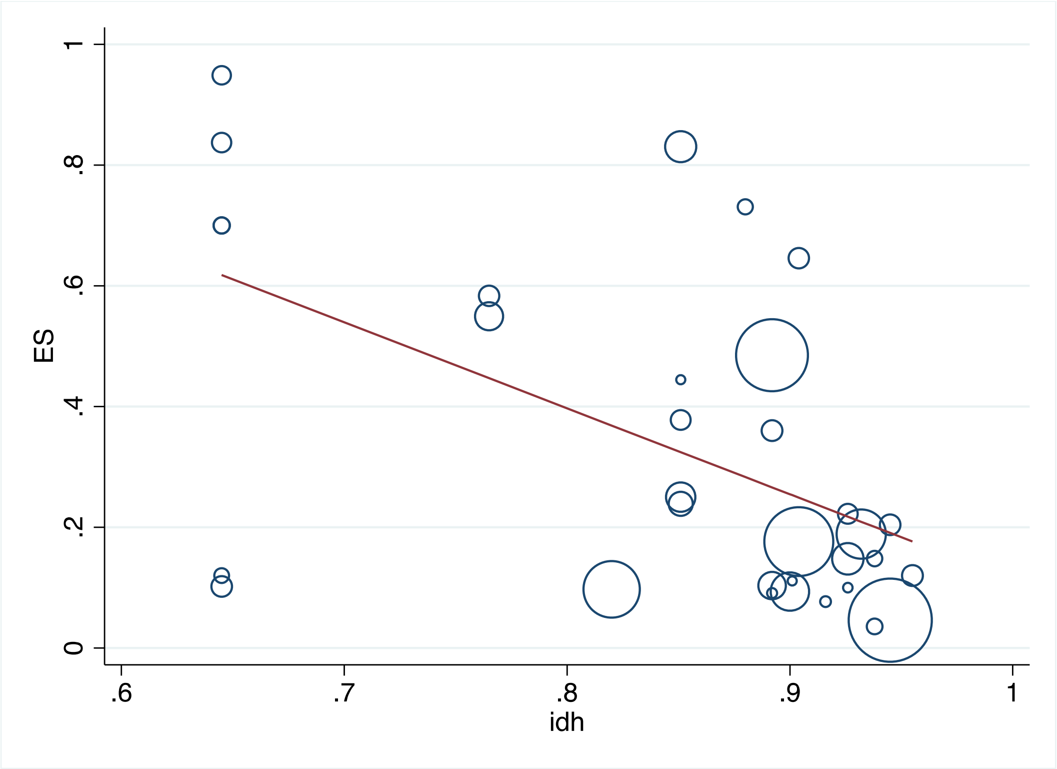

The red line exhibits the fitted regression line together with blue circles representing the estimates from each individual study, sized according to the precision of each estimate (the inverse of its within-study variance).

3.2.10 Effect of risk of bias on the prevalence of depression among OLP patients.

Figure S16. Bubble plot graphically representing the potential effect of risk of bias (expressed as overall score) on depression prevalence (derived from meta-regression analysis; see table 2).

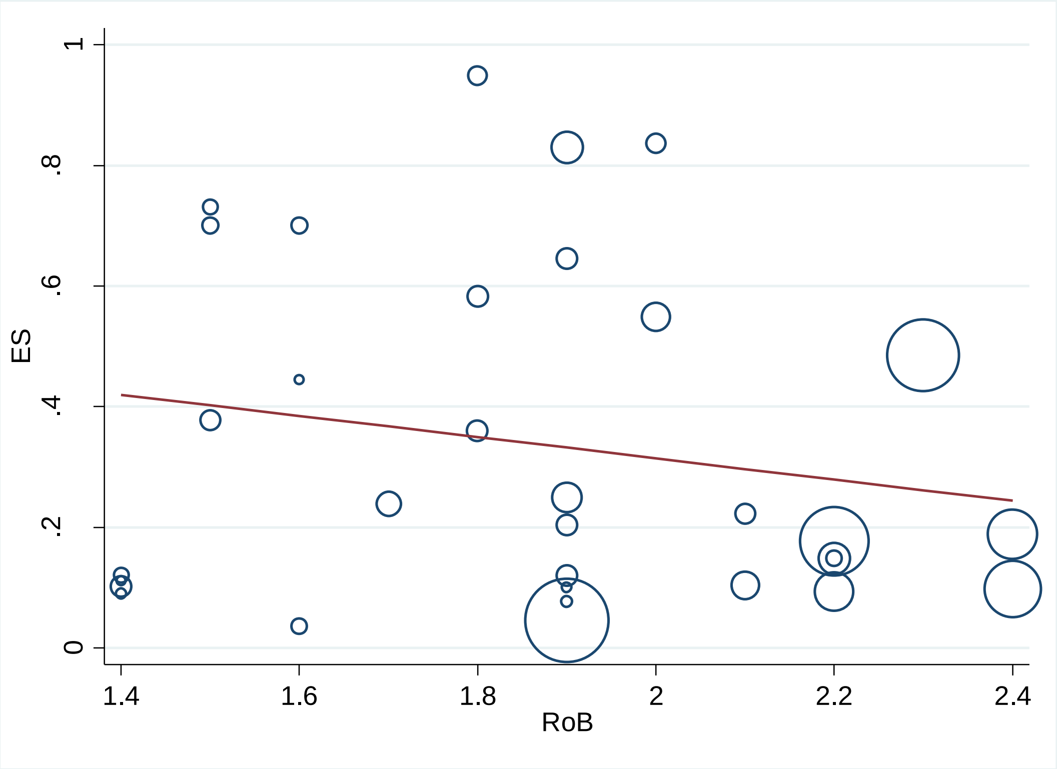

The red line exhibits the fitted regression line together with blue circles representing the estimates from each individual study, sized according to the precision of each estimate (the inverse of its within-study variance).

4. Anxiety meta-analyses.

4.1 Magnitude of association between OLP and anxiety.

Figure S17. Forest plot graphically representing the analysis of the magnitude of association -using OR as effect size measure- between anxiety and OLP.

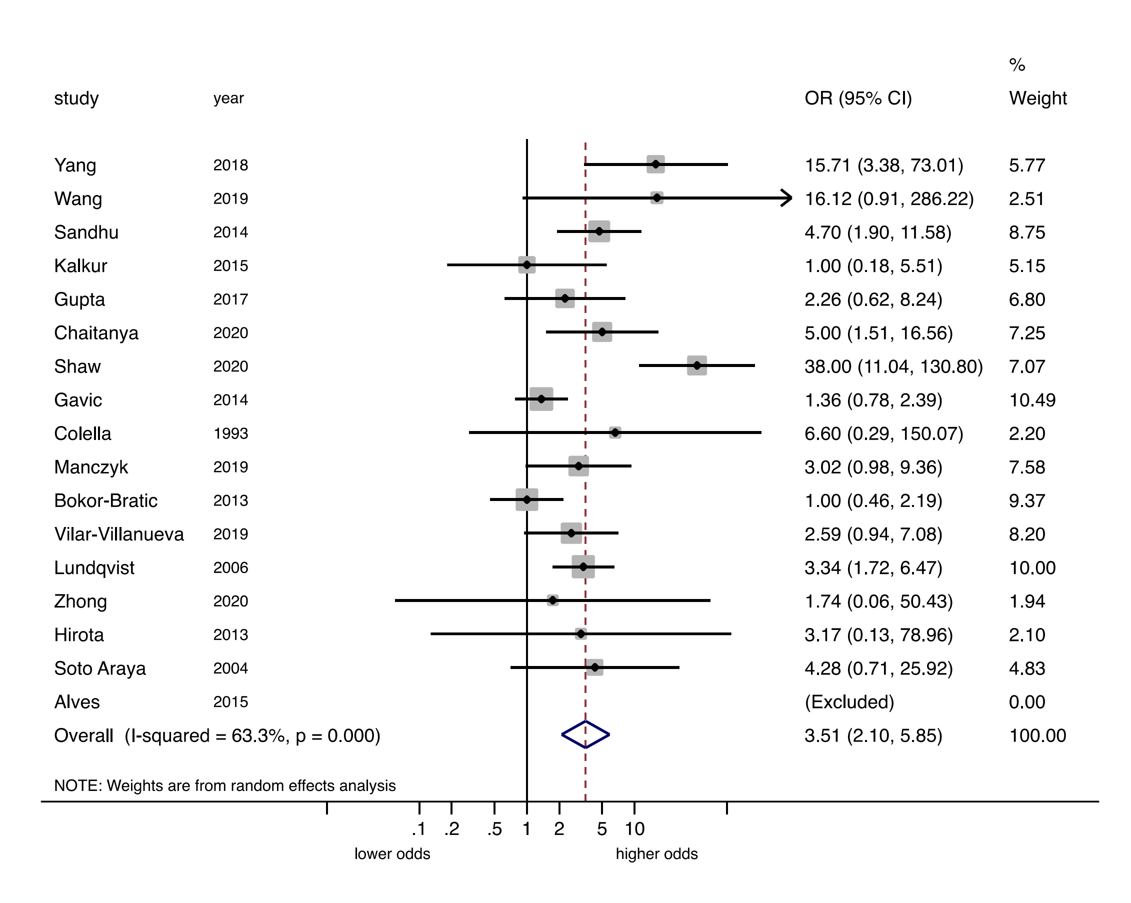

OR, odds ratio; CI, confidence interval; Random-effects model.

4.2 Prevalence of anxiety among OLP patients. Subgroup meta-analyses.

4.2.1 Prevalence of anxiety among OLP patients by continent.

Figure S18. Forest plot graphically representing the stratified analysis of the prevalence of anxiety -using pooled proportions as effect size measure- among OLP patients by continent.

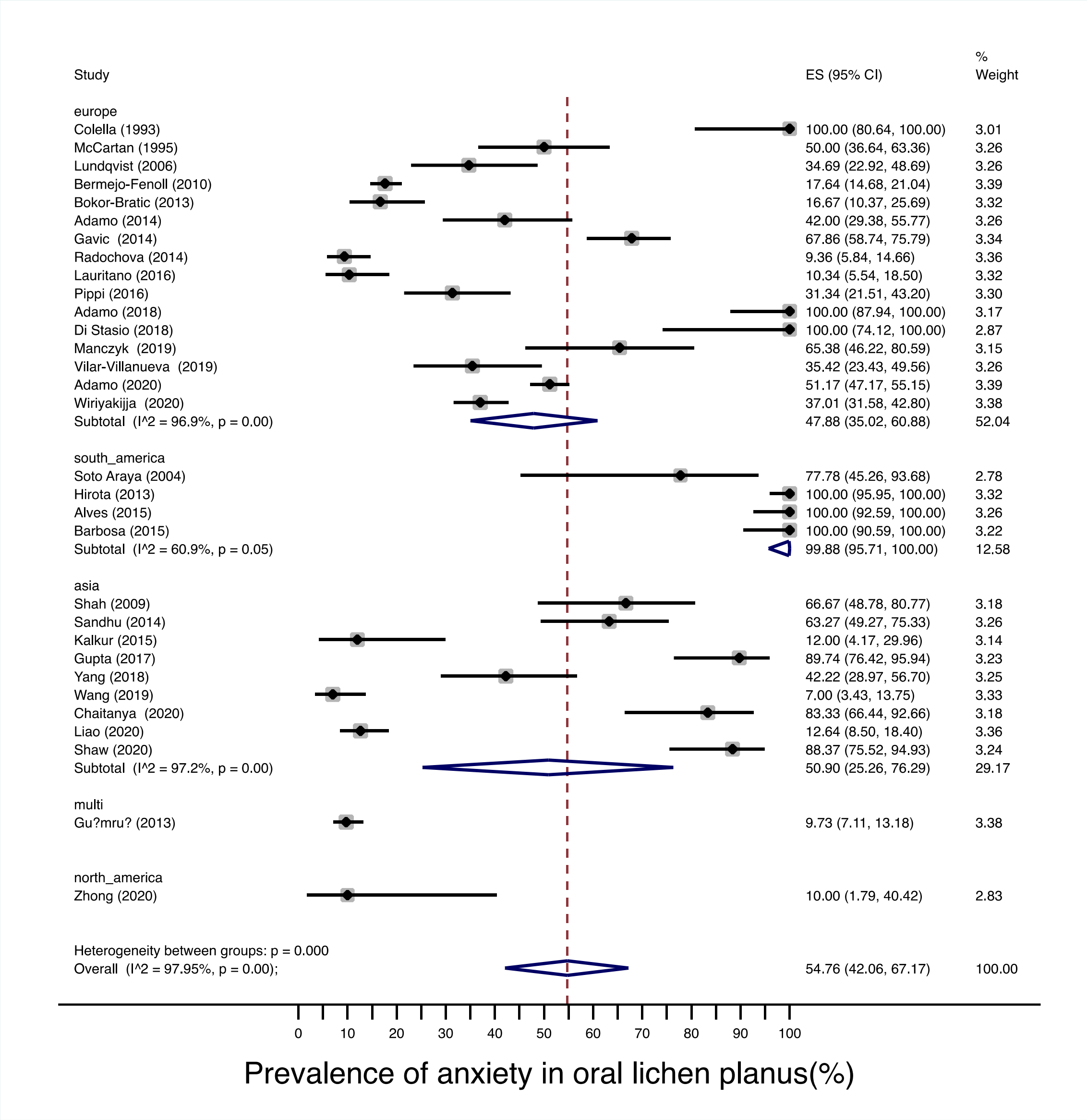

ES, estimation; CI, confidence interval; Random-effects model.

4.2.2 Prevalence of anxiety among OLP patients by diagnostic anxiety test.

Figure S19. Forest plot graphically representing the stratified analysis of the prevalence of anxiety -using pooled proportions as effect size measure- among OLP patients by diagnostic anxiety test.

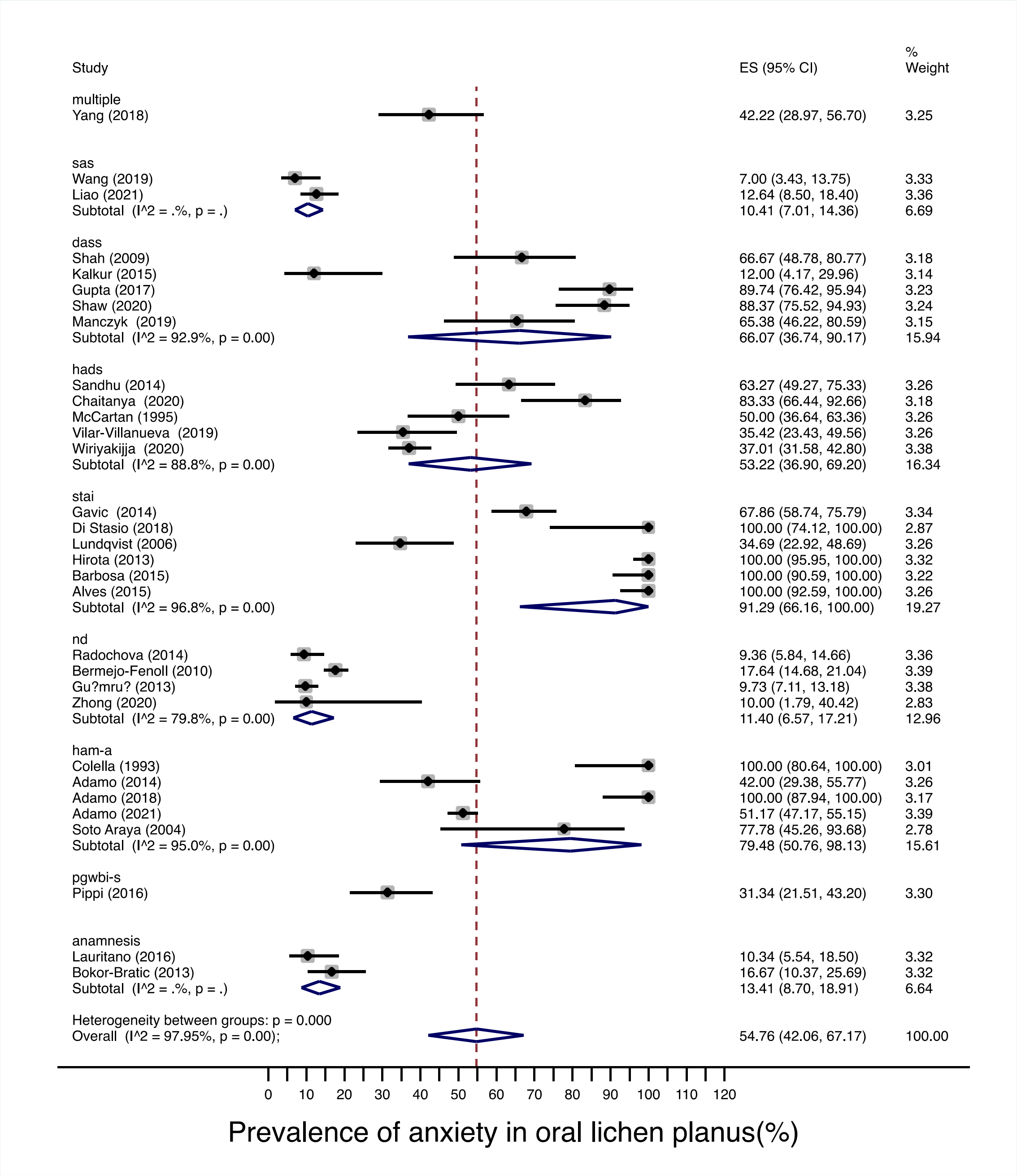

ES, estimation; CI, confidence interval; Random-effects model.

4.2.3 Prevalence of anxiety among OLP patients by specialist implied in diagnosis of anxiety.

Figure S20. Forest plot graphically representing the stratified analysis of the prevalence of anxiety -using pooled proportions as effect size measure- among OLP patients by specialist implied in diagnosis of anxiety.

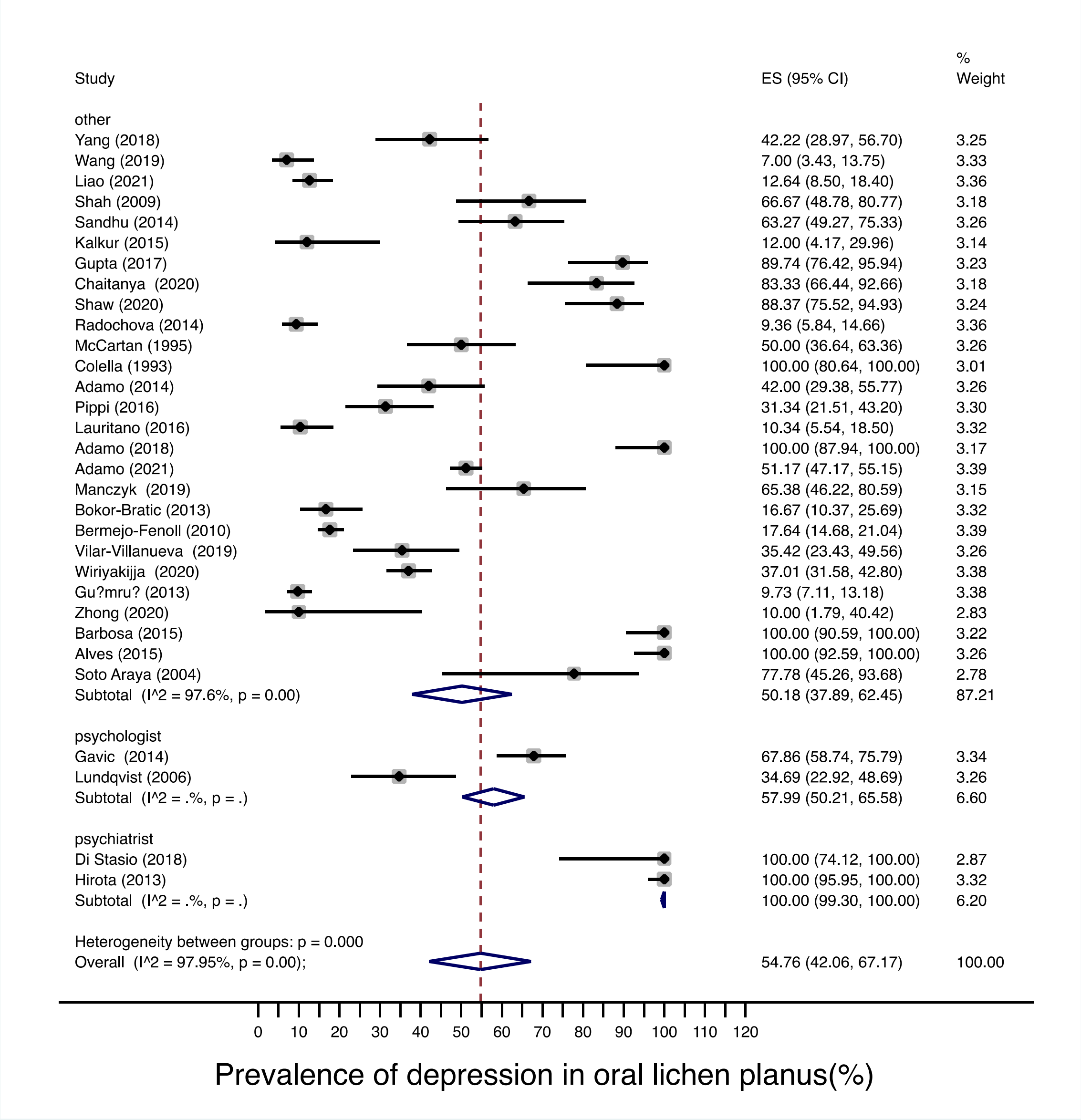

ES, estimation; CI, confidence interval; Random-effects model.

4.2.4 Prevalence of anxiety among OLP patients by publication language.

Figure S21. Forest plot graphically representing the stratified analysis of the prevalence of anxiety -using pooled proportions as effect size measure- among OLP patients by publication language.

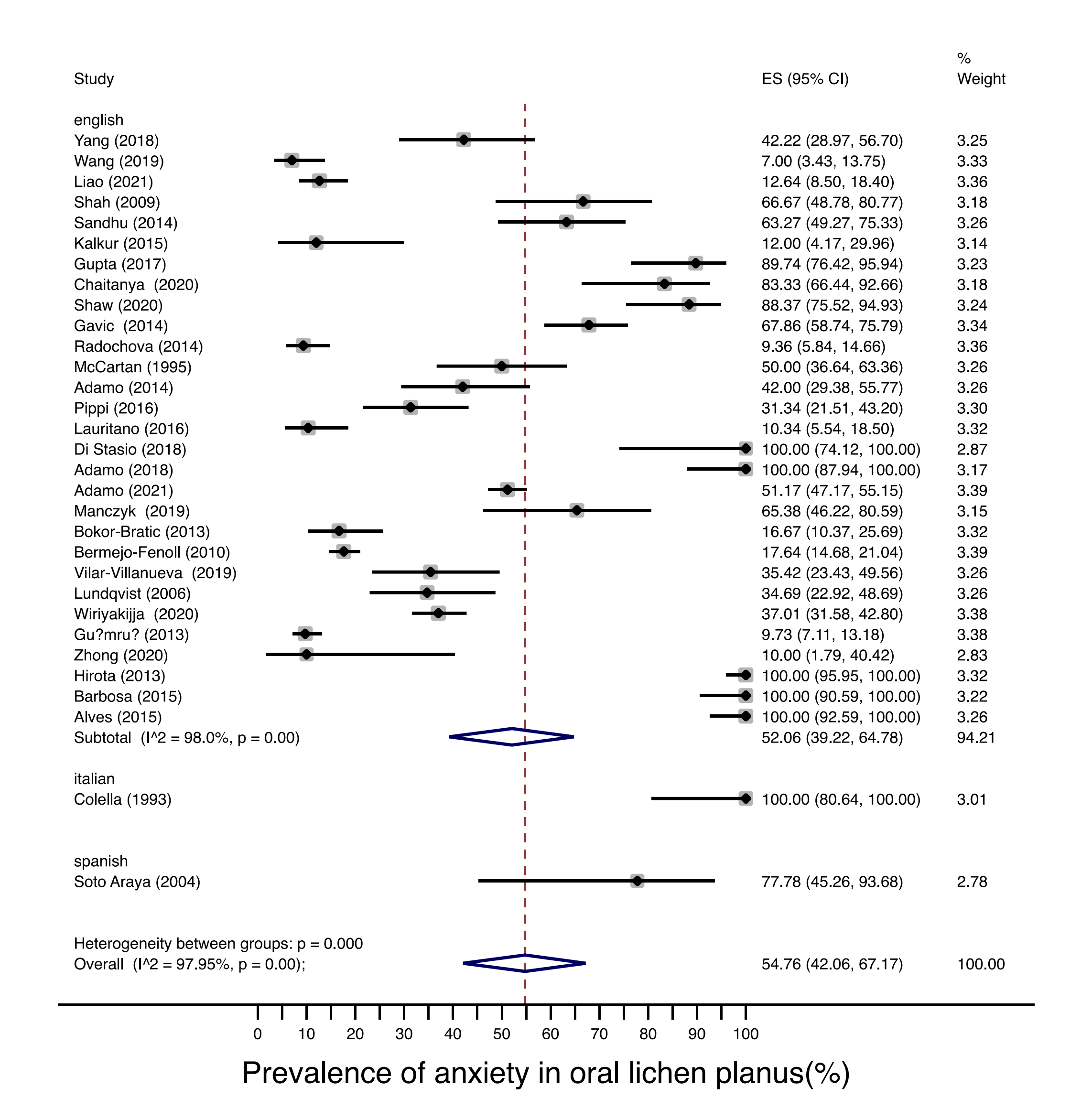

ES, estimation; CI, confidence interval; Random-effects model.

4.2.5 Prevalence of anxiety among OLP patients by sex.

Figure S22. Forest plot graphically representing the stratified analysis of the prevalence of anxiety -using pooled proportions as effect size measure- among OLP patients by sex.

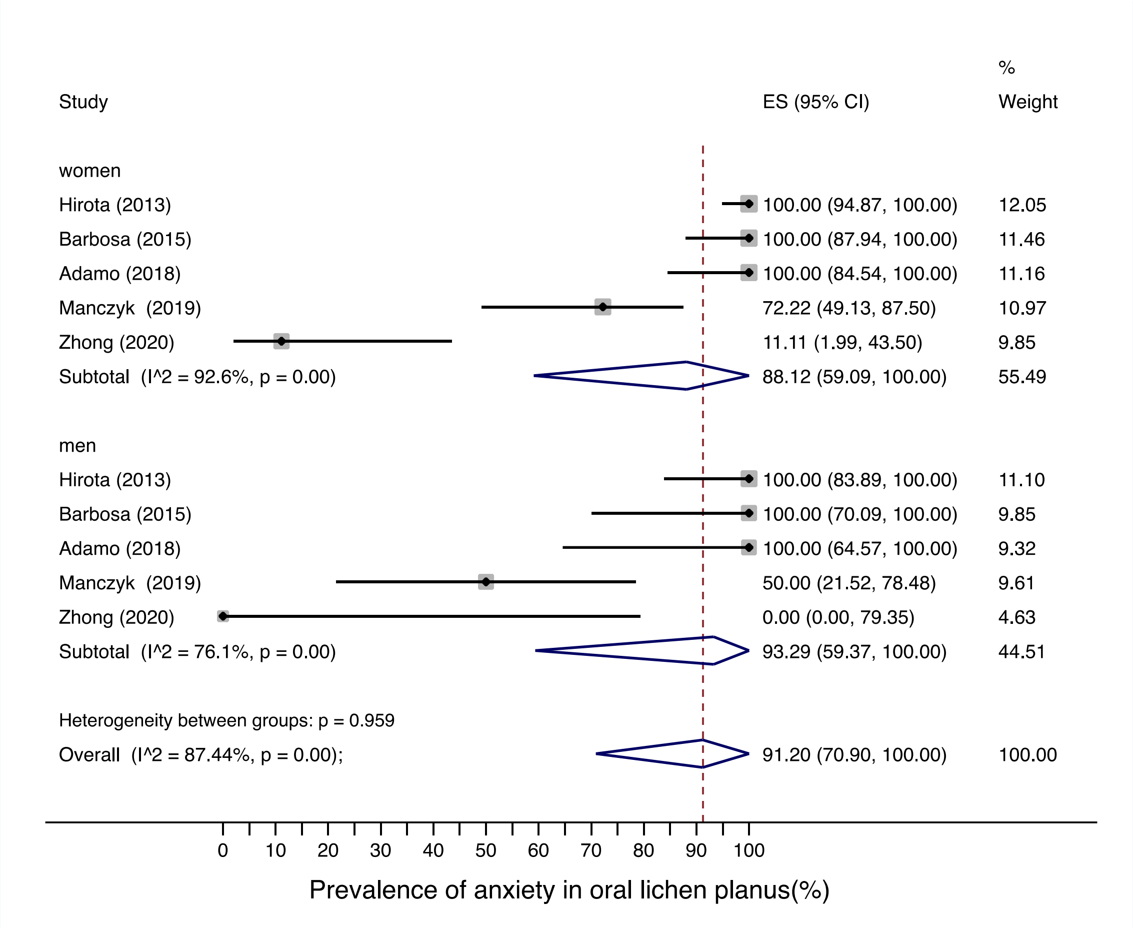

ES, estimation; CI, confidence interval; Random-effects model.

4.3 Prevalence of anxiety among OLP patients. Meta-regression analyses.

4.3.1 Effect of sex on the prevalence of anxiety among OLP patients.

Figure S23. Bubble plot graphically representing the potential effect of sex (% of female patients) on anxiety prevalence (derived from meta-regression analysis; see table 2).

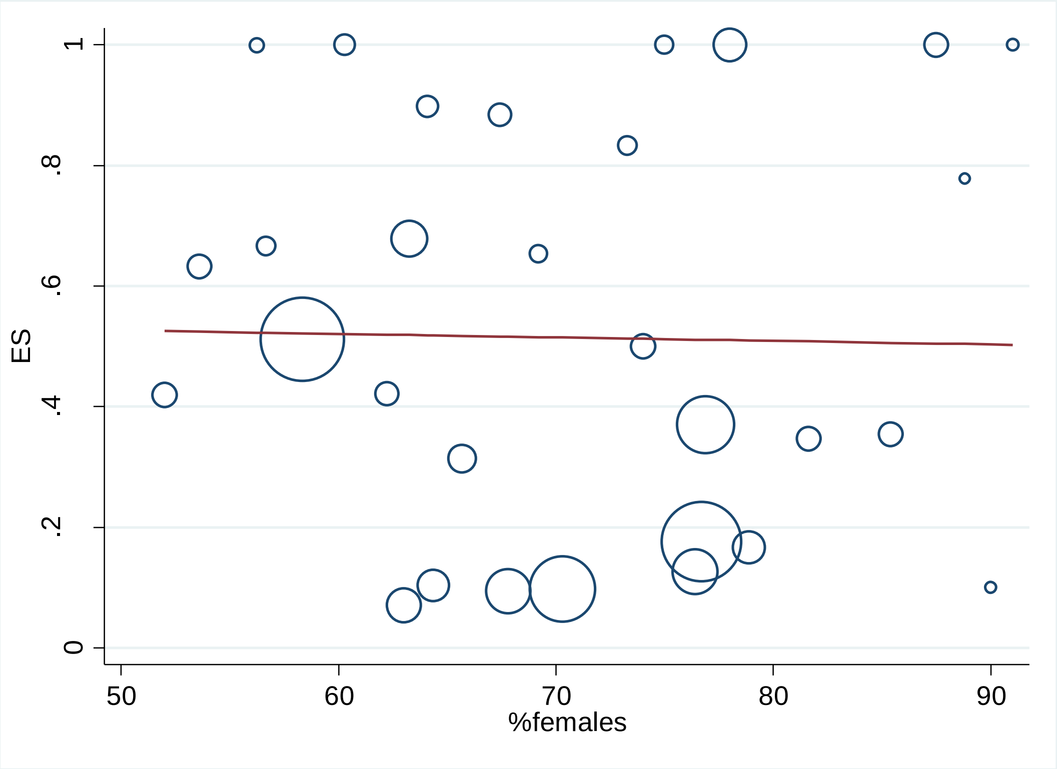

The red line exhibits the fitted regression line together with blue circles representing the estimates from each individual study, sized according to the precision of each estimate (the inverse of its within-study variance).

4.3.2 Effect of age on the prevalence of anxiety among OLP patients.

Figure S24. Bubble plot graphically representing the potential effect of age (expressed in years) on anxiety prevalence (derived from meta-regression analysis; see table 2).

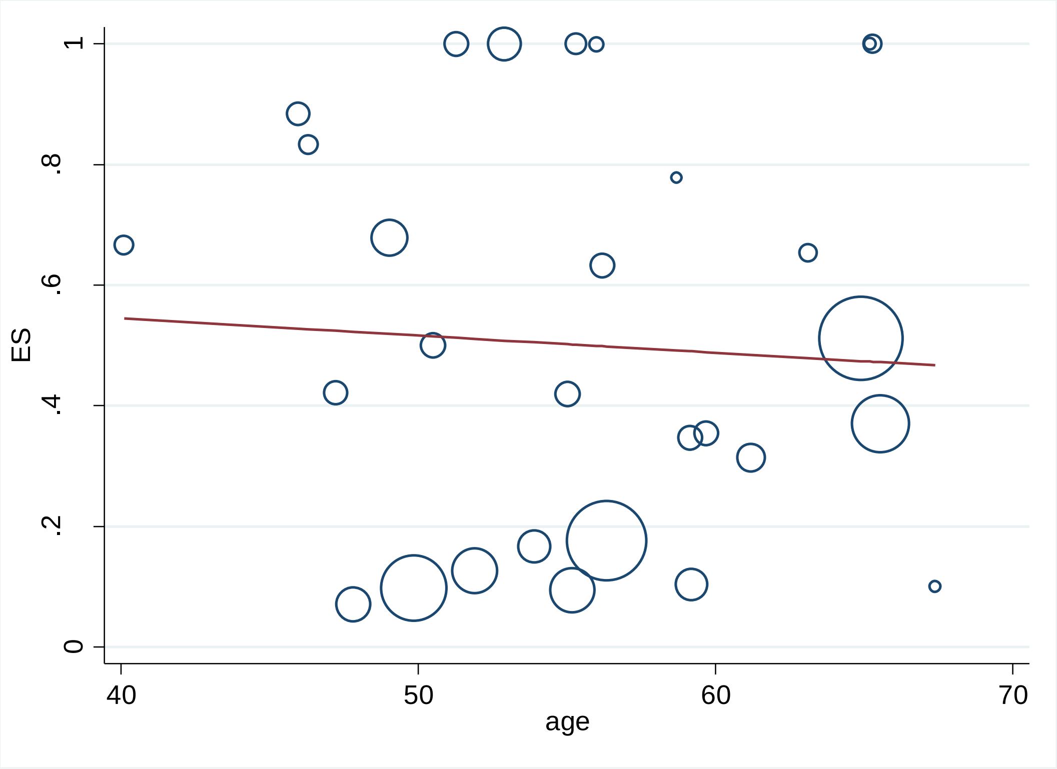

The red line exhibits the fitted regression line together with blue circles representing the estimates from each individual study, sized according to the precision of each estimate (the inverse of its within-study variance).

4.3.3 Effect of tobacco on the prevalence of anxiety among OLP patients.

Figure S25. Bubble plot graphically representing the potential effect of tobacco (% of smokers) on anxiety prevalence (derived from meta-regression analysis; see table 2).

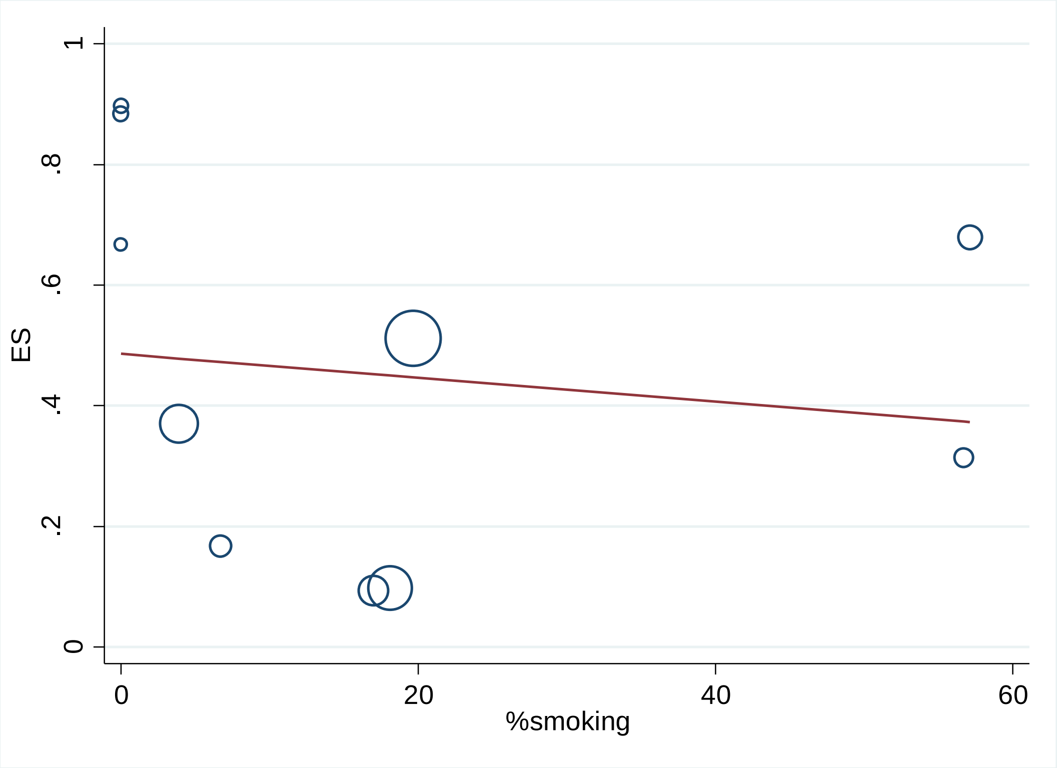

The red line exhibits the fitted regression line together with blue circles representing the estimates from each individual study, sized according to the precision of each estimate (the inverse of its within-study variance).

4.3.4 Effect of alcohol on the prevalence of anxiety among OLP patients.

Figure S26. Bubble plot graphically representing the potential effect of alcohol (% of drinkers) on anxiety prevalence (derived from meta-regression analysis; see table 2).

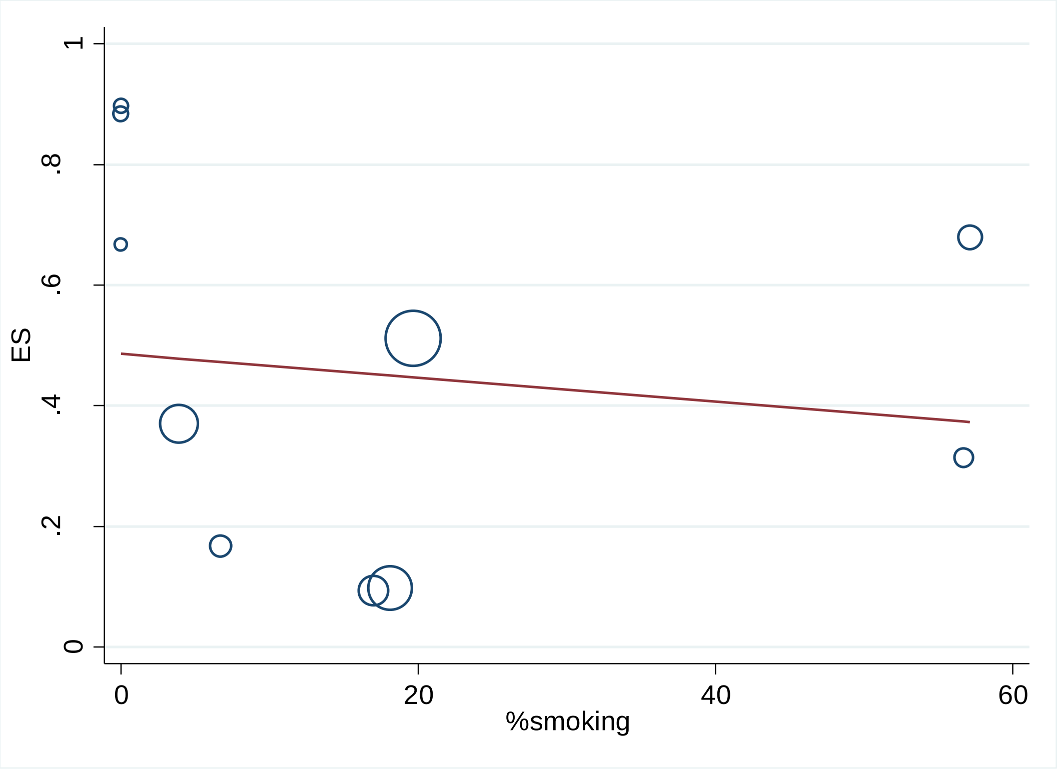

The red line exhibits the fitted regression line together with blue circles representing the estimates from each individual study, sized according to the precision of each estimate (the inverse of its within-study variance).

4.3.5 Effect of OLP type on the prevalence of anxiety among OLP patients.

Figure S27. Bubble plot graphically representing the potential effect of OLP type (% of patients with red lesions) on anxiety prevalence (derived from meta-regression analysis; see table 2).

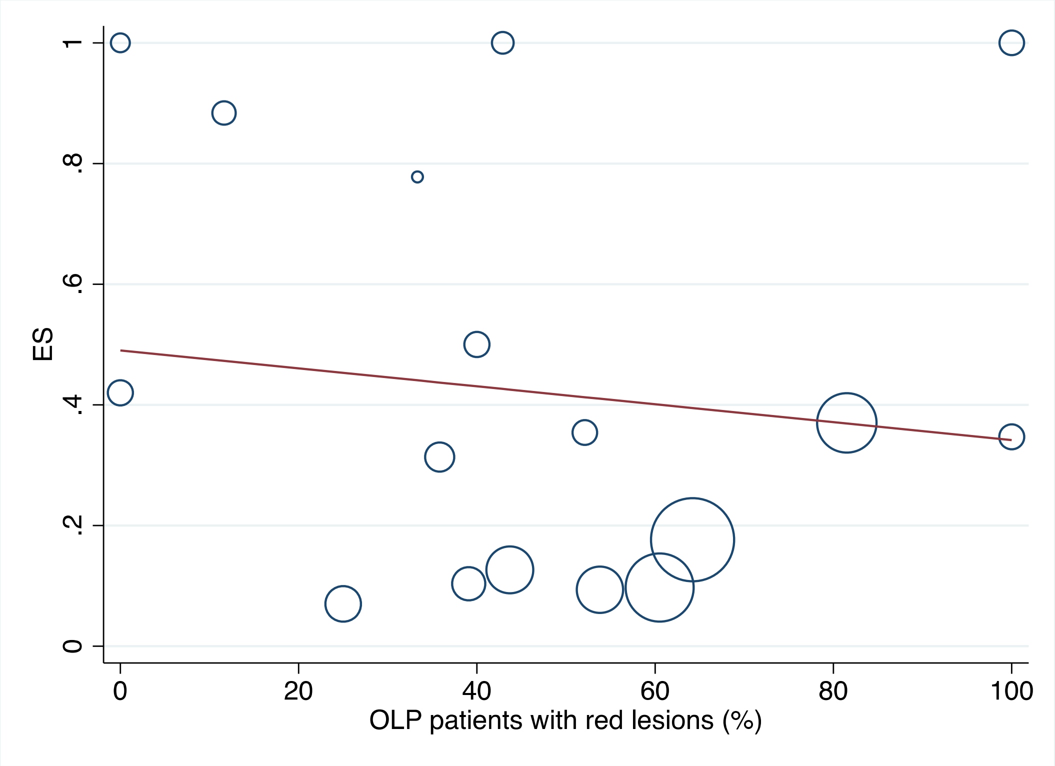

The red line exhibits the fitted regression line together with blue circles representing the estimates from each individual study, sized according to the precision of each estimate (the inverse of its within-study variance).

4.3.6 Effect of publication year on the prevalence of anxiety among OLP patients.

Figure S28. Bubble plot graphically representing the potential effect of publication year on anxiety prevalence (derived from meta-regression analysis; see table 2).

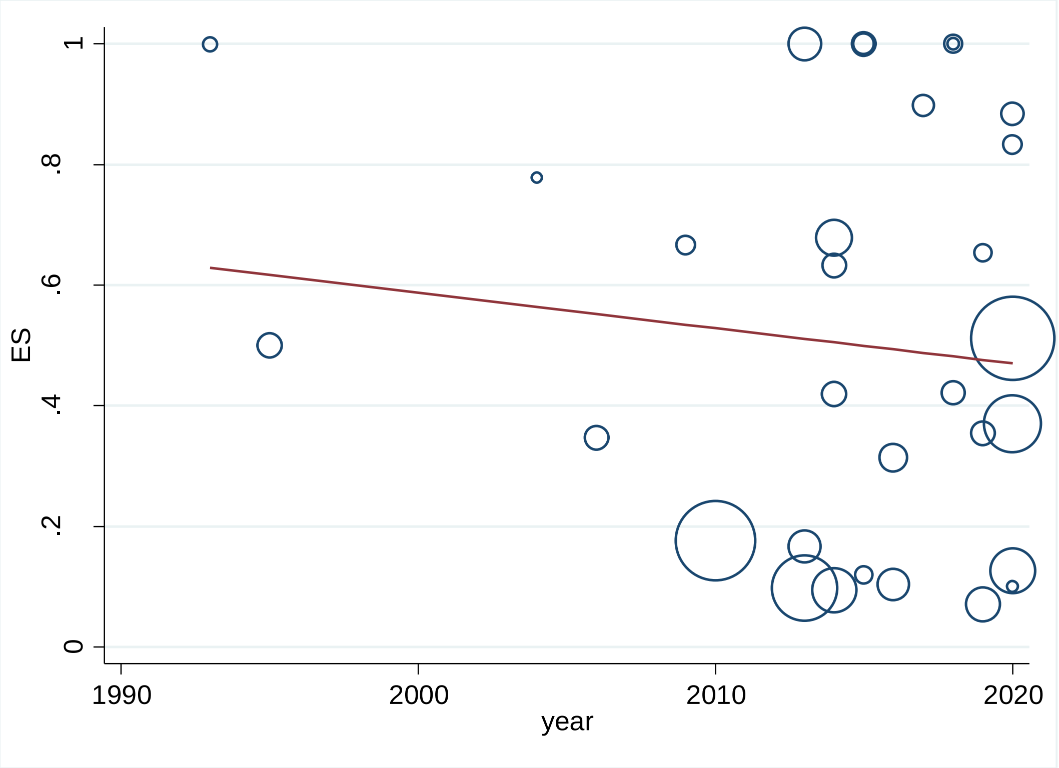

The red line exhibits the fitted regression line together with blue circles representing the estimates from each individual study, sized according to the precision of each estimate (the inverse of its within-study variance).

4.3.7 Effect of human development index on the prevalence of anxiety among OLP patients.

Figure S29. Bubble plot graphically representing the potential effect of human development index on anxiety prevalence (derived from meta-regression analysis; see table 2).

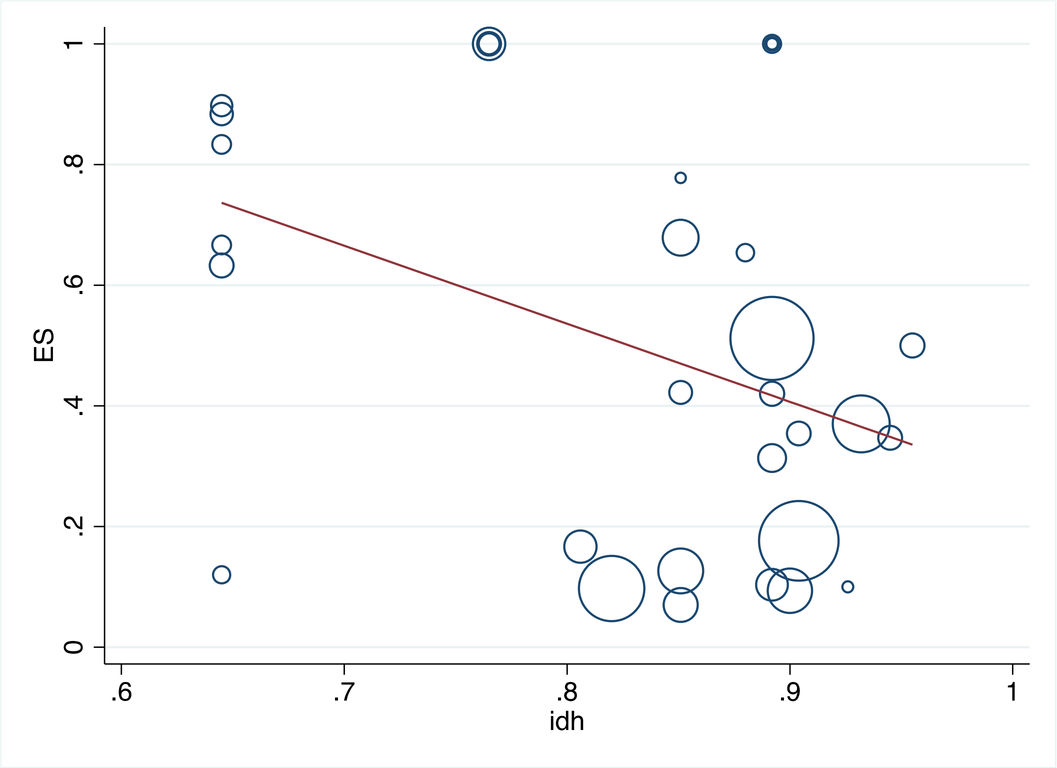

The red line exhibits the fitted regression line together with blue circles representing the estimates from each individual study, sized according to the precision of each estimate (the inverse of its within-study variance).

4.3.8 Effect of risk of bias on the prevalence of anxiety among OLP patients.

Figure S30. Bubble plot graphically representing the potential effect of risk of bias (expressed as overall score) on anxiety prevalence (derived from meta-regression analysis; see table 2).

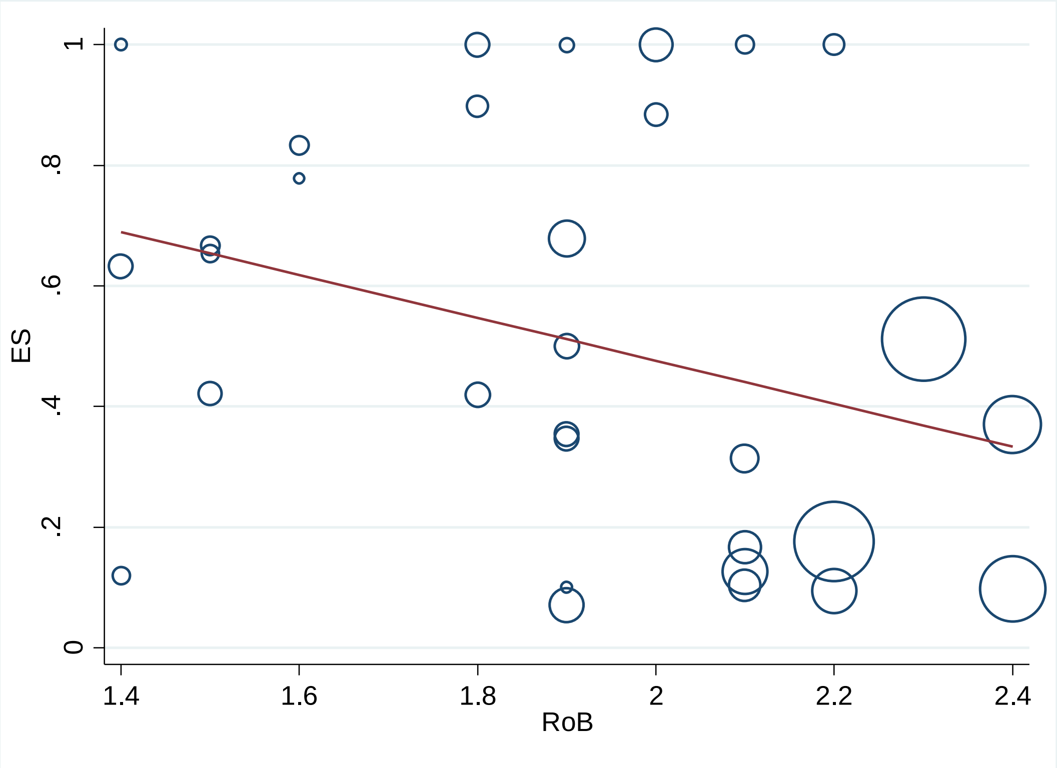

The red line exhibits the fitted regression line together with blue circles representing the estimates from each individual study, sized according to the precision of each estimate (the inverse of its within-study variance).

5. Stress meta-analyses.

5.1 Magnitude of association between OLP and stress.

Figure S31. Forest plot graphically representing the analysis of the magnitude of association -using OR as effect size measure- between stress and OLP.

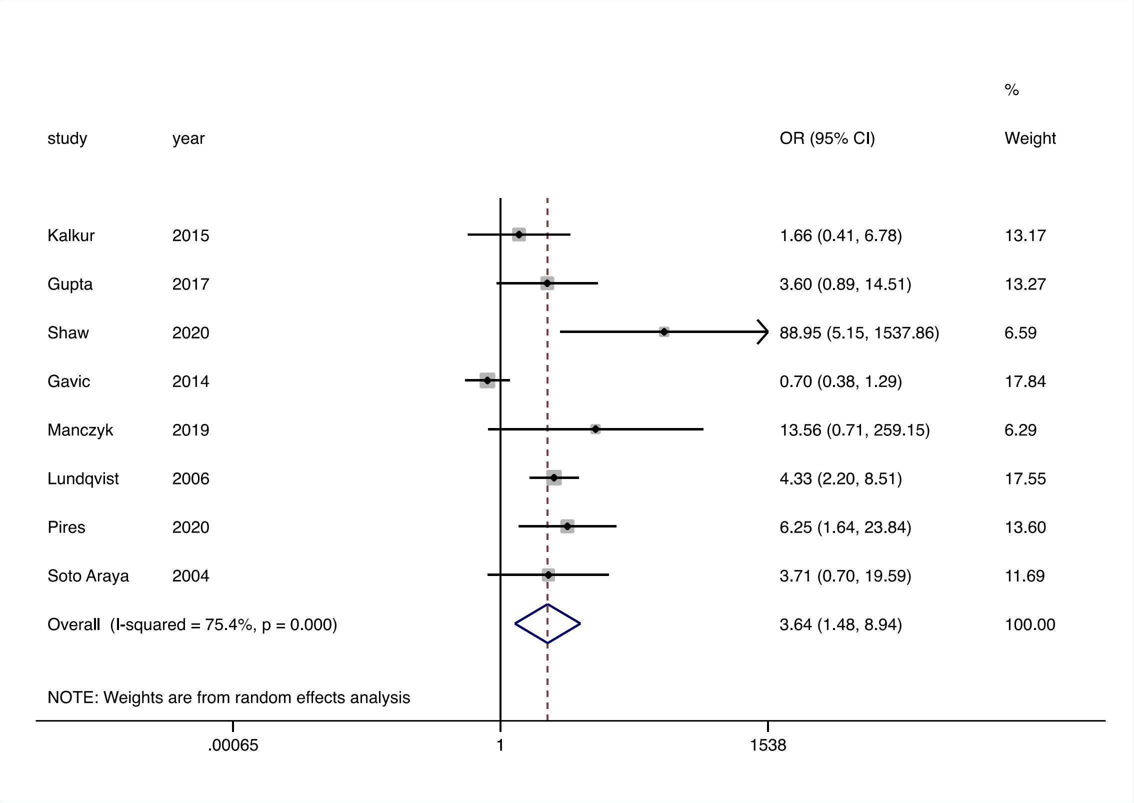

OR, odds ratio; CI, confidence interval; Random-effects model.

5.2 Prevalence of stress among OLP patients. Subgroup meta-analyses.

5.2.1 Prevalence of stress among OLP patients by continent.

Figure S32. Forest plot graphically representing the stratified analysis of the prevalence of stress -using pooled proportions as effect size measure- among OLP patients by continent.

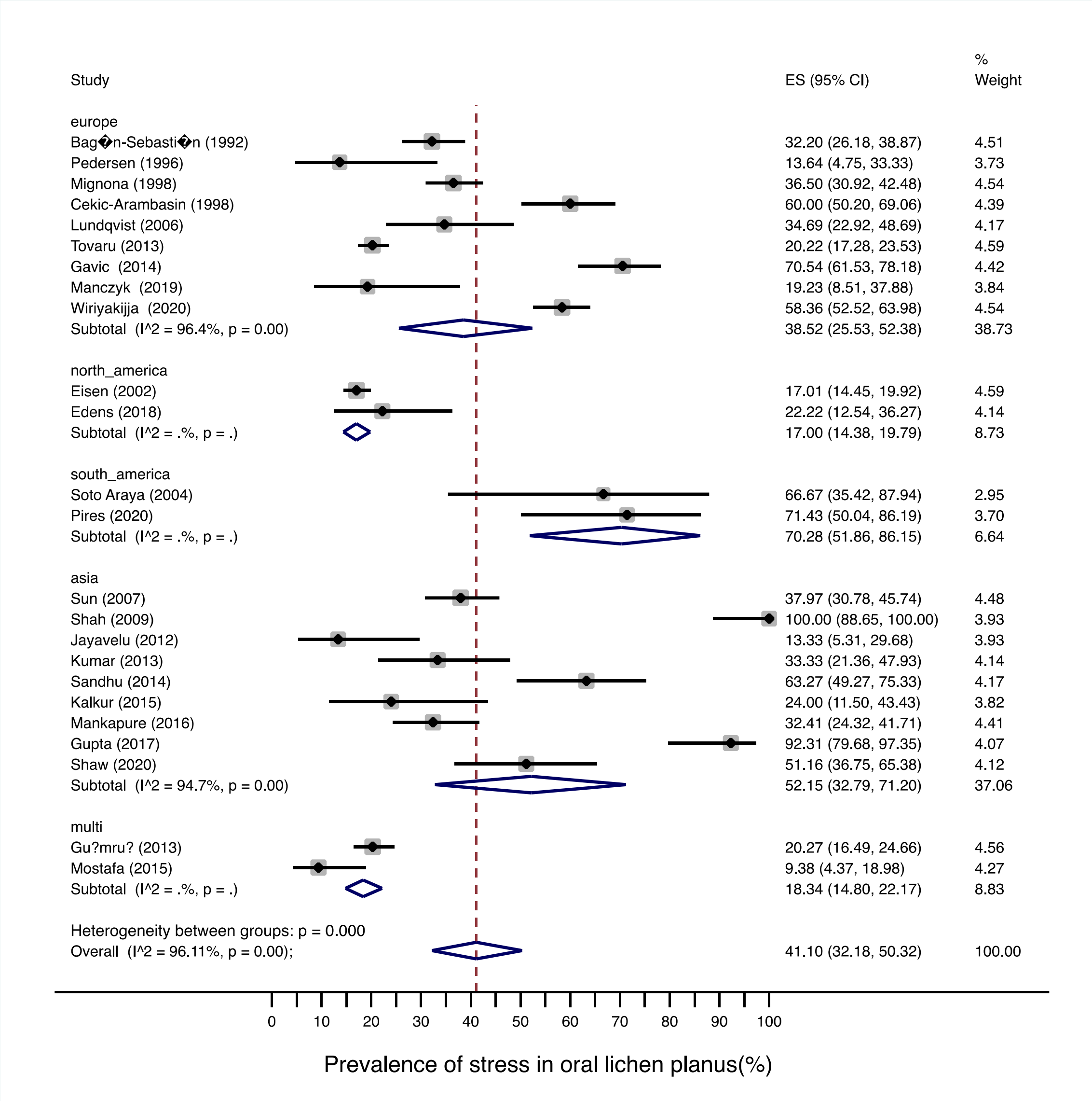

ES, estimation; CI, confidence interval; Random-effects model.

5.2.2 Prevalence of stress among OLP patients by diagnostic stress test.

Figure S33. Forest plot graphically representing the stratified analysis of the prevalence of stress -using pooled proportions as effect size measure- among OLP patients by diagnostic stress test.

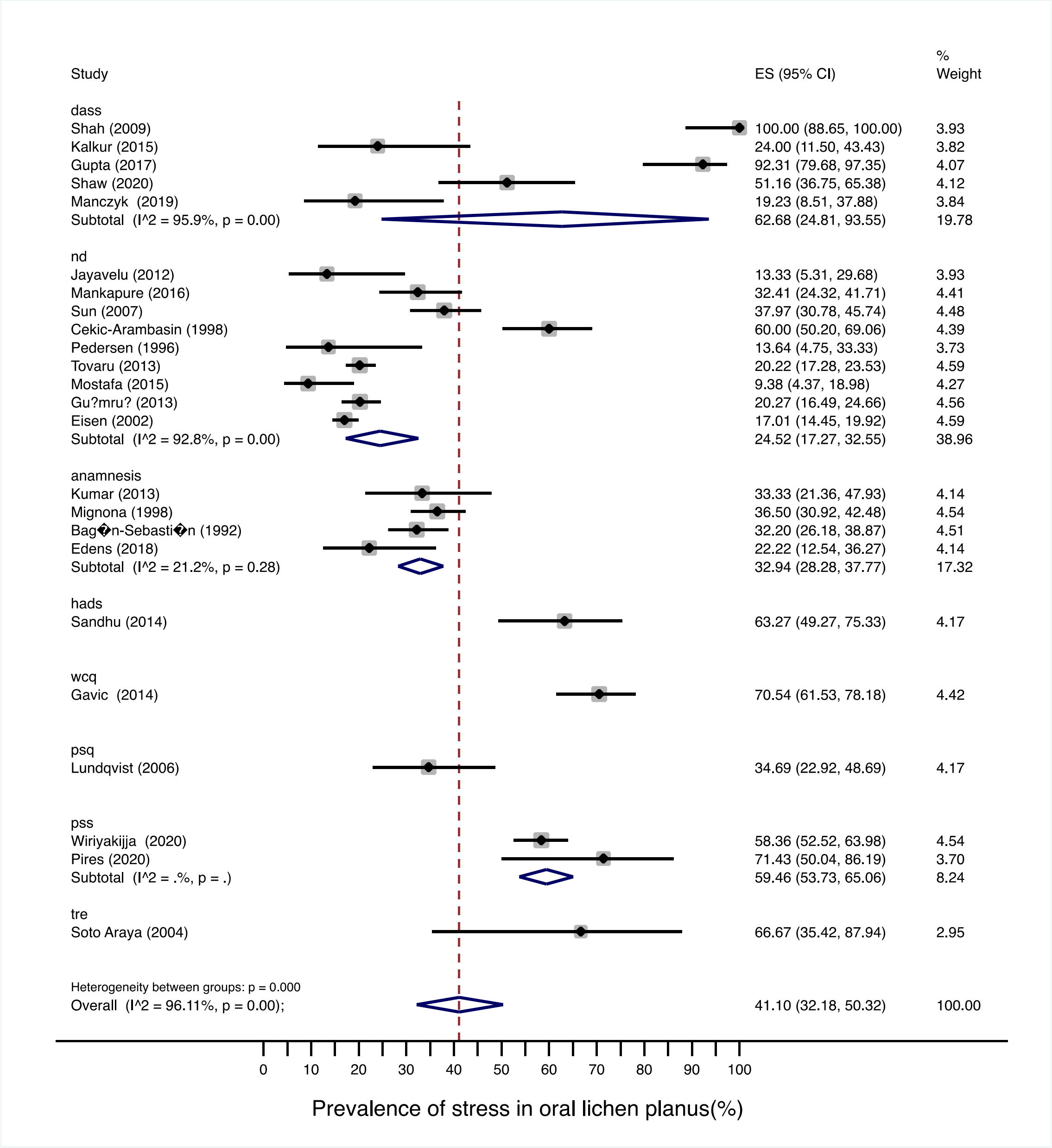

ES, estimation; CI, confidence interval; Random-effects model.

5.2.3 Prevalence of stress among OLP patients by specialist implied in diagnosis of stress.

Figure S34. Forest plot graphically representing the stratified analysis of the prevalence of stress -using pooled proportions as effect size measure- among OLP patients by specialist implied in diagnosis of stress.

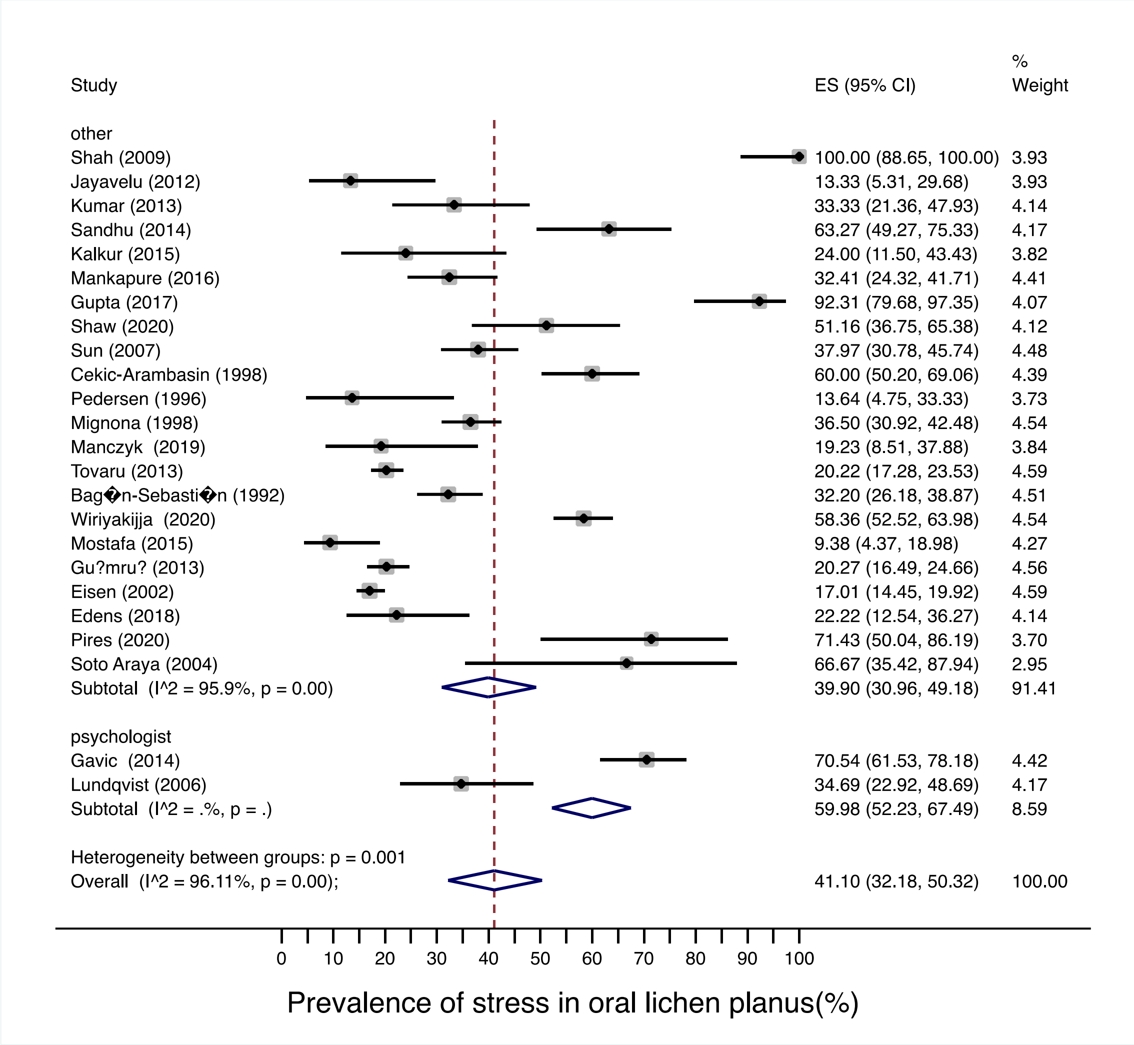

ES, estimation; CI, confidence interval; Random-effects model.

5.2.4 Prevalence of stress among OLP patients by publication language.

Figure S35. Forest plot graphically representing the stratified analysis of the prevalence of stress -using pooled proportions as effect size measure- among OLP patients by publication language.

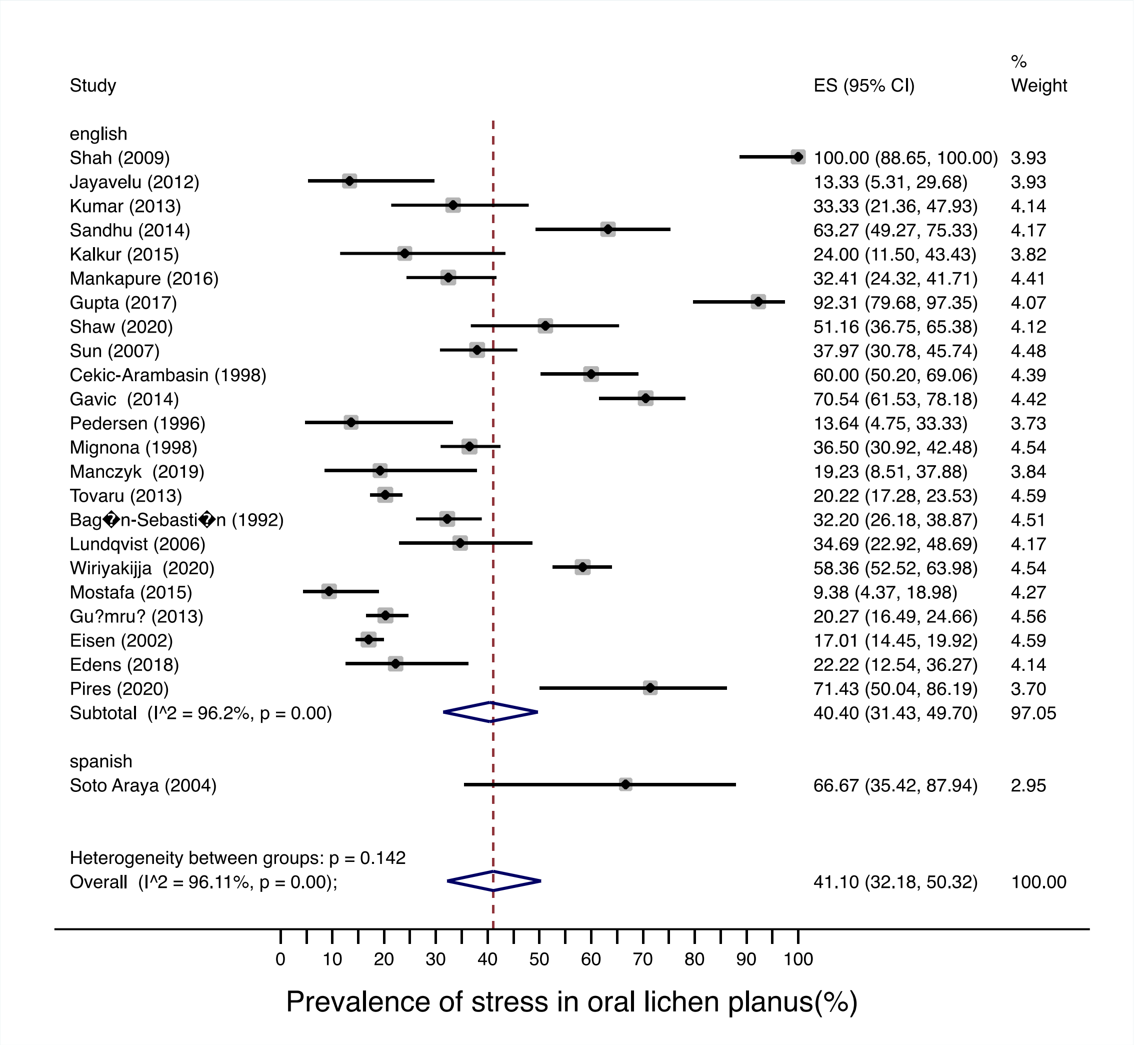

ES, estimation; CI, confidence interval; Random-effects model.

5.2.5 Prevalence of stress among OLP patients by sex.

Figure S36. Forest plot graphically representing the stratified analysis of the prevalence of stress -using pooled proportions as effect size measure- among OLP patients by sex.

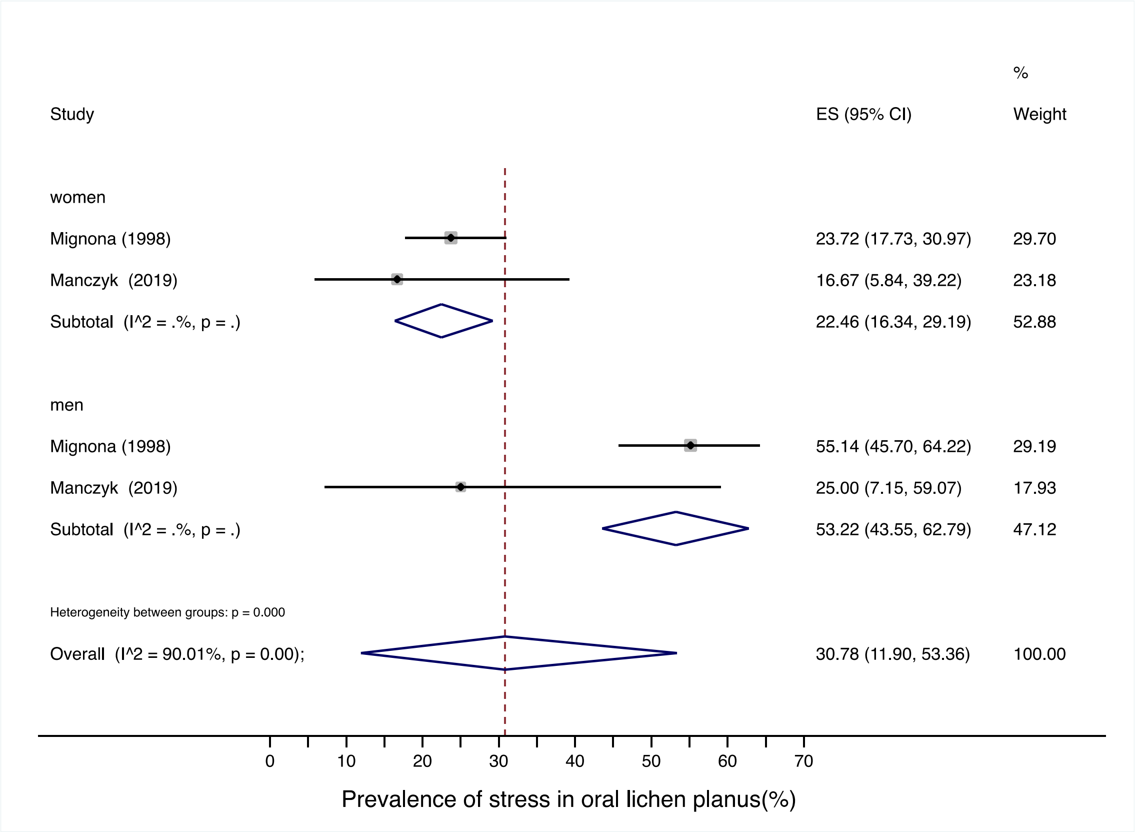

ES, estimation; CI, confidence interval; Random-effects model.

5.3 Prevalence of stress among OLP patients. Univariable meta-regression analyses.

5.3. Effect of sex on the prevalence of stress among OLP patients.

Figure S37. Bubble plot graphically representing the potential effect of sex (% of female patients) on stress prevalence (derived from meta-regression analysis; see table 2).

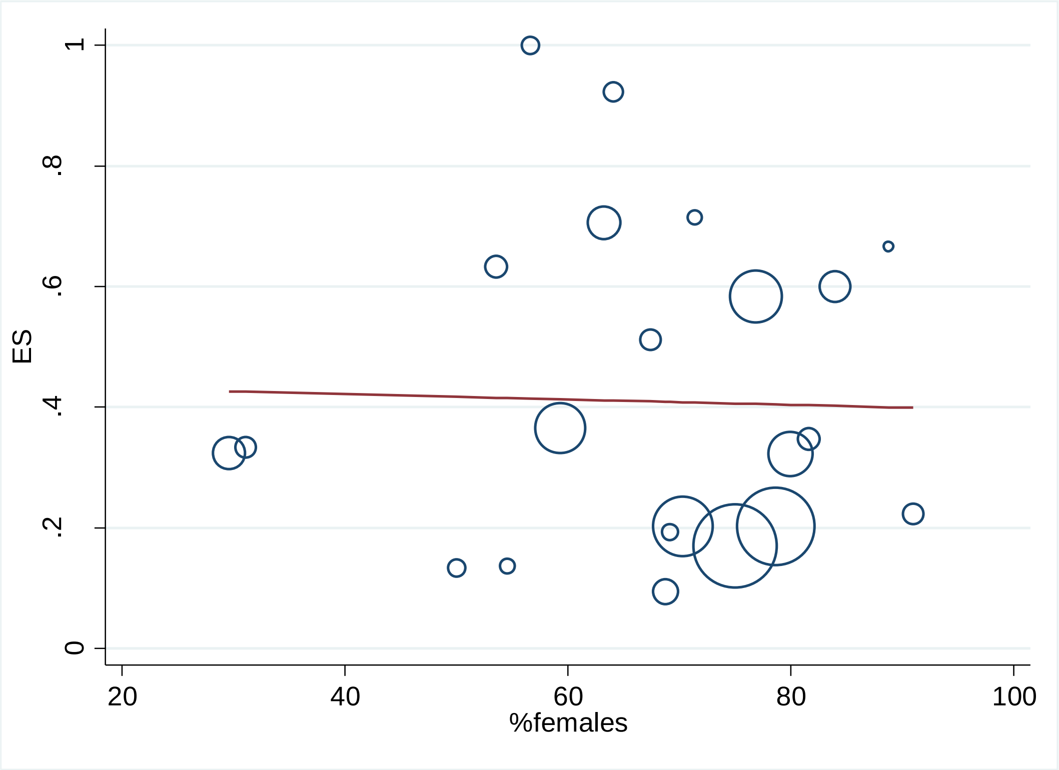

The red line exhibits the fitted regression line together with blue circles representing the estimates from each individual study, sized according to the precision of each estimate (the inverse of its within-study variance).

5.3.2 Effect of age on the prevalence of stress among OLP patients.

Figure S38. Bubble plot graphically representing the potential effect of age (expressed in years) on stress prevalence (derived from meta-regression analysis; see table 2).

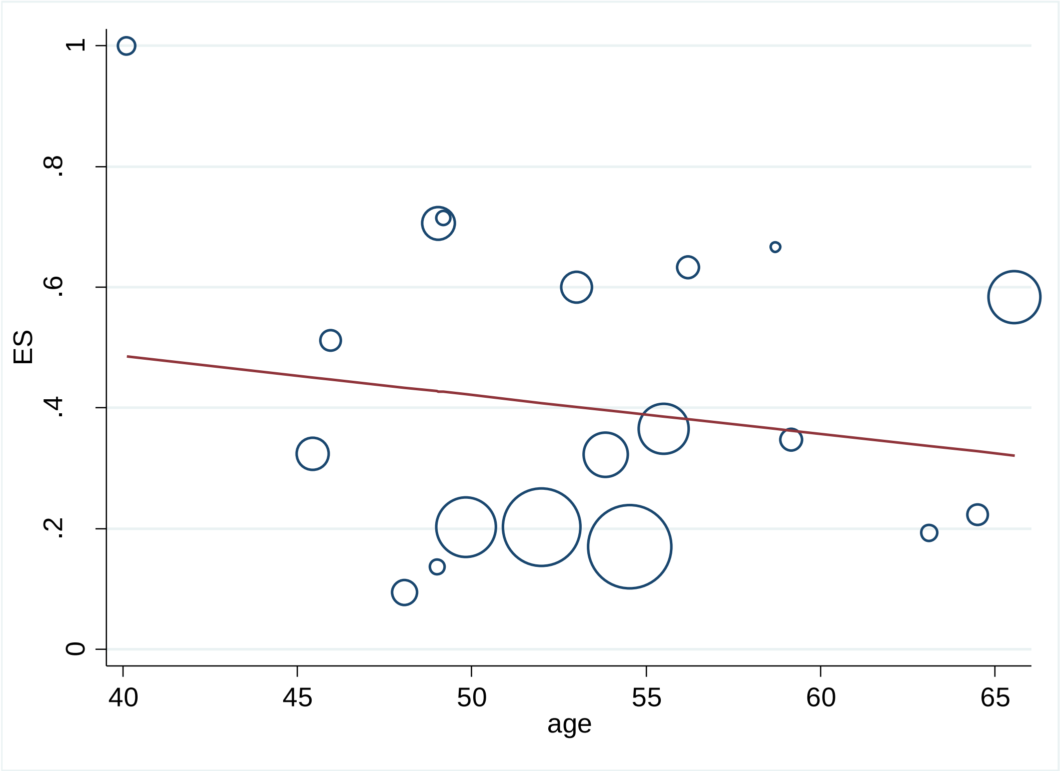

The red line exhibits the fitted regression line together with blue circles representing the estimates from each individual study, sized according to the precision of each estimate (the inverse of its within-study variance).

5.3.3 Effect of tobacco on the prevalence of stress among OLP patients.

Figure S39. Bubble plot graphically representing the potential effect of tobacco (% of smokers) on stress prevalence (derived from meta-regression analysis; see table 2).

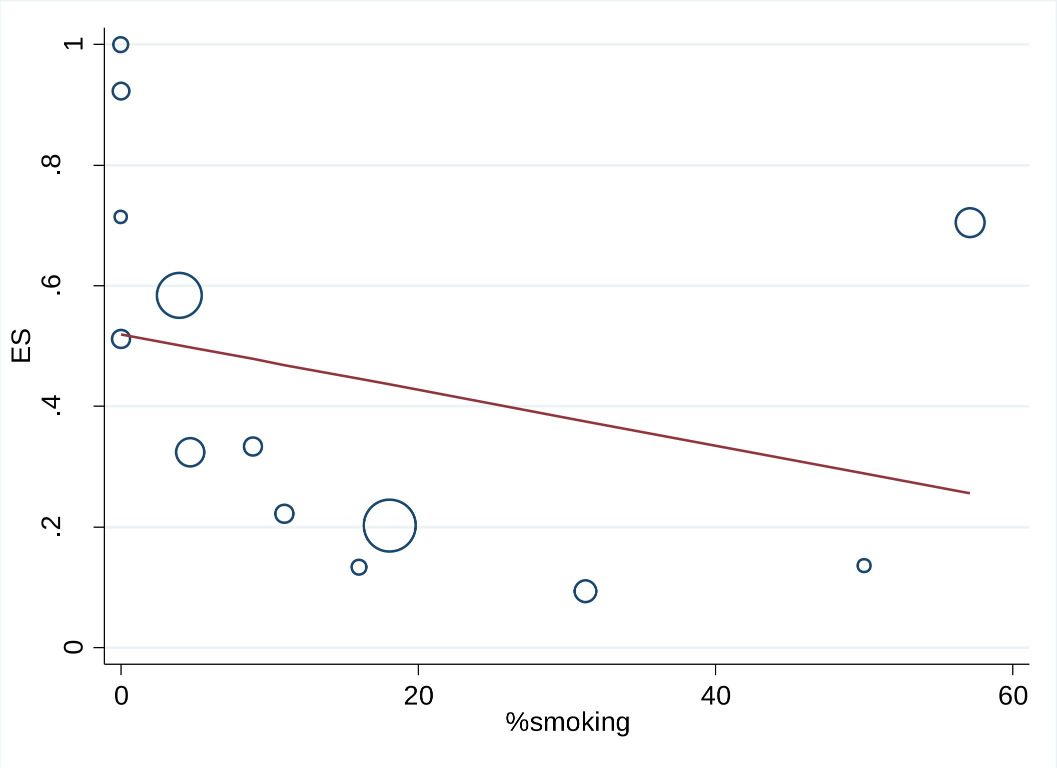

The red line exhibits the fitted regression line together with blue circles representing the estimates from each individual study, sized according to the precision of each estimate (the inverse of its within-study variance).

5.3.4 Effect of alcohol on the prevalence of stress among OLP patients.

Figure S40. Bubble plot graphically representing the potential effect of alcohol (% of drinkers) on stress prevalence (derived from meta-regression analysis; see table 2).

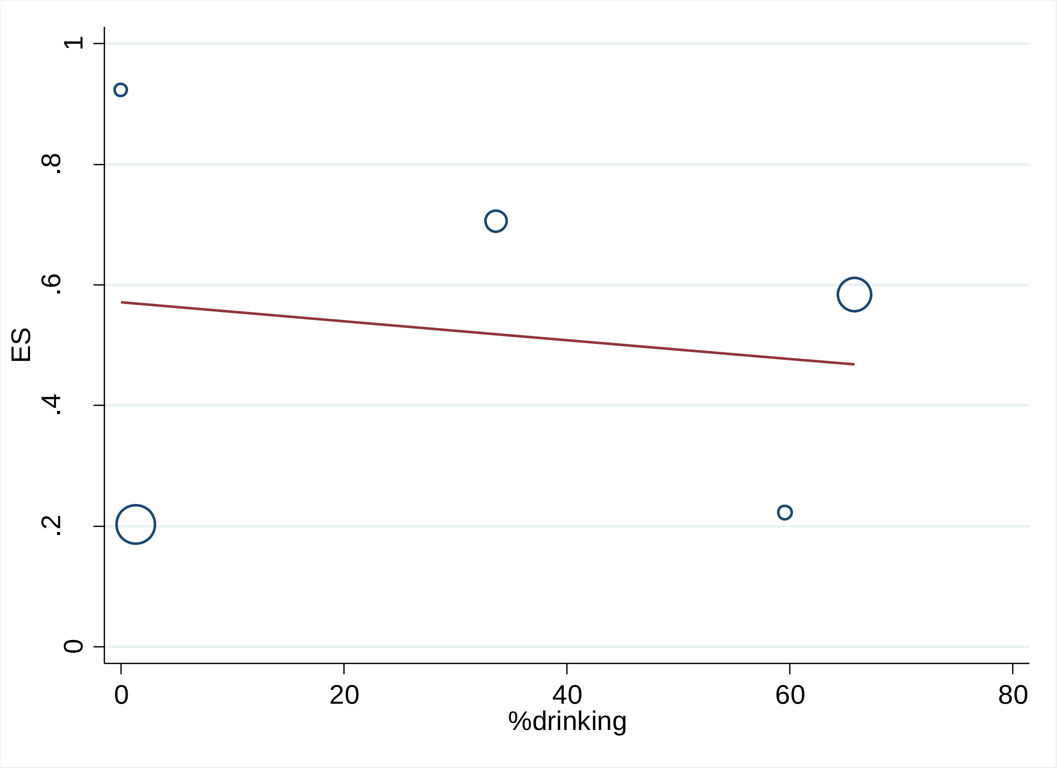

The red line exhibits the fitted regression line together with blue circles representing the estimates from each individual study, sized according to the precision of each estimate (the inverse of its within-study variance).

5.3.5 Effect of OLP type on the prevalence of stress among OLP patients.

Figure S41. Bubble plot graphically representing the potential effect of OLP type (% of patients with red lesions) on stress prevalence (derived from meta-regression analysis; see table 2).

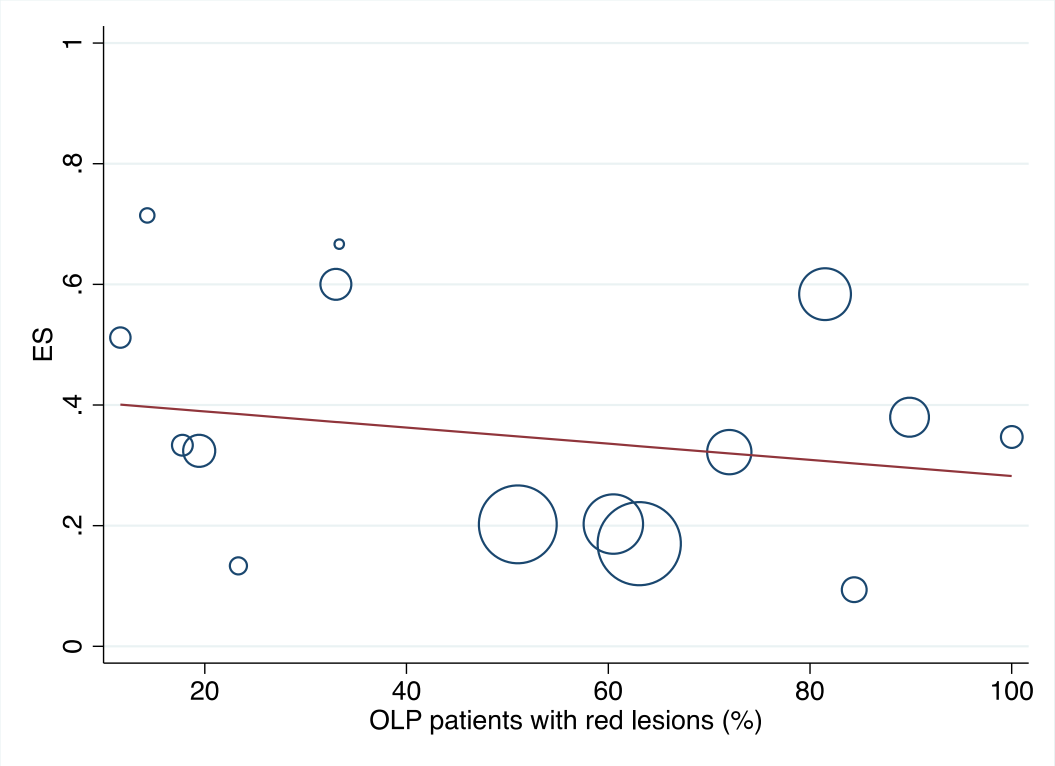

The red line exhibits the fitted regression line together with blue circles representing the estimates from each individual study, sized according to the precision of each estimate (the inverse of its within-study variance).

5.3.6 Effect of publication year on the prevalence of stress among OLP patients.

Figure S42. Bubble plot graphically representing the potential effect of publication year on stress prevalence (derived from meta-regression analysis; see table 2).

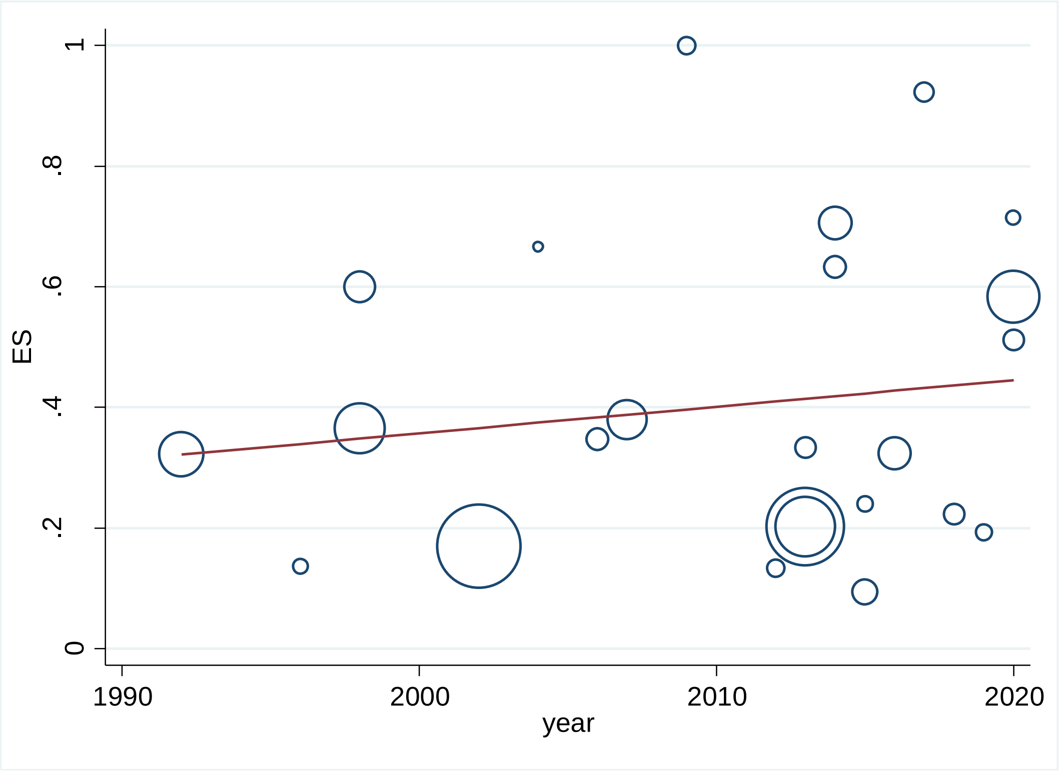

The red line exhibits the fitted regression line together with blue circles representing the estimates from each individual study, sized according to the precision of each estimate (the inverse of its within-study variance).

5.3.7 Effect of human development index on the prevalence of stress among OLP patients.

Figure S43. Bubble plot graphically representing the potential effect of human development index on stress prevalence (derived from meta-regression analysis; see table 2).

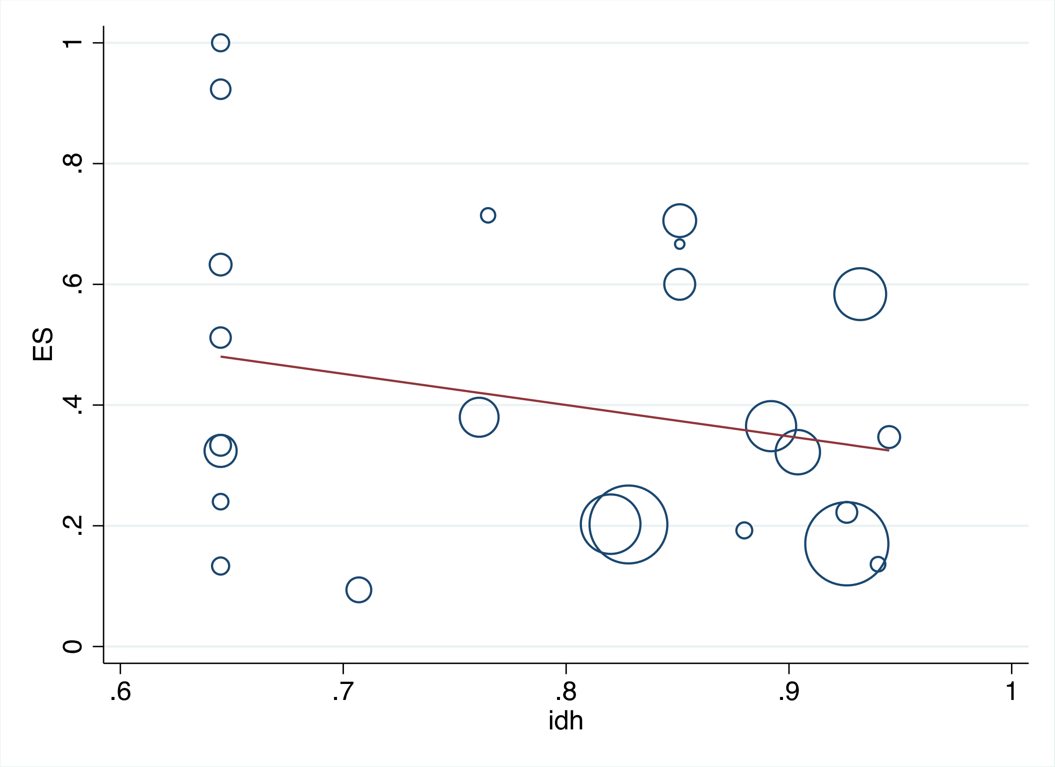

The red line exhibits the fitted regression line together with blue circles representing the estimates from each individual study, sized according to the precision of each estimate (the inverse of its within-study variance).

5.3.8 Effect of risk of bias on the prevalence of stress among OLP patients.

Figure S44. Bubble plot graphically representing the potential effect of risk of bias (expressed as overall score) on stress prevalence (derived from meta-regression analysis; see table 2).

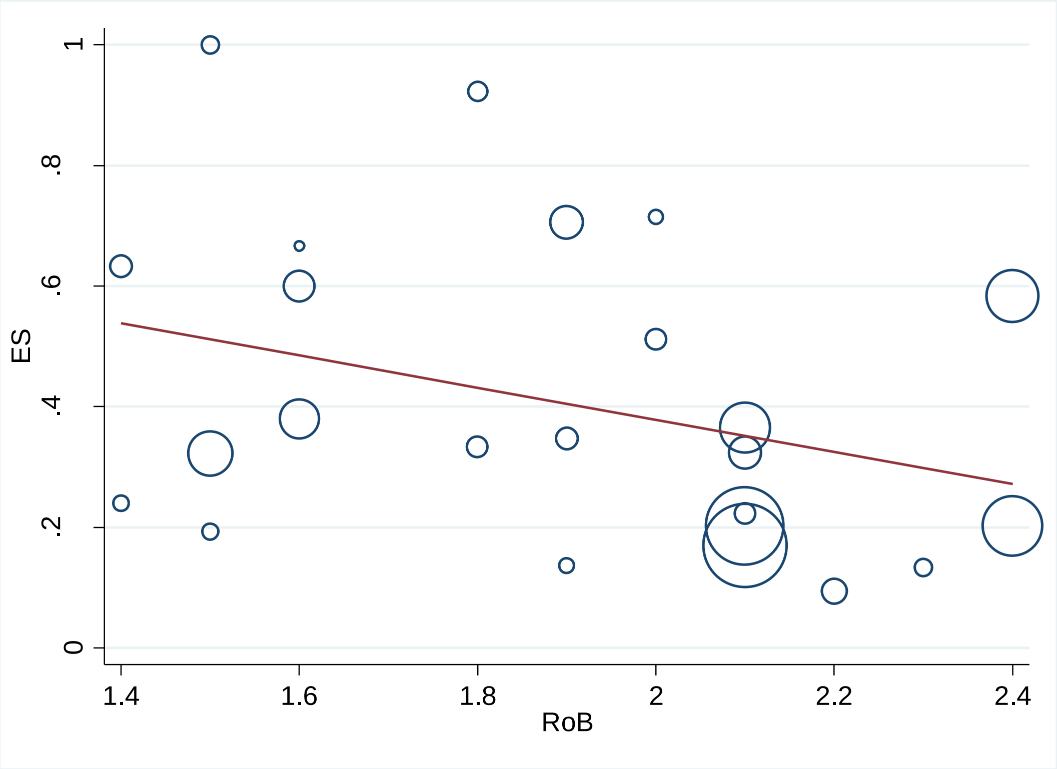

The red line exhibits the fitted regression line together with blue circles representing the estimates from each individual study, sized according to the precision of each estimate (the inverse of its within-study variance).

6. Analysis of small-study effects

6.1 Prevalence of depression among OLP patients

Figure S45. A funnel plot of estimated transformed proportions against their standard errors, graphically representing the analysis of “small-study” effects on the prevalence of depression among OLP patients.

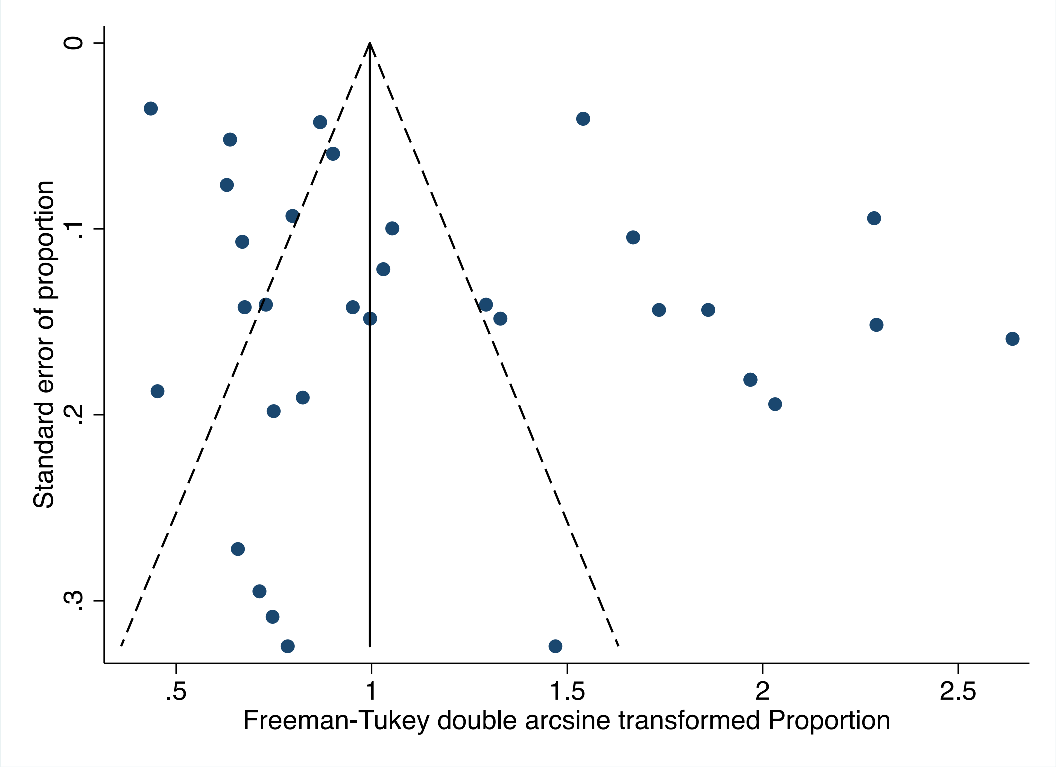

The black vertical line corresponds to the pooled estimated transformed prevalence. The two diagonal intermittent lines represent the pseudo-95% confidence interval. The blue circles represent the published studies.

6.2 Prevalence of anxiety among OLP patients

Figure S46. A funnel plot of estimated transformed proportions against their standard errors, graphically representing the analysis of "small-study" effects on the prevalence of anxiety among OLP patients.

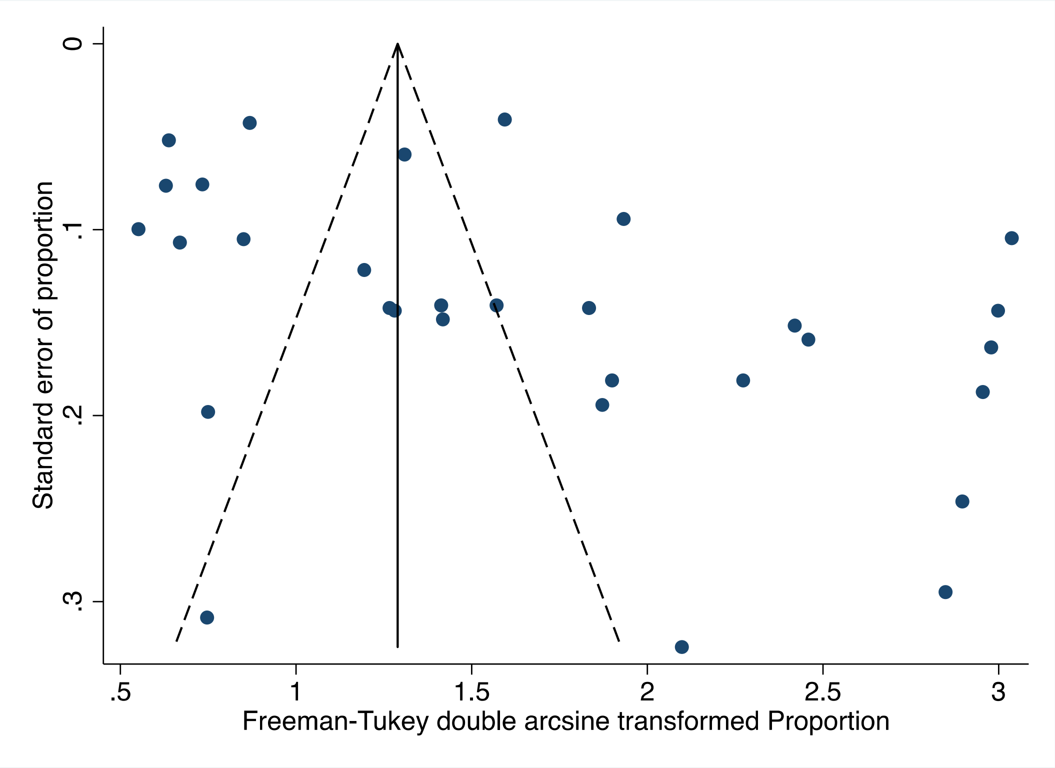

The black vertical line corresponds to the pooled estimated transformed prevalence. The two diagonal intermittent lines represent the pseudo-95% confidence interval. The blue circles represent the published studies.

6.3 Prevalence of stress among OLP patients

Figure S47. A funnel plot of estimated transformed proportions against their standard errors, graphically representing the analysis of "small-study" effects on the prevalence of stress among OLP patients.

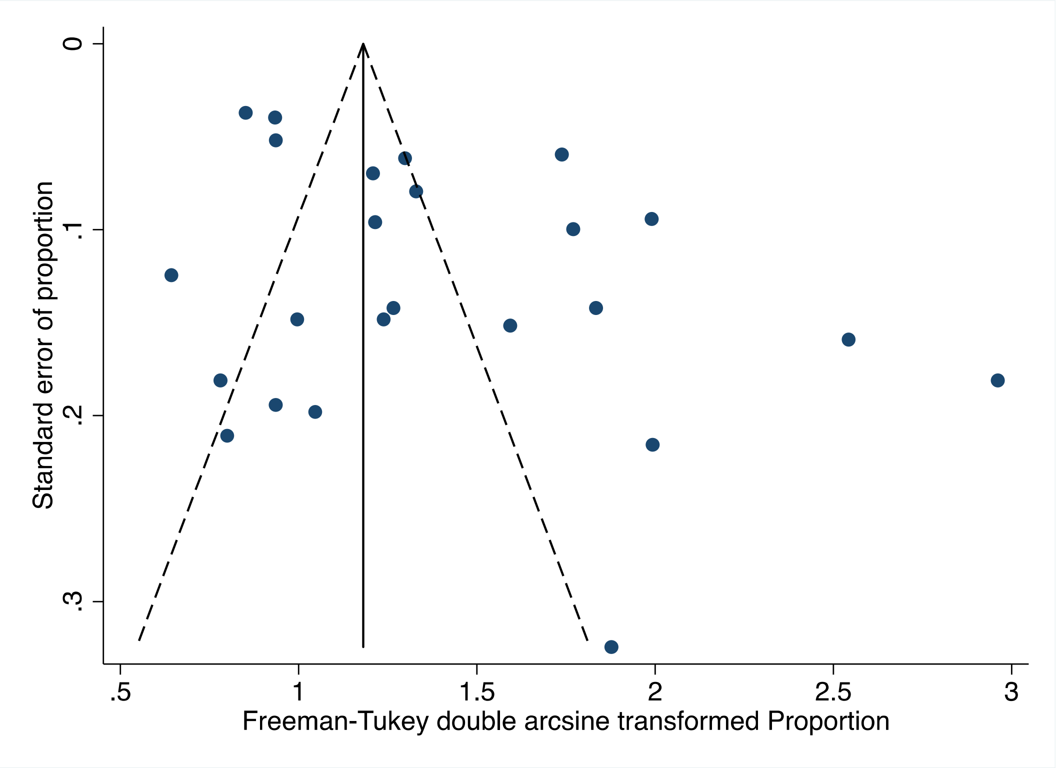

The black vertical line corresponds to the pooled estimated transformed prevalence. The two diagonal intermittent lines represent the pseudo-95% confidence interval. The blue circles represent the published studies.

7. Sensitivity analysis (leave-one-out method).

7.1 Prevalence of depression among OLP patients

Table S3. Sensitivity analysis of pooled studies in the meta-analysis on prevalence of depression among OLP patients.

Sensitivity analysis				
Study omitted		Estimate	95% confidence intervals	
1	McCartan 1995	31.90	22.73	41.79
2	Soto Araya et al. 2004	30.91	21.93	40.62
3	Lundqvist et al. 2006	31.56	22.41	41.44
4	Lundström 2009	32.45	23.75	41.76
5	Shah et al. 2009	30.06	21.20	39.69
6	Bermejo-Fenoll et al. 2010	31.69	21.97	42.24
7	Wu X et al. 2011	31.44	22.28	41.34
8	Park et al. 2012	31.81	22.57	41.79
9	Gümrü 2013	32.08	22.68	42.21
10	Hirota et al. 2013	30.44	21.50	40.13
11	Adamo et al. 2014	31.04	21.95	40.87
12	Gavic et al. 2014	29.46	21.17	38.44
13	Radochova et al. 2014	32.09	22.82	42.08
14	Sandhu et al. 2014	31.98	22.81	41.87
15	Alves et al. 2015	30.36	21.43	40.06
16	Kalkur et al. 2015	31.84	22.71	41.68
17	Choi YS et al. 2016	31.92	22.82	41.72
18	Lauritano et al. 2016	32.01	22.80	41.94
19	Gupta et al. 2017	29.04	20.56	38.28
20	Siponen et al. 2017	31.74	22.61	41.58
21	Di Stasio et al. 2018	31.83	22.75	41.62
22	Edens et al. 2018	31.49	22.35	41.36
23	Yang et al. 2018	30.98	21.91	40.80
24	Manczyk et al. 2019	29.99	21.14	39.61
25	Vilar-Villanueva et al. 2019	30.17	21.30	39.81
26	Wang et al. 2019	31.40	22.20	41.36
27	Adamo et al. 2020	30.62	21.78	40.18
28	Birckel et al. 2020	31.73	22.67	41.50
29	Chaitanya et al. 2020	30.06	21.20	39.69
30	Shaw et al. 2020	29.54	20.87	38.97
31	Vehviläinen et al. 2020	32.28	23.13	42.13
32	Wiriyakijja et al. 2020	31.64	22.21	41.86
33	Zhong et al. 2020	31.78	22.71	41.56
Combined		31.19	22.27	40.82

Sensitivity analysis ("leave-one-out" method) of the meta-analysis results, sequentially omitting one study at a time.

7.2 Magnitude of association between OLP and depression.

Table S4. Sensitivity analysis of pooled studies in the meta-analysis on the magnitude of association between OLP and depression.

Sensitivity analysis				
Study omitted		Estimate	95% confidence intervals	
1	Soto Araya et al. 2004	6.12	2.62	14.28
2	Lundqvist et al. 2006	6.60	2.67	16.29
3	Wu X et al. 2011	6.03	2.59	14.04
4	Hirota et al. 2013	7.25	3.01	17.46
5	Gavic et al. 2014	7.10	2.87	17.55
6	Sandhu et al. 2014	4.61	2.19	9.69
7	Alves et al. 2015	7.56	3.37	16.98
8	Kalkur et al. 2015	7.12	3.05	16.62
9	Gupta et al. 2017	6.17	2.62	14.51
10	Yang et al. 2018	5.85	2.51	13.62
11	Manczyk et al. 2019	6.21	2.61	14.76
12	Vilar-Villanueva et al. 2019	5.81	2.49	13.57
13	Wang et al. 2019	5.52	2.43	12.55
14	Chaitanya et al. 2020	5.36	2.38	12.08
15	Shaw et al. 2020	5.51	2.42	12.53
16	Zhong et al. 2020	6.45	2.80	14.88
Combined		6.15	2.72	13.89

Sensitivity analysis (“leave-one-out” method) of the meta-analysis results, sequentially omitting one study at a time.

7.3 Prevalence of anxiety among OLP patients

Table S5. Sensitivity analysis of pooled studies in the meta-analysis on prevalence of anxiety among OLP patients.

Sensitivity analysis				
Study omitted		Estimate	95% confidence intervals	
1	Colella et al. 1993	52.78	40.07	65.33
2	McCartan 1995	54.93	41.92	67.63
3	Soto Araya et al. 2004	54.11	41.27	66.70
4	Lundqvist et al. 2006	55.46	42.42	68.14
5	Shah et al. 2009	54.37	41.44	67.01
6	Bermejo-Fenoll et al. 2010	56.24	42.70	69.35
7	Bokor-Bratic et al. 2011	56.20	43.15	68.86
8	Gümrü 2013	56.60	43.68	69.09
9	Hirota et al. 2013	52.19	40.39	63.87
10	Adamo et al. 2014	55.21	42.17	67.90
11	Gavic et al. 2014	54.29	41.33	66.98
12	Radochova et al. 2014	56.60	43.64	69.15
13	Sandhu et al. 2014	54.47	41.51	67.15
14	Alves et al. 2015	52.40	40.03	64.63
15	Barbosa et al. 2015	52.48	39.98	64.83
16	Kalkur et al. 2015	56.27	43.34	68.81
17	Lauritano et al. 2016	56.51	43.53	69.07
18	Pippi et al. 2016	55.60	42.52	68.31
19	Gupta et al. 2017	53.39	40.63	65.93
20	Adamo et al. 2018	52.57	39.97	65.01
21	Di Stasio et al. 2018	52.95	40.20	65.52
22	Yang et al. 2018	55.19	42.17	67.88
23	Manczyk et al. 2019	54.42	41.49	67.06
24	Vilar-Villanueva et al. 2019	55.43	42.40	68.11
25	Wang et al. 2019	56.72	43.81	69.20
26	Adamo et al. 2020	54.98	41.12	68.47
27	Chaitanya et al. 2020	53.73	40.89	66.33
28	Liao et al. 2020	56.43	43.36	69.08
29	Shaw et al. 2020	53.45	40.69	65.99
30	Wiriyakijja et al. 2020	55.46	41.91	68.63
31	Zhong et al. 2020	56.11	43.23	68.60
Combined		54.76	42.06	67.17

Sensitivity analysis ("leave-one-out" method) of the meta-analysis results, sequentially omitting one study at a time.

7.4 Magnitude of association between OLP and anxiety.

Table S6. Sensitivity analysis of pooled studies in the meta-analysis on magnitude of association between OLP and anxiety.

Sensitivity analysis				
Study omitted		Estimate	95% confidence intervals	
1	Colella et al. 1993	3.46	2.05	5.85
2	Soto Araya et al. 2004	3.48	2.04	5.95
3	Lundqvist et al. 2006	3.58	2.01	6.39
4	Bokor-Bratic et al. 2011	3.97	2.36	6.65
5	Hirota et al. 2013	3.52	2.08	5.96
6	Gavic et al. 2014	3.91	2.29	6.66
7	Sandhu et al. 2014	3.43	1.98	5.97
8	Alves et al. 2015	3.51	2.10	5.85
9	Kalkur et al. 2015	3.76	2.21	6.38
10	Gupta et al. 2017	3.64	2.11	6.29
11	Yang et al. 2018	3.18	1.91	5.27
12	Manczyk et al. 2019	3.58	2.06	6.21
13	Vilar-Villanueva et al. 2019	3.63	2.08	6.34
14	Wang et al. 2019	3.37	2.01	5.66
15	Chaitanya et al. 2020	3.43	1.99	5.90
16	Shaw et al. 2020	2.74	1.83	4.10
17	Zhong et al. 2020	3.57	2.11	6.03
Combined		3.51	2.10	5.85

Sensitivity analysis (“leave-one-out” method) of the meta-analysis results, sequentially omitting one study at a time.

7.5 Prevalence of stress among OLP patients

Table S7. Sensitivity analysis of pooled studies in the meta-analysis on prevalence of stress among OLP patients.

Sensitivity analysis				
Study omitted		Estimate	95% confidence intervals	
1	Bagán-Sebastián et al. 1992	41.58	32.11	51.35
2	Pedersen 1996	42.28	33.12	51.70
3	Cekic-Arambasin et al. 1998	40.22	31.24	49.52
4	Mignona et al. 1998	41.37	31.86	51.20
5	Eisen 2002	42.43	33.06	52.07
6	Soto Araya et al. 2004	40.40	31.43	49.70
7	Lundqvist et al. 2006	41.40	32.19	50.90
8	Sun et al. 2007	41.28	31.92	50.95
9	Shah et al. 2009	37.85	29.59	46.46
10	Jayavelu et al. 2012	42.38	33.20	51.81
11	Gümrü 2013	42.24	32.69	52.07
12	Kumar et al. 2013	41.45	32.25	50.95
13	Tovaru et al. 2013	42.26	32.55	52.27
14	Gavic et al. 2014	39.66	31.00	48.64
15	Sandhu et al. 2014	40.13	31.14	49.46
16	Kalkur et al. 2015	41.81	32.65	51.26
17	Mostafa et al. 2015	42.79	33.63	52.20
18	Mankapure et al. 2016	41.54	32.22	51.15
19	Gupta et al. 2017	38.61	30.11	47.46
20	Edens et al. 2018	41.98	32.76	51.47
21	Manczyk et al. 2019	42.04	32.87	51.48
22	Pires et al. 2020	39.96	31.00	49.25
23	Shaw et al. 2020	40.68	31.57	50.10
24	Wiriyakijja et al. 2020	40.23	31.46	49.31
Combined		41.10	32.18	50.32

Sensitivity analysis (“leave-one-out” method) of the meta-analysis results, sequentially omitting one study at a time.

7.6 Magnitude of association between OLP and stress.

Table S8. Sensitivity analysis of pooled studies in the meta-analysis on the magnitude of association between OLP and stress.

Sensitivity analysis				
Study omitted		Estimate	95% confidence intervals	
1	Soto Araya et al. 2004	3.71	1.37	10.06
2	Lundqvist et al. 2006	3.69	1.25	10.86
3	Gavic et al. 2014	4.49	2.55	7.91
4	Kalkur et al. 2015	4.23	1.52	11.76
5	Gupta et al. 2017	3.75	1.35	10.38
6	Manczyk et al. 2019	3.33	1.31	8.45
7	Pires et al. 2020	3.38	1.26	9.09
8	Shaw et al. 2020	2.84	1.22	6.60
Combined		3.34	1.48	8.94

Sensitivity analysis (“leave-one-out” method) of the meta-analysis results, sequentially omitting one study at a time.

8. Validation of methodological quality.

8.1 List S1. AMSTAR2 checklist.

AMSTAR 2: a critical appraisal tool for systematic reviews that include randomised or non-randomised studies of healthcare interventions, or both

1. Did the research questions and inclusion criteria for the review include the components of PICO?

For Yes:
- ☑ Population
- ☑ Intervention
- ☑ Comparator group
- ☑ Outcome

Optional (recommended)
- ☑ Timeframe for follow-up

- ☑ Yes
- ☐ No

2. Did the report of the review contain an explicit statement that the review methods were established prior to the conduct of the review and did the report justify any significant deviations from the protocol?

For Partial Yes:
The authors state that they had a written protocol or guide that included ALL the following:
- ☑ review question(s)
- ☑ a search strategy
- ☑ inclusion/exclusion criteria
- ☑ a risk of bias assessment

For Yes:
As for partial yes, plus the protocol should be registered and should also have specified:
- ☑ a meta-analysis/synthesis plan, if appropriate, *and*
- ☑ a plan for investigating causes of heterogeneity
- ☑ justification for any deviations from the protocol

- ☑ Yes
- ☐ Partial Yes
- ☐ No

3. Did the review authors explain their selection of the study designs for inclusion in the review?

For Yes, the review should satisfy ONE of the following:
- ☐ *Explanation for* including only RCTs
- ☑ OR *Explanation for* including only NRSI
- ☐ OR *Explanation for* including both RCTs and NRSI

- ☑ Yes
- ☐ No

4. Did the review authors use a comprehensive literature search strategy?

For Partial Yes (all the following):
- ☑ searched at least 2 databases (relevant to research question)
- ☑ provided key word and/or search strategy
- ☑ justified publication restrictions (e.g. language)

For Yes, should also have (all the following):
- ☑ searched the reference lists / bibliographies of included studies
- ☐ searched trial/study registries — Not relevant for observational studies
- ☑ included/consulted content experts in the field
- ☐ where relevant, searched for grey literature — Considered not relevant and very controversial.
- ☑ conducted search within 24 months of completion of the review

- ☑ Yes
- ☐ Partial Yes
- ☐ No

5. Did the review authors perform study selection in duplicate?

For Yes, either ONE of the following:
- ☑ at least two reviewers independently agreed on selection of eligible studies and achieved consensus on which studies to include
- ☐ OR two reviewers selected a sample of eligible studies and achieved good agreement (at least 80 percent), with the remainder selected by one reviewer.

- ☑ Yes
- ☐ No

AMSTAR 2: a critical appraisal tool for systematic reviews that include randomised or non-randomised studies of healthcare interventions, or both

6. Did the review authors perform data extraction in duplicate?

For Yes, either ONE of the following:	
☑ at least two reviewers achieved consensus on which data to extract from included studies<br>☐ OR two reviewers extracted data from a sample of eligible studies <u>and</u> achieved good agreement (at least 80 percent), with the remainder extracted by one reviewer.	☑ Yes<br>☐ No

7. Did the review authors provide a list of excluded studies and justify the exclusions?

For Partial Yes:	For Yes, must also have:	
☑ provided a list of all potentially relevant studies that were read in full-text form but excluded from the review	☑ Justified the exclusion from the review of each potentially relevant study	☑ Yes<br>☐ Partial Yes<br>☐ No

8. Did the review authors describe the included studies in adequate detail?

For Partial Yes (ALL the following):	For Yes, should also have ALL the following:	
☑ described populations<br>☑ described interventions<br>☑ described comparators<br>☑ described outcomes<br>☑ described research designs	☑ described population in detail<br>☑ described intervention in detail (including doses where relevant)<br>☑ described comparator in detail (including doses where relevant)<br>☑ described study's setting<br>☑ timeframe for follow-up	☑ Yes<br>☐ Partial Yes<br>☐ No

9. Did the review authors use a satisfactory technique for assessing the risk of bias (RoB) in individual studies that were included in the review?

**RCTs**<br>For Partial Yes, must have assessed RoB from	For Yes, must also have assessed RoB from:	
☐ unconcealed allocation, *and*<br>☐ lack of blinding of patients and assessors when assessing outcomes (unnecessary for objective outcomes such as all-cause mortality)	☐ allocation sequence that was not truly random, *and*<br>☐ selection of the reported result from among multiple measurements or analyses of a specified outcome	☐ Yes<br>☐ Partial Yes<br>☐ No<br>☑ Includes only NRSI
**NRSI**<br>For Partial Yes, must have assessed RoB:	For Yes, must also have assessed RoB:	
☑ from confounding, *and*<br>☑ from selection bias	☑ methods used to ascertain exposures and outcomes, *and*<br>☑ selection of the reported result from among multiple measurements or analyses of a specified outcome	☑ Yes<br>☐ Partial Yes<br>☐ No<br>☐ Includes only RCTs

10. Did the review authors report on the sources of funding for the studies included in the review?

For Yes	
☐ Must have reported on the sources of funding for individual studies included in the review. Note: Reporting that the reviewers looked for this information but it was not reported by study authors also qualifies	☐ Yes<br>☑ No

AMSTAR 2: a critical appraisal tool for systematic reviews that include randomised or non-randomised studies of healthcare interventions, or both

11. If meta-analysis was performed did the review authors use appropriate methods for statistical combination of results?

RCTs
For Yes:

- ☐ The authors justified combining the data in a meta-analysis
  - ☐ AND they used an appropriate weighted technique to combine study results and adjusted for heterogeneity if present.
  - ☐ AND investigated the causes of any heterogeneity

☐ Yes
☐ No
☐ No meta-analysis conducted

For NRSI
For Yes:

- ☑ The authors justified combining the data in a meta-analysis
  - ☑ AND they used an appropriate weighted technique to combine study results, adjusting for heterogeneity if present
  - ☑ AND they statistically combined effect estimates from NRSI that were adjusted for confounding, rather than combining raw data, or justified combining raw data when adjusted effect estimates were not available
  - ☑ AND they reported separate summary estimates for RCTs and NRSI separately when both were included in the review

☑ Yes
☐ No
☐ No meta-analysis conducted

12. If meta-analysis was performed, did the review authors assess the potential impact of RoB in individual studies on the results of the meta-analysis or other evidence synthesis?

For Yes:

- ☐ included only low risk of bias RCTs
- ☑ OR, if the pooled estimate was based on RCTs and/or NRSI at variable RoB, the authors performed analyses to investigate possible impact of RoB on summary estimates of effect.

☑ Yes
☐ No
☐ No meta-analysis conducted

13. Did the review authors account for RoB in individual studies when interpreting/ discussing the results of the review?

For Yes:

- ☐ included only low risk of bias RCTs
- ☑ OR, if RCTs with moderate or high RoB, or NRSI were included the review provided a discussion of the likely impact of RoB on the results

☑ Yes
☐ No

14. Did the review authors provide a satisfactory explanation for, and discussion of, any heterogeneity observed in the results of the review?

For Yes:

- ☐ There was no significant heterogeneity in the results
- ☑ OR if heterogeneity was present the authors performed an investigation of sources of any heterogeneity in the results and discussed the impact of this on the results of the review

☑ Yes
☐ No

15. If they performed quantitative synthesis did the review authors carry out an adequate investigation of publication bias (small study bias) and discuss its likely impact on the results of the review?

For Yes:

- ☑ performed graphical or statistical tests for publication bias and discussed the likelihood and magnitude of impact of publication bias

☑ Yes
☐ No
☐ No meta-analysis conducted

AMSTAR 2: a critical appraisal tool for systematic reviews that include randomised or non-randomised studies of healthcare interventions, or both

16. Did the review authors report any potential sources of conflict of interest, including any funding they received for conducting the review?

For Yes:	
☑ The authors reported no competing interests OR	☑ Yes
☐ The authors described their funding sources and how they managed potential conflicts of interest	☐ No

To cite this tool: Shea BJ, Reeves BC, Wells G, Thuku M, Hamel C, Moran J, Moher D, Tugwell P, Welch V, Kristjansson E, Henry DA. AMSTAR 2: a critical appraisal tool for systematic reviews that include randomised or non-randomised studies of healthcare interventions, or both. BMJ. 2017 Sep 21;358:j4008.

8.2 Table S9. AMSTAR2 scoring system

Tool	Study design	Items																Overall rating	Score
		1	2	3	4	5	6	7	8	9	10	11	12	13	14	15	16		
AMSTAR2	Systematic review and meta-analysis																	HIGH	15

Explanation:

The methodological quality of this systematic review followed the *Assesing the Methodological Quality of Systematic Reviews-2* (AMSTAR2) recommendations and was validated using this tool. AMSTAR2 was designed to develop, evaluate and validate high quality systematic reviews through 16 items. An overall rating is obtained based on weaknesses(*) in the following critical and non-critical items (the checklist was also included in the precedent appendix page):

1. Did the research questions and inclusion criteria for the review include the components of PICO?
2. Did the report of the review contain an explicit statement that the review methods were established prior to the conduct of their review, and did the report justify any significant deviations from the protocol?*
3. Did the review authors explain their selection of the study designs for inclusion in the review?
4. Did the review authors use a comprehensive literature search strategy?*
5. Did the review authors perform study selection in duplicate?
6. Did the review authors perform data extraction in duplicate?
7. Did the review authors provide a list of excluded studies and justify the exclusions?*
8. Did the review authors describe the included studies in adequate detail?
9. Did the review authors use a satisfactory technique for assessing the risk of bias (RoB) in individual studies that were included in the review?*
10. Did the review authors report on the sources of funding for the studies included in the review?
11. If meta-analysis was performed, did the review authors use appropriate methods for statistical combination of results?*
12. If meta-analysis was performed, did the review authors assess the potential impact of RoB in individual studies on the results of the metaanalysis or other evidence synthesis?
13. Did the review authors account for RoB in individual studies when interpreting/discussing the results of the review?*
14. Did the review authors provide a satisfactory explanation for, and discussion of, any heterogeneity observed in the results of the review?
15. If they performed quantitative synthesis, did the review authors carry out an adequate investigation of publication bias (small study bias) and discuss its likely impact on the results of the review?*
16. Did the review authors report any potential sources of conflict of interest, including any funding they received for conducting the review?

High overall rating: No or one non-critical weakness. The review provides an accurate and comprehensive summary of the results of the available studies that address the question of interest.

9. Depression, Anxiety and Stress Scale (DASS-21 questionnaire).

Depression, Anxiety and Stress Scale (DASS21)

For each statement below, please circle the number in the column that best represents how you have been feeling in the last week.

Statement	Did not apply to me at all	Applied to me to some degree or some of the time	Applied to me a considerable degree or a good part of the time	Applied to me very much or most of the time
1. I found it hard to wind down	0	1	2	3
2. I was aware of dryness of my mouth	0	1	2	3
3. I couldn't seem to experience any positive feeling at all	0	1	2	3
4. I experienced breathing difficulty (eg, excessively rapid breathing, breathlessness in the absence of physical exertion)	0	1	2	3
5. I found it difficult to work up the initiative to do things	0	1	2	3
6. I tended to over-react to situations	0	1	2	3
7. I experienced trembling (eg, in the hands)	0	1	2	3
8. I felt that I was using a lot of nervous energy	0	1	2	3
9. I was worried about situations in which I might panic and make a fool of myself	0	1	2	3
10. I felt that I had nothing to look forward to	0	1	2	3
11. I found myself getting agitated	0	1	2	3
12. I found it difficult to relax	0	1	2	3
13. I felt down-hearted and blue	0	1	2	3
14. I was intolerant of anything that kept me from getting on with what I was doing	0	1	2	3
15. I felt I was close to panic	0	1	2	3
16. I was unable to become enthusiastic about anything.	0	1	2	3
17. I felt I wasn't worth much as a person	0	1	2	3
18. I felt that I was rather touchy	0	1	2	3
19. I was aware of the action of my heart in the absence of physical exertion (eg, sense of heart rate increase, heart missing a beat)	0	1	2	3
20. I felt scared without any good reason.	0	1	2	3
21. I felt that life was meaningless	0	1	2	3

Lovibond, S.H. & Lovibond, P.F. (1995). Manual for the Depression Anxiety Stress Scales. (2nd. Ed.) Sydney: Psychology Foundation

DASS21 SCORING

1) For questions numbered 3, 5, 10, 13, 16, 17, 21 add up the numbers circled then multiply that number by 2 and enter it here: ________
2) For questions numbered 2, 4, 7, 9, 15, 19, 20 add up the numbers circled then multiply that number by 2 and enter it here: ________
3) For questions numbered 1, 6, 8, 11, 12, 14, 18 add up the numbers circled then multiply that number by 2 and enter it here: ________

Refer to the chart below and for each numbered question above, refer to the same number in the table below to determine how mild or serious each condition may be.

Rating	Depression #1	Anxiety #2	Stress #3
Normal	0-9	0-7	0-14
Mild	10-13	8-9	15-18
Moderate	14-20	10-14	19-25
Severe	21-27	15-19	26-33
Extremely Severe	28+	20+	37+

Provided to you by Depression-Test.net for educational purposes only. If there is an indication that you might be depressed, please check out the site for additional information, tools and support.

If there is an indication that It might be serious then please see the help of a mental health professional.

10. List of included studies (n=51). List S2.

Adamo, D., Calabria, E., Coppola, N., Lo Muzio, L., Giuliani, M., Bizzoca, M. E., Azzi, L., Croveri, F., Colella, G., Boschetti, C. E., Montebugnoli, L., Gissi, D., Gabriele, M., Nisi, M., Sardella, A., Lodi, G., Varoni, E. M., Giudice, A., Antonelli, A., Mignogna, M. D. (2021). Psychological profile and unexpected pain in oral lichen planus: A case-control multicenter SIPMO study(a). Oral Diseases. [In Press]

Adamo, D., Mignogna, M. D., Pecoraro, G., Aria, M., & Fortuna, G. (2018). Management of reticular oral lichen planus patients with burning mouth syndrome-like oral symptoms: a pilot study. The Journal of Dermatological Treatment, 29(6), 623–629.

Adamo, D., Ruoppo, E., Leuci, S., Aria, M., Amato, M., & Mignogna, M. D. (2015). Sleep disturbances, anxiety and depression in patients with oral lichen planus: A case-control study. Journal of the European Academy of Dermatology and Venereology, 29(2), 291–297.

Bagan-Sebastian, J. V, Milian-Masanet, M. A., Penarrocha-Diago, M., Jimenez (1992). A clinical study of 205 patients with oral lichen planus. Journal of Oral and Maxillofacial Surgery, 50(2), 116–118.

Barbosa, N. G., Silveira, É. J., Lima, E. N., Oliveira, P. T., Soares, M. S., & de Medeiros, A. M. (2015). Factors associated with clinical characteristics and symptoms in a case series of oral lichen planus. International Journal of Dermatology, 54(1), e1--e6.

Bermejo-Fenoll, A., Sanchez-Siles, M., Lopez-Jornet, P., Camacho-Alonso, F., & Salazar-Sanchez, N. (2010). A retrospective clinicopathological study of 550 patients with oral lichen planus in south-eastern Spain. Journal of Oral Pathology and Medicine, 39(6), 491–496.

Birckel, E., Lipsker, D., & Cribier, B. (2020). [Efficacy of photopheresis in the treatment of erosive lichen planus: A retrospective study]. Annales de dermatologie et de venereologie, 147(2), 86–92.

Bokor-Bratic, M., Cankovic, M., & Dragnic, N. (2013). Unstimulated whole salivary flow rate and anxiolytics intake are independently associated with oral Candida infection in patients with oral lichen planus. European Journal of Oral Sciences, 121(5), 427–433.

Cekic-Arambasin, A., Biocina-Lukenda, D., & Lazic-Segula, B. (1998). Characteristics of oral lichen in the Croatian population. Collegium Antropologicum, 22, 73–81.

Chaitanya, N. C., Reshmapriyanka, D., Pallavi, K., Ameer, S., Appala, A., Chowdhary, A., Prabhath, T., Ratna, M. P., Sowmya, B. S., Vaishnavi, C., & Bontala, P. (2020). Serological and psychological assessment of patients with oral lichen planus using serum cortisol levels and hads questionnaire-a case control study. Journal of Population Therapeutics and Clinical Pharmacology = Journal de La Therapeutique Des Populations et de La Pharmacologie Clinique, 27(2), e19–e27.

Choi, Y. S. Y., Kim, Y., Yoon, H.-J., Baek, K. J., Alam, J. & Park, H. K. (2016). The presence of bacteria within tissue provides insights into the pathogenesis of oral lichen planus. Scientific Reports, 6, 29186.

Colella, G., Gritti, P., De Luca, F., & de Vito, M. (1993). The psychopathological aspects of oral lichen planus (OLP). Minerva Stomatologica, 42(6), 265–270.

Edens, M. H., Carpenter, M. D., Napenas, J. J., Brennan (2018). Impact of salivary hypofunction on incidence of orofungal infections with use of topical steroids for management of oral lichen planus and xerostomia. Oral Surgery, Oral Medicine, Oral Pathology and Oral Radiology, 126(6), 501–505.

Eisen, D. (2002). The clinical features, malignant potential, and systemic associations of oral lichen planus: A study of 723 patients. Journal of the American Academy of Dermatology, 46(2), 207–214.

Gavic, L., Cigic, L., Biocina Lukenda, D., Gruden, V., Gruden Pokupec (2014). The role of anxiety, depression, and psychological stress on the clinical status of recurrent aphthous stomatitis and oral lichen planus. Journal of Oral Pathology and Medicine, 43(6), 410–417.

Gümrü, B., Guemrue, B.(2013). A retrospective study of 370 patients with oral lichen planus in Turkey. Medicina Oral, Patologia Oral y Cirugia Bucal, 18(3), e427--e432.

Gupta, A., Mohan, R. P. S., Gupta, S., Malik, S. S., Goel, S., Kamarthi, N (2017). Roles of serum uric acid, prolactin levels, and psychosocial factors in oral lichen planus. Journal of Oral Science, 59(1), 139–146.

Hirota, S. K., Moreno, R. A., Dos Santos, C. H. R., Seo, J., & Migliari, D. A. (2013). Psychological profile (anxiety and depression) in patients with oral lichen planus: A controlled study. Minerva Stomatologica, 62(3), 51–56.

Jayavelu, P., & Sambandan, T. (2012). Prevalence of hepatitis C and hepatitis B virus infection(s) in patients with oral lichen planus. Journal of Pharmacy & Bioallied Sciences, 4, S397--405.

Kalkur, C., Sattur, A. P., & Guttal, K. S. (2015). Role of Depression, Anxiety and Stress in Patients with Oral Lichen Planus: A Pilot Study. Indian Journal of Dermatology, 60(5), 445–449.

Kumar, M. K. P., Jois, H. S., Hallikerimath, S., Kale, A. D. (2013). Oral lichen planus as an extra-hepatic manifestation of viral hepatitis-evaluation in Indian subpopulation. Journal of Clinical and Diagnostic Research, 7(9), 2068–2069.

Lauritano, D., Arrica, M., Lucchese, A., Valente, M., Pannone, G., Lajolo, C., Ninivaggi, R., Petruzzi, M (2016). Oral lichen planus clinical characteristics in Italian patients: a retrospective analysis. Head & Face Medicine, 12, 18.

Liao, H., Luo, Y., Long, L., Peng, J., Qiu, X., Yuan, P., Xu, H., & Jiang, L. (2021). Anxiety and oral lichen planus. Oral Diseases, 27(3), 506–514.

Lundqvist, E. N., Wahlin, Y. B., Bergdahl, M., & Bergdahl, J. (2006). Psychological health in patients with genital and oral erosive lichen planus. Journal of the European Academy of Dermatology and Venereology, 20(6), 661–666.

Lundström, I.M. (2009). Orofacial and general disorders in oral medicine patients. Oral and medical history. Swedish Dental Journal, 33(1), 27–39.

Manczyk, B., Gołda, J., Biniak, A., Reszelewska, K., Mazur, B., Zając, K., Bińczak, P., Chomyszyn-Gajewska, M., & Oruba, Z. (2019). Evaluation of depression, anxiety and stress levels in patients with oral lichen planus. Journal of Oral Science, 61(3), 391–397.

Mankapure, P. K., Humbe, J. G., Mandale, M. S., & Bhavthankar, J. D. (2016). Clinical profile of 108 cases of oral lichen planus. Journal of Oral Science, 58(1), 43–47.

McCartan, B. E. (1995). Psychological factors associated with oral lichen planus. Journal of Oral Pathology & Medicine: Official Publication of the International Association of Oral Pathologists and the American Academy of Oral Pathology, 24(6), 273–275.

Mignogna, M. D., Lo Muzio, L., Favia, G., Mignogna, R. E., Carbone, R., & Bucci, E. (1998). Oral lichen planus and HCV infection: A clinical evaluation of 263 cases. International Journal of Dermatology, 37(8), 575–578.

Mostafa, B., & Ahmed, E. (2015). Prevalence of oral lichen planus among a sample of the Egyptian population. Journal of Clinical and Experimental Dentistry, 7(1), e7--e12.

Oliveira Alves, M. G., do Carmo Carvalho, B. F., Balducci, I., Guimaraes Cabral, L. A., Nicodemo, D., Almeida, J. D. (2015). Emotional assessment of patients with oral lichen planus. International Journal of Dermatology, 54(1), 29–32.

Park, H.-K., Hurwitz, S., & Woo, S.-B. (2012). Oral lichen planus: REU scoring system correlates with pain. Oral Surgery, Oral Medicine, Oral Pathology and Oral Radiology, 114(1), 75–82.

Pedersen, A. (1996). Abnormal EBV immune status in oral lichen planus. Oral Diseases, 2(2), 125–128.

Pippi, R., Romeo, U., Santoro, M., Del Vecchio, A., Scully, C., & Petti, S. (2016). Psychological disorders and oral lichen planus: Matched case-control study and literature review. Oral Diseases, 22(3), 226–234.

Pires, A., Simoura, J., Cerqueira, J., Lima-Arsati, Y., Arsati, F., Dos Santos, J. N., & Freitas, V. S. (2020). Relationship of psychological factors with salivary flow rate and cortisol levels in individuals with oral lichen planus: A case-control study. Oral Surgery, Oral Medicine, Oral Pathology and Oral Radiology, 130(6), 675–680.

Radochová, V., Drízhal, I., Slezák, R. (2014). A retrospective study of 171 patients with oral lichen planus in the East Bohemia - Czech Republic - Single center experience. Journal of Clinical and Experimental Dentistry, 6(5), e556--e561.

Sandhu, S. V, Sandhu, J. S., Bansal, H., & Dua, V. (2014). Oral lichen planus and stress: An appraisal. Contemporary Clinical Dentistry, 5(3), 352–356.

Shah, B., Ashok, L., & Sujatha, G. P. (2009). Evaluation of salivary cortisol and psychological factors in patients with oral lichen planus. Indian Journal of Dental Research, 20(3), 288–292.

Shaw, H., Konidena, A., Malhotra, A., Yumnam, N., Farooq, F., & Bansal, V. (2020). Psychological status and uric acid levels in oral lichen planus patients - A case- control study. Indian Journal of Dental Research: Official Publication of Indian Society for Dental Research, 31(3), 368–375.

Siponen, M., Huuskonen, L., Kallio-Pulkkinen, S., Nieminen, P., Salo, T. (2017). Topical tacrolimus, triamcinolone acetonide, and placebo in oral lichen planus: a pilot randomized controlled trial. Oral Diseases, 23(5), 660–668.

Soto Araya, M., Rojas Alcayaga, G., Esguep, A. (2004). Association between psychological disorders and the presence of Oral lichen planus, Burning mouth syndrome and Recurrent aphthous Stomatitis. Medicina Oral, 9(1), 1–7.

Sun, A., Chia, J. S., Wang, J. T., & Chiang, C. P. (2007). Levamisole can reduce the high serum tumour necrosis factor-α level to a normal level in patients with erosive oral lichen planus. Clinical and Experimental Dermatology, 32(3), 308–310.

Torrente-Castells, E., Figueiredo, R., Berini-Aytes, L., Gay-Escoda, C. (2010). Clinical features of oral lichen planus. A retrospective study of 65 cases. Medicina Oral, Patologia Oral y Cirugia Bucal, 15(5), e685--e690.

Tovaru, S., Parlatescu, I., Gheorghe, C., Tovaru, M., Costache, M., Sardella. (2013). Oral lichen planus: A retrospective study of 633 patients from Bucharest, Romania. Medicina Oral, Patologia Oral y Cirugia Bucal, 18(2), e201--e206.

Vehviläinen, M., Salem, A., Asghar, M. Y., Salo, T., & Siponen, M. (2020). No detection of TSH or TSHR in oral lichen planus lesions in patients with or without hypothyroidism. Acta Odontologica Scandinavica, 78(5), 337–344.

Vilar-Villanueva, M., Gándara-Vila, P., Blanco-Aguilera, E., Otero-Rey, E. M., Rodríguez-Lado, L., García-García, A., & Blanco-Carrión, A. (2019). Psychological disorders and quality of life in oral lichen planus patients and a control group. Oral Diseases, 25(6), 1645–1651.

Wang, C., Li, S., Shen, C., Shan, J., Fan, Y. (2019). Expression and significance of phosphodiesterase 4B gene in peripheral blood of patients with oral lichen planus. International Journal of Dermatology, 58(3), 302–310.

Wiriyakijja, P., Porter, S., Fedele, S., Hodgson, T., McMillan, R., Shephard, M., & Riordain, R. N. (2020). The patient acceptable symptom state in oral lichen planus: identification of cut-off threshold scores in measures of pain and quality of life. Clinical oral investigations. [In Press]

Wu, X., He, X., Wu, S., Xia, H. (2011). Study of association between oral lichen planus and depression in female patients. Shanghai Kou Qiang Yi Xue = Shanghai Journal of Stomatology, 20(5), 548–552.

Yang, C., Liu, L., Shi, H., & Zhang, Y. (2018). Psychological problems and quality of life of patients with oral mucosal diseases: a preliminary study in Chinese population. BMC Oral Health, 18(1), 226.

Zhong, E. F., Chang, A., Stucky, A., Chen, X., Mundluru, T., Khalifeh, M., & Sedghizadeh, P. P. (2020). Genomic Analysis of Oral Lichen Planus and Related Oral Microbiome Pathogens. Pathogens (Basel, Switzerland), 9(11).

11. List of excluded studies with reasons (n=1,445).

11.1 List S3. Mental disorder not reported (n=1,443).

Abbey, L. & Shklar, G. (1971). A histochemical study of oral lichen planus. Oral Surgery, Oral Medicine, and Oral Pathology, 31(2), 226–233.

Abdel Hay, R., Rashed, L., Hegazy, R., Rashwan, W., Samir, N., & Nour-Edin, F. (2016). Association of interleukin (IL)18 and IL10 gene polymorphisms with oral lichen planus risk; a case-control study. Journal of Dermatological Science, 83(3), 244–247.

Abdel-Latif, A. M., Abuel-Ela, H. A., & El-Shourbagy, S. H. (2009). Increased caspase-3 and altered expression of apoptosis-associated proteins, Bcl-2 and Bax in lichen planus. Clinical and Experimental Dermatology, 34(3), 390–395.

Abdolsamadi, H., Rafieian, N., Goodarzi, M. T., Feradmal, J., Davoodi, P., Jazayeri, M., Taghavi, Z., Hoseyni, S.-M., & Ahmadi-Motamayel, F. (2014). Levels of salivary antioxidant vitamins and lipid peroxidation in patients with oral lichen planus and healthy individuals. Chonnam Medical Journal, 50(2), 58–62.

Abed, P. F., Karagah, T., Tabrizi, R., Soltani, L., Warren, R., & Rayati, F. (2017). The Treatment of Recurrent Oral Lichen Planus Lesions Utilizing Free Soft Tissue Autografts: A Case Series. International journal of advanced biotechnology and research, 8(2), 482–488.

Aboushelib, M. N., & Elsafi, M. H. (2017). Clinical Management Protocol for Dental Implants Inserted in Patients with Active Lichen Planus. Journal of Prosthodontics: Official Journal of the American College of Prosthodontists, 26(1), 29–33.

Acay, R. R., Felizzola, C. R., de Araujo, N. & de Sousa, S. O. M. (2006). Evaluation of proliferative potential in oral lichen planus and oral lichenoid lesions using immunohistochemical expression of p53 and Ki67. Oral Oncology, 42(5), 475–480.

Accurso, B. T., Warner, B. M., Knobloch, T. J., Weghorst, C. M., Shumway, B. S., Allen, C. M., & Kalmar, J. R. (2011). Allelic imbalance in oral lichen planus and assessment of its classification as a premalignant condition. Oral Surgery, Oral Medicine, Oral Pathology, Oral Radiology and Endodontology, 112(3), 359–366.

Acha-Sagredo, A., Jimenez, Y., Bagan, J. V, Echebarria-Goicouria, M. A., & Aguirre-Urizar, J. M. (2011). Cytometric analysis of oral scrapings of patients with oral lichen planus. Cytopathology, 22(2), 106–110.

Adami, G. R., Yeung, A. C. F., Stucki, G., Kolokythas, A., Sroussi, H. Y., Cabay, R. J., Kuzin, I., & Schwartz, J. L. (2014). Gene expression based evidence of innate immune response activation in the epithelium with oral lichen planus. Archives of Oral Biology, 59(3), 354–361.

Agha-Hosseini, F, Khalili, M., & Rohani, B. (2009). Immunohistochemistry Analysis of P53 and Ki-67 Proteins in Oral Lichen Planus and Normal Oral Mucosa. Iranian journal of public health, 38(2), 37–43.

Agha-Hosseini, F, Mirzaii-Dizgah, I., Mikaili, S. & Abdollahi, M. (2009). Increased salivary lipid peroxidation in human subjects with oral lichen planus. International Journal of Dental Hygiene, 7(4), 246–250.

Agha-Hosseini, F, Mirzaii-Dizgah, I., Mohebbian, M., & Sarookani, M.-R. (2018). Vascular endothelial growth factor in serum and saliva of oral lichen planus and oral squamous cell carcinoma patients. Journal of Kerman University of Medical Sciences, 25(1), 27–33.

Agha-Hosseini, F, Moslemi, E. & Mirzaii-Dizgah, I. (2012). Comparative evaluation of low-level laser and CO2 laser in treatment of patients with oral lichen planus. International Journal of Oral and Maxillofacial Surgery, 41(10), 1265–1269.

Agha-Hosseini, F., & Mirzaii-Dizgah, I. (2013). p53 as a neoplastic biomarker in patients with erosive and plaque like forms of oral lichen planus. Journal of Contemporary Dental Practice, 14(1), 1–3.

Agha-Hosseini, F., Barati, H., & Moosavi, M. S. (2020). Aquaporin3 (AQP3) expression in oral epithelium in oral lichen planus. Experimental and Molecular Pathology, 115, 104441.

Agha-Hosseini, F., Borhan-Mojabi, K., Monsef-Esfahani, H.-R., Mirzaii-Dizgah, I., Etemad-Moghadam, S., & Karagah, A. (2010). Efficacy of purslane in the treatment of oral lichen planus. Phytotherapy Research, 24(2), 240–244.

Agha-Hosseini, F., Mirzaii-Dizgah, I., & Miri-Zarandi, N. (2015). Unstimulated salivary p53 in patients with oral lichen planus and squamous cell carcinoma. Acta Medica Iranica, 53(7), 439–443.

Agha-Hosseini, F., Mirzaii-Dizgah, I., & Mohammadpour, N. (2016). Muscarinic cholinergic receptors (MR3) in saliva of patients with oral lichen planus. Archives of Dermatological Research, 308(7), 481–486.

Agha-Hosseini, F., Mohebbian, M., Sarookani, M.-R., Harirchi, I., & Mirzaii-Dizgah, I. (2015). Comparative evaluation of EGF in oral lichen planus and oral squamous cell carcinoma. Acta Medica Iranica, 53(8), 471–475.

Agha-Hosseini, F., Moosavi, M. S., Mirzaii-Dizgah, I., & Samami, M. (2020). Muscarinic cholinergic receptors in minor salivary gland tissues of patients with oral lichen planus: A case-control study. Journal of Oral Pathology & Medicine: Official Publication of the International Association of Oral Pathologists and the American Academy of Oral Pathology, 49(8), 816–821.

Agha-Hosseini, Farzaneh, & Mirzaii-Dizgah, I. (2015). Serum and saliva collagenase-3 (MMP-13) in patients with oral lichen planus and oral squamous cell carcinoma. Medical Journal of the Islamic Republic of Iran, 29, 218.

Agha-Hosseini, Farzaneh, Imanpour, M., Mirzaii-Dizgah, I., & Moosavi, M.-S. (2017). Mucin 5B in saliva and serum of patients with oral lichen planus. Scientific Reports, 7(1), 12060.

Agha-Hosseini, Farzaneh, Mirzaii-Dizgah, I., Farmanbar, N., & Abdollahi, M. (2012). Oxidative stress status and DNA damage in saliva of human subjects with oral lichen planus and oral squamous cell carcinoma. Journal of Oral Pathology and Medicine, 41(10), 736–740.

Agha-Hosseini, Farzaneh, Mirzaii-Dizgah, I., Mahboobi, N., Shirazian, S., & Harirchi, I. (2015). Serum and Saliva MMP-3 in Patients with OLP and Oral SCC. The Journal of Contemporary Dental Practice, 16(2), 107–111.

Aghahosseini, F., Arbabi-Kalati, F., Fashtami, L. A., Djavid, G. E., Fateh, M., Beitollahi, J. M. (2006). Methylene blue-mediated photodynamic therapy: A possible alternative treatment for oral lichen planus. Lasers in Surgery and Medicine, 38(1), 33–38.

Aghbari, S. M. H., Abushouk, A. I., Shakir, O. G., Zayed, S. O., Attia, A. (2018). Correlation between tissue expression of microRNA-137 and CD8 in oral lichen planus. Clinical Oral Investigations, 22(3), 1463–1467.

Aghbari, S. M. H., Gaafar, S. M., Shaker, O. G., Ashiry, S. El, Zayed, S. O. (2018). Evaluating the accuracy of microRNA27b and microRNA137 as biomarkers of activity and potential malignant transformation in oral lichen planus patients. Archives of Dermatological Research, 310(3), 209–220.

Aguirre, J. M., Bagan, J. V, Rodriguez, C., Jimenez, Y., Martinez-Conde, R., de Rojas, F., Ponte, A. (2004). Efficacy of mometasone furoate microemulsion in the treatment of erosive-ulcerative oral lichen planus: Pilot study. Journal of Oral Pathology and Medicine, 33(7), 381–385.

Ahlgren, C., Axell, T., Moller, H., Isaksson, M., Liedholm, R., & Bruze, M. (2014). Contact allergies to potential allergens in patients with oral lichen lesions. Clinical Oral Investigations, 18(1), 227–237.

Ahlgren, C., Bruze, M., Moller, H., Gruvberger, B., Axell, T., Liedholm, R., & Nilner, K. (2012). Contact allergy to gold in patients with oral lichen lesions. Acta Dermato-Venereologica, 92(2), 138–143.

Ahlgren, C., Isaksson, M., Moller, H., Axell, T., Liedholm, R., & Bruze, M. (2014). The necessity of a test reading after 1 week to detect late positive patch test reactions in patients with oral lichen lesions. Clinical Oral Investigations, 18(5), 1525–1531.

Ahmadi-Motamayel, F., Bayat, Z., Hajilooi, M., Shahryar-Hesami, S., Mahdavinezhad, A., Samie, L., & Solgi, G. (2017). Evaluation of the miRNA-146a and miRNA-155 Expression Levels in Patients with Oral Lichen Planus. Iranian Journal of Immunology, 14(4), 316–324.

Ahmed Haji Omar, A., Hietanen, J., Kero, M., Lukinmaa, P.-L. P.-L., Hagstrm, J., Omar, A. A. H., Hietanen, J., Kero, M., Lukinmaa, P.-L. P.-L., Hagstrom, J. (2009). Oral lichen planus and chronic junctional stomatitis: Differences in lymphocyte subpopulations. Acta Odontologica Scandinavica, 67(6), 366–369.

Akpinar Kara, Y. (2017). The measurement of serum TNF-alpha levels in patients with lichen planus. Acta Dermatovenerologica Alpina, Pannonica, et Adriatica, 26(4), 85–88.

Aksoy, B., & Hapa, F. A. (2018). Do we really not need to treat patients with white reticular lesions of oral lichen planus?: Case control pilot study. Turkderm-turkish archives of dermatology and venerology, 52(1), 24–28.

Al-Drobie, B. F., & Al-Hasnawi, Z. A. (2018). Assessment of SNPs (rs4774, rs6498122) of CIITA Gene in Buccal Swabs and Blood of Oral Lichen Planus Patients in Iraq. International journal of medical research & health sciences, 7(1), 110–115.

Al-Hamadi, K. I. (2008). Can deram be a cause of oral lichen planus?, Saudi Medical Journal, 29(7), 1028–1030.

Al-Hassiny, A., Friedlander, L. T., Parachuru, V. P. B., Seo, B., Hussaini, H. M., & Rich, A. M. (2018). Upregulation of angiogenesis in oral lichen planus. Journal of Oral Pathology & Medicine: Official Publication of the International Association of Oral Pathologists and the American Academy of Oral Pathology, 47(2), 173–178.

Al-Janaby, H., El-Sakka, H., Masood, M., Ashani W Mendis, W., M Slack-Smith, L., Parsons, R., & M Frydrych, A. (2017). Xerostomia and Salivary Gland Hypofunction in Patients with Oral Lichen Planus Before and After Treatment with Topical Corticosteroids. The Open Dentistry Journal, 11, 155–163.

Al-Mohaya, M. A., Al-Harthi, F., Arfin, M., & Al-Asmari, A. (2015). TNF-alpha, TNF-beta and IL-10 gene polymorphism and association with oral lichen planus risk in Saudi patients. Journal of Applied Oral Science : Revista FOB, 23(3), 295–301.

Alaeddini, M., Barghammadi, R., Eshghyar, N., & Etemad-Moghadam, S. (2014). An analysis of biopsy-proven tongue lesions among 8,105 dental outpatients. The Journal of Contemporary Dental Practice, 15(1), 1–7.

Albanidou-Farmaki, E., Kayavis, I., Sideropoulos, I., Papanayiotou, P., & Polymenidis, Z. (1990). Serum immunoglobulins IgA, IgG and IgM, and oral lichen planus. Stomatologia, 47(2), 114–120.

Alberdi-Navarro, J., Marichalar-Mendia, X., Lartitegui-Sebastian, M.-J., Gainza-Cirauqui, M.-L., Echebarria-Goikouria, M.-A., Aguirre-Urizar, J.-M (2017). Histopathological characterization of the oral lichenoid disease subtypes and the relation with the clinical data. Medicina Oral, Patologia Oral y Cirugia Bucal, 22(3), e307--e313.

Alejandra Masquijo-Bisio, P., Silvia Gandolfo, M., Keszler, A., Elina Itoiz, M., & Luisa Paparella, M. (2017). Usefulness of a direct immunofluorescence in the diagnosis of plaque type oral lichen planus. Annals of diagnostic pathology, 31, 20–22.

Aliev, A. D., Mikhailovskii, V. M., Perlamutrov, Y. N., Kushlinskii, N. E. (2007). Soluble Fas antigen in the serum of women with oral lichen planus. Bulletin of Experimental Biology and Medicine, 143(6), 727–729.

Alikhani, M., Ghalaiani, P., Askariyan, E., Khunsaraki, Z. A., Tavangar, A., Naderi (2017). Association between the clinical severity of oral lichen planus and anti-TPO level in thyroid patients. Brazilian Oral Research, 31, e10–e10.

Allen, C. M., Beck, F. M., Rossie, K. M., & Kaul, T. J. (1986). Relation of stress and anxiety to oral lichen planus. Oral Surgery Oral Medicine and Oral Pathology, 61(1), 44–46.

Allen, K., & Farah, C. S. (2015). Patient perspectives of diagnostic delay for suspicious oral mucosal lesions. Australian Dental Journal, 60(3), 397–403.

Allon, I., Vered, H., Hirshberg, A (2015). Programmed cell removal biomarkers calreticulin and CD47 implicated in oral lichen planus. Oral Diseases, 21(7), 894–898.

Almoznino, G., Zadik, Y., Vered, M., Becker, T., Yahalom, R., Derazne, E., Aframian, D. J., & Czerninski, R. (2015). Oral and maxillofacial pathologies in young- and middle-aged adults. Oral Diseases, 21(4), 493–500.

Amanat, D., Ebrahimi, H., Zahedani, M. Z., Zeini, N., Pourshahidi, S., Ranjbar, Z (2014). Comparing the effects of cryotherapy with nitrous oxide gas versus topical corticosteroids in the treatment of oral lichen planus. Indian Journal of Dental Research: Official Publication of Indian Society for Dental Research, 25(6), 711–716.

Aminzadeh, A., Jahanshahi, G., & Ahmadi, M. (2013). A retrospective comparative study on clinico-pathologic features of oral lichen planus and oral lichenoid lesions. Dental Research Journal, 10(2), 168–172.

Amirchaghmaghi, M., Delavarian, Z., Iranshahi, M., Shakeri, M. T., Mosannen Mozafari, P., Mohammadpour, A. H., Farazi, F., & Iranshahy, M. (2015). A Randomized Placebo-controlled Double Blind Clinical Trial of Quercetin for Treatment of Oral Lichen Planus. Journal of Dental Research, Dental Clinics, Dental Prospects, 9(1), 23–28.

Amirchaghmaghi, M., Hashemy, S. I., Alirezaei, B., Jahed Keyhani, F., Kargozar, S., Vasigh, S., Gharaei, S., & Pakfetrat, A. (2016). Evaluation of Plasma Isoprostane in Patients with Oral Lichen Planus. Journal of Dentistry (Shiraz, Iran), 17(1), 21–25.

Amirchaghmaghi, M., Pakfetrat, A., Delavarian, Z., Ghalavani, H., Ghazi, A (2016). Evaluation of the efficacy of curcumin in the treatment of oral lichen planus: A randomized controlled trial. Journal of Clinical and Diagnostic Research, 10(5), ZC134-ZD137.

Andreasen, J. O. (1968). Oral lichen planus 2. A histologic evaluation of 97 cases. Oral surgery oral medicine oral pathology oral radiology and endodontology, 25(2), 158-158.

Angelin, D., & Nair, B. J. (2020). Comparative evaluation of survivin expression in leukoplakia, lichen planus, and oral squamous cell carcinoma: An immunohistochemical study. Journal of Cancer Research and Therapeutics, 16(3), 569–574.

Anitua, E., Pinas, L., Escuer-Artero, V., Fernandez, R. S., & Alkhraisat, M. H. (2018). Short dental implants in patients with oral lichen planus: a long-term follow-up. British Journal of Oral and Maxillofacial Surgery, 56(3), 216–220.

Aniyan, K. Y., Guledgud, M. V, & Patil, K. (2018). Alterations of Serum Lipid Profile Patterns in Oral Lichen Planus Patients: A Case-Control Study. Contemporary Clinical Dentistry, 9, S112--S121.

Antonenko, M., Paryi, A., Zelinskaya, N., Palamarchuk, S., & Mayborodina, D. (2017). Role of oral hygiene in the immuno-genetic component of the pathogenesis of oral lichen planus. international journal of medical dentistry, 21(3), 178–182.

Arão, T. C., Guimarães, A. L. S., Batista De Paula, A. M., Gomes, C. C., Gomez, R. S. (2012). Increased miRNA-146a and miRNA-155 expressions in oral lichen planus. Archives of Dermatological Research, 304(5), 371–375.

Arbabi-Kalati, F., & Farahmand, M.-M. (2017). Evaluation of the efficacy of lycopene in the management of oral lichen planus: A pilot randomized clinical trial. Tehran University Medical Journal, 75(9), 658–662.

Arduino, P. G. et al. (2014) 'Pimecrolimus vs. tacrolimus for the topical treatment of unresponsive oral erosive lichen planus: A 8 week randomized double-blind controlled study', Journal of the european academy of dermatology and venereology, 28(4), pp. 475–482.

Arduino, P. G. et al. (2017) 'Subgingival Microbiota in White Patients With Desquamative Gingivitis: A Cross-Sectional Study', Journal of periodontology, 88(7), pp. 643–650.

Arduino, P. G. et al. (2018) 'Randomized, placebo-controlled, double-blind trial of clobetasol propionate 0.05% in the treatment of oral lichen planus', Oral Diseases, 24(5), pp. 772–777.

Arirachakaran, P., Chansaengroj, J., Lurchachaiwong, W., Kanjanabud, P., Thongprasom, K., Poovorawan (2013). Oral lichen planus in Thai patients has a low prevalence of human papillomavirus. ISRN Dentistry, 2013, 362750.

Arreaza, A. J., Rivera, H., Correnti, M. (2014). Expression of COX-2 and bcl-2 in oral lichen planus lesions and lichenoid reactions. E cancer medical science, 8(1), 411.

Arreaza, A., Rivera, H., & Correnti, M. (2015). p53 expression in oral lichenoid lesions and oral lichen planus. General Dentistry, 63(1), 69–72.

Artico, G., Freitas, R. S., Santos Filho, A. M., Benard, G., Romiti, R., & Migliari, D. A. (2014). Prevalence of Candida spp., xerostomia, and hyposalivation in oral lichen planus - A controlled study. Oral Diseases, 20(3), e36--e41.

Arunkumar, S., Kalappanavar, A. N., Annigeri, R. G., & Kalappa, S. G. (2015). Relative efficacy of pimecrolimus cream and triamcinolone acetonide paste in the treatment of symptomatic oral lichen planus. Indian Journal of Dentistry, 6(1), 14–19.

Attia, E. A. S., Abdel Fattah, N. S. A., & Abdella, H. M. (2010). Upper gastrointestinal findings and detection of Helicobacter pylori in patients with oral lichen planus: Clinical dermatology. Clinical and Experimental Dermatology, 35(4), 355–360.

Awan, K. H., Morgan, P. R., & Warnakulasuriya, S. (2011). Utility of chemiluminescence (ViziLite) in the detection of oral potentially malignant disorders and benign keratoses. Journal of Oral Pathology & Medicine: Official Publication of the International Association of Oral Pathologists and the American Academy of Oral Pathology, 40(7), 541–544.

Awan, K. H., Morgan, P. R., Warnakulasuriya (2011). Evaluation of an autofluorescence based imaging system (VELscopeTM) in the detection of oral potentially malignant disorders and benign keratoses. Oral Oncology, 47(4), 274–277.

Azab, N. A., Abd El Salam, L., Ahmed, E., El Sharkawy, M., ElSharkawy, A., & El Asheiry, S. G. (2018). Interferon gamma and interleukin 8 gene polymorphisms in

patients with hepatitis C virus related oral lichen planus. Archives of Oral Biology, 96, 189–194.

Azizi, A., & Farshchi, F. (2012). Comparison of salivary and plasma antioxidant levels in lichen planus patients and healthy subjects. Journal of Oral Pathology & Medicine: Official Publication of the International Association of Oral Pathologists and the American Academy of Oral Pathology, 41(7), 524–526.

Azizi, A., & Lawaf, S. (2007). The comparison of efficacy of adcortyl ointment and topical tacrolimus in treatment of erosive oral lichen planus. Journal of Dental Research, Dental Clinics, Dental Prospects, 1(3), 99–102.

Azizi, A., & Rezaee, M. (2012). Comparison of periodontal status in gingival oral lichen planus patients and healthy subjects. Dermatology Research and Practice, 2012, 561232.

Azizi, A., Dadras, O. G., Jafari, M., Ghadim, N. M., Lawaf, S., & Sadri, D. (2016). Efficacy of 0.1% triamcinolone with nanoliposomal carrier formulation in orabase for oral lichen planus patients: A clinical trial. European journal of integrative medicine, 8(3), 275–280.

Backman, K., & Jontell, M. (2007). Microbial-associated oral lichenoid reactions. Oral Diseases, 13(4), 402–406.

Bagan Sebastian, J. V, Aguirre Urizar, J. M., Milian Masanet, A., Penarrocha Diago, M., Garcia Pola Vallejo, M. J. (1991). A morphometric study of 74 cases of oral lichen planus. Revue de Stomatologie et de Chirurgie Maxillo-Faciale, 92(4), 265–268.

Bagan, J. V, Donat, J. S., Penarrocha, M., Milian, M. A., Sanchis, J. M. (1993). Oral lichen planus and diabetes mellitus. A clinico-pathological study. Bulletin Du Groupement International Pour La Recherche Scientifique En Stomatologie & Odontologie, 36(1), 3–6.

Baghaei, F., Shojaei, S., Afshar-Moghaddam, N., Zargaran, M., Rastin, V., Nasr, M., & Moghimbeigi, A. (2015). Study of P21 Expression in Oral Lichen Planus and Oral Squamous Cell Carcinoma by Immunohistochemical Technique. Journal of Dentistry (Shiraz, Iran), 16(3), 156–161.

Bahramian, A., Bahramian, M., Mehdipour, M., Falsafi, P., Khodadadi, S., Dabaghi Tabriz, F., & Deljavanghodrati, M. (2018). Comparing Vitamin D Serum Levels in Patients with Oral Lichen Planus and Healthy Subjects. Journal of Dentistry (Shiraz, Iran), 19(3), 212–216.

Bai, J, Zhang, Y., Lin, M., Zeng, X., Wang, Z., Shen, J., Jiang, L., Gao, F., Chen, Q. (2007). Interleukin-18 gene polymorphisms and haplotypes in patients with oral lichen planus: A study in an ethnic Chinese cohort. Tissue Antigens, 70(5), 390–397.

Bai, Jingping, Jiang, L., Lin, M., Zeng, X., Wang, Z., & Chen, Q. (2009). Association of polymorphisms in the tumor necrosis factor-alpha and interleukin-10 genes with oral lichen planus: a study in a chinese cohort with Han ethnicity. Journal of Interferon & Cytokine Research: The Official Journal of the International Society for Interferon and Cytokine Research, 29(7), 381–388.

Bai, Jingping, Lin, M., Zeng, X., Zhang, Y., Wang, Z., Shen, J., Jiang, L., Gao, F., Chen, Q. (2008). Association of polymorphisms in the human IFN-$γ$ and IL-4 gene with oral lichen planus: A study in an ethnic Chinese cohort. Journal of Interferon and Cytokine Research, 28(6), 351–358.

Bán, A., Marincsák, R., Bíró, T., Perkecz, A., Gömöri, E., Sándor, K., Tóth, I. B., Bánvölgyi, A., Szolcsányi, J., & Pintér, E. (2010). Upregulation of transient receptor potential vanilloid type-1 receptor expression in oral lichen planus. NeuroImmunoModulation, 17(2), 103–108.

Bánóczy, J., Roed-Petersen, B., Pindborg, J. J., & Inovay, J (1979) 'Clinical and histologic studies on electrogalvanically induced oral white lesions', Oral Surgery Oral Medicine and Oral Pathology, 48(4), pp. 319–323.

Bao, Z. X. et al. (2020) 'The profile of hematinic deficiencies in patients with oral lichen planus: a case-control study.', BMC oral health, 20(1), p. 252.

Bao, Z.-X., Yang, X.-W., Shi, J., Liu, L.-X. (2016). Serum zinc levels in 368 patients with oral mucosal diseases: A preliminary study. Medicina Oral, Patologia Oral y Cirugia Bucal, 21(3), e335--e340.

Barabash, A. G., Kats, A. G., & Getling, Z. M. (1995). [Experience in treating patients with lichen ruber planus by using a helium-neon laser]. Stomatologiia, 74(1), 20–21.

Barabash, A. G., Tsvetkova, G. M., Prokaeva, T. B., & Getling, Z. M. (1998). [The clinico-morphological basis for the combined treatment of lichen ruber planus of the oral mucosa using Solcoseryl]. Stomatologiia, 77(3), 31–33.

Barakat, S. M. M., Siar, C. H. (2015). Differential expression of stem cell-like proteins in normal, hyperplastic and dysplastic oral epithelium. Journal of Applied Oral Science: Revista FOB, 23(1), 79–86.

Bascones-Ilundain, C., Gonzalez-Moles, M. A., Esparza-Gomez, G., Gil-Montoya, J. A., & Bascones-Martinez, A. (2006). Importance of apoptotic mechanisms in inflammatory infiltrate of oral lichen planus lesions. Anticancer Research, 26(1), 357–362.

Bascones-Ilundain, C., Gonzalez-Moles, M. A., Esparza, G., Gil-Montoya, J. A., Bascones-Martinez, A. (2007). Significance of liquefaction degeneration in oral lichen planus: A study of its relationship with apoptosis and cell cycle arrest markers. Clinical and Experimental Dermatology, 32(5), 556–563.

Bascones, C., Gonzalez-Moles, M. A., Esparza, G., Bravo, M., Acevedo, A., Gil-Montoya, J. A. (2005). Apoptosis and cell cycle arrest in oral lichen planus: Hypothesis on their possible influence on its malignant transformation. Archives of Oral Biology, 50(10), 873–881.

Basheer, S., Shameena, P. M., Sudha, S., Varma, S., Vidyanath, S., & Varekar, A. (2017). Expression of survivin and p53 in oral lichen planus, lichenoid reaction and lichenoid dysplasia: An immunohistochemical study. Journal of Oral and Maxillofacial Pathology, 21(3), 456–457.

Battino, M., Greabu, M., Totan, A., Bullon, P., Bucur, A., Tovaru, S., Mohora, M., Didilescu, A., Parlatescu, I., Spinu, T., & Totan, C. (2008) 'Oxidative stress markers in oral lichen planus', BioFactors, 33(4), pp. 301–310.

Batu, S., Ofluoglu, D., Ergun, S., Warnakulasuriya, S., Uslu, E., Guven, Y., Tanyeri, H. (2016). Evaluation of prolidase activity and oxidative stress in patients with oral lichen planus and oral lichenoid contact reactions. Journal of Oral Pathology and Medicine, 45(4), 281–288.

Baudet-Pommel, M., Janin-Mercier, A., Souteyrand, P. (1991). Sequential immunopathologic study of oral lichen planus treated with tretinoin and etretinate. Oral Surgery Oral Medicine and Oral Pathology, 71(2), 197–202.

Baudet-Pommel, M., Janin-Mercier, A., Souteyrand, P., & Peri, G. (1990) 'Ongoing clinical study of oral lichen planus (OLP) treated with retinoids: one used locally, (tretinoin) the other orally (etretinate)', Actualités odonto-stomatologiques, 44(170), pp. 337–348.

Baykal, L., Arica, D. A., Yayli, S., Orem, A., Bahadir, S., Altun, E., & Yaman, H. (2015). Prevalence of Metabolic Syndrome in Patients with Mucosal Lichen Planus: A Case-Control Study. American Journal of Clinical Dermatology, 16(5), 439–445.

Becker, J., Loning, T., Reichart, P., Hartmann, N. (1983). Oral lichen planus - characterization of immunocompetent cells with hybridoma antibodies. Journal of Oral Pathology, 12(2), 117–123.

Becker, J., Schuppan, D. (1995). Altered expression of extracellular matrix proteins and integrins in oral lichen planus (OLP). Journal of Oral Pathology and Medicine, 24(4), 159–164.

Becker, J., von Keyeserlingk-Eberius, H., Neukam, F. W., Marggraf, E., & Reichart, P. (1985) 'The distribution of histocompatibility antigen in patients with oral lichen planus', Deutsche Zeitschrift für Mund-, Kiefer- und Gesichts-Chirurgie, 9(3), pp. 181–183.

Bediaga, N. G., Marichalar-Mendia, X., Aguirre-Urizar, J. M., Calvo, B., Echebarria-Goicouria, M. A., de Pancorbo, M. M., & Acha-Sagredo, A. (2014). Global DNA methylation: uncommon event in oral lichenoid disease. Oral Diseases, 20(8), 821–826.

Beevi, B. H., Nayak, S. R., Peter, C. D., Haridas, A. K., Jacob, L., Aboobakker, A. (2019). Analysis of Ki-67 expression in oral premalignant lesions and normal oral mucosa: An immunohistochemical study. Journal of Pharmacy and Bioallied Sciences, 11(6), S232--S235.

Beigom Taheri, J., Anbari, F., Maleki, Z., Boostani, S., Zarghi, A., & Pouralibaba, F. (2010). Efficacy of Elaeagnus angustifolia Topical Gel in the Treatment of Symptomatic Oral Lichen Planus. Journal of Dental Research, Dental Clinics, Dental Prospects, 4(1), 29–32.

Belal, M. H. (2015). Management of symptomatic erosive-ulcerative lesions of oral lichen planus in an adult Egyptian population using Selenium-ACE combined with topical corticosteroids plus antifungal agent. Contemporary Clinical Dentistry, 6(4), 454–460.

Bennardo, F., Liborio, F., Barone, S., Antonelli, A., Buffone, C., Fortunato, L., & Giudice, A. (2021). Efficacy of platelet-rich fibrin compared with triamcinolone acetonide as injective therapy in the treatment of symptomatic oral lichen planus: a pilot study. Clinical oral investigations. Advance online publication.

Bergdahl, J., Ostman, P. O., Anneroth, G., Perris, H., & Skoglund, A. (1995). Psychologic aspects of patients with oral lichenoid reactions. Acta Odontologica Scandinavica, 53(4), 236–241.

Bermejo-Fenoll, A, Sanchez-Siles, M., Lopez-Jornet, P., Camacho-Alonso, F., Salazar-Sanchez, N. (2009). Premalignant nature of oral lichen planus. A retrospective study of 550 oral lichen planus patients from south-eastern Spain. Oral Oncology, 45(8), e54--e56.

Bermejo-Fenoll, Ambrosio, Lopez-Jornet, P., Camacho-Alonso, F., Saura-Ingles, A., Panchon-Ruiz, A. (2009). Morphometric analysis of the dorsum linguae in patients with Oral Lichen Planus. Medicina Oral, Patologia Oral y Cirugia Bucal, 14(8), e388--e392.

Bessar H, Hammad NM, Ismail T, Teama MA and Youssef A (2021), "Serum angiopoietin-2 level as a novel potential biomarker in oral lichen planus.", Journal of cosmetic dermatology.

Best, D. L., Herzog, C., Powell, C., Braun, T., Ward, B. B., & Moe, J. (2020). Oral Lichen Planus-Associated Oral Cavity Squamous Cell Carcinoma Is Associated With Improved Survival and Increased Risk of Recurrence. Journal of Oral and Maxillofacial Surgery, 78(7), 1193–1202.

Bez, C., Hallett, R., Carrozzo, M., Lodi, G., Gandolfo, S., Carrassi, A., Scully, C., Porter, S. (2001). Lack of association between hepatotropic transfusion transmitted virus infection and oral lichen planus in British and Italian populations. British Journal of Dermatology, 145(6), 990–993.

Bez, C., Moneghini, L., Nicali, A., Cazzaniga, A., Lodi, G., Bosari, S., Sardella, A., & Carrassi, A. (2006). Characterization of induced mucosal connective tissue separation - A comparison of six different techniques. Journal of Cutaneous Pathology, 33(3), 220–226.

Bhatnagar, P., Rai, S., Bhatnagar, G., Kaur, M., Goel, S., & Prabhat, M. (2013). Prevalence study of oral mucosal lesions, mucosal variants, and treatment required for patients reporting to a dental school in North India: In accordance with WHO guidelines. Journal of Family & Community Medicine, 20(1), 41–48.

Bhuvana, K., Suma, G. N., Mamatha, N. S., Sowbhagya, M. B., Komali, G., Krishnamoorthy, B. (2014). Lipid profile and metabolic syndrome status in patients with oral lichen planus, oral lichenoid reaction and healthy individuals attending a dental college in northern India -a descriptive study. Journal of Clinical and Diagnostic Research, 8(11), ZC92--ZC95.

Bianco, L., Romano, F., Maggiora, M., Bongiovanni, L., Guzzi, N., Curmei, E., Arduino, P. G., Aimetti, M. (2019). Effect of sonic versus manual supervised toothbrushing on both clinical and biochemical profiles of patients with desquamative gingivitis associated

with oral lichen planus: A randomized controlled trial. International Journal of Dental Hygiene, 17(2), 161–169.

Bidarra, M., Buchanan, J. A., Scully, C., Moles, D. R., & Porter, S. R. (2008). Oral lichen planus: a condition with more persistence and extra-oral involvement than suspected?. Journal of Oral Pathology and Medicine, 37(10), pp. 582–586.

Biocina-Lukenda, D., Cekic-Arambasin, A., Markeljevic, J., & Bukovic, D. (2008). Serum immunoglobulins IgG, IgA and IgM in patients with oral lichen ruber. Collegium Antropologicum, 32(1), 161–163.

Bloor, B. K., Malik, F. K., Odell, E. W., Morgan, P. R. (1999). Quantitative assessment of apoptosis in oral lichen planus. Oral Surgery, Oral Medicine, Oral Pathology, Oral Radiology, and Endodontics, 88(2), 187–195.

Bloor, B. K., Seddon, S. V, Morgan, P. R. (2000). Gene expression of differentiation-specific keratins (K4, K13, K1 and K10) in oral non-dysplastic keratoses and lichen planus. Journal of Oral Pathology and Medicine, 29(8), 376–384.

Bocheva, G. (2010). A possible role of mast cell tryptase and proteinase-activated receptor-2 in the pathogenesis of oral lichen planus. comptes rendus de l academie bulgare des sciences, 63(7), 1057–1060.

Boisnic, S., Branchet, M. C., Pascal, F., Ben Slama, L., Rostin, M., & Szpirglas, H. (1994). [Topical tretinoin in the treatment of lichen planus and leukoplakia of the mouth mucosa. A clinical evaluation]. Annales de Dermatologie et de Venereologie, 121(6), 459–463.

Bombeccari, G. P., Gianni, A. B., & Spadari, F. (2017). Immunoexpression of cytokeratin-19 in the oral lichen planus and related oral squamous cell carcinoma. Annali Di Stomatologia, 8(3), 104–109.

Bomstein, M. M., Hakimi, B., Persson, G. R. (2008). Microbiological findings in subjects with asymptomatic oral lichen planus: A cross-sectional comparative study. Journal of Periodontology, 79(12), 2347–2355.

Bonnardot, L., Bardet, E., Steichen, O., Cassagnau, E., Piot, B., Salam, A. P., Campion, L., Ferron, C., de Montreuil, C., Malard, O. (2011). Prognostic factors for T1-T2 squamous cell carcinomas of the mobile tongue: A retrospective cohort study. Head and Neck, 33(7), 928–934.

Boñar-Alvarez, P., Pérez Sayáns, M., Garcia-Garcia, A., Chamorro-Petronacci, C., Gándara-Vila, P., Luces-González, R., Otero Rey, E., Blanco-Carrión, A., Suárez-Peñaranda, J. M. (2019). Correlation between clinical and pathological features of oral lichen planus: A retrospective observational study. Medicine, 98(8), e14614–e14614.

Boras, V. V., Rogic, D., Brailo, V., Vidovic-Juras, D., Glazar, I., & Muhvic-Urek, M. (2011). Sialochemistry in Patients with Oral Lichen Planus. Acta stomatologica croatica, 45(3), 184–189.

Bornstein, M. M., Frei, M., Sendi, P., Ramseier, C. A., & Reichart, P. A. (2012). Patients' awareness of the potential benefit of smoking cessation. A study evaluating self-reported

and clinical data from patients referred to an oral medicine unit. Clinical Oral Investigations, 16(1), 55–62.

Bouza, M. D., Simon, M. A., & Seoane, J. M. (2002). An evaluation of pharmacological treatment combined with stress inoculation training in the management of oral lichen planus. Psychology & health, 17(6), 793–799.

Brands, R. C., Kohler, O., Rauthe, S., Hartmann, S., Ebhardt, H., Seher, A., Linz, C., Kubler, A. C., Muller-Richter, U. (2017). The prognostic value of GLUT-1 staining in the detection of malignant transformation in oral mucosa. Clinical Oral Investigations, 21(5), 1631–1637.

Bratel, J., Dahlgren, U., Simark Mattsson, C., & Jontell, M. (1998) 'The frequency of different T-cell receptor V-families in oral lichen planus and lichenoid contact lesions: An immunohistochemical study', Journal of Oral Pathology and Medicine, 27(9), pp. 415–419.

Bratel, J., Hakeberg, M., Jontell, M. (1996). Effect of replacement of dental amalgam on oral lichenoid reactions. Journal of Dentistry, 24(1), 41–45.

Brennan, P. A., Umar, T., Palacios-Callender, M., Spedding, A. V, Mellor, T. K., Buckley, J., Langdon, J. D. (2000). A study to assess inducible nitric oxide synthase expression in oral lichen planus. Journal of Oral Pathology and Medicine, 29(6), 249–254.

Brunotto, M., Zárate, A. M., Cismondi, A., Fernández, M., & Noher de Halac, R. I. (2005) 'Valuation of exfoliative cytology as prediction factor in oral mucosa lesions.', Medicina oral, patología oral y cirugía bucal, 10, pp. E92--102.

Brzak, B. L., Mravak-Stipetic, M., Canjuga, I., Baricevic, M., Balicevic, D., Sikora, M., Filipovic-Zore, I. (2012). The frequency and malignant transformation rate of oral lichen planus and leukoplakia - A retrospective study. Collegium Antropologicum, 36(3), 773–777.

Buajeeb, W., Kraivaphan, P., Amornchat, C., Suthamajariya, K. (2008). Reduction of micronuclei in oral lichen planus supplemented with beta-carotene. Journal of Oral Science, 50(4), 461–467.

Byakodi, R., Shipurkar, A., Byakodi, S., Marathe, K. (2011). Prevalence of oral soft tissue lesions in Sangli, India. Journal of Community Health, 36(5), 756–759.

Byun, J.-S., Hong, S.-H., Choi, J.-K., Jung, J.-K., Lee, H.-J. (2015). Diagnostic profiling of salivary exosomal microRNAs in oral lichen planus patients. Oral Diseases, 21(8), 987–993.

Cafaro, A., Arduino, P. G., Massolini, G., Romagnoli, E., & Broccoletti, R. (2014). Clinical evaluation of the efficiency of low-level laser therapy for oral lichen planus: A prospective case series. Lasers in Medical Science, 29(1), 185–190.

Calenic, B., Okamura, K., Yaegaki, K., Tovaru, S., Tanaka, T., & Imai, T. (2014). Role of p53-mediated apoptotic pathway in oral lichen planus: Relationship among pro-

apoptotic, anti-apoptotic, and keratinocytic markers. Journal of oral and maxillofacial surgery medicine and pathology, 26(2), 221–227.

Camacho-Alonso, F., Lopez-Jornet, P., Bermejo-Fenoll, A. (2007). Gingival involvement of oral lichen planus. Journal of Periodontology, 78(4), 640–644.

Campisi, G., Giandalia, G., De Caro, V., Di Liberto, C., Arico, P., & Giannola, L. I. (2004). A new delivery system of clobetasol-17-propionate (lipid-loaded microspheres 0.025%) compared with a conventional formulation (lipophilic ointment in a hydrophilic phase 0.025%) in topical treatment of atrophic/erosive oral lichen planus. British Journal of Dermatology, 150(5), 984–990.

Campisi, G., Giovannelli, L., Arico, P., Lama, A., Di Liberto, C., Ammatuna, P., & D'Angelo, M. (2004). HPV DNA in clinically different variants of oral leukoplakia and lichen planus. Oral Surgery, Oral Medicine, Oral Pathology, Oral Radiology, and Endodontics, 98(6), 705–711.

Čanković, M., Bokor-Bratić, M., & Novović, Z. (2015). Stressful Life Events and Personality Traits in Patients with Oral Lichen Planus. Acta Dermatovenerologica Croatica, 23(4), pp. 270–276.

Cao, H. K., Zhang, S. L., Xu, W. N., & Zhou, Z. T. (1996). [Teatment of oral lichen planus with chloroquine and triamcinolone-A]. Shanghai Kou Qiang Yi Xue = Shanghai Journal of Stomatology, 5(3), 140–142.

Cao, J., Jin, J. Q., Deng, D. J., & Liu, H. W. (2016) 'Determination of human papillomavirus in oral leukoplakia,oral lichen planus and oral squamous cell carcinoma', Beijing da xue xue bao. Yi xue ban = Journal of Peking University. Health sciences, 48(1), pp. 84–88.

Cao, J., Liu, H. W., Liu, X. S., Jin, J. Q., & Zhang, P. (2011) '[Correlation between the quantity of oral mucosal micronucleus cells and cancerization].', Beijing da xue xue bao. Yi xue ban = Journal of Peking University. Health sciences, 43(4), pp. 600–602. A

Carbone, M., Arduino, P. G., Carrozzo, M., Caiazzo, G., Broccoletti, R., Conrotto, D., Bezzo, C., & Gandolfo, S. (2009). Topical clobetasol in the treatment of atrophic-erosive oral lichen planus: a randomized controlled trial to compare two preparations with different concentrations. Journal of Oral Pathology and Medicine, 38(2), pp. 227–233.

Carbone, M., Carrozzo, M., Castellano, S., Conrotto, D., Broccoletti, R., & Gandolfo, S. (1998). Systemic corticosteroid therapy of oral vesiculoerosive diseases (OVED). An open trial. Minerva Stomatologica, 47(10), 479–487.

Carbone, M., Goss, E., Carrozzo, M., Castellano, S., Conrotto, D., Broccoletti, R., Gandolfo, S. (2003). Systemic and topical corticosteroid treatment of oral lichen planus: A comparative study with long-term follow-up. Journal of Oral Pathology and Medicine, 32(6), 323–329.

Cardozo, A. L., Moura-Castro, C., Figueiredo, M., Cuzzi, T., Ramos-e-Silva, M. (2009). Oral lichen planus and dermal dendrocytes. Actas Dermo-Sifiliograficas, 100(1), 46–52.

Carli, J. P., Oliveira Da Silva, S., Salete Sandini Linden, M., Busin, C. S., Paranhos, L. R., & Henrique Couto Souza, P. (2014). Evaluation of cellular proliferative activity in patients with oral lichen planus and hepatitis c through AgNOR method. Brazilian Dental Journal, 25(6), 461–465.

Carrozzo, M., Dametto, E., Fasano, M. E., Arduino, P., Bertolusso, G., de Capei, F., Rendine, S., Amoroso, A. (2007). Cytokine gene polymorphisms in hepatitis C virus-related oral lichen planus. Experimental Dermatology, 16(9), 730–736.

Carrozzo, M., Elia, A., Mereu, V., Dametto, E., Fasano, M., Broccoletti, R., Rendine, S., & Amoroso, A. (2011). HLA-C/KIR genotypes in oral lichen planus patients infected or non-infected with hepatitis C virus. Oral Diseases, 17(3), pp. 309–313.

Carrozzo, M., Uboldi de Capei, M., Dametto, E., Fasano, M. E., Arduino, P., Broccoletti, R., Vezza, D., Rendine, S., Curtoni, E. S., & Gandolfo, S. (2004). Tumor necrosis factor-alpha and interferon-gamma polymorphisms contribute to susceptibility to oral lichen planus. Journal of Investigative Dermatology, 122(1), pp. 87–94.

Cassol-Spanemberg, J, Blanco-Carrion, A., Rodriguez-de Rivera-Campillo, M.-E., Estrugo-Devesa, A., Jane-Salas, E., Lopez-Lopez, J. (2019). Cutaneous, genital and oral lichen planus: A descriptive study of 274 patients. Medicina Oral Patologia Oral y Cirugia Bucal, 24(1), e1--e7.

Cawson, R. A. & Warin, R. P. (1968). Treatment of oral lichen planus with betamethasone. British Medical Journal, 1(5584), 86–89.

Cebeci, I., Gulsahi, A., Kamburoglu, K., Orhan, B. K., Kocyigit, P., Elhan, A., Erdem, E., & Oygur, T. (2009). Oral Lichen Planus in Turkish Patients: Prevalence and Clinical and Histopathologic Characteristics. Turkiye klinikleri tip bilimleri dergisi, 29(5), 1071–1075.

Cernea, P., Kuffer, R., & Brocheriou, C. (1971). [Epithelioma on oral lichen planus (apropos of 40 new cases)]. Actualites Odonto-Stomatologiques, 96, 473–490.

Chainani-Wu, N., Collins, K., & Silverman, S., Jr. (2012) ‘Use of curcuminoids in a cohort of patients with oral lichen planus, an autoimmune disease’, Phytomedicine, 19(5), pp. 418–423.

Chainani-Wu, N., Lozada-Nur, F., & Silverman, S. (2000). Oral lichen planus patient profile, disease progression and treatment responses. Journal of dental research, 79, 377.

Chaiyarit, P., Jintakanon, D., Klanrit, P., Siritapetawee, M., Thongprasom, K. (2009). Immunohistochemical analyses of survivin and heat shock protein 90 expression in patients with oral lichen planus. Journal of Oral Pathology and Medicine, 38(1), 55–62.

Chaiyarit, P., Klanrit, P., Phothipakdee, P., Subarnbhesaj, A., Thongprasom, K., & Giraud, A. S. (2014). Trefoil factor expression by immunohistochemistry in patients with oral lichen planus. Asian biomedicine, 8(6), 743–749.

Chaiyarit, P., Taweechaisupapong, S., Jaresitthikunchai, J., Phaonakrop, N., Roytrakul, S. (2015). Comparative evaluation of 5-15-kDa salivary proteins from patients with

different oral diseases by MALDI-TOF/TOF mass spectrometry. Clinical Oral Investigations, 19(3), 729–737.

Challacombe, S. J. (1986) ‘Haematological abnormalities in oral lichen planus, candidiasis, leukoplakia and non-specific stomatitis.’, International journal of oral and maxillofacial surgery, 15(1), pp. 72–80.

Chandrakala, J., Vidya, M., Hemavathy, S., Srinath, S., Suresh, T., & Yadav, T. S. (2017). Estimation of silver nucleolar organizer regions in oral lichen planus, oral lichenoid reactions and oral lichenoid dysplasia. Journal of Oral and Maxillofacial Pathology, 21(3), 454.

Chang, Julia Y-F, Chen, I.-C., Wang, Y.-P., Wu, Y.-H., Chen, H.-M., & Sun, A. (2016). Anemia and hematinic deficiencies in gastric parietal cell antibody-positive and antibody-negative erosive oral lichen planus patients with thyroid antibody positivity. Journal of the Formosan Medical Association = Taiwan Yi Zhi, 115(11), 1004–1011.

Chang, Julia Yu-Fong, Chiang, C.-P., Hsiao, C. K., Sun, A. (2009). Significantly higher frequencies of presence of serum autoantibodies in Chinese patients with oral lichen planus. Journal of Oral Pathology and Medicine, 38(1), 48–54.

Chang, Julia Yu-Fong, Wang, Y.-P., Wu, Y.-C., Wu, Y.-H., Tseng, C.-H., & Sun, A. (2016). Hematinic deficiencies and anemia statuses in antigastric parietal cell antibody-positive erosive oral lichen planus patients with desquamative gingivitis. Journal of the Formosan Medical Association = Taiwan Yi Zhi, 115(10), 860–866.

Chang, Julia Yu-Fong, Wang, Y.-P., Wu, Y.-H., Su, Y.-X., Tu, Y.-K., & Sun, A. (2018). Hematinic deficiencies and anemia statuses in anti-gastric parietal cell antibody-positive or all autoantibodies-negative erosive oral lichen planus patients. Journal of the Formosan Medical Association = Taiwan Yi Zhi, 117(3), 227–234.

Chankong, T., Chotjumlong, P., Sastraruji, T., Pongsiriwet, S., Iamaroon, A., & Krisanaprakornkit, S. (2016). Increased cyclooxygenase 2 expression in association with oral lichen planus severity. Journal of Dental Sciences, 11(3), 238–244.

Chatterjee, K., Bhattacharya, S., Mukherjee, C. G., & Mazumdar, A. (2012). A retrospective study of oral lichen planus in paediatric population. Journal of Oral and Maxillofacial Pathology, 16(3), 363–367.

Chaudhary, S. (2004) ‘Psychosocial stressors in oral lichen planus’, Australian Dental Journal, 49(4), pp. 192–195.

Chauhan, I., Beena, V. T., Srinivas, L., Sathyan, S., Banerjee, M. (2013). Association of cytokine gene polymorphisms with oral lichen Planus in Malayalam-speaking ethnicity from South India (Kerala). Journal of Interferon and Cytokine Research, 33(8), 420–427.

Chen H. R. (1989), "A newly developed method for treatment of oral lichen planus with ultraviolet irradiation.", Taiwan yi xue hui za zhi. Journal of the Formosan Medical Association. Vol. 88(3), pp. 248-252.

Chen Y, Li H-B, Zhang Y-H, Chen H-Z and Chen Y (2015), "Expression of human DNA mismatch-repair protein, hMSH2, In patients with oral lichen planus", Experimental and Therapeutic Medicine. Vol. 9(1), pp. 203-206.

Chen, H. M., Wang, Y. P., Chang, J. Y., Wu, Y. C., Cheng, S. J., & Sun, A. (2015), "Significant association of deficiencies of hemoglobin, iron, folic acid, and vitamin B12 and high homocysteine level with oral lichen planus", Journal of the Formosan Medical Association. Vol. 114(2), pp. 124-129.

Chen, H. X., Blasiak, R., Kim, E., Padilla, R., Culton, D. A. (2017). Triggers of oral lichen planus flares and the potential role of trigger avoidance in disease management. Oral Surgery, Oral Medicine, Oral Pathology and Oral Radiology, 124(3), 248–252.

Chen, J., Du, G., Chang, Y., Wang, Y., Shi, L., Mi, J., Tang, G. (2019). Downregulated miR-27b promotes keratinocyte proliferation by targeting PLK2 in oral lichen planus. Journal of Oral Pathology and Medicine, 48(4), 326–334.

Chen, J., Du, G., Wang, Y., Shi, L., Mi, J., & Tang, G. (2017) ‘Integrative analysis of mRNA and miRNA expression profiles in oral lichen planus: preliminary results’, Oral surgery, oral medicine, oral pathology and oral radiology, 124(4), pp. 390–402.

Chen, J., Feng, J., Chen, X., Xu, H., Zhou, Z., Shen, X., Bao, Z., Liu, W., & Shen, Z. (2013) ‘Immunoexpression of interleukin-22 and interleukin-23 in oral and cutaneous lichen planus lesions: A preliminary study’, Mediators of Inflammation, 2013, p. 801974.

Chen, J., Wang, Y. Y., Du, G., Zhang, W., Cao, T., Shi, L., Wang, Y. Y., Mi, J., Tang, G. (2019). Down-regulation of miRNA-27b-3p suppresses keratinocytes apoptosis in oral lichen planus. Journal of Cellular and Molecular Medicine, 23(6), 4326–4337.

Chen, Z. L. (1987) ‘Preliminary study on cytogenetics in patients with oral lichen planus’, Zhonghua kou qiang yi xue za zhi = Zhonghua kouqiang yixue zazhi = Chinese journal of stomatology, 22(5), pp. 279-282,310-311.

Chen, Z.-L., Deng, G.-H., Chen, H.-B., Cheng, J. (2008). [Expression of TGF-beta1, Smad7 and cell apoptosis in epithelium of oral lichen planus]. Zhonghua Kou Qiang Yi Xue Za Zhi = Zhonghua Kouqiang Yixue Zazhi = Chinese Journal of Stomatology, 43(2), 95–98.

Cheng, B., Rhodus, N. L., Williams, B., & Griffin, R. J. (2004). Detection of apoptotic cells in whole saliva of patients with oral premalignant and malignant lesions: a preliminary study. Oral Surgery, Oral Medicine, Oral Pathology, Oral Radiology, and Endodontics, 97(4), 465–470.

Chher, T., Hak, S., Kallarakkal, T. G., Durward, C., Ramanathan, A., Ghani, W. M. N., Razak, I. A., Harun, M. H., Ashar, N. A. M., Rajandram, R. K., Prak, P., Hussaini, H. M., & Zain, R. B. (2018). Prevalence of oral cancer, oral potentially malignant disorders and other oral mucosal lesions in Cambodia. Ethnicity & Health, 23(1), 1–15.

Chiappelli, F., Alwan, J., Prolo, P., Christensen, R., Fiala, M., Cajulis, O. S., & Bernard, G. (2005). Neuro-immunity in stress-related oral ulcerations: A fractal analysis. Frontiers in Bioscience, 10, 3034–3041.

Chiappelli, F., Kung, M. A., Nguyen, P., Villanueva, P., Farhadian, E. A., Eversole, L. R. (1997). Cellular immune correlates of clinical severity in oral lichen planus: Preliminary association with mood states. Oral Diseases, 3(2), 64–70.

Chitturi, R. T., Nirmal, R. M., Sunil, P. M., Devy, A. S., & Reddy, B. V. R. (2014). Evaluation of ploidy status using DNA-image cytometry of exfoliated mucosal cells in oral lichen planus. Journal of Cytology, 31(3), 131–135.

Chitturi, R. T., Sindhuja, P., Parameswar, R. A., Nirmal, R. M., Reddy, B. V. R., Dineshshankar, J., Yoithapprabhunath, T. R. (2015). A clinical study on oral lichen planus with special emphasis on hyperpigmentation. Journal of Pharmacy and Bioallied Sciences, 7(6), S495--S498.

Chiu, C.-T., Chuang, C.-Y., Li, J.-R., Huang, H.-Y., Chang, S.-W., & Hung, Y.-C. (2010). Greater therapeutic efficacy of prednisolone plus medicinal herbs than prednisolone or medicinal herbs alone in patients with oral lichen planus. Journal of dental sciences, 5(4), 209–215.

Choonhakarn, C., Busaracome, P., Sripanidkulchai, B., & Sarakarn, P. (2008). The efficacy of aloe vera gel in the treatment of oral lichen planus: A randomized controlled trial. British Journal of Dermatology, 158(3), 573–577.

Chou, M. J., Daniels, T. E. (1989). Langerhans cells expressing HLA-DQ, HLA-DR and T6 antigens in normal oral mucosa and lichen planus. Journal of Oral Pathology and Medicine, 18(10), 573–576.

Christensen, E., Holmstrup, P., Wiberg-Jorgensen, F., Neumann-Jensen, B., & Pindborg, J. J. (1977). Arterial blood pressure in patients with oral lichen planus. Journal of Oral Pathology, 6(3), 139–142.

Chruściel H., Szymczykowa B., Smigla K., Koryci'nska-Wro'nska W. and Jedy'nska-Markiewicz B. (1979), "Incidence and treatment of oral lichen planus", Czasopismo stomatologiczne. Vol. 32(6), pp. 541-548.

Chung, C.-H., Yang, Y.-H., Wang, T.-Y., Shieh, T.-Y., & Warnakulasuriya, S. (2005). Oral precancerous disorders associated with areca quid chewing, smoking, and alcohol drinking in southern Taiwan. Journal of Oral Pathology & Medicine: Official Publication of the International Association of Oral Pathologists and the American Academy of Oral Pathology, 34(8), 460–466.

Chuykin, S. V, Akmalova, G. M., Mirsayapova, I. A., Ron, G. I., Chernysheva, N. D., Khairullina, R. M. (2019). Features of immune status in patients with various clinical forms of oral lichen planus. infektsiya i immunitet, 9(1), 128–134.

Cigic, L., Gavic, L., Simunic, M., Ardalic, Z., Biocina-Lukenda, D. (2015). Increased prevalence of celiac disease in patients with oral lichen planus. Clinical Oral Investigations, 19(3), 627–635.

Cilurzo, F., Gennari, C. G., Selmin, F., Epstein, J. B., Gaeta, G. M., Colella, G., & Minghetti, P. (2010). A new mucoadhesive dosage form for the management of oral lichen planus: Formulation study and clinical study. European Journal of Pharmaceutics and Biopharmaceutics, 76(3), 437–442.

Conrotto, D., Barattero, R., Carbone, M., Gambino, A., Sciannameo, V., Ricceri, F., Conrotto, F., Broccoletti, R., & Arduino, P.-G. (2018). Can atrophic-erosive oral lichen planus promote cardiovascular diseases? A population-based study. Oral Diseases, 24(1–2), 215–218.

Cooper, S. M., Dean, D., Allen, J., Kirtschig, G., & Wojnarowska, F. (2005). Erosive lichen planus of the vulva: weak circulating basement membrane zone antibodies are present. Clinical and Experimental Dermatology, 30(5), 551–556.

Corrocher, G., Di Lorenzo, G., Martinelli, N., Mansueto, P., Biasi, D., Nocini, P. F., Lombardo, G., Pacor, M. L. (2008). Comparative effect of tacrolimus 0.1% ointment and clobetasol 0.05% ointment in patients with oral lichen planus. Journal of Clinical Periodontology, 35(3), 244–249.

Cortés-Ramírez, D. A., Rodrguez-Tojo, M. J., Gainza-Cirauqui, M. L., Martnez-Conde, R., & Aguirre-Urizar, J. M. (2010). Overexpression of cyclooxygenase-2 as a biomarker in different subtypes of the oral lichenoid disease. Oral Surgery, Oral Medicine, Oral Pathology, Oral Radiology and Endodontology, 110(6), 738–743.

Cortés-Ramírez, D.-A., Rodríguez-Tojo, M.-J., Coca-Meneses, J.-C. J.-C., Marichalar-Mendia, X., Aguirre-Urizar, J.-M. J.-M. (2014). Epidermal growth factor receptor expression in different subtypes of oral lichenoid disease. Medicina Oral, Patologia Oral y Cirugia Bucal, 19(5), e451--e458.

Cozzani, E., Russo, R., Mazzola, F., Garofolo, S., Camerino, M., Burlando, M., Peretti, G., & Parodi, A. (2019). Narrow-band imaging: a useful tool for early recognition of oral lichen planus malignant transformation? European Journal of Dermatology.

Cruz, A. F., de Resende, R. G., de Lacerda, J. C. T., Pereira, N. B., Melo, L. A., Diniz, M. G., Gomes, C. C., Gomez, R. S. (2018). DNA methylation patterns of genes related to immune response in the different clinical forms of oral lichen planus. Journal of Oral Pathology and Medicine, 47(1), 91–95.

Cruz, A. F., Vitorio, J. G., Duarte-Andrade, F. F., Diniz, M. G., Canuto, G. A. B., de Toledo, J. S., Fonseca, F. P., Fernandes, A. P., Gomez, R. S. (2019). Reticular and erosive oral lichen planus have a distinct metabolomic profile: A preliminary study using gas chromatography-mass spectrometry. Journal of Oral Pathology and Medicine, 48(5), 400–405.

Czerninski, R., Zadik, Y., Kartin-Gabbay, T., Zini, A., & Touger-Decker, R. (2014). Dietary alterations in patients with oral vesiculoulcerative diseases. Oral Surgery, Oral Medicine, Oral Pathology and Oral Radiology, 117(3), 319–323.

Czerninski, R., Zeituni, S., Maly, A., & Basile, J. (2015). Clinical characteristics of lichen and dysplasia vs lichen planus cases and dysplasia cases. Oral Diseases, 21(4), 478–482.

Daftary, D. K., Bhonsle, R. B., Murti, R. B., Pindborg, J. J., & Mehta, F. S. (1980). An oral lichen planus-like lesion in Indian betel-tobacco chewers. Scandinavian Journal of Dental Research, 88(3), 244–249.

Dalirsani, Z., Taghavi Zenouz, A., Mehdipour, M., Alavi, F., & Javadzadeh, Y. (2010). Comparison of the effect of combination of triamcinolone acetonide and vitamin a

mouthwash with triamcinolone mouthwash alone on oral lichen planus. Journal of Dental Research, Dental Clinics, Dental Prospects, 4(1), 21–24.

Dan, H., Liu, W., Wang, J., Wang, Z., Wu, R., Chen, Q., Zeng, X., Zhou, Y. (2011). Elevated IL-10 concentrations in serum and saliva from patients with oral lichen planus. Quintessence International (Berlin, Germany : 1985), 42(2), 157–163.

Dan, H., Liu, W., Zhou, Y., Wang, J., Chen, Q., Zeng, X. (2010). Association of interleukin-8 gene polymorphisms and haplotypes with oral lichen planus in a Chinese population. Inflammation, 33(2), 76–81.

Dang, J., Bian, Y.-Q., Sun, J. Y., Chen, F., Dong, G.-Y., Liu, Q., Wang, X.-W., Kjems, J., Gao, S., Wang, Q.-T. (2013). MicroRNA-137 promoter methylation in oral lichen planus and oral squamous cell carcinoma. Journal of Oral Pathology and Medicine, 42(4), 315–321.

Dangore-Khasbage, S., Khairkar, P. H., Degwekar, S. S., Bhowate, R. R., Bhake, A. S., Singh, A., Lohe, V. K. (2012). Prevalence of oral mucosal disorders in institutionalized and non-institutionalized psychiatric patients: a study from AVBR Hospital in central India. Journal of Oral Science, 54(1), 85–91.

Danielsson, K, Boldrup, L., Rentoft, M., Coates, P. J., Ebrahimi, M., Nylander, E., Wahlin, Y. B., Nylander, K.(2013). Autoantibodies and decreased expression of the transcription factor ELF-3 together with increased chemokine pathways support an autoimmune phenotype and altered differentiation in lichen planus located in oral mucosa. Journal of the European Academy of Dermatology and Venereology, 27(11), 1410–1416.

Danielsson, K, Ebrahimi, M., Wahlin, Y. B., Nylander, K., Boldrup, L. (2012). Increased levels of COX-2 in oral lichen planus supports an autoimmune cause of the disease. Journal of the European Academy of Dermatology and Venereology, 26(11), 1415–1419.

Danielsson, K, Olah, J., Zohori-Zangeneh, R., Nylander, E., & Ebrahimi, M. (2018). Increased expression of p16 in both oral and genital lichen planus. Medicina Oral, Patologia Oral y Cirugia Bucal, 23(4), e449--e453.

Danielsson, K, Wahlin, Y. B., Coates, P. J., Nylander, K. (2010). Increased expression of Smad proteins, and in particular Smad3, in oral lichen planus compared to normal oral mucosa. Journal of Oral Pathology and Medicine, 39(8), 639–644.

Danielsson, K, Wahlin, Y. B., Gu, X., Boldrup, L., Nylander, K. (2012). Altered expression of miR-21, miR-125b, and miR-203 indicates a role for these microRNAs in oral lichen planus. Journal of Oral Pathology and Medicine, 41(1), 90–95.

Danielsson, Karin, Ebrahimi, M., Nylander, E., Wahlin, Y. B., & Nylander, K. (2017). Alterations in Factors Involved in Differentiation and Barrier Function in the Epithelium in Oral and Genital Lichen Planus. Acta Dermato-Venereologica, 97(2), 214–218.

Darczuk, D., Krzyściak, W., Bystrowska, B., Kęsek, B., Kościelniak, D., Chomyszyn-Gajewska, M., & Kaczmarzyk, T. (2019). The Relationship between the Concentration of Salivary Tyrosine and Antioxidants in Patients with Oral Lichen Planus. Oxidative Medicine and Cellular Longevity, 2019, 5801570.

Darczuk, D., Krzysciak, W., Vyhouskaya, P., Kesek, B., Galecka-Wanatowicz, D., Lipska, W., Kaczmarzyk, T., Gluch-Lutwin, M., Mordyl, B., & Chomyszyn-Gajewska, M. (2016). Salivary oxidative status in patients with oral lichen planus. Journal of Physiology and Pharmacology, 67(6), 885–894.

Dave, A., Shariff, J., & Philipone, E. (2020). Association between oral lichen planus and systemic conditions and medications: Case-control study. Oral Diseases, 27(3), 515–524.

Davidopoulou, S., Theodoridis, H., Nazer, K., Kessopoulou, E., Menexes, G., Kalfas, S. (2014). Salivary concentration of the antimicrobial peptide LL-37 in patients with oral lichen planus. Journal of Oral Microbiology, 6(1), 1–6.

de Barros Gallo, C., Marichalar-Mendia, X., Setien-Olarra, A., Acha-Sagredo, A., Bediaga, N. G., Gainza-Cirauqui, M. L., Sugaya, N. N., Aguirre-Urizar, J. M. (2017). Toll-like receptor 2 rs4696480 polymorphism and risk of oral cancer and oral potentially malignant disorder. Archives of Oral Biology, 82, 109–114.

de Blanc, S., Gendelman, H., Itoiz, M. E., Lanfranchi, H. (1996). Study of the vascular pattern in oral lichen planus. Acta Odontológica Latinoamericana, 9(1), 27–36.

de Brito Monteiro, B. V., dos Santos Pereira, J., Nonaka, C. F. W., Godoy, G. P., da Silveira, E. J. D., da Costa Miguel, M. C. (2015). Immunoexpression of Th17-related Cytokines in Oral Lichen Planus. Applied Immunohistochemistry and Molecular Morphology, 23(6), 409–415.

de Camargo, A. R., Tenorio, J. R., Martins, F., Grando, L. J., Correa, E. B. D., Trierveiler, M., Ortega, K. L. (2019). Subset of CD8+ and FOXP3 + T cells in lichen planus associated with chronic hepatitis C infection. Oral Diseases, 25(4), 1100–1106.

de Carvalho Fraga, C. A., Alves, L. R., Marques-Silva, L., de Sousa, A. A., Jorge, A. S. B., de Jesus, S. F., Vilela, D. N., Pinheiro, U. B., Jones, K. M., de Paula, A. M. B., & Guimaraes, A. L. S. (2013). High HIF-1alpha expression genotypes in oral lichen planus. Clinical Oral Investigations, 17(9), 2011–2015.

de Carvalho, C. H., dos Santos, B. R., Vieira, C. de C., de Araujo Lima, E. das N., de Andrade Santos, P. P., & Freitas, R. de A. (2011). An epidemiological study of immune-mediated skin diseases affecting the oral cavity. Anais Brasileiros de Dermatologia, 86(5), 905–909.

de Gutierrez, E., Di Fabio, A., Salomon, S., & Lanfranchi, H. (2014). Topical treatment of oral lichen planus with anthocyanins. Medicina Oral, Patologia Oral y Cirugia Bucal, 19(5), E459--E466.

de Gutierrez, E., Innocenti, A., Cippitelli, M., Salomon, S., Vargas-Roig, L. (2014). Determination of cytokeratins 1, 13 and 14 in oral lichen planus. Medicina Oral, Patologia Oral y Cirugia Bucal, 19(4), e359--e365.

de Lima S. L., de Arruda J. A. (2019). Clinicopathologic data of individuals with oral lichen planus: A Brazilian case series. Journal of Clinical and Experimental Dentistry, 11(12), e1109–e1119.

de Sousa, F. A. C. G., Paradella, T. C., Carvalho, Y. R., & Rosa, L. E. B. (2009). Immunohistochemical expression of PCNA, p53, bax and bcl-2 in oral lichen planus and epithelial dysplasia. Journal of Oral Science, 51(1), 117–121.

de Sousa, F. A. C. G., Paradella, T. C., Carvalho, Y. R., Rosa, L.E.B.(2009). Comparative analysis of cell proliferation ratio in oral lichen planus, epithelial dysplasia and oral squamous cell carcinoma. Medicina Oral, Patologia Oral y Cirugia Bucal, 14(11), e563--e567.

De Sousa, F.A.C.G., Paradella, T. C., Brandão, A. A. H., Rosa, L. E. B. (2009). Comparative study of cell alterations in oral lichen planus and epidermoid carcinoma of the mouth mucosa. Brazilian Journal of Otorhinolaryngology, 75(2), 245–248.

de Sousa, Fernando Augusto Cervantes Garcia, Paradella, T. C., Brandao, A. A. H., Rosa, L. E. B. (2009). Oral lichen planus versus epithelial dysplasia: Difficulties in diagnosis. Brazilian Journal of Otorhinolaryngology, 75(5), 716–720.

de Sousa, Fernando Augusto Cervantes Garcia, Paradella, T. C., Carvalho, Y. R., Rosa, L. E. B. (2009). Comparative analysis of the expression of proliferating cell nuclear antigen, p53, bax, and bcl-2 in oral lichen planus and oral squamous cell carcinoma. Annals of Diagnostic Pathology, 13(5), 308–312.

De, D., Arora, A. K., Handa, S., Chatterjee, D., Saikia, U. N., Radotra, B. D., Kishore, K., & Khullar, G. (2020). Clinical and pathological characterization of oral mucosal “lichen planus-like lesions” in patients with pemphigus vulgaris: An observational study. Indian Journal of Dermatology, Venereology and Leprology, 86(3), 278–283.

Deganello, A., Paderno, A., Morello, R., Fior, M., Berretti, G., Del Bon, F., Alparone, M., Bardellini, E., Majorana, A., & Nicolai, P. (2021). Diagnostic Accuracy of Narrow Band Imaging in Patients with Oral Lichen Planus: A Prospective Study. The Laryngoscope, 131(4), E1156–E1161.

Dejong, W. F. B., Albrecht, M., Banoczy, J., & Vander Waal, I. (1984). Epithelial dysplasia in oral lichen planus - a preliminary-report of a dutch-hungarian study of 100 cases. International journal of oral surgery, 13(3), 221–225.

del Olmo, J. A., Pascual, I., Bagan, J. V, Serra, M. A., Escudero, A., Rodriguez, F., & Rodrigo, J. M. (2000). Prevalence of hepatitis C virus in patients with lichen planus of the oral cavity and chronic liver disease. European Journal of Oral Sciences, 108(5), 378–382.

Delavarian, Z., Javadzadeh-Bolouri, A., Dalirsani, Z., Arshadi, H.-R., Toofani-Asl, H. (2010). The evaluation of psychiatric drug therapy on oral lichen planus patients with psychiatric disorders. Medicina Oral, Patologia Oral y Cirugia Bucal, 15(2), e322--e327.

Devi, M., Vijayalakshmi, D., Dhivya, K., Janane, M. (2017). Memory T cells (CD45RO) role and evaluation in pathogenesis of lichen planus and lichenoid mucositis. Journal of Clinical and Diagnostic Research, 11(5), ZC84--ZC86.

Deyhimi, P., & Arzhang, E. (2018). Study of extrinsic apoptotic pathway in oral Lichen Planus using TNFR 1 and FasL immunohistochemical markers and TUNEL technique. Journal of oral and maxillofacial surgery medicine and pathology, 30(4), 380–385.

Di Fede, O., Belfiore, P., Cabibi, D., De Cantis, S., Maresi, E., Kerr, A. R., Campisi, G. (2006). Unexpectedly high frequency of genital involvement in women with clinical and histological features of oral lichen planus. Acta Dermato-Venereologica, 86(5), 433–438.

Dillenburg, C. S., Martins, M. A. T. M. D., Munerato, M. C., Marques, M. M., Carrard, V. C., Sant'Ana Filho, M., Castilho, R. M., & Martins, M. (2014). Efficacy of laser phototherapy in comparison to topical clobetasol for the treatment of oral lichen planus: a randomized controlled trial. Journal of Biomedical Optics, 19(6), 68002.

Dillenburg, C. S., Martins, M. D. M. A. T., Almeida, L. O., Meurer, L., Squarize, C. H., Martins, M. D. M. A. T., Castilho, R. M. (2015). Epigenetic modifications and accumulation of DNA double-strand breaks in oral lichen planus lesions presenting poor response to therapy. Medicine (United States), 94(30), e997–e997.

Ding, M., Xu, J. Y., & Fan, Y. (2010). Altered expression of mRNA for HIF-1alpha and its target genes RTP801 and VEGF in patients with oral lichen planus. Oral Diseases, 16(3), 299–304.

Ding, M., Xu, J. Y., Fan, Y. (2010). Altered expression of mRNA for HIF-1α and its target genes RTP801and VEGF in patients with oral lichen planus. Oral Diseases, 16(3), 299–304.

Ding, M., Zeng, J., Sroussi, H., Yu, J., Xu, J., Cheng, X., Fan, Y. (2014). Interactions between Golli-MBP and Th1/Th2 cytokines in patients with oral lichen planus. Oral Diseases, 20(2), 205–211.

Diop, A et al. (2020). Epidemiology, clinical features, and associated factors in 78 cases of lichen planus on black skin. International Journal of Dermatology, 59(2), 137–142.

Ditrichova, D., Kapralova, S., Tichy, M., Ticha, V., Dobesova, J., Justova, E., Eber, M., & Pirek, P. (2007). Oral lichenoid lesions and allergy to dental materials. Biomedical Papers of the Medical Faculty of the University Palacky, Olomouc, Czechoslovakia, 151(2), 333–339.

Divya, V. C., & Sathasivasubramanian, S. (2014). Estimation of serum and salivary immunoglobulin G and immunoglobulin A in oral pre-cancer: a study in oral submucous fibrosis and oral lichen planus. Journal of Natural Science, Biology, and Medicine, 5(1), 90–94.

do Carmo, M. A. V., Gleber-Netto, F. O., Romano, M. L. de F., Caldeira, P. C., & de Aguiar, M. C. F. (2014). Clinical and demographic overlaps among immunologically mediated oral diseases: a challenge for clinicians. General Dentistry, 62(1), 67–72.

Doddawad, V. G. (2014). Histopathological analysis of apoptotic cell count and its role in oral lichen planus. Journal of Oral and Maxillofacial Pathology : JOMFP, 18(1), 42–45.

Dombi, C., Voros-Balog, T., Czegledy, A., Hermann, P., Vincze, N., Banoczy, J. (2001). Risk group assessment of oral precancer attached to X-ray lung-screening examinations. Community Dentistry and Oral Epidemiology, 29(1), 9–13.

Drogoszewska, B., Chomik, P., Polcyn, A., Michcik, A. (2014). Clinical diagnosis of oral erosive lichen planus by direct oral microscopy. Postepy Dermatologii i Alergologii, 31(4), 222–228.

Družijanić, A et al. (2019). Inflammatory Markers and Incidence of other Autoimmune Diseases in Patients with Oral Lichen Planus. Acta stomatologica croatica, 53(4), 363–370.

Du, G.-H., Qin, X.-P., Li, Q., Zhou, Y.-M., Shen, X.-M., Tang, G.-Y. (2011). The high expression level of programmed death-1 ligand 2 in oral lichen planus and the possible costimulatory effect on human T cells. Journal of Oral Pathology and Medicine, 40(7), 525–532.

Du, G., Chen, J., Wang, Y., Cao, T., Zhou, L., Wang, Y., Han, X., & Tang, G. (2018). Differential expression of STAT-3 in subtypes of oral lichen planus: a preliminary study. Oral Surgery, Oral Medicine, Oral Pathology and Oral Radiology, 125(3), 236--243.e1.

Du, G., Chen, J., Wang, Y., Cao, T., Zhou, L., Wang, Y., Han, X., & Tang, G. (2018). Differential expression of STAT-3 in subtypes of oral lichen planus: a preliminary study. Oral Surgery, Oral Medicine, Oral Pathology and Oral Radiology, 125(3), 236–243.

Du, J., Li, R., Yu, F., Yang, F., Wang, J., Chen, Q., Wang, X., Zhao, B., & Zhang, F. (2017). Experimental study on 1,25$(OH)_2$D3 amelioration of oral lichen planus through regulating NF-kappaB signaling pathway. Oral Diseases, 23(6), 770–778.

Dunsche, A., Kastel, I., Terheyden, H., Springer, I. N. G., Christophers, E., & Brasch, J. (2003). Oral lichenoid reactions associated with amalgam: Improvement after amalgam removal. British Journal of Dermatology, 148(1), 70–76.

Dvorak, G., Monshi, B., Hof, M., Bernhart, T., Bruckmann, C., & Rappersberger, K. (2015). Gender aspects in oral health-related quality of life of oral lichen planus patients. International journal of stomatology & occlusion medicine, 8(2), 33–40.

E, A., L, P., & MH, A. (2019). Histopathological features of oral lichen planus and its response to corticosteroid therapy: A retrospective study. Medicine, 98(51), e18321.

Ebner H, Mischer P and Raff M (1973), "Local treatment of lichen ruber planus of the oral mucosa with retinoic acid", H+G Zeitschrift fur Hautkrankheiten. II. Univ. Hautklin., Wien Vol. 48(18), pp. 735-740.

Ebrahimi, H., Pakshir, K., Pourshahidi, S., Zomorodian, K., Saki, M., Saki, N., Valizadeh, M., & Kardeh, S. (2014). Prevalence of Common Candida Species in Oral Lichen Planus Patients: A Cross-Sectional Study in South of Iran. Galen Medical Journal, 3(4), 252–255.

Ebrahimi, M., Boldrup, L., Coates, P. J., Wahlin, Y. B., Bourdon, J. C., Nylander, K. (2008). Expression of novel p53 isoforms in oral lichen planus. Oral Oncology, 44(2), 156–161.

Ebrahimi, M., Boldrup, L., Wahlin, Y.-B., Coates, P. J., Nylander, K. (2008). Decreased expression of the p63 related proteins $β$-catenin, E-cadherin and EGFR in oral lichen planus. Oral Oncology, 44(7), 634–638.

Ebrahimi, M., Wahlin, Y.-B., Coates, P. J., Sjostrom, B., Nylander, K. (2006). Decreased expression of p63 in oral lichen planus and graft-vs.-host disease associated with oral inflammation. Journal of Oral Pathology and Medicine, 35(1), 46–50.

Ebrahimzadeh, M., Dehghani, F., Amirniroumand, N. (2014). High frequency of genital involvement in lichen planus: A cross sectional study. Iranian Journal of Dermatology, 17(70), 130–133.

Eisen, D. (1993). Hydroxychloroquine sulfate (Plaquenil) improves oral lichen planus: An open trial. Journal of the American Academy of Dermatology, 28(4), 609–612.

Eisen, D. (1994). The vulvo-vaginal-gingival-syndrome of lichen-planus - The clinical characteristics of 22 patients. Archives of dermatology, 130(11), 1379–1382.

Eisen, D. (1999). The evaluation of cutaneous, genital, scalp, nail, esophageal, and ocular involvement in patients with oral lichen planus. Oral Surgery, Oral Medicine, Oral Pathology, Oral Radiology, and Endodontics, 88(4), 431–436.

Eisen, D., Ellis, C. N., Duell, E. A., Griffiths, C. E., Voorhees, J. J. (1990). Effect of topical cyclosporine rinse on oral lichen planus. A double-blind analysis. New England Journal of Medicine, 323(5), 290–294.

El-Labban, N. G. (1977). An ultrastructural study of Langerhans cells crossing the basal complex in oral lichen planus. Archives of oral biology, 22(10), 629–631.

El-Labban, N. G., Kramer, I. R. (1975). Light and electron microscopic study of liquefaction degeneration in oral lichen planus. Archives of Oral Biology, 20(10), 653–657.

El-Zefzaf, E., Abd-el-Razzak, M., Ghoneim, S., Abu-Azma, N. (1994). Demonstration of T-cell subsets in oral lichen planus. Egyptian Dental Journal, 40(1), 633–638.

Elia, A., Della Ferrera, F., Carbone, M., Arduino, P. G., Gambino, A., Nole, E., & Broccoletti, R. (2012). Pimecrolimus vs tacrolimus in unresponsive oral lichen planus: a randomized controlled trial. Oral diseases, 18(1), 14–15.

Elshenawy, H. M., Eldin, A. M., Abdelmonem, M. A. (2015). Clinical assessment of the efficiency of low level laser therapy in the treatment of oral lichen planus. Macedonian Journal of Medical Sciences, 3(4), 717–721.

Eltatawy, R. A. R., Hassan, G. F. R., Qandeel, A. M. A., & Mohammed, D. A. (2019). Immunohistochemical study of Wnt5a expression in cutaneous and oral lichen planus. Journal of the Egyptian Women’s Dermatologic Society, 16(2), 81–88.

Enomoto, A., Sato, E., Yasuda, T., Isomura, T., Nagao, T., Chikazu, D. (2018). Intraepithelial CD8+ lymphocytes as a predictive diagnostic biomarker for the remission of oral lichen planus. Human Pathology, 74, 43–53.

Enomoto, Y., Suzuki, H., Kimoto, A., Asai, T., Takeuchi, J., Matsumoto, K., & Komori, T. (2016). Concordance between clinical and histopathological diagnoses of oral lichen planus. Journal of oral and maxillofacial surgery medicine and pathology, 28(5), 381–384.

Ertugrul, A. S., Dursun, R., Dundar, N., Avunduk, M. C., Hakki, S. S. (2013). MMP-1, MMP-9, and TIMP-1 levels in oral lichen planus patients with gingivitis or periodontitis. Archives of Oral Biology, 58(7), 843–852.

Eslami, H., Pakdel, F., Babaloo, Z., Pouralibaba, F., Falsafi, P., Neghad, S. K., Pakdel, M. V, & Fakhrzadeh, V. (2016). Evaluation of serum IL-1B and IL-8 in patients with Oral Lichen Planus. International Journal of Clinical Dentistry, 9(4), 241–245.

Ezzatt, O. M., Helmy, I. M. (2019). Topical pimecrolimus versus betamethasone for oral lichen planus: a randomized clinical trial. Clinical Oral Investigations, 23(2), 947–956.

F., R., F., G., G., L., G.M., G., C., E., R., S., C., D. N., A., M., & P., M. (1993). Malignant transformation of oral lichen planus by Candida albicans. International Journal of Immunopathology and Pharmacology, 6(2), 125–134.

Faedler, A., Hartmann, T., Bernhart, T., Monshi, B., Rappersberger, K., Hof, M., Dvorak, G. (2015). Effect of personality traits on the oral health-related quality of life in patients with oral mucosal disease. Clinical Oral Investigations, 19(6), 1245–1250.

Fahimeh, R., Neshat, A., & Jannan, G. (2013). Oral lichen planus resistency to topical corticosteroid therapy. Middle East Journal of Scientific Research, 16(9), 1245–1248.

Falsafi, P., Khorshidi-Khiavi, R., Ghanizadeh, M., Rezaei, F., Dolatkhah, H., Bahramian, A., & Pirayesh, T. (2019). Salivary Transferrin Levels in Patients with Oral Lichen Planus. Pesquisa brasileira em odontopediatria e clinica integrada, 19(1).

Fan, Y., Xu, J.-Y., Liu, Q.-L. (2009). [Effect of Yupingfeng on the level of salivary epidermal growth factor in oral lichen planus]. Zhonghua Kou Qiang Yi Xue Za Zhi = Zhonghua Kouqiang Yixue Zazhi = Chinese Journal of Stomatology, 44(7), 392–394.

Fang, L., Liu, Q., He, P., Wang, X., Wang, Y., Wei, M., Chen, L. (2018). Alteration of salivary glycopatterns in oral lichen planus. Biomarkers, 23(2), 188–195.

Fang, M., Zhang, W., Chen, Y., He, Z. (2009). Malignant transformation of oral lichen planus: a retrospective study of 23 cases. Quintessence International (Berlin, Germany: 1985), 40(3), 235–242.

Fantozzi, P. J., Treister, N., Shekar, R., Woo, S.-B., & Villa, A. (2019). Intralesional triamcinolone acetonide therapy for inflammatory oral ulcers. Oral Surgery, Oral Medicine, Oral Pathology and Oral Radiology, 128(5), 485–490.

Farah, C. S., & McCullough, M. J. (2007). A pilot case control study on the efficacy of acetic acid wash and chemiluminescent illumination (ViziLite) in the visualisation of oral mucosal white lesions. Oral Oncology, 43(8), 820–824.

Farah, C. S., Simanovic, B., & Savage, N. W. (2008). Scope of practice, referral patterns and lesion occurrence of an oral medicine service in Australia. Oral Diseases, 14(4), 367–375.

Farhadi, S., Sadri, D., & Bagheri, F. (2018). Expression of junb in oral lichen planus: a preliminary study. Annals of dental specialty, 6(4), 397–401.

Farthing, P. M. et al. (1989) ‘Expression of MHC Class II antigens (HLA DR, DP and DQ) by keratinocytes in oral lichen planus’, Journal of Oral Pathology and Medicine, 18(5), pp. 305–309.

Farthing, P. M. et al. (1990) ‘The activation of langerhans cells in oral lichen planus’, Journal of Oral Pathology and Medicine, 19(2), pp. 81–85.

Farthing, P. M. et al. (1992) ‘Langerhans cell distribution and keratinocyte expression of HLADR in oral lichen planus’, Journal of Oral Pathology and Medicine, 21(10), pp. 451–455.

Farzin, M., Mardani, M., Ghabanchi, J., Fattahi, M. J., Rezaee, M., Heydari, S. T., Andisheh Tadbir, A. (2012). Serum level of matrix metalloproteinase-3 in patients with oral Lichen planus. Iranian Red Crescent Medical Journal, 14(1), 10–13.

Fathi, M. S., El Dessouky, H. F., Breni, H. A. (2013). CD4+CD25+ T regulatory cells and MMP-9 as diagnostic salivary biomarkers in oral lichen planus. The Egyptian Journal of Immunology/ Egyptian Association of Immunologists, 20(2), 39–53.

Favia, G., Corsalini, M., Iacobellis, M., & Maiorano, E. (1994). [Squamous cell carcinoma in oral lichen ruber planus. A clinico-pathological and immunohistochemical study of 11 cases]. Minerva Stomatologica, 43(10), 479–491.

Feher, E., Gall, T., Murvai, M., Kis, A., Boda, R., Sapy, T., Tar, I., Gergely, L., Szarka, K. (2009). Investigation of the occurrence of torque tenovirus in malignant and potentially malignant disorders associated with human papillomavirus. Journal of Medical Virology, 81(11), 1975–1981.

Feher, E., Kardos, G., Gall, T., Kis, A., Gergely, L., & Szarka, K. (2011). Comparison of diversity of torque teno virus 1 in different mucosal tissues and disorders. Acta Microbiologica et Immunologica Hungarica, 58(4), 319–337.

Femiano, F., & Scully, C. (2005). Functions of the cytokines in relation oral lichen planus-hepatitis C. Medicina Oral, Patología Oral y Cirugía Bucal, 10, E40--44.

Femiano, F., Scully, C. (2005). DNA cytometry of oral leukoplakia and oral lichen planus. Medicina Oral, Patología Oral y Cirugía Bucal, 10, E9--14.

Feng, J., Zhou, Z., Shen, X., Wang, Y. Y., Shi, L., Wang, Y. Y., Hu, Y., Sun, H., Liu, W. (2015). Prevalence and distribution of oral mucosal lesions: A cross-sectional study in Shanghai, China. Journal of Oral Pathology and Medicine, 44(7), 490–494.

Feng, Z., Shi, W., Cai, Z., Hua, H., & Zhou, D. (2014). Detection of microRNA expressions in tissues and exfoliative cells reveals the potential role of miR-203 in oral lichen planus. Journal of Chinese Pharmaceutical Sciences, 23(5), 279–286.

Fernandez-Gonzalez, F., Vazquez-Alvarez, R., Reboiras-Lopez, D., Gandara-Vila, P., Garcia-Garcia, A., Gandara-Rey, J. (2011). Histopathological findings in oral lichen planus and their correlation with the clinical manifestations. Medicina Oral, Patologia Oral y Cirugia Bucal, 16(5), e641--e646.

Ferri, E. P., Cunha, K., Abboud, C. S., de Barros Gallo, C., de Sousa Sobral, S., de Fatima Teixeira da Silva, D., Horliana, A., Franco, A. L., & Rodrigues, M. (2020). Photobiomodulation is effective in oral lichen planus: A randomized, controlled, double-blind study. Oral diseases. Advance online publication.

Ferri, E. P., Gallo, C. de B., Abboud, C. S., Yanaguizawa, W. H., Horliana, A. C. R. T., da Silva, D. de F. T., Pavani, C., Rodrigues, M. F. S. D. (2018). Efficacy of photobiomodulation on oral lichen planus: A protocol study for a double-blind, randomised controlled clinical trial. BMJ Open, 8(10), e024083–e024083.

Finne, K., Goransson, K., Winckler, L. (1982). Oral lichen planus and contact allergy to mercury. International Journal of Oral Surgery, 11(4), 236–239.

Firth, F. A., Friedlander, L. T., Parachuru, V. P. B., Kardos, T. B., Seymour, G. J., & Rich, A. M. (2015). Regulation of immune cells in oral lichen planus. Archives of Dermatological Research, 307(4), 333–339.

Fonseca, L. M. D., do Carmo, M. (2001). Identification of the AgNORs, PCNA and ck16 proteins in oral lichen planus lesions. Oral Diseases, 7(6), 344–348.

Fornaini, C. (2012). LLLT in the symptomatic treatment of oral Lichen Planus. Laser Therapy, 21(1), 51–53.

Fu, J., Zhu, X., Dan, H., Zhou, Y., Liu, C., Wang, F., Li, Y., Liu, N., Chen, Q., Xu, Y., Zeng, X., & Jiang, L. (2012) ‘Amlexanox is as effective as dexamethasone in topical treatment of erosive oral lichen planus: A short-term pilot study’, Oral Surgery, Oral Medicine, Oral Pathology and Oral Radiology, 113(5), pp. 638–643.

Fujita H, Kobayashi T, Tai H, Nagata M, Hoshina H, Nishizawa R, Takagi R and Yoshie H (2009), "Assessment of 14 functional gene polymorphisms in Japanese patients with oral lichen planus: a pilot case-control study", International Journal of Oral and Maxillofacial Surgery. Vol. 38(9), pp. 978-983.

Fujita, H., Nagata, M., Hoshina, H., Nagashima, K., Seki, Y., Tanaka, K., Nishizawa, R., Shingaki, S., Ohnishi, M., Takagi, R. (2004). Clinical significance and usefulness of quantification of telomerase activity in oral malignant and nonmalignant lesions. International Journal of Oral and Maxillofacial Surgery, 33(7), 693–699.

Fulling H. J. (1973), "Cancer development in oral lichen planus. A follow-up study of 327 patients.", Archives of dermatology. Vol. 108(5), pp. 667-669.

Furrer, V. E., Benitez, M. B., Furnes, M., Lanfranchi, H. E., & Modesti, N. M. (2006). Biopsy vs. superficial scraping: detection of human papillomavirus 6, 11, 16, and 18 in potentially malignant and malignant oral lesions. Journal of Oral Pathology & Medicine: Official Publication of the International Association of Oral Pathologists and the American Academy of Oral Pathology, 35(6), 338–344.

Gabriella, D., Klemens, R., Xiao-Hui, R. F., Corinna, B., & Eva, H. (2021). Effect of personality traits on the oral health-related quality of life in patients with oral lichen planus undergoing treatment. Clinical Oral Investigations, 25(4), 2381–2389.

Gambino, A., Cabras, M., Cafaro, A., Broccoletti, R., Carossa, S., Hopper, C., Chiusa, L., El Haddad, G., Porter, S. R., & Arduino, P. G. (2020). In-vivo usefulness of optical coherence tomography in atrophic-erosive oral lichen planus: Comparison between histopathological and ultrastructural findings. Journal of photochemistry and photobiology. B, Biology, 211, 112009.

Gambino, A., Carbone, M., Arduino, P.-G., Carrozzo, M., Conrotto, D., Tanteri, C., Carbone, L., Elia, A., Maragon, Z., & Broccoletti, R. (2015). Clinical features and histological description of tongue lesions in a large Northern Italian population. Medicina Oral, Patologia Oral y Cirugia Bucal, 20(5), e560--e565.

Gandara Rey, J., Garcia Garcia, A., Blanco Carrion, A., Gandara Vila, P., Rodriguez Nunez, I. (2001). Cellular immune alterations in fifty-two patients with oral lichen planus. Medicina Oral, 6(4), 246–262.

Gandara, B. K., Izutsu, K. T., Truelove, E. L., Ensign, W. Y., & Sommers, E. E. (1985). Age-related salivary flow-rate changes in controls and patients with oral lichen planus. Journal of dental research, 64(9), 1149–1151.

Gandara, B. K., Izutsu, K. T., Truelove, E. L., Mandel, I. D., Sommers, E. E., & Ensign, W. Y. (1987). Sialochemistry of whole, parotid, and labial minor gland saliva in patients with oral lichen planus. Journal of Dental Research, 66(11), 1619–1622.

García-García, V., Bascones-Martínez, A., García-Kass, A. I., Martinelli-Kläy, C. P., Küffer, R., … Lombardi, T. (2013). Analysis of the expression of heat-shock protein 27 in patients with oral lichen planus. Oral Diseases, 19(1), 65–72.

Garcia-Pola Vallejo, M. J., Anitua Roldan, M. J., Fernandez Alvarez, B. E., Garcia Martin, J. M., Lopez-Muniz, A. (2001). Study comparative of Ki-67 expression in oral lichen planus and oral leukoplakia. Quantitative analysis. Medicina Oral, 6(5), 364–370.

Garcia-Pola Vallejo, M. J., Garcia Martin, J. M., Gonzalez Garcia, M., & Telenti Arnaiz, P. (1997). [Precancerous lesions (oral leukoplakia and lichen planus) in the geriatric patient]. Atencion Primaria, 20(1), 41–44.

Garcia-Pola, M-J, Llorente-Pendas, S., Gonzalez-Garcia, M., Garcia-Martin, J.-M. (2016). The development of proliferative verrucous leukoplakia in oral lichen planus. A preliminary study. Medicina Oral, Patologia Oral y Cirugia Bucal, 21(3), e328--e334.

Garcia-Pola, M.-J., & Huerta, G. (2000). Ansiety as an etiologic factor in oral lichen planus. Medicina Oral : Organo Oficial de La Sociedad Espanola de Medicina Oral y de La Academia Iberoamericana de Patologia y Medicina Bucal, 5(1), 7–13.

Ge, Y., Xu, Y., Sun, W., Man, Z., Zhu, L., Xia, X., Zhao, L., Zhao, Y., & Wang, X. (2012). The molecular mechanisms of the effect of Dexamethasone and Cyclosporin A on TLR4 /NF-kappaB signaling pathway activation in oral lichen planus. Gene, 508(2), 157–164.

Ghabanchi, J., Fattahi, M. J., Mardani, M., Tadbir, A. A., Paydar, A. A. (2009). Polymorphism of tumor protein p53 codon 72 showed no association with oral lichen planus in Shiraz, Iran. The Journal of Craniofacial Surgery, 20(6), 2168–2170.

Ghalayani, P., Jahanshahi, G., & Saberi, Z. (2012). Degranulated mast cells and TNF-alpha in oral lichen planus and oral lichenoid reactions diseases. Advanced Biomedical Research, 1, 52.

Ghalayani, P., Razavi, S. M., & Gholami, D. (2009). Comparative study of number and distribution of IgG cells in oral lichen planus and oral lichenoid lesions. Dental Research Journal, 6(1), 1–5.

Ghaleyani, P., Sardari, F., & Akbari, M. (2012). Salivary IgA and IgG in oral lichen planus and oral lichenoid reactions diseases. Advanced Biomedical Research, 1, 73.

Ghallab, N. A., El-Wakeel, N., & Shaker, O. G. (2010). Levels of salivary IFN-gamma, TNF-alfa, and TNF receptor-2 as prognostic markers in (erosive) oral lichen planus. Mediators of Inflammation, 2010, 847632.

Ghallab, N. A., Kasem, R. F., El-Ghani, S. F. A., Shaker, O. G. (2017). Gene expression of miRNA-138 and cyclin D1 in oral lichen planus. Clinical Oral Investigations, 21(8), 2481–2491.

Ghapanchi, J., Andisheh-Tadbir, A., Torkaman, P., Malekzadeh, M., & Mardani, M. (2019) 'Evaluation of the serum levels of galectin-3 in patients with oral lichen planus disease', Oral Diseases, 25(2), pp. 466–470.

Ghapanchi, Janan, Ghaderi, H., Haghshenas, M. R., Jamshidi, S., Rezazadeh, F., Azad, A., Farzin, M., Derafshi, R., Kalantari, A. H. (2019). Observational Molecular Case-Control Study of Genetic Polymorphisms 1 in Programmed Cell Death Protein-1 in Patients with Oral Lichen Planus. Asian Pacific Journal of Cancer Prevention, 20(2), 421–424.

Ghapanchi, Jannan, Haghshenas, M. R., Ghaderi, H., Amanpour, S., Nemati, V., & Kamali, F. (2014). Ctla-4 gene polymorphism in +49 a/g position: a case control study on patients with oral lichen planus. Journal of International Oral Health, 6(5), 17–21.

Ghodratnama, F., Riggio, M. P., & Wray, D. (1997). Search for human herpesvirus 6, human cytomegalovirus and varicella zoster virus DNA in recurrent aphthous stomatitis tissue. Journal of Oral Pathology and Medicine, 26(4), 192–197.

Ghodratnama, F., Wray, D., Bagg, J. (1999). Detection of serum antibodies against cytomegalovirus, varicella zoster virus and human herpesvirus 6 in patients with recurrent aphthous stomatitis. Journal of Oral Pathology and Medicine, 28(1), 12–15.

Gholizadeh, N, Mehdipour, M., Najafi, S., Bahramian, A., Garjani, S., & Khoeini Poorfar, H. (2014). Evaluation of the serum zinc level in erosive and non-erosive oral lichen planus. Journal of Dentistry (Shiraz, Iran), 15(2), 52–56.

Gholizadeh, Narges, Khoini Poorfar, H., TaghaviZenouz, A., Vatandoost, M., & Mehdipour, M. (2015). Comparison of Serum Autoantibodies to Desmogleins I, III in Patients with Oral Lichen Planus and Healthy Controls. Iranian Journal of Pathology, 10(2), 136–140.

Giovannelli, L., Campisi, G., Colella, G., Capra, G., Di Liberto, C., Caleca, M. P., Matranga, D., D'Angelo, M., Lo Muzio, L., Ammatuna, P. (2006). Brushing of oral

mucosa for diagnosis of HPV infection in patients with potentially malignant and malignant oral lesions. Molecular Diagnosis and Therapy, 10(1), 49–55.

Girardi, C., Luz, C., Cherubini, K., de Figueiredo, M. A. Z., Nunes, M. L. T., & Salum, F. G. (2011). Salivary cortisol and dehydroepiandrosterone (DHEA) levels, psychological factors in patients with oral lichen planus. Archives of Oral Biology, 56(9), 864–868.

Girish, H. C., Murgod, S., & Savita, J. K. (2010). Epithelial dysplasia in lichenplanus. Journal of advanced oral research, 1(1), 19–26.

Girod, S. C., Krueger, G., & Pape, H. D. (1993). p53 and Ki 67 expression in preneoplastic and neoplastic lesions of the oral mucosa. International Journal of Oral and Maxillofacial Surgery, 22(5), 285–288.

Gissi, D. B., Gabusi, A., Tarsitano, A., Asioli, S., Rossi, R., Marchetti, C., Montebugnoli, L., Foschini, M. P., & Morandi, L. (2020). Application of a non-invasive oral brushing procedure based on bisulfite sequencing of a 13-gene panel to study high-risk OSCC patients. Cancer Biomarkers: Section A of Disease Markers, 28(4),

Giustina, T. A., Stewart, J. C., Ellis, C. N., Regezi, J. A., Annesley, T., Woo, T. Y., Voorhees, J. J. (1986). Topical application of isotretinoin gel improves oral lichen planus. A double-blind study. Archives of Dermatology, 122(5), 534–536.

Goel, S., Khurana, N., Marwah, A., & Gupta, S. (2015). Expression of cdk4 and p16 in Oral Lichen Planus. Journal of Oral & Maxillofacial Research, 6(2), e4–e4.

Goel, S., Marwah, A., Kaushik, S., Garg, V. K., & Gupta, S. (2015). Role of serum interleukin-6 in deciding therapy for multidrug resistant oral lichen planus. Journal of Clinical and Experimental Dentistry, 7(4), e477--82.

Gonzaga, A., Lopes, M., Squarize, C. H., Castilho, R. M., de Medeiros, A., Rocha, K., & da Silveira, É. (2020). Expression profile of DNA repair proteins and histone H3 lys-9 acetylation in cutaneous and oral lichen planus. Archives of Oral Biology, 119, 104880.

Gonzalez-Moles, M A, Bravo, M., Gonzalez-Ruiz, L., Ramos, P., Gil-Montoya, J. A. (2018). Outcomes of oral lichen planus and oral lichenoid lesions treated with topical corticosteroid. Oral Diseases, 24(4), 573–579.

Gonzalez-Moles, M A, Gil-Montoya, J. A., Ruiz-Avila, I., Bravo, M. (2017). Is oral cancer incidence among patients with oral lichen planus/oral lichenoid lesions underestimated? Journal of Oral Pathology and Medicine, 46(2), 148–153.

Gonzalez-Moles, M A, Gonzalez-Moles, S., Ruiz-Avila, I., Esteban, F., Galindo-Moreno, P., Rodriguez-Archilla, A. (1996). Epithelial response to the immunitary aggression in oral lichen planus. Acta Stomatologica Belgica, 93(3), 119–123.

Gonzalez-Moles, M A, Rodriguez-Archilla, A., Ruiz Avila, I., Esteban, F., Gonzalez-Moles, S. (1998). Presence of HPV 16 sequences in oral lichen planus lesions. Bulletin Du Groupement International Pour La Recherche Scientifique En Stomatologie & Odontologie, 40(2), 92–97.

Gonzalez-Moles, M A, Scully, C. (2010). HPA-suppressive effects of aqueous clobetasol propionate in the treatment of patients with oral lichen planus. Journal of the European Academy of Dermatology and Venereology, 24(9), 1055–1059.

González-Moles, M. A., Bascones-Ilundain, C., Gil Montoya, J. A., Ruiz-Avila, I., Delgado-Rodríguez, M., Bascones-Martínez, A. (2006). Cell cycle regulating mechanisms in oral lichen planus: Molecular bases in epithelium predisposed to malignant transformation. Archives of Oral Biology, 51(12), 1093–1103.

Gonzalez-Moles, M. Á., Ruiz-Avila, I., Rodriguez-Archilla, A., Morales-Garcia, P., Mesa-Aguado, F., Bascones-Martinez, A., & Bravo, M. (2003). Treatment of severe erosive gingival lesions by topical application of clobetasol propionate in custom trays. Oral Surgery, Oral Medicine, Oral Pathology, Oral Radiology, and Endodontics, 95(6), 688–692.

Gonzalez-Moles, M.Á. , Ruiz-Avila, I., Muwaquet-Rodriguez, S., Fernandez-Martinez, J., Bravo, I., & Esteban-Ortega, F. (1997). HLA-DR and intercelular adhesion molecula-1 (ICAM-1) expression in oral lichen planus. Medicina Oral: Órgano Oficial de La Sociedad Espanola de Medicina Oral y de La Academia Iberoamericana de Patologia y Medicina Bucal, 2(1), 14–20.

Gorouhi, F., Solhpour, A., Beitollahi, J. M., Afshar, S., Davari, P., Hashemi, P., Nassiri Kashani, M., & Firooz, A. (2007). Randomized trial of pimecrolimus cream versus triamcinolone acetonide paste in the treatment of oral lichen planus. Journal of the American Academy of Dermatology, 57(5), 806–813.

Gorsky, M., Epstein, J. B., Hasson-Kanfi, H., & Kaufman, E. (2004). Smoking habits among patients diagnosed with oral lichen planus. Tobacco Induced Diseases, 2(2), 103–108.

Gorugantula, L. M., Rees, T., Plemons, J., Chen, H.-S., Cheng, Y.-S. (2012). Salivary basic fibroblast growth factor in patients with oral squamous cell carcinoma or oral lichen planus. Oral Surgery, Oral Medicine, Oral Pathology and Oral Radiology, 114(2), 215–222.

Griffin, C. J. (1980). The fine structure of epithelial cells in normal and pathological buccal mucosa. IV. Interactions between epithelial cells, lymphocytes, and macrophages. Australian Dental Journal, 25(5), 284–294.

Griffith, M., Kaufman, H. S., Silverman, S. (1974). Studies on oral lichen planus. I. Serum immunoglobulins and complement. Journal of dental research, 53(3), 623–626.

Gu, G. M., Martin, M. D., Darveau, R. P., Truelove, E., Epstein, J. (2004). Oral and serum IL-6 levels in oral lichen planus patients. Oral Surgery, Oral Medicine, Oral Pathology, Oral Radiology, and Endodontics, 98(6), 673–678.

Guan, G., Mei, L., Polonowita, A., Hussaini, H., Seo, B., & Rich, A. M. (2020). Malignant transformation in oral lichen planus and lichenoid lesions: a 14-year longitudinal retrospective cohort study of 829 patients in New Zealand. Oral Surgery, Oral Medicine, Oral Pathology and Oral Radiology, 130(4), 411–418.

Guan, Z. J., Liu, Z. J., Tian, C. Z., Sun, Q., Zhu, X. H., & Yan, Y. X. (1996). [Detection of T cell subtypes in the epithelium of oral lichen planus lesions]. Shanghai Kou Qiang Yi Xue = Shanghai Journal of Stomatology, 5(3), 174–175.

Gueiros, L. A., Arao, T., Souza, T., Vieira, C. L., Gomez, R. S., Almeida, O. P., Lodi, G., Leao, J. C. (2018). IL17A polymorphism and elevated IL17A serum levels are associated with oral lichen planus. Oral Diseases, 24(3), 377–383.

Guido, A., Maglione, M., Crispo, A., Perri, F., Villano, S., Pavone, E., Aversa, C., Longo, F., Feroce, F., Botti, G., Ionna, F. (2019). Oral lichen planus and other confounding factors in narrow band imaging (NBI) during routine inspection of oral cavity for early detection of oral squamous cell carcinoma: a retrospective pilot study. BMC oral health, 19(1), 70.

Guido, L., Massimo, V., Elettra, C., Glauco, C., Maria, P., Lorenzini, G., Viviano, M., Chisci, E., Chisci, G., & Picciotti, M. (2013). A comparative immunohistochemical and immunophenotypical study on lymphocytes expression in patients affected by oral lichen planus. Journal of oral pathology & medicine, 42(8), 642–647.

Gunatheesan, S., Tam, M. M., Tate, B., Tversky, J., Nixon, R. (2012). Retrospective study of oral lichen planus and allergy to spearmint oil. Australasian Journal of Dermatology, 53(3), 224–228.

Guo, Y. S., Li, S., Lyu, M. Y., Yang, D., & Hua, H. (2017). [Analysis of type C behavior in patients with oral lichen planus]. Beijing Da Xue Xue Bao. Yi Xue Ban = Journal of Peking University. Health Sciences, 49(1), 120–124.

Gupta, P.C. et al. (1995) ‘Effect of cessation of tobacco use on the incidence of oral mucosal lesions in a 10-yr follow-up study of 12,212 users.’, Oral diseases, 1(1), pp. 54–58.

Hakkinen L., Kainulainen T., Salo T., Grenman R. and Larjava H. (1999), "Expression of integrin alpha9 subunit and tenascin in oral leukoplakia, lichen planus, and squamous cell carcinoma.", Oral diseases. Vol. 5(3), pp. 210-217.

Halonen, P. et al. (2020) ‘Incidence of Lichen Planus and Subsequent Mortality in Finnish Women.’, Acta dermato-venereologica, 100(17), p. adv00303.

Hämäläinen L., Soini Y., Pasonen-Seppänen S., Siponen M., Hämäläinen L., Soini Y., Pasonen-Seppänen S., Siponen M. (2019), "Alterations in the expression of EMT-related proteins claudin-1, claudin-4 and claudin-7, E-cadherin, TWIST1 and ZEB1 in oral lichen planus", Journal of Oral Pathology and Medicine. Vol. 48(8), pp. 735-744.

Hambly J. L., Haywood A., Hattingh L., Nair R. G. (2017), "Comparison between self-formulation and compounded-formulation dexamethasone mouth rinse for oral lichen planus: a pilot, randomized, cross-over trial", Journal of investigative and clinical dentistry. Vol. 8(3)

Hashem, A. S., Issrani, R., Elsayed, T. E. E., & Prabhu, N. (2019). Topical hyaluronic acid in the management of oral lichen planus: A comparative study. Journal of Investigative and Clinical Dentistry, 10(2), e12385–e12385.

Hashemy S. I., Gharaei S., Vasigh S., Kargozar S., Alirezaei B., Keyhani F. J., Amirchaghmaghi M. (2016), "Oxidative stress factors and C-reactive protein in patients with oral lichen planus before and 2 weeks after treatment", Journal of Oral Pathology and Medicine. Vol. 45(1), pp. 35-40.

Hashimoto T., Fukuda A., Himejima A., Morita S., Tsuruta D., Koga H., Krol R. P. and Ishii N. (2015), "Ten cases of severe oral lichen planus showing granular C3 deposition in oral mucosal basement membrane zone.", European journal of dermatology. Vol. 25(6), pp. 539-547.

Hassona Y., Scully C., Almangush A., Baqain Z. and Sawair F. (2014), "Oral potentially malignant disorders among dental patients: a pilot study in Jordan.", Asian Pacific journal of cancer prevention. Vol. 15(23), pp. 10427-10431.

Hatchuel D. A., Peters E., Lemmer J., Hille J. J., McGaw W. T. (1990), "Candidal infection in oral lichen planus", Oral Surgery Oral Medicine and Oral Pathology. Vol. 70(2), pp. 172-175.

Hazzaa H. H. A., El-Wakeel N. M., Attia E. A. S., Abo Hager E. A. (2016), "ALK1 expression in oral lichen planus: A possible relation to microvessel density", Journal of Oral Pathology and Medicine. Vol. 45(5), pp. 373-380.

He H., Xia X., Yang H., Peng Q. and Zheng J. (2020), "A pilot study: a possible implication of Candida as an etiologically endogenous pathogen for oral lichen planus.", BMC oral health. Vol. 20(1), pp. 72.

Hebbar P. B., Pai A. and Sujatha D. (2013), "Mycological and histological associations of Candida in oral mucosal lesions.", Journal of oral science. Vol. 55(2), pp. 157-160.

Hegarty A. M., Hodgson T. A., Lewsey J. D. and Porter S. R. (2002), "Fluticasone propionate spray and betamethasone sodium phosphate mouthrinse: A randomized crossover study for the treatment of symptomatic oral lichen planus", Journal of the American Academy of Dermatology. Vol. 47(2), pp. 271-279.

Hegarty, A. M., McGrath, C., Hodgson, T. A., & Porter, S. R. (2002). Patient-centred outcome measures in oral medicine: Are they valid and reliable? International Journal of Oral and Maxillofacial Surgery, 31(6), 670–674.

Helenius-Hietala, J., Ruokonen, H., Grönroos, L., Rissanen, H., Vehkalahti, M. M., Suominen, L., Isoniemi, H., & Meurman, J. H. (2014) ‘Oral mucosal health in liver transplant recipients and controls’, Liver Transplantation, 20(1), pp. 72–80.

Heo J-Y, Ok S-M, Ahn Y-W, Ko M-Y and Jeong S-H (2015), "The application of neuropathic pain questionnaires in burning mouth syndrome patients.", Journal of oral & facial pain and headache. Vol. 29(2), pp. 177-182.

Hernández, G., Lopez-Pintor, R. M., Arriba, L., Torres, J., & de Vicente, J. C. (2012). Implant treatment in patients with oral lichen planus: A prospective-controlled study. Clinical Oral Implants Research, 23(6), 726–732.

Hettiarachchi PVKS, Hettiarachchi RM, Jayasinghe RD and Sitheeque M (2017), "Comparison of topical tacrolimus and clobetasol in the management of symptomatic oral

lichen planus: A double-blinded, randomized clinical trial in Sri Lanka.", Journal of investigative and clinical dentistry. Vol. 8(4)

Hirota J., Osaki T. (1992), "Electron microscopic study on cell-to-cell interactions in oral lichen planus", Pathology Research and Practice. Vol. 188(8), pp. 1033-1041.

Hirota M, Ito T, Okudela K, Kawabe R, Yazawa T, Hayashi H, Nakatani Y, Fujita K and Kitamura H (2002), "Cell proliferation activity and the expression of cell cycle regulatory proteins in oral lichen planes", Journal of oral pathology & medicine: official publication of the International Association of Oral Pathologists and the American Academy of Oral Pathology. Vol. 31(4), pp. 204-212. Wiley.

Hirota, J., Osaki, T., & Tatemoto, Y. (1990) 'Immunohistochemical staining of infiltrates in oral lichen planus', Pathology Research and Practice, 186(5), pp. 625–632.

Hirota, J., Yoneda, K., & Osaki, T. (1989) 'Destruction of basement membrane and cell infiltrates in oral lichen planus', Pathology Research and Practice, 185(2), pp. 218–224.

Hodak E, Yosipovitch G, David M, Ingber A, Chorev L, Lider O, Cahalon L and Cohen IR (1998), "Low-dose low-molecular-weight heparin (enoxaparin) is beneficial in lichen planus: a preliminary report.", Journal of the American Academy of Dermatology. Vol. 38(4), pp. 564-568.

Holbrook W. P., Kristmundsdottir T. and Loftsson T. (1998), "Aqueous hydrocortisone mouthwash solution: clinical evaluation.", Acta odontologica Scandinavica. Vol. 56(3), pp. 157-160.

Holmstrup P. and Pindborg J. J. (1979), "Erythroplakic lesions in relation to oral lichen planus", Acta Dermato-Venereologica. Vol. 59, pp. 77-84.

Holmstrup P., Dabelsteen E. (1974), "The frequency of Candida in oral lichen planus", Scandinavian Journal of Dental Research. Vol. 82(8), pp. 584-587.

Holmstrup P., Thorn J. J., Rindum J., Pindborg J. J. (1988), "Malignant development of lichen planus-affected oral mucosa.", Journal of oral pathology. Vol. 17(5), pp. 219-225.

Hosni ES, Yurgel L. S. & da Silva V. D. (2010), "DNA ploidy in oral lichen planus, determined by image cytometry", Journal of Oral Pathology and Medicine. Vol. 39(3), pp. 206-211.

Hsieh P-C, Chen Y-K, Tsai K-B, Shieh T-Y, Chang Y-Y, Chang J-G, Wu H-L and Lin S-F (2010), "Expression of BUBR1 in human oral potentially malignant disorders and squamous cell carcinoma.", Oral surgery, oral medicine, oral pathology, oral radiology, and endodontics. Vol. 109(2), pp. 257-267.

Hsu H-J, Yang Y-H, Shieh T-Y, Chen C-H, Kao Y-H, Yang C-F and Ko EC-C (2014), "Role of cytokine gene (interferon-gamma, transforming growth factor-beta 1, tumor necrosis factor-alpha, interleukin-6, and interleukin-10) polymorphisms in the risk of oral precancerous lesions in Taiwanese", Kaohsiung journal of medical sciences. Vol. 30(11), pp. 551-558. Elsevier taiwan.

Hsue S-S, Wang W-C, Chen C-H, Lin C-C, Chen Y-K and Lin L-M (2007), "Malignant transformation in 1458 patients with potentially malignant oral mucosal disorders: a follow-up study based in a Taiwanese hospital.", Journal of oral pathology & medicine. Vol. 36(1), pp. 25-29.

Hu J-Y, Zhang J, Cui J-L, Liang X-Y, Lu R, Du G-F, Xu X-Y, Zhou G. (2013), "Increasing CCL5/CCR5 on CD4+ T cells in peripheral blood of oral lichen planus", Cytokine. G. Zhou, Department of Oral Medicine, School and Hospital of Stomatology, Wuhan University, Luoyu Road 237, Wuhan 430079, China Vol. 62(1), pp. 141-145.

Hu, A. P. & Liu, Z. X. (2016). Clinical effect of Nd:YAG laser combined with total glucosides of paeony for the treatment of erosive oral lichen planus. Shanghai Kou Qiang Yi Xue = Shanghai Journal of Stomatology, 25(4), 481–483.

Huang Y, Zhou S, Cai Y (2016), "Expression of interleukin-12 and interleukin-27 proteins and immune status in serum of patients with oral lichen planus", Hua xi kou qiang yi xue za zhi = Huaxi kouqiang yixue zazhi = West China journal of stomatology. Vol. 34(2), pp. 140-144.

Huang, M. K. (1980) ‘Therapeutic effects of “shung dong” and “snow flakes” in 56 cases of oral lichen planus (author’s transl)’, Zhonghua kou qiang ke za zhi [Chinese journal of stomatology], 15(3), pp. 166–168.

Hulimavu SR, Mohanty L, Tondikulam NV, Shenoy S, Jamadar S, Bhadranna A. (2014), "No evidence for Helicobacter pylori in oral lichen planus", Journal of Oral Pathology and Medicine. Vol. 43(8), pp. 576-578.

Hüpsch-Marzec H. and Wiench R. (2016), "Results of 0.2% hyaluronan therapy in patients suffering from oral atrophy: A subjective evaluation of patients", Dental and Medical Problems. Vol. 53(1), pp. 153-154.

Hussein A. A. (2019), "Evaluation of syndecan-1 and p53 protein in patients with oral lichen panus", Research Journal of Biotechnology. Vol. 14, pp. 140-144. World Research Association.

Hyman, G. A., Fingerhut, B., Zegarelli, E. V, Zegarelli, D. J., & Zegarelli-Schmidt, E. C. (1982). Autoradiographic studies of oral lichen planus. Oral Surgery Oral Medicine and Oral Pathology, 54(2), 172–179.

Ichimura, M., Hiratsuka, K., Ogura, N., Utsunomiya, T., Sakamaki, H., Kondoh, T., Abiko, Y., Otake, S., Yamamoto, M. (2006). Expression profile of chemokines and chemokine receptors in epithelial cell layers of oral lichen planus. Journal of Oral Pathology and Medicine, 35(3), 167–174.

Iijima, W., Ohtani, H., Nakayama, T., Sugawara, Y., Sato, E., Nagura, H., Yoshie, O., Sasano, T. (2003). Infiltrating CD8+ T cells in oral lichen planus predominantly express CCR5 and CXCR3 and carry respective chemokine ligands RANTES/CCL5 and IP-10/CXCL10 in their cytolytic granules: A potential self-recruiting mechanism. American Journal of Pathology, 163(1), 261–268.

Ikeda, N., Downer, M. C., Ishii, T., Fukano, H., Nagao, T., & Inoue, K. (1995). Annual screening for oral cancer and precancer by invitation to 60-year-old residents of a city in Japan. Community Dental Health, 12(3), 133–137.

Ikeda, N., Handa, Y., Khim, S. P., Durward, C., Axell, T., Mizuno, T., Fukano, H., & Kawai, T. (1995). Prevalence study of oral mucosal lesions in a selected Cambodian population. Community Dentistry and Oral Epidemiology, 23(1), 49–54.

Irani, S., Esfahani, A. M., & Ghorbani, A. (2016). Dysplastic change rate in cases of oral lichen planus: A retrospective study of 112 cases in an Iranian population. Journal of Oral and Maxillofacial Pathology. 20(3), 395–399.

Ito, D., Sugawara, Y., Jinbu, Y., Nakamura, S., Fujibayashi, T., Maeda, H., Hasegawa, H., Saku, T., Tanaka, A., & Komiyama, K. (2017). A retrospective multi-institutional study on the clinical categorization and diagnosis of oral lichen planus. Journal of oral and maxillofacial surgery medicine and pathology, 29(5), 452–457.

Ivanyi, L. (1990). Elevated antibody levels to mycobacterial 65-kDa stress protein in patients with superficial candidiasis. Journal of Infectious Diseases, 162(2), 519–522.

J., M. (1976) ‘Comparative immunofluorescent investigations in oral lichen planus and leucoplakia (Polish)’, Czasopismo Stomatologisczne, 29(9), pp. 735–743.

J., P. et al. (1998) ‘Immunoregulation effect of traditional Chinese medicine treatment on patients with oral lichen planus’, Zhonghua kou qiang yi xue za zhi = Zhonghua kouqiang yixue zazhi = Chinese journal of stomatology, 33(1), pp. 48–49.

J., S. et al. (2014) ‘Immunohistochemical expression of p16 protein in oral squamous cell carcinoma and lichen planus’, Annals of Diagnostic Pathology, 18(4), pp. 210–213.

J., S., J.R., R., et al. (2004) ‘Expression of heat shock protein (HSP70) in oral lichen planus and non-dysplastic oral leucoplakia’, Clinical Otolaryngology and Allied Sciences, 29(2), pp. 191–196.

J., S., M.A., R., et al. (2004) ‘Oral lichen planus: A clinical and morphometric study of oral lesions in relation to clinical presentation’, Brazilian Dental Journal, 15(1), pp. 9–12.

J., Shan, C., S., et al. (2019) ‘Potential roles of the CCL17-CCR4 axis in immunopathogenesis of oral lichen planus’, Journal of Oral Pathology and Medicine.

J., Shan, S., L., et al. (2019) ‘Expression and biological functions of the CCL5-CCR5 axis in oral lichen planus’, Experimental Dermatology, 28(7), pp. 816–821.

J., Shen et al. (2019) ‘Aberrant histone modification and inflammatory cytokine production of peripheral CD4+ T cells in patients with oral lichen planus’, Journal of Oral Pathology and Medicine, 48(2), pp. 136–142.

J., T. et al. (2018) ‘Impact of oral potentially malignant disorders on quality of life’, Journal of Oral Pathology and Medicine, 47(1), pp. 60–65.

J., W. et al. (2016) ‘MiR-125b inhibits keratinocyte proliferation and promotes keratinocyte apoptosis in oral lichen planus by targeting MMP-2 expression through PI3 K/Akt/mTOR pathway’, Biomedicine and Pharmacotherapy, 80, pp. 373–380.

J., X. et al. (2006) ‘Short-term clinical evaluation of intralesional triamcinolone acetonide injection for ulcerative oral lichen planus’, Journal of Oral Pathology and Medicine, 35(6), pp. 327–331.

J., Y. et al. (2016) ‘The positive correlation of the CCL2-CCR2 axis with the disease activity may indicate the fundamental role in the pathogenesis of oral lichen planus’, Journal of Oral Pathology and Medicine, 45(1), pp. 41–47.

J., Z. et al. (2011) ‘The progress in expression of genes of the oligodendrocyte lineage-myelin basic protein in oral lichen planus’, Hua xi kou qiang yi xue za zhi = Huaxi kouqiang yixue zazhi = West China journal of stomatology, 29(6), pp. 576–579.

J.A., R. et al. (1986) ‘Histologic changes associated with topical use of isotretinoin on oral lichen planus’, Oral Surgery Oral Medicine and Oral Pathology, 61(5), pp. 479–484.

J.A., R. et al. (1994) ‘Increased submucosal factor XIIIa-positive dendrocytes in oral lichen planus.’, Journal of oral pathology & medicine, 23(3), pp. 114–118.

J.B., T. et al. (2016) ‘Distribution of ABO blood groups and Rh type in patients with oral lichen planus’, Journal of Mazandaran University of Medical Sciences, 26(141), pp. 155–159.

J.J., J. and G., S. (1976) ‘Lichen planus of the gingiva’, Journal of Periodontology, 47(12), pp. 724–733.

J.J., P. et al. (1972) ‘Prevalence of oral lichen planus among 7639 Indian villagers in Kerala, South India.’, Acta dermato-venereologica, 52(3), pp. 216–220.

J.J., T. et al. (1988) ‘Course of various clinical forms of oral lichen planus. A prospective follow-up study of 611 patients.’, Journal of oral pathology, 17(5), pp. 213–218.

J.L., R.-M. et al. (1998) ‘Psychologic factors and oral lichen planus. A psychometric evaluation of 100 cases.’, Oral surgery, oral medicine, oral pathology, oral radiology, and endodontics, 86(6), pp. 687–691.

J.M., B. et al. (2008) ‘Role of apoptosis in erosive and reticular oral lichen planus exhibiting variable epithelial thickness.’, Brazilian dental journal, 19(3), pp. 179–185.

J.M., M. et al. (2015) ‘Galectin-9 as an important marker in the differential diagnosis between oral squamous cell carcinoma, oral leukoplakia and oral lichen planus’, Immunobiology, 220(8), pp. 1006–1011.

J.M., S.-B. et al. (1994) ‘Oral lichen planus. An evolutive clinical and histological study of 45 patients followed up on for five years.’, Bulletin du Groupement international pour la recherche scientifique en stomatologie & odontologie, 37(1), pp. 45–49.

J.M.C., B. et al. (2012) ‘A comparative study of apoptosis in reticular and erosive oral lichen planus’, Brazilian Dental Journal, 23(5), pp. 564–569.

J.O., A. and Andreasen, J. O. (1968) ‘Oral lichen planus. 1. A clinical evaluation of 115 cases.’, Oral surgery, oral medicine, and oral pathology, 25(1), pp. 31–42.

J.S., L. et al. (2009) ‘Neoplasia/dysplasia surveillance of oral lichen planus in Malaysia: a preliminary study using topography maps.’, Asian Pacific journal of cancer prevention, 10(6), pp. 1071–1074.

J.S., P. et al. (2012) ‘FoxP3+ T regulatory cells in oral lichen planus and its correlation with the distinct clinical appearance of the lesions’, International Journal of Experimental Pathology, 93(4), pp. 287–294.

J.T., W. et al. (2015) ‘Oral lichen planus: Study of 21 cases’, Anais Brasileiros de Dermatologia, 90(3), pp. 321–326.

J.T., W. et al. (2016) ‘CLA and CD62E expression in oral lichen planus lesions’, Journal of Oral Pathology and Medicine, 45(3), pp. 218–223.

J.V., B., A., P. and F.J., V. S. (1988) ‘Ultrastructural study of oral lichen planus. Analysis of 10 cases’, Archivos de odonto estomatologia, 4(1), pp. 9–14.

J.Y.-F., C. et al. (2017) ‘Antigastric parietal cell and antithyroid autoantibodies in patients with desquamative gingivitis’, Journal of Oral Pathology and Medicine, 46(4), pp. 307–312.

Jaafari-Ashkavandi, Z. et al. (2011) ‘Oral mucocutaneous diseases: Clinicopathologic analysis and malignant transformation’, Journal of Craniofacial Surgery, 22(3), pp. 949–951.

Jaafari-Ashkavandi, Z. et al. (2013) ‘Evaluation of proliferation activity in dysplastic and nondysplastic oral lichen planus through the analysis of argyrophilic nucleolar organizer regions’, The Journal of craniofacial surgery, 24(3), pp. 788–791.

Jaafari-Ashkavandi, Z. et al. (2017) ‘Caveolin-1 expression in oral lichen planus, dysplastic lesions and squamous cell carcinoma’, Pathology Research and Practice, 213(7), pp. 809–814.

Jablonska, E., Garley, M., Surazynski, A., Grubczak, K., Iwaniuk, A., Borys, J., Moniuszko, M., & Ratajczak-Wrona, W. (2020). Neutrophil extracellular traps (NETs) formation induced by TGF-β in oral lichen planus - Possible implications for the development of oral cancer. Immunobiology, 225(2), 151901.

Jacques, C. M. C., Pereira, A. L. C., Maia, V., Cuzzi, T., & Ramos-e-Silva, M. (2009). Expression of cytokeratins 10, 13, 14 and 19 in oral lichen planus. Journal of Oral Science, 51(3), 355–365.

Jagtap, K. et al. (2012) ‘Estimation of salivary nitric oxide in recurrent aphthous ulcer and oral lichen planus patients with its clinical significance’, Journal of Contemporary Dental Practice, 13(5), pp. 623–626.

Jahanbani, J. (2003) ‘Prevalence of oral leukoplakia and lichen planus in 1167 Iranian textile workers.’, Oral diseases, 9(6), pp. 302–304.

Jahanshahi, G., Ghalayani, P. and Maleki, L. (2012) 'Mast cells distribution and variations in epithelium thickness and basement membrane in oral lichen planus lesion and oral lichenoid reaction.', Dental research journal, 9(2), pp. 180–184.

Jajarm H. H., Falaki F., Mahdavi O. (2011), "A comparative pilot study of low intensity laser versus topical corticosteroids in the treatment of erosive-atrophic oral lichen planus.", Photomedicine and laser surgery. Vol. 29(6), pp. 421-425.

Jajarm, H. H. et al. (2015) 'A comparative study of toluidine blue-mediated photodynamic therapy versus topical corticosteroids in the treatment of erosive-atrophic oral lichen planus: a randomized clinical controlled trial', Lasers in Medical Science, 30(5), pp. 1475–1480.

Jana, A. et al. (2017) 'P-glycoprotein expression in oral lichen planus', Brazilian oral research, 31, pp. e95–e95.

Jana, A., & Ghosh, P. (2014). Altered hematological profile of oral lichen planus patients. Research Journal of Pharmaceutical, Biological and Chemical Sciences, 5(5), 1271–1277.

Janardhanam, S. B. et al. (2012) 'Differential expression of TLR-2 and TLR-4 in the epithelial cells in oral lichen planus', Archives of Oral Biology, 57(5), pp. 495–502.

Janardhanan, M. and Ramesh, V. (2010) 'Mast cells in oral lichen planus', oral & maxillofacial pathology journal, 1(2).

Javadzadeh, A. et al. (2008) 'Efficacy of Clobetasol, Ketoconazole and Amitryptiline Mouthwash on Oral Lichen Planus', Iranian journal of pharmaceutical research, 7(3), pp. 171–178.

Jeon, I. K., On, H. R. and Kim, S.-C. (2016) 'Quality of Life and Economic Burden in Recessive Dystrophic Epidermolysis Bullosa.', Annals of dermatology, 28(1), pp. 6–14.

Jiang, C., Yao, H., Cui, B., Zhou, Y., Wang, Y., & Tang, G. (2015). Association of interleukin 12A gene polymorphisms with oral lichen planus in Chinese population. Journal of Oral Pathology and Medicine, 44(8), 602–606.

Jiménez, M. E. F. et al. (2009) 'Epithelial dysplasia as histopathologic characteristic of oral lichen planus [Displasia epitelial como caracteristica histopatologica del liquen plano bucal]', Revista Habanera de Ciencias Medicas, 8(4).

Jin, Z. et al. (2001) 'A study of millimeter wave's clinical and immunological effects on oral lichen planus patients', Hua xi kou qiang yi xue za zhi = Huaxi kouqiang yixue zazhi = West China journal of stomatology, 19(6), pp. 366–368.

Jolly, M. and Nobile, S. (1977) 'Vitamin status of patients with oral lichen planus.', Australian dental journal, 22(6), pp. 446–450.

Jose, S. et al. (2019) 'Estimation of serum cortisol levels in oral lichen planus patients with electrochemiluminescence', Journal of Pharmacy and Bioallied Sciences, 11(6), pp. S265--S268.

Joshy, A. et al. (2018) ‘To Evaluate the Efficacy of Topical Propolis in the Management of Symptomatic Oral Lichen Planus: A Randomized Controlled Trial.’, Contemporary clinical dentistry, 9(1), pp. 65–71.

Ju, H. M., Ahn, Y. W., Jeong, S. H., Jeon, H. M., Kim, K. H., Song, B. S., & Ok, S. M. (2019) ‘Characteristics of the patients who perceived dental treatment as a cause of oral mucosal lesions’, Journal of Oral Pathology and Medicine, 48, p. 12.

Juneja, M. et al. (2006) ‘Histochemical analysis of pathological alterations in oral lichen planus and oral lichenoid lesions.’, Journal of oral science, 48(4), pp. 185–193.

Jungell, P. (1990) ‘Immunoelectron microscopic study of the basement membrane in oral lichen planus’, Journal of Cutaneous Pathology, 17(2), pp. 72–76.

Juretic, M. et al. (2013) ‘Salivary levels of TNF-alpha and IL-6 in patients with oral premalignant and malignant lesions.’, Folia biologica, 59(2), pp. 99–102.

K, B. et al. (2020) ‘Characterization of intratissue bacterial communities and isolation of Escherichia coli from oral lichen planus lesions.’, Scientific reports, 10(1), p. 3495.

K, H. et al. (2020) ‘Oral bacterial diversity is inversely correlated with mucosal inflammation.’, Oral diseases, 26(7), pp. 1566–1575.

K, L. et al. (2020) ‘Increased regulatory T cells and eosinophils characterize atopic dermatitis-like graft-versus-host disease compared with lichen planus-like graft-versus-host disease.’, Journal of the American Academy of Dermatology, 83(3), pp. 824–831.

K, W. et al. (2020) ‘Evaluation of synuclein-γ levels by novel monoclonal antibody in saliva and cancer tissues from oral squamous cell carcinoma patients.’, Neoplasma, 67(3), pp. 707–713.

K.-M., W. et al. (2013) ‘Modulation of serum smooth muscle antibody levels by levamisole treatment in patients with oral lichen planus’, Journal of the Formosan Medical Association, 112(6), pp. 352–357.

K., C.-C. et al. (2008) ‘Assessment of the peripheral immunocompetent cells in patients with reticular and atrophic-erosive lichen planus.’, Oral surgery, oral medicine, oral pathology, oral radiology, and endodontics, 105(2), pp. 202–205.

K., D. et al. (2014) ‘Genes involved in epithelial differentiation and development are differentially expressed in oral and genital lichen planus epithelium compared to normal epithelium’, Acta Dermato-Venereologica, 94(5), pp. 526–530.

K., D. et al. (2018) ‘Epstein-barr virus is not detected in mucosal lichen planus’, Medicina Oral Patologia Oral y Cirugia Bucal, 23(5), pp. e560--e563.

K., E.-S., A., Y. and A., K. (2013) ‘A self-controlled single blinded clinical trial to evaluate oral Lichen planus after topical treatment with Aloe vera’, Journal of Gastroenterology and Hepatology Research, 2(4), pp. 503–507.

K., H. et al. (1982) ‘Severe oral lichen planus: Treatment with an aromatic retinoid (etretinate)’, British Journal of Dermatology, 106(1), pp. 77–80.

K., I. et al. (2005) ‘Psychological profile in oral lichen planus’, Journal of Clinical Periodontology, 32(10), pp. 1034–1040.

K., L. et al. (2019) ‘Multiple superficial mucoceles concomitant with oral lichen planus: a case series’, Oral surgery, oral medicine, oral pathology and oral radiology, 127(4), pp. e95--e101.

K., M. H. et al. (2013) ‘Relationships of Personality Factors to Perceived Stress, Depression, and Oral Lichen Planus Severity’, International journal of behavioral medicine, 20(2), pp. 286–292.

K., P. et al. (2013) ‘Phospholipase, esterase and hemolytic activities of Candida spp. isolated from onychomycosis and oral lichen planus lesions’, Journal de Mycologie Medicale, 23(2), pp. 113–118.

K., S. et al. (2018) ‘Ectopic transglutaminase 1 and 3 expression accelerating keratinization in oral lichen planus’, Journal of International Medical Research, 46(11), pp. 4722–4730.

K., V. et al. (2013) ‘The influence of psychological state on oral lichen planus’, Acta Clinica Croatica, 52(2), pp. 145–150.

K.D., R. et al. (2018) ‘A study of prevalence of autoantibodies in patients with lichen planus from Mumbai, India’, Indian Journal of Dermatology, Venereology and Leprology, 84(6), pp. 667–671.

K.P., S. et al. (2017) ‘Role of cathepsin B as a marker of malignant transformation in oral lichen planus: An immunohistochemical study’, Journal of Clinical and Diagnostic Research, 11(7), pp. ZC81--ZC84.

K.R., L., J.D., J., J., R., C., Z., A.M.L., P., et al. (2017) ‘Symptomatic oral lesions may be associated with contact allergy to substances in oral hygiene products’, Clinical oral investigations, 21(8), pp. 2543–2551.

K.R., L., J.D., J., J., R., C., Z., K., R., et al. (2017) ‘Filaggrin gene mutations and the distribution of filaggrin in oral mucosa of patients with oral lichen planus and healthy controls’, Journal of the European Academy of Dermatology and Venereology, 31(5), pp. 887–893.

K.S., A. et al. (2018) ‘Prevention of Malignant Transformation of Oral Leukoplakia and Oral Lichen Planus Using Laser: An Observational Study’, Asian Pacific journal of cancer prevention, 19(12), pp. 3635–3641.

Kabiraj, A. et al. (2016) ‘Screening of Oral Potentially Malignant Disorders Using Exfoliative Cytology: A Diagnostic Modality’, Journal of Cancer Epidemiology, 2016, p. 8134832.

Kaliakatsou, F., Hodgson, T. A., Lewsey, J. D., Hegarty, A. M., Murphy, A. G., & Porter, S. R. (2002). Management of recalcitrant ulcerative oral lichen planus with topical tacrolimus. Journal of the American Academy of Dermatology, 46(1), 35–41.

Kalogerakou, F. et al. (2008) ‘Detection of T cells secreting type 1 and type 2 cytokines in the peripheral blood of patients with oral lichen planus’, Hippokratia, 12(4), pp. 230–235.

Kammerer, P. W. et al. (2013) ‘Prospective, blinded comparison of cytology and DNA-image cytometry of brush biopsies for early detection of oral malignancy.’, Oral oncology, 49(5), pp. 420–426.

Kanemoto, K. et al. (1988) ‘Evaluation of Langerhans cells and T cell subsets in oral lichen planus, in comparison with oral leukoplakia.’, Shōwa Shigakkai zasshi = The Journal of Showa University Dental Society, 8(1), pp. 85–91.

Kaplan, I., Nabiochtchikov, I., Leshno, A., Moshkowitz, M., Shlomi, B., Kleinman, S., Dagan, Kraus, S. (2015). Association of CD24 and the adenomatous polyposis coli gene polymorphisms with oral lichen planus. Oral Surgery, Oral Medicine, Oral Pathology and Oral Radiology, 120(3), 378–385.

Kapoor, A. et al. (2014) ‘Evaluation of efficacy of a bioresorbable membrane in the treatment of oral lichen planus.’, Dental research journal, 11(3), pp. 386–394.

Kapoor, C. et al. (2013) ‘Triology of nitric oxide, mast cell and stress in pathogenesis of oral lichen planus.’, Journal of oral and maxillofacial pathology, 17(2), pp. 156–162.

Kar, H. K. et al. (1996) ‘Comparison of topical tretinoin and betamethasone in oral lichen planus.’, Indian journal of dermatology, venereology and leprology, 62(5), pp. 304–305.

Karagouni, E. E., Dotsika, E. N., & Sklavounou, A. (1994). Alteration in peripheral blood mononuclear cell function and serum cytokines in oral lichen planus. Journal of Oral Pathology and Medicine, 23(1), 28–35.

Karatasli, B. et al. (2018) ‘Healing of Oral Lichenoid Lesions following Replacement of Dental Amalgam Restorations with Feldspathic Ceramic Inlay-Onlay Restorations: Clinical Results of a Follow-Up Period Varied from Three Months up to Five Years.’, BioMed research international, 2018, p. 7918781.

Karatsaidis, A. et al. (2003) ‘Inhibition of the transforming growth factor-beta/Smad signaling pathway in the epithelium of oral lichen.’, The Journal of investigative dermatology, 121(6), pp. 1283–1290.

Karatsaidis, A., Hayashi, K., Schreurs, O., Helgeland, K., & Schenck, K. (2007). Survival signalling in keratinocytes of erythematous oral lichen planus. Journal of Oral Pathology and Medicine, 36(4), 215–222.

Karbach, J. et al. (2014) ‘Oral health-related quality of life of patients with oral lichen planus, oral leukoplakia, or oral squamous cell carcinoma’, Journal of Oral and Maxillofacial Surgery, 72(8), pp. 1517–1522.

Kashima H. K., Kutcher M., Kessis T., Levin L. S., de Villiers E. M. and Shah K. (1990), "Human papillomavirus in squamous cell carcinoma, leukoplakia, lichen planus, and clinically normal epithelium of the oral cavity", Annals of Otology, Rhinology and Laryngology. Vol. 99(1), pp. 55-61.

Kashyap, B. et al. (2015) ‘Evaluation of oral epithelial dysplastic features in oral lichen planus: The diagnostic difficulties’, Clinical cancer investigation journal, 4(3), pp. 327–332.

Katarkar, A. et al. (2014) ‘Comparative evaluation of genotoxicity by micronucleus assay in the buccal mucosa over comet assay in peripheral blood in oral precancer and cancer patients’, Mutagenesis, 29(5), pp. 325–334.

Katarkar, A. et al. (2015) ‘Association of oral tumor suppressor gene deleted in oral cancer-1 (DOC-1) in progression of oral precancer to cancer’, Oral science international, 12(1), pp. 15–21.

Kato, S. et al. (2015) ‘Human Papillomavirus in Oral Lichen Planus of Japanese Patients’, Journal of hard tissue biology, 24(2), pp. 181–188.

Kaur, J. and Jacobs, R. (2015) ‘Proinflammatory cytokine levels in oral lichen planus, oral leukoplakia, and oral submucous fibrosis.’, Journal of the Korean Association of Oral and Maxillofacial Surgeons, 41(4), pp. 171–175.

Kaur, J., Politis, C. and Jacobs, R. (2015) ‘Salivary apoptotic cells in oral (pre-) cancer as a potential diagnostic means.’, Journal of clinical and experimental dentistry, 7(3), pp. e400--4.

Kaur, J., Politis, C., & Jacobs, R. (2016) ‘Salivary 8-hydroxy-2-deoxyguanosine, malondialdehyde, vitamin C, and vitamin E in oral pre-cancer and cancer: diagnostic value and free radical mechanism of action’, Clinical oral investigations, 20(2), pp. 315–319.

Kazancioglu, H. O. and Erisen, M. (2015) ‘Comparison of Low-Level Laser Therapy versus Ozone Therapy in the Treatment of Oral Lichen Planus.’, Annals of dermatology, 27(5), pp. 485–491.

Kazanowska-Dygdala, M. et al. (2016) ‘The presence of Helicobacter pylori in oral cavities of patients with leukoplakia and oral lichen planus’, Journal of applied oral science : revista FOB, 24(1), pp. 18–23.

Ke, Y. et al. (2017) ‘Semaphrin4D drives CD8(+) T cells skin trafficking in oral lichen planus via CXCL9 and CXCL10 upregulations in oral keratinocytes’, Journal of Investigative Dermatology, 137(5), pp. S3–S3.

Khamaysi, Z., Bergman, R. and Weltfriend, S. (2006) ‘Positive patch test reactions to allergens of the dental series and the relation to the clinical presentations’, Contact dermatitis, 55(4), pp. 216–218.

Khattab, F. M., & Samir, M. A. (2020). Measurement of squamous cell carcinoma antigen 2 in lichen planus patients. Journal of Cosmetic Dermatology, 19(7), 1780–1784.

Khoo, S. P. et al. (2001) ‘Nuclear and cellular volumetric alterations in oral lichen planus and lichenoid lesions: a histomorphometric study.’, Journal of oral science, 43(3), pp. 151–157.

Kia, S. J. et al. (2015) 'Comparative Efficacy of Topical Curcumin and Triamcinolone for Oral Lichen Planus: A Randomized, Controlled Clinical Trial.', Journal of dentistry (Tehran, Iran), 12(11), pp. 789–796.

Kilpi, A. et al. (1996) 'Studies of the inflammatory process and malignant potential of oral mucosal lichen planus.', Australian dental journal, 41(2), pp. 87–90.

Kilpi, A. M. (1987). Activation marker analysis of mononuclear cell infiltrates of oral lichen planus in situ. Scandinavian Journal of Dental Research, 95(2), 174–180.

Kim, J. et al. (2001) 'Evaluation of premalignant potential in oral lichen planus using interphase cytogenetics', Journal of Oral Pathology and Medicine, 30(2), pp. 65–72.

Kim, T.-W. et al. (2015) 'Patch Testing with Dental Screening Series in Oral Disease.', Annals of dermatology, 27(4), pp. 389–393.

Kimkong, I. et al. (2011) 'Tumour necrosis factor-alpha gene polymorphisms and susceptibility to oral lichen planus', Oral Diseases, 17(2), pp. 206–209.

Kimkong, I., Nakkuntod, J., Sodsai, P., Hirankarn, N., & Kitkumthorn, N. (2012). Association of interferon-gamma gene polymorphisms with susceptibility to oral lichen planus in the Thai population. Archives of Oral Biology, 57(5), 491–494.

Kirby, A. C. et al. (1995) 'Expression of lymphocyte function-associated antigen 3 in oral lichen planus.', Oral diseases, 1(4), pp. 193–197.

Kis, A., Feher, E., Gall, T., Tar, I., Boda, R., Toth, E. D., Mehes, G., Gergely, L., & Szarka, K. (2009). Epstein-Barr virus prevalence in oral squamous cell cancer and in potentially malignant oral disorders in an eastern Hungarian population. European Journal of Oral Sciences, 117(5), 536–540.

Kitkhajornkiat, A et al. (2020). The expression of Cathepsin L in oral lichen planus. Journal of oral biology and craniofacial research, 10(3), 281–286.

Kłosek, S. K. et al. (2011) 'Cigarette smoking induces overexpression of c-Met receptor in microvessels of oral lichen planus', Archives of Medical Science, 7(4), pp. 706–712.

Koch, F. P. et al. (2011) 'Effectiveness of autofluorescence to identify suspicious oral lesions--a prospective, blinded clinical trial.', Clinical oral investigations, 15(6), pp. 975–982.

Koray, M. et al. (2003) 'The evaluation of anxiety and salivary cortisol levels in patients with oral lichen planus', Oral Diseases, 9(6), pp. 298–301.

Kordbacheh, F., Bhatia, N., & Farah, C. S. (2016). Patterns of differentially expressed genes in oral mucosal lesions visualised under autofluorescence (VELscopeTM). Oral Diseases, 22(4), 285–296.

Kragelund, C. et al. (2013) 'Oral candidosis in lichen planus: The diagnostic approach is of major therapeutic importance', Clinical Oral Investigations, 17(3), pp. 957–965.

Kragelund, C. et al. (2014) 'Subgrouping of patients with oral lichen planus according to cytochrome P450 enzyme phenotype and genotype', Oral surgery, oral medicine, oral pathology and oral radiology, 118(4), pp. 469–474.

Kragelund, C. et al. (2019) 'The oral microbiome in oral lichen planus during a 1-year randomized clinical trial', Oral Diseases, 25(1), pp. 327–338.

Kragelund, C., Hansen, C., Reibel, J., Nauntofte, B., Broesen, K., Pedersen, A. M. L., Smidt, D., Eiberg, H., & Torpet, L. A. (2009). Polymorphic drug metabolizing CYP-enzymes - A pathogenic factor in oral lichen planus? Journal of Oral Pathology and Medicine, 38(1), 63–71.

Kraivaphan, P. et al. (2007) 'Frequency of micronucleated exfoliated cells in oral lichen planus.', Journal of dental research, 627(2), pp. 191–196.

Krauss, E. et al. (2011) 'MAGE-A antigens in lesions of the oral mucosa', Clinical Oral Investigations, 15(3), pp. 315–320.

Krogh, P. et al. (1987) 'Yeast species and biotypes associated with oral leukoplakia and lichen planus.', Oral surgery, oral medicine, and oral pathology, 63(1), pp. 48–54.

Kroona, L. et al. (2018) 'Carvone Contact allergy in Sou hero Sweden: a 21-year retrospective Study', Acta dermato-venereologica, 98(10), pp. 938–942.

Küçükkolbas, H. et al. (2011) 'Determination of defensin HNP-1 in human saliva of patients with oral mucosal diseases', Journal of Immunoassay and Immunochemistry, 32(4), pp. 284–295.

Kujundzic, B. et al. (2016) 'Association of vdr, cyp27b1, cyp24a1 and mthfr gene polymorphisms with oral lichen planus risk', Clinical oral investigations, 20(4), pp. 781–789.

Kulkarni, G. et al. (2016) 'Expression of CD1a by Langerhan's cells in oral lichen planus - A retrospective analysis', Journal of Clinical and Diagnostic Research, 10(6), pp. ZC28--ZC31.

Kulthanan, K. et al. (2007) 'Direct immunofluorescence study in patients with lichen planus.', International journal of dermatology, 46(12), pp. 1237–1241.

Kun, J. et al. (2017) 'TRPA1 receptor is upregulated in human oral lichen planus', Oral Diseases, 23(2), pp. 189–198.

Kunz, M. et al. (2016) 'Efficacy and safety of oral alitretinoin in severe oral lichen planus - Results of a prospective pilot study', Journal of the European Academy of Dermatology and Venereology, 30(2), pp. 293–298.

Kuo, R.-C. et al. (2013) 'Prompt healing of erosive oral lichen planus lesion after combined corticosteroid treatment with locally injected triamcinolone acetonide plus oral prednisolone', Journal of the Formosan Medical Association, 112(4), pp. 216–220.

L, B. S. et al. (2020) 'Impairment of myocardial functions and arterial stiffness in patients with lichen planus.', Anais brasileiros de dermatologia, 95(2), pp. 180–186.

L, D. et al. (2020) ‘Does the Clinical Form of Oral Lichen Planus (OLP) Influence the Oral Health-Related Quality of Life (OHRQoL)?’, International journal of environmental research and public health, 17(18).

L., C., Z., L. and Q., M. (1999) ‘The study on erythrocyte immunity function and pathogenesis of patients with oral lichen planus’, Journal of Xi’an Medical University, Chinese Edition, 20(2), pp. 232--233+247.

L., L. et al. (2014) ‘Foxp3 gene expression in oral lichen planus: A clinicopathological study’, Molecular Medicine Reports, 9(3), pp. 928–934.

L., L. R. M. et al. (2010) ‘Effect of desquamative gingivitis on periodontal status: A pilot study’, Oral Diseases, 16(1), pp. 102–107.

L., M. et al. (2013) ‘Bmi1 expression in oral lichen planus and the risk of progression to oral squamous cell carcinoma’, Annals of Diagnostic Pathology, 17(4), pp. 327–330.

L., M. M. et al. (2014) ‘p16(INK4) expression is not associated with human papillomavirus in oral lichen planus’, Oral surgery, oral medicine, oral pathology and oral radiology, 118(6), pp. 694–702.

L., M. M. et al. (2015) ‘DNA methylation analysis by bisulfite next-generation sequencing for early detection of oral squamous cell carcinoma and high-grade squamous intraepithelial lesion from oral brushing’, Journal of Cranio-Maxillofacial Surgery, 43(8), pp. 1494–1500.

L., R. et al. (2008) ‘A comparative treatment study of topical tacrolimus and clobetasol in oral lichen planus.’, Oral surgery, oral medicine, oral pathology, oral radiology, and endodontics, 105(2), pp. 187–193.

L., S. et al. (2012) ‘Definitive and differential diagnosis of desquamative gingivitis through direct immunofluorescence studies’, Journal of Periodontology, 83(10), pp. 1270–1278.

L., S. et al. (2013) ‘CD133 expression in oral lichen planus correlated with the risk for progression to oral squamous cell carcinoma’, Annals of Diagnostic Pathology, 17(6), pp. 486–489.

L., W. et al. (2008) ‘Clinical and microbiological study of compound of light yellow Sophora root collutory on treatment of oral lichen planus’, Shanghai kou qiang yi xue = Shanghai journal of stomatology, 17(2), pp. 118–120.

L., W. et al. (2018) ‘Microrna microarray-based identification of involvement of mir-155 and mir-19a in development of oral lichen planus (OLP) by modulating th1/th2 balance via targeting enos and toll-like receptor 2 (TLR2)’, Medical Science Monitor, 24, pp. 3591–3603.

L., W. et al. (2019) ‘MiR-122 and miR-199 synergistically promote autophagy in oral lichen planus by targeting the Akt/mTOR pathway’, International Journal of Molecular Medicine, 43(3), pp. 1373–1381.

L.A., G. et al. (2012) ‘Increased number of Langerhans cells in oral lichen planus and oral lichenoid lesions’, Oral Surgery, Oral Medicine, Oral Pathology and Oral Radiology, 113(5), pp. 661–666.

L.J., M. et al. (2015) ‘Direct immunofluorescence testing results in cases of premalignant and malignant oral lesions’, Oral surgery, oral medicine, oral pathology and oral radiology, 119(6), pp. 675–683.

L.J., S. et al. (2004) ‘Expressions of Fas/FasL and granzyme B in oral lichen planus and their significance’, Di 1 jun yi da xue xue bao = Academic journal of the first medical college of PLA, 24(12), pp. 1362–1366.

L.J., W. et al. (1989) ‘Expression of CDw29 and CD45R antigens on epithelial cells in oral lichen planus’, Journal of Oral Pathology and Medicine, 18(6), pp. 360–365.

L.J., W. et al. (1997) ‘Cutaneous lymphocyte associated antigen (CLA) and alpha e beta 7 integrins are expressed by mononuclear cells in skin and oral lichen planus’, Journal of Oral Pathology and Medicine, 26(9), pp. 402–407.

L.N., R. et al. (2017) ‘Allelic loss in amalgam-associated oral lichenoid lesions compared to oral lichen planus and mucosa’, Oral Diseases, 23(4), pp. 471–476.

L.P., S. et al. (2002) ‘Prevalence of Epstein-Barr virus in oral squamous cell carcinoma, oral lichen planus, and normal oral mucosa.’, Oral surgery, oral medicine, oral pathology, oral radiology, and endodontics, 93(5), pp. 586–592.

L.R., E. et al. (1984) ‘The role of dental restorative metals in the pathogenesis of oral lichen planus’, Oral Surgery Oral Medicine and Oral Pathology, 57(4), pp. 383–387.

L.S., M. et al. (2015) ‘Outcomes of invitational and opportunistic oral cancer screening initiatives in Oporto, Portugal’, Journal of Oral Pathology and Medicine, 44(2), pp. 145–152.

Laeijendecker, R. et al. (2005) ‘Premalignant nature of oral lichen planus’, Acta Dermato-Venereologica, 85(6), pp. 516–520.

Lage, D., Pimentel, V. N., Soares, T. C. B., Souza, E. M., Metze, K., & Cintra, M. L. (2011). Perforin and granzyme B expression in oral and cutaneous lichen planus - a comparative study. Journal of Cutaneous Pathology, 38(12), 973–978.

Lajevardi, V et al. (2016) ‘Behandlung von erosivem oralem Lichen planus mit Methotrexat’, JDDG - Journal of the German Society of Dermatology, 14(3), pp. 286–294.

Lajevardi, Vahideh et al. (2016) ‘Treatment of erosive oral lichen planus with methotrexate’, JDDG - Journal of the German Society of Dermatology, 14(3), pp. 286–293.

Lamey, P. J. et al. (1995) ‘Basal cell cytoplasmic autoantibodies in oral lichenoid reactions.’, Oral surgery, oral medicine, oral pathology, oral radiology, and endodontics, 79(1), pp. 44–49.

Larsen, K. R., Johansen, J. D., Reibel, J., Zachariae, C. and Pedersen, A. M. L. (2017) ‘Serum cytokine profile and clinicopathological findings in oral lichen planus, oral lichenoid lesions and stomatitis.’, Clinical and experimental dental research, 3(6), pp. 220–226.

Larsen, K. R., Johansen, J. D., Reibel, J., Zachariae, C., Rosing, K., et al. (2017) ‘Oral symptoms and salivary findings in oral lichen planus, oral lichenoid lesions and stomatitis’, BMC oral health, 17(1), p. 103.

Larsson, A. and Warfvinge, G. (1995) ‘The histopathology of oral mucosal lesions associated with amalgam or porcelain-fused-to-metal restorations.’, Oral diseases, 1(3), pp. 152–158.

Larsson, A. and Warfvinge, G. (1998) ‘Immunohistochemistry of “tertiary lymphoid follicles” in oral amalgam-associated lichenoid lesions.’, Oral diseases, 4(3), pp. 187–193.

Laskaris, G. et al. (1982) ‘Direct immunofluorescence in oral lichen planus’, Oral Surgery Oral Medicine and Oral Pathology, 53(5), pp. 483–487.

Lavaee, F. and Shadmanpour, M. (2019) ‘Comparison of the effect of photodynamic therapy and topical corticosteroid on oral lichen planus lesions.’, Oral diseases.

Lavaee, F. et al. (2018) ‘The evaluation of the serum level of IL-10 in OLP patients’, Comparative Clinical Pathology, 27(1), pp. 131–134.

Lavaee, F., Nazhvani, A. D. and Razavi, N. (2018) ‘Cytomorphometric analysis of exfoliated cells in patients with oral lichen planus’, Comparative Clinical Pathology, 27(4), pp. 1073–1077.

Lee, C. H., Ko, A. M., Warnakulasuriya, S., Yin, B. L., Sunarjo, Zain, R. B., Ibrahim, S. O., Liu, Z. W., Li, W. H., Zhang, S. S., Kuntoro, Utomo, B., Rajapakse, P. S., Warusavithana, S. A., Razak, I. A., Abdullah, N., Shrestha, P., Kwan, A. L., Shieh, T. Y., Chen, M. K., … Ko, Y. C. (2011). Intercountry prevalences and practices of betel-quid use in south, southeast and eastern Asia regions and associated oral preneoplastic disorders: an international collaborative study by Asian betel-quid consortium of south and east Asia. International journal of cancer, *129*(7), 1741–1751.

Lee, J.-J. et al. (2005) ‘Higher expressions of p53 and proliferating cell nuclear antigen (PCNA) in atrophic oral lichen planus and patients with areca quid chewing’, Oral Surgery, Oral Medicine, Oral Pathology, Oral Radiology, and Endodontics, 99(4), pp. 471–478.

Lee, Y. C. et al. (2013) ‘Intralesional injection versus mouth rinse of triamcinolone acetonide in oral lichen planus: A randomized controlled study’, Otolaryngology - Head and Neck Surgery (United States), 148(3), pp. 443–449.

Lei, L. et al. (2008) ‘[Expression of TGF-beta receptors in CD8+ T cells of oral lichen planus].’, Zhonghua kou qiang yi xue za zhi = Zhonghua kouqiang yixue zazhi = Chinese journal of stomatology, 43(2), pp. 99–100.

Leyva-Huerta, E.-R., Ledesma-Montes, C., Rojo-Botello, R.-E., & Vega-Memije, E. (2012). P53 and bcl-2 immunoexpression in patients with oral lichen planus and oral squamous cell carcinoma. Medicina Oral, Patologia Oral y Cirugia Bucal, 17(5), e745--e750.

Li, J. et al. (2011) 'Antifungal susceptibility test of genotypes of Candida albicans from patients with atrophic or erosive oral lichen planus', Shanghai kou qiang yi xue = Shanghai journal of stomatology, 20(3), pp. 300–303.

Li, M. and He, S.-L. (2013) 'Reliability and validity of the Chinese version of the chronic oral mucosal diseases questionnaire.', Journal of oral pathology & medicine, 42(2), pp. 194–199.

Li, T.-J. et al. (2013) 'COX-2, MMP-7 expression in oral lichen planus and oral squamous cell carcinoma', Asian Pacific Journal of Tropical Medicine, 6(8), pp. 640–643.

Li, X. X. et al. (2015) 'The expression and changes of apoptosis protein Bcl-2 and Bax in oral lichen planus', Shanghai kou qiang yi xue = Shanghai journal of stomatology, 24(4), pp. 465–469.

Li, X.-Z. et al. (2017) 'Urine metabolic profiling for the pathogenesis research of erosive oral lichen planus.', Archives of oral biology, 73, pp. 206–213.

Li, Y. et al. (2019) 'Salivary mycobiome dysbiosis and its potential impact on bacteriome shifts and host immunity in oral lichen planus', International journal of oral science, 11(2), p. 13.

Liang, J. et al. (2016) 'Correlation of miRNA-155 and IL-17 mRNA expression in peripheral blood of female patients with oral lichen planus', International journal of clinical and experimental pathology, 9(10), pp. 10569–10574.

Likar-Manookin, K. et al. (2013) 'Prevalence of oral lesions of autoimmune etiology in patients with primary Sjogren's syndrome.', Oral diseases, 19(6), pp. 598–603.

Lin H-P, Wang Y-P, Chia J-S and Sun A (2011), "Modulation of serum antinuclear antibody levels by levamisole treatment in patients with oral lichen planus", Journal of the Formosan Medical Association. Vol. 110(5), pp. 316-321.

Lin, H-P et al. (2011) 'Modulation of serum gastric parietal cell antibody level by levamisole and vitamin B12 in oral lichen planus', Oral Diseases, 17(1), pp. 95–101.

Lin, Hung-Pin et al. (2011) 'Modulation of serum anti-thyroglobulin and anti-thyroid microsomal autoantibody levels by levamisole in patients with oral lichen planus', Journal of the Formosan Medical Association, 110(3), pp. 169–174.

Lin, L. et al. (2005) 'Comparative observation on the effects of Radix Tripterygium hypoglaucum tablet and tripterygium glycosides tablet in treating erosive oral lichen planus', Chinese Journal of Integrative Medicine, 11(2), pp. 149–150.

Lin, S. C. et al. (1988) 'Subsets of T lymphocytes in peripheral blood of patients with oral lichen planus', International Journal of Oral and Maxillofacial Surgery, 17(2), pp. 84–86.

Lin, S. C. et al. (1990) ‘HLA-DR and DQ antigens in Chinese patients with oral lichen planus’, Journal of Oral Pathology and Medicine, 19(7), pp. 298–300.

Lind, P. O. et al. (1986) ‘Amalgam-related oral lichenoid reaction.’, Scandinavian journal of dental research, 94(5), pp. 448–451.

Lipperheide, V. et al. (1996) ‘Candida biotypes in patients with oral leukoplakia and lichen planus. Candida biotypes in leukoplakia and lichen planus.’, Mycopathologia, 134(2), pp. 75–82.

Lisa Cheng, Y. S. et al. (2014) ‘Salivary interleukin-6 and -8 in patients with oral cancer and patients with chronic oral inflammatory diseases’, Journal of periodontology, 85(7), pp. 956–965.

Liu H.W. (1994), "Research on malignant changes in oral lichen planus", Zhonghua kou qiang yi xue za zhi = Zhonghua kouqiang yixue zazhi = Chinese journal of stomatology. H.W. Liu Vol. 29(3), pp. 167-169.

Liu, C. et al. (2013) ‘Efficacy of intralesional betamethasone for erosive oral lichen planus and evaluation of recurrence: A randomized, controlled trial’, Oral Surgery, Oral Medicine, Oral Pathology and Oral Radiology, 116(5), pp. 584–590.

Liu, J. et al. (2018) ‘Candida albicans induces TLR2/MyD88/NF-kappa B signaling and inflammation in oral lichen planus-derived keratinocytes’, Journal of infection in developing countries, 12(9), pp. 780–786.

Liu, L.-J. et al. (2012) ‘Generic and oral quality of life is affected by oral mucosal diseases.’, BMC oral health, 12, p. 2.

Liu, Q. et al. (2015) ‘Genes involved in keratinization, keratinocyte and epithelium differentiation are aberrantly regulated in oral lichen planus’, Genes & genomics, 37(9), pp. 751–757.

Liu, T. et al. (2018) ‘Study on expression of p16 and human papillomavirus 16 and 18 (E6) in OLP and its malignant transformation’, Pathology Research and Practice, 214(2), pp. 296–302.

Liu, W.-Z. et al. (2014) ‘Interferon-gamma and interleukin-4 detected in serum and saliva from patients with oral lichen planus.’, International journal of oral science, 6(1), pp. 22–26.

Liu, Y. et al. (2011) ‘[Levels of interlukin-6 and tumor necrosis factor-alpha in saliva of patients with type 2 diabetes mellitus and oral lichen planus].’, Beijing da xue xue bao. Yi xue ban = Journal of Peking University. Health sciences, 43(4), pp. 596–599.

Liu, Y. et al. (2016) ‘SOX4 promotes progression in OLP-associated squamous cell carcinoma’, Journal of Cancer, 7(11), pp. 1534–1540.

Liu, Z. W., Lin, T. N., & He, G. Z. (2000) ‘Research of compound cyclosporin a mouthwash in the treatmentof oral lichen planus’, Bulletin of Hunan Medical University, 25(2), pp. 183–184.

Lo Muzio, L. et al. (2001) 'The treatment of oral aphthous ulceration or erosive lichen planus with topical clobetasol propionate in three preparations: a clinical and pilot study on 54 patients.', Journal of oral pathology & medicine: official publication of the International Association of Oral Pathologists and the American Academy of Oral Pathology, 30(10), pp. 611–617.

Lodi, G. et al. (2000) 'Hepatitis C virus-associated oral lichen planus: No influence from hepatitis G virus co-infection', Journal of Oral Pathology and Medicine, 29(1), pp. 39–42.

Lodi, G., Olsen, I., Piattelli, A., D'Amico, E., Artese, L., & Porter, S. R. (1997). Antibodies to epithelial components in oral lichen planus (OLP) associated with hepatitis C virus (HCV) infection. Journal of Oral Pathology and Medicine, 26(1), 36–39.

Lombardi, T. et al. (2001) 'Absence of leukocyte microchimerism in oral lichen planus (OLP): An in situ hybridisation study', Journal of Oral Pathology and Medicine, 30(7), pp. 398–401.

Loncar-Brzak, B. et al. (2018) 'Expression of small leucine-rich extracellular matrix proteoglycans biglycan and lumican reveals oral lichen planus malignant potential', Clinical oral investigations, 22(2), pp. 1071–1082.

Lopez Jornet, P. et al. (2016) 'Efficacy of topical chamomile management vs. placebo in patients with oral lichen planus: a randomized double-blind study', Journal of the European Academy of Dermatology and Venereology, 30(10), pp. 1783–1786.

López López, J., & Roselló Llabrés, X. (1995) 'Cyclosporine A, an alternative to the oral lichen planus erosive treatment.', Bulletin du Groupement international pour la recherche scientifique en stomatologie & odontologie, 38(1), pp. 33–38.

López-Jornet, P. et al. (2004) 'The clinicopathological characteristics of oral lichen planus and its relationship with dental materials', Contact Dermatitis, 51(4), pp. 210–211.

López-Jornet, P. et al. (2012) 'Periodontal conditions in patients with oral lichen planus: a pilot study.', Quintessence international (Berlin, Germany : 1985), 43(2), pp. 147–152.

Lopez-Jornet, P. et al. (2014) 'Dental implants in patients with oral lichen planus: a cross-sectional study', Clinical implant dentistry and related research, 16(1), pp. 107–115.

López-Jornet, P. et al. (2016) 'Oral lichen planus: Salival biomarkers cortisol, immunoglobulin A, adiponectin', Journal of Oral Pathology and Medicine, 45(3), pp. 211–217.

López-Jornet, P., Camacho-Alonso, F, et al. (2010) 'Clinical assessment of oral lichen planus based on different scales', International Journal of Dermatology, 49(3), pp. 272–275.

López-Jornet, P., Camacho-Alonso, F., & Lucero Berdugo, M. (2009). Measuring the impact of oral mucosa disease on quality of life. European Journal of Dermatology, 19(6), pp. 603–606.

López-Jornet, P., Camacho-Alonso, F., & Molina-Miñano, F. (2009). Quantitative analysis of epithelial papillae in patients with oral lichen planus. Journal of the European Academy of Dermatology and Venereology, 23(6), pp. 692–696.

López-Jornet, P., Camacho-Alonso, Fabio, et al. (2010) 'Quality of life in patients with oral lichen planus', Journal of Evaluation in Clinical Practice, 16(1), pp. 111–113.

Lopez-Jornet, P., Martinez-Canovas, A. and Pons-Fuster, A. (2014) 'Salivary biomarkers of oxidative stress and quality of life in patients with oral lichen planus.', Geriatrics & gerontology international, 14(3), pp. 654–659.

Lopez-Jornet, P., Martinez-Canovas, A., & Pons-Fuster, A. (2014). Salivary biomarkers of oxidative stress and quality of life in patients with oral lichen planus. Geriatrics and Gerontology International, 14(3), pp. 654–659.

Lowental, U. et al. (1984) 'Oral lichen planus according to the modern medical model.', Journal of oral medicine, 39(4), pp. 224–226.

Lozada-Nur, F. I. et al. (2006) 'Tacrolimus powder in Orabase 0.1% for the treatment of oral lichen planus and oral lichenoid lesions: An open clinical trial', Oral Surgery, Oral Medicine, Oral Pathology, Oral Radiology, and Endodontics, 102(6), pp. 744–749.

Lozada-Nur, F., Miranda, C. and Maliksi, R. (1994) 'Double-blind clinical trial of 0.05% clobetasol propionate (corrected from proprionate) ointment in orabase and 0.05% fluocinonide ointment in orabase in the treatment of patients with oral vesiculoerosive diseases.', Oral surgery, oral medicine, and oral pathology, 77(6), pp. 598–604.

Lu, C. et al. (2017) 'Detection of AMA-M2 in human saliva: Potentials in diagnosis and monitoring of primary biliary cholangitis', Scientific reports, 7(1), p. 796.

Lu, R. et al. (2011) 'Expression of T-bet and GATA-3 in peripheral blood mononuclear cells of patients with oral lichen planus', Archives of Oral Biology, 56(5), pp. 499–505.

Lu, S.-L. et al. (2016) 'Clinical characteristics and analysis of familial oral lichen planus in eight Chinese families', Experimental and Therapeutic Medicine, 12(4), pp. 2281–2284.

Lu, S.-Y. et al. (2009) 'Increased oral lichen planus in a chronic hepatitis patient associated with elevated transaminase levels before and after interferon/ribavirin therapy', Journal of dental sciences, 4(4), pp. 191–197.

Lu, S.-Y. et al. (2019) 'Treatment effectiveness of levamisole plus prednisolone on oral lichen planus patients with emphasis on levamisole-induced agranulocytosis or pancytopenia', Journal of the Formosan Medical Association, 118(8), pp. 1193–1201.

Lucchese, A., Gentile, E., Capone, G., De Vico, G., Serpico, R., & Landini, G. (2015). Fractal analysis of mucosal microvascular patterns in oral lichen planus: a preliminary study. Oral Surgery, Oral Medicine, Oral Pathology and Oral Radiology, 120(5), 609–615.

Luengtrakoon, K. et al. (2017) ‘Increased melatonin in oral mucosal tissue of oral lichen planus (OLP) patients: A possible link between melatonin and its role in oral mucosal inflammation’, Archives of oral biology, 78, pp. 13–19.

Lukac, J. et al. (2003) ‘Phagocytic functions of salivary neutrophils in oral mucous membrane diseases’, Journal of Oral Pathology and Medicine, 32(5), pp. 271–274.

Lukač, J. et al. (2006) ‘Serum autoantibodies to desmogleins 1 and 3 in patients with oral lichen planus’, Croatian Medical Journal, 47(1), pp. 53–58.

Lundquist, G. et al. (1995) ‘Photochemotherapy of oral lichen planus. A controlled study.’, Oral surgery, oral medicine, oral pathology, oral radiology, and endodontics, 79(5), pp. 554–558.

Lundstrom, I. M. (1984). Allergy and corrosion of dental materials in patients with oral lichen planus. International Journal of Oral Surgery, 13(1), 16–24.

M, Amirchaghmaghi, R, M., et al. (2020) ‘Assessment of salivary thioredoxin levels in oral lichen planus and oral squamous cell carcinoma.’, Clinical and experimental dental research.

M, Bakhshi, S, G., et al. (2020) ‘Combination Therapy with 1% Nanocurcumin Gel and 0.1% Triamcinolone Acetonide Mouth Rinse for Oral Lichen Planus: A Randomized Double-Blind Placebo Controlled Clinical Trial.’, Dermatology research and practice, 2020, p. 4298193.

M, D., SA, T. and B, I. (2020) ‘The relationship between lichen planus and metabolic syndrome.’, Journal of cosmetic dermatology.

M, Mardani, S, T. A., et al. (2020) ‘Serum Levels of IL-22 in Patients with Oral Lichen Planus and Cutaneous Lichen Planus.’, Journal of dentistry (Shiraz, Iran), 21(4), pp. 330–334.

M, Popovska, A, A.-S., et al. (2020) ‘Oral Lichen Planus - Related Connection with HLA-System Antigens.’, Prilozi (Makedonska akademija na naukite i umetnostite. Oddelenie za medicinski nauki), 41(1), pp. 65–77.

M, Samimi et al. (2020) ‘Topical rapamycin versus betamethasone dipropionate ointment for treating oral erosive lichen planus: a randomized, double-blind, controlled study.’, Journal of the European Academy of Dermatology and Venereology, 34(10), pp. 2384–2391.

M, Shteiner et al. (2020) ‘Submucosal Fibrotic Bands in Oral Lichen Planus: A Clinico-Pathological Investigation of a Newly Described Phenomenon.’, Head and neck pathology.

M., B. et al. (2016) ‘Evaluation of mast cell distribution in oral lichen planus and lichenoid lesions by immunohistochemical and histochemical analysis’, American Journal of Immunology, 12(4), pp. 99–106.

M., B.-B. and I., P. (2001) ‘The prevalence of precancerous oral lesions. Oral lichen planus’, Archive of Oncology, 9(2), pp. 107–109.

M., C. C. et al. (2012) ‘Clinical and histological features of gingival lesions: A 17-year retrospective analysis in a northern Italian population’, Medicina Oral, Patologia Oral y Cirugia Bucal, 17(4), pp. 555–561.

M., D. et al. (2014) ‘Langerhans cells in lichen planus and lichenoid mucositis an immunohistochemical study’, Journal of Pharmacy and Bioallied Sciences, 6, pp. S146--S149.

M., E. et al. (2007) ‘A scoring system for mucosal disease severity with special reference to oral lichen planus’, British Journal of Dermatology, 157(4), pp. 765–770.

M., I. et al. (1997) ‘Oral lichen planus is not associated with IgG circulating antibodies to epithelial antigens.’, Oral surgery, oral medicine, oral pathology, oral radiology, and endodontics, 84(2), pp. 175–178.

M., I. et al. (2006) ‘Oral lichen planus: A retrospective study of 690 British patients’, Oral Diseases, 12(5), pp. 463–468.

M., J. et al. (1986) ‘Mast cells in oral lichen planus.’, Journal of oral pathology, 15(5), pp. 273–275.

M., J. et al. (1990) ‘Human papilloma virus in erosive oral lichen planus’, Journal of Oral Pathology and Medicine, 19(6), pp. 273–277.

M., K. et al. (2015) ‘Molecular targeting of her-2/neu protein is not recommended as an adjuvant therapy in oral squamous cell carcinoma and oral lichen planus’, Advanced Pharmaceutical Bulletin, 5, pp. 649–652.

M., L. et al. (2015) ‘Difficulties detecting miRNA-203 in human whole saliva by the use of PCR’, Medicina Oral, Patologia Oral y Cirugia Bucal, 20(2), pp. e130--e134.

M., M. et al. (2011) ‘Assessment of frequency of micronucleated exfoliated buccal cells in relation to oxidative stress in oral lichen planus in coastal Karnataka, India’, Asian Pacific Journal of Tropical Disease, 1(4), pp. 287–288.

M., M. et al. (2018) ‘Diagnostic and prognostic relevance of salivary microRNA-21, -125a, -31 and -200a levels in patients with oral lichen planus - a short report’, Cellular Oncology, 41(3), pp. 329–334.

M., M.-S. M. et al. (2014) ‘Clinicopathologic correlation of oral lichen planus and oral lichenoid lesions: A preliminary study’, Scientific World Journal, 2014, p. 746874.

M., Ognjenović, D., K., M., M., et al. (1998) ‘Oral lichen planus and HLA DR.’, Collegium antropologicum, 22, pp. 93–96.

M., Ognjenović, D., K., V.V., C., et al. (1998) ‘Oral lichen planus and HLA A.’, Collegium antropologicum, 22, pp. 89–92.

M., P. C. (1987) ‘Epithelial dysplasia in oral lichen planus’, Anales de la Facultad de Odontología, (24), pp. 19–26.

M., P. et al. (1999) ‘Oral lichen planus versus oral lichenoid eruption as a manifestation of contact allergy’, Contact Dermatitis, 40(6), pp. 333–334.

M., P. et al. (2008) ‘The prevalence of oral mucosal lesions in adults from the Turin area’, Oral Diseases, 14(4), pp. 356–366.

M., P. et al. (2014) ‘Role of humoral mechanisms in etiology of lichen planus’, Prilozi (Makedonska akademija na naukite i umetnostite. Oddelenie za medicinski nauki), 35(3), pp. 185–194.

M., P. et al. (2017) ‘High-resolution DNA content analysis of microbiopsy samples in oral lichen planus’, Oral Diseases, 23(3), pp. 318–323.

M., R. et al. (2009) ‘Correlation between clinical and histopathologic diagnoses of oral lichen planus based on modified WHO diagnostic criteria’, Oral Surgery, Oral Medicine, Oral Pathology, Oral Radiology and Endodontology, 107(6), pp. 796–800.

M., R. et al. (2015) ‘Relevance of psychosocial factors to quality of life in oral cancer and oral lichen planus: a prospective comparative study’, British Journal of Oral and Maxillofacial Surgery, 53(7), pp. 621–626.

M., R.-O. et al. (2018) ‘Psychopathological profile and quality of life of patients with oral lichen planus’, Journal of applied oral science: revista FOB, 26, pp. e20170146–e20170146.

M., R., A.A., K. and M., K.-R. (2000) ‘Reticular form of oral lichen planus. A 19-year observation period in 75 patients from Slovenia’, Acta Dermatovenerologica Alpina, Panonica et Adriatica, 9(4), pp. 137–141.

M., S. et al. (1983) ‘Autoantibodies in patients with oral lupus erythematosus, lichen planus and leukoplakia, an aid in diagnosis’, Acta Pathologica Microbiologica et Immunologica Scandinavica - Section C Immunology, 91(1), pp. 59–63.

M., S. et al. (1991) ‘Effect of diethyldithiocarbamate on interleukin 2 production of patients with oral lichen planus and its clinical therapeutic efficacy’, Acta Pharmacologica Sinica, 12(4), pp. 378–380.

M., S. et al. (2014) ‘Effect of cedar honey in the treatment of oral lichen planus’, Iranian Journal of Otorhinolaryngology, 26(76), pp. 151–161.

M., S. et al. (2015) ‘Altered expression of hyaluronan, HAS1-2, and HYAL1-2 in oral lichen planus’, Journal of Oral Pathology and Medicine, 44(6), pp. 401–409.

M., S. et al. (2016) ‘Image-based DNA ploidy analysis aids prediction of malignant transformation in oral lichen planus’, Oral surgery, oral medicine, oral pathology and oral radiology, 121(6), pp. 643–650.

M., S. et al. (2017) ‘A clinical evaluation of the efficacy of photodynamic therapy in the treatment of erosive oral lichen planus: A case series’, Photodiagnosis and Photodynamic Therapy, 18, pp. 12–19.

M., Schifter et al. (1998) 'Epithelial p53 gene expression and mutational analysis, combined with growth fraction assessment, in oral lichen planus', Journal of Oral Pathology and Medicine, 27(7), pp. 318–324.

M., Sutinen et al. (1998) 'Expression of matrix metalloproteinases (MMP-1 and -2) and their inhibitors (TIMP-1, -2 and -3) in oral lichen planus, dysplasia, squamous cell carcinoma and lymph node metastasis', British Journal of Cancer, 77(12), pp. 2239–2245.

M., T. et al. (2004) 'Low-Dose Excimer 308-nm Laser for the Treatment of Oral Lichen Planus', Archives of Dermatology, 140(4), pp. 415–420.

M., T. et al. (2011) 'Scanning electron microscopic study of surface epithelial cells in erosive and nonerosive oral lichen planus', Journal of Contemporary Dental Practice, 12(6), pp. 463–468.

M., V. D. et al. (2002) 'Oral lichen planus: immunohistology of mucosal lesions.', Journal of oral pathology & medicine, 31(7), pp. 410–414.

M., V. et al. (2013) 'Inflammatory cells of immunosuppressive phenotypes in oral lichen planus have a proinflammatory pattern of expression and are associated with clinical parameters', Clinical oral investigations, 17(5), pp. 1365–1373.

M., V. et al. (2015) 'Peripheral and local human papillomavirus 16-specific CD8+T-cell expansions characterize erosive oral lichen planus', Journal of Investigative Dermatology, 135(2), pp. 418–424.

M., Y. et al. (2017) 'Myeloid dendritic cells stimulated by thymic stromal lymphopoietin promote Th2 immune responses and the pathogenesis of oral lichen planus', PLoS ONE, 12(3), pp. e0173017–e0173017.

M., Z. et al. (2013) 'Suitability/unsuitability of cell proliferation as an indicator of malignant potential in oral lichen planus: An immunohistochemical study', Asian Pacific Journal of Cancer Prevention, 14(11), pp. 6979–6983.

M., Z., F., B. and A., M. (2018) 'Comparative study of β-catenin and CD44 immunoexpression in oral lichen planus and squamous cell carcinoma', International Journal of Dermatology, 57(7), pp. 794–798.

M.A. et al. (2015) 'TNF-α, TNF-β and IL-10 gene polymorphism and association with oral lichen planus risk in Saudi patients', Journal of applied oral science: revista FOB, 23(3), pp. 295–301.

M.A. et al. (2016) 'Association of genetic polymorphisms in interferon-γ, interleukin-6 and transforming growth factor-β1 gene with oral lichen planus susceptibility', BMC oral health, 16(1), p. 76.

M.Á., P. et al. (2018) 'Diferentes patrones de expresión de la anhidrasa carbónica IX en liquen plano bucal y leucoplasia', Acta odontologica latinoamericana, 31(2), pp. 77–81.

M.C., L. et al. (2003) 'Oral mucosal keratinocytes express RANTES and ICAM-1, but not interleukin-8, in oral lichen planus and oral lichenoid reactions induced by amalgam fillings', Clinical and Experimental Dermatology, 28(1), pp. 64–69.

M.D., M. et al. (2001) ‘Clinical guidelines in early detection of oral squamous cell carcinoma arising in oral lichen planus: A 5-year experience’, Oral Oncology, 37(3), pp. 262–267.

M.D., M. et al. (2003) ‘Oral lichen planus and dental materials: A case-control study’, Contact Dermatitis, 48(6), pp. 331–336.

M.D., M. et al. (2006) ‘Dysplasia/neoplasia surveillance in oral lichen planus patients: A description of clinical criteria adopted at a single centre and their impact on prognosis’, Oral Oncology, 42(8), pp. 819–824.

M.D., M. et al. (2011) ‘Unexplained somatic comorbidities in patients with burning mouth syndrome: a controlled clinical study.’, Journal of orofacial pain, 25(2), pp. 131–140.

M.H., T. et al. (2003) ‘Amalgam-contact hypersensitivity lesions and oral lichen planus.’, Oral surgery, oral medicine, oral pathology, oral radiology, and endodontics, 95(3), pp. 291–299.

M.H., T. et al. (2006) ‘The role of histopathological characteristics in distinguishing amalgam-associated oral lichenoid reactions and oral lichen planus’, Journal of Oral Pathology and Medicine, 35(4), pp. 233–240.

M.J., G.-P. V. and G., H.-Z. (2000) ‘Anxiety as an etiologic factor in oral lichen planus (OLP)’, Medicina Oral, 5(1), pp. 7–13.

M.J., G.-P. V. et al. (2001) ‘Anxiety and depression as risk factors for oral lichen planus’, Dermatology, 203(4), pp. 303–307.

M.L., G.-C. et al. (2013) ‘Production of carcinogenic acetaldehyde by Candida albicans from patients with potentially malignant oral mucosal disorders’, Journal of Oral Pathology and Medicine, 42(3), pp. 243–249.

M.L., P. et al. (1994) ‘The efficacy of cyclosporin for topical use in oral lichen planus’, Minerva stomatologica, 43(4), pp. 129–132.

M.M., B., I., L.-V., et al. (2006) ‘Oral mucosal lesions diagnosed in a stomatology service. An examination of clinico-pathological findings from the year 2003’, Schweizer Monatsschrift für Zahnmedizin = Revue mensuelle suisse d’odonto-stomatologie = Rivista mensile svizzera di odontologia e stomatologia / SSO, 116(5), pp. 468–475.

M.M., B., L., K., et al. (2006) ‘Oral lichen planus and malignant transformation: A retrospective follow-up study of clinical and histopathologic data’, Quintessence International, 37(4), pp. 261–271.

M.M., K. et al. (2020) ‘Efficacy of 1064 Q switched Nd:YAG laser in the treatment of oral lichen planus’, The Journal of dermatological treatment, 31(6), pp. 1–5.

M.M., N. et al. (2018) ‘Bacillus Calmette-Guerin, polysaccharide nucleic acid in the treatment of cutaneous and oral lichen planus’, Dermatologic Therapy, 31(3), pp. e12591–e12591.

M.M., Z. et al. (2010) ‘Immunohistochemical study of syndecan-1 down-regulation and the expression of P35 protein in oral lichen planus: a clinicopathologic correlation with hepatitis C infection in the Egyptian population’, Annals of Diagnostic Pathology, 14(3), pp. 153–161.

M.Z., M. et al. (2018) ‘Effects of Low Level Laser Therapy on Erosive-atrophic Oral Lichen Planus’, Folia medica, 60(3), pp. 417–424.

Ma, J.-M. et al. (2016) ‘Intracellular Ca2+ and related proteins in patients with oral lichen planus’, Immunologic Research, 64(2), pp. 531–539.

Ma, R.-J. et al. (2019) ‘Aberrant IGF1–PI3K/AKT/MTOR signaling pathway regulates the local immunity of oral lichen planus’, Immunobiology, 224(3), pp. 455–461.

Madkour, G. G. et al. (2012) ‘Expression levels of microRNA-21 and microRNA-146a in patients with oral lichen planus’, Life science journal-acta zhengzhou university overseas edition, 9(4), pp. 4666–4670.

Maeda, H. et al. (1994) ‘Keratin staining pattern in clinically normal and diseased oral mucosa of lichen planus patients.’, Scandinavian journal of dental research, 102(4), pp. 210–215.

Magnusson, M. et al. (2004) ‘Basic fibroblast growth factor (bFGF) in saliva and oral mucosa in patients with oral lichen planus: Preliminary observations’, Oral Surgery, Oral Medicine, Oral Pathology, Oral Radiology and Endodontology, 98(3), pp. 324–326.

Mahdi, K. A. (2019) ‘Comparative effect of topical tacrolimus and topical isotretinoin in patients with oral lichen planus’, Indian Journal of Public Health Research and Development, 10(2), pp. 693–697.

Mahmoud, M. M. et al. (2016) ‘Anti-angiogenic therapy (bevacizumab) in the management of oral lichen planus’, European journal of oral sciences, 124(2), pp. 119–126.

Maia, H. C. de M. et al. (2016) ‘Potentially malignant oral lesions: clinicopathological correlations’, Einstein (Sao Paulo, Brazil), 14(1), pp. 35–40.

Maidhof, R. (1979) ‘[Systemic treatment of oral lichen planus with an aromatic retinoid (Ro 10-9359)].’, Zeitschrift fur Hautkrankheiten, 54(19), pp. 816–873.

Malarkodi, T. and Sathasivasubramanian, S. (2015) ‘Quantitative Analysis of Salivary TNF-alpha in Oral Lichen Planus Patients.’, International journal of dentistry, 2015, p. 283465.

Maldonado Cid, P. et al. (2020) ‘Frontal Fibrosing Alopecia: A Retrospective Study of 75 Patients.’, Actas dermo-sifiliograficas, 111(6), pp. 487–495.

Malekzadeh, H. et al. (2015) ‘Salivary Interferon Gamma and Interleukin-4 Levels in Patients Suffering from Oral Lichen Planus.’, Cell journal, 17(3), pp. 554–558.

Malhotra, A. K. et al. (2008) ‘Betamethasone oral mini-pulse therapy compared with topical triamcinolone acetonide (0.1%) paste in oral lichen planus: A randomized

comparative study', Journal of the American Academy of Dermatology, 58(4), pp. 596–602.

Malik, U. et al. (2012) 'Treatment of symptomatic oral lichen planus (OLP) with 0.1% tacrolimus powder in Oraguard-B - A pilot prospective study', Saudi Dental Journal, 24(3), pp. 143–148.

Maloth, A. K. et al. (2015) 'A Comparative lmmunohistochemical Analysis of Langerhans Cells in Oral Mucosa, Oral Lichen Planus and Oral Squamous Cell Carcinoma', Journal of Clinical and Diagnostic Research, 9(7), pp. 76–79.

Mane, D. R., Kale, A. D. and Belaldavar, C. (2017) 'Validation of immunoexpression of tenascin-C in oral precancerous and cancerous tissues using ImageJ analysis with novel immunohistochemistry profiler plugin: An immunohistochemical quantitative analysis.', Journal of oral and maxillofacial pathology, 21(2), pp. 211–217.

Mansourian, A. et al. (2008) 'A randomized double blind controlled trial to compare metronidazole with doxycycline for the treatment of oral lichen planus', Journal of Medical Sciences, 8(2), pp. 201–204.

Mansourian, A. et al. (2011) 'Comparison of aloe vera mouthwash with triamcinolone acetonide 0.1% on oral lichen planus: A randomized double-blinded clinical trial', American Journal of the Medical Sciences, 342(6), pp. 447–451.

Mansourian, A. et al. (2017) 'Salivary oxidative stress in oral lichen planus treated with triamcinolone mouthrinse.', Dental research journal, 14(2), pp. 104–110.

Mansourian, A., Shanbehzadeh, N., Kia, S. J., & Moosavi, M.-S. (2017). Increased salivary aldehyde dehydrogenase 1 in non-reticular oral lichen planus. Anais Brasileiros de Dermatologia, 92(2), 168–171.

Mansur, A. T., Kilic, Z., & Atalay, F. (2004). Psychological evaluation of patients with cutaneous lichen planus. Dermatology and Psychosomatics, 5(3), 132–136.

Marable, D. R., Bowers, L. M., Stout, T. L., Stewart, C. M., Berg, K. M., Sankar, V., DeRossi, S. S., Thoppay, J. R., & Brennan, M. T. (2016). Oral candidiasis following steroid therapy for oral lichen planus. Oral Diseases, 22(2), 140–147.

Maraki, D. et al. (2006) 'Cytologic and DNA-cytometric examination of oral lesions in lichen planus.', Journal of oral pathology & medicine : official publication of the International Association of Oral Pathologists and the American Academy of Oral Pathology, 35(4), pp. 227–232.

Mardani, M. et al. (2012) 'Serum level of vascular endothelial growth factor in patients with different clinical subtypes of oral lichen planus', Iranian Journal of Medical Sciences, 37(4), pp. 233–237.

Mardani, M., Tadbir, A. A. and Ahmadi, R. (2011) 'The prevalence of malignant transformation in oral lichen planus in two main centers in Shiraz (2006-2009)', Journal of Mazandaran University of Medical Sciences, 21(84), pp. 145–148.

Marell, L. et al. (2014) ‘Regression of oral lichenoid lesions after replacement of dental restorations’, Journal of oral rehabilitation, 41(5), pp. 381–391.

Maria Zarate, A. et al. (2017) ‘Study of the TP53 codon 72 polymorphism in oral cancer and oral potentially malignant disorders in Argentine patients’, Tumor Biology, 39(5), pp. 1010428317699113–1010428317699113.

Mariela Sano, S. et al. (2008) ‘Sensitivity of direct immunofluorescence in oral diseases. Study of 125 cases’, Medicina Oral, Patologia Oral y Cirugia Bucal, 13(5), pp. 287–291.

Marinho Resende, J. P. et al. (2013) ‘Oral lichen planus treated with tacrolimus 0.1%’, International Journal of Clinical and Experimental Medicine, 6(10), pp. 917–921.

Markopoulos, A. K., Antoniades, D., Papanayotou, P., & Trigonidis, G. (1996). Desquamative gingivitis: a clinical, histopathologic, and immunologic study. Quintessence International (Berlin, Germany : 1985), 27(11), 763–767.

Marques Soares, M. S. et al. (2011) ‘Oral Conditions in Patients with Oral Lichen Planus’, Pesquisa brasileira em odontopediatria e clinica integrada, 11(4), pp. 507–510.

Marshall, A., Celentano, A., Cirillo, N., Mirams, M., McCullough, M., & Porter, S. (2017). Immune receptors CD40 and CD86 in oral keratinocytes and implications for oral lichen planus. Journal of Oral Science, 59(3), 373–382.

Martín-Ezquerra, G. et al. (2010) ‘Multiple genetic copy number alterations in oral squamous cell carcinoma: Study of MYC, TP53, CCDN1, EGFR and ERBB2 status in primary and metastatic tumours’, British Journal of Dermatology, 163(5), pp. 1028–1035.

Martin-Ezquerra, G. et al. (2011) ‘CDC28 protein kinase regulatory subunit 1B (CKS1B) expression and genetic status analysis in oral squamous cell carcinoma’, Histology and Histopathology, 26(1), pp. 71–77.

Martinez, A. et al. (2001) ‘Expression of p53, Ki-67, Bcl-2, Bax in Oral Lichen Planus’, Journal of dental research, 80(4), p. 1185.

Mastrangelo, F., Dolci, M., Stuppia, L., Paolantonio, M., Salini, L., Zizzari, V., Tranasi, M., Patrono, A., & Tete, S. (2007). SEM analysis of oral lichen planus before and after treatment with 13 cis-retinoic acid. International Journal of Immunopathology and Pharmacology, 20(1), 75–79.

Matravers, J. et al. (1978) ‘Scanning electron microscopy of oral epithelial cells. Part II. Potentially malignant lesions (a computer assisted study)’, British Journal of Oral Surgery, 15(3), pp. 203–214.

Matsumoto, K. et al. (2019) ‘Clinical Evaluation of CO2 Laser Vaporization Therapy for Oral Lichen Planus: A Single-Arm Intervention Study.’, Photobiomodulation, photomedicine, and laser surgery, 37(3), pp. 175–181.

Matthews, J. B. et al. (1984) ‘Oral lichen planus: An immunoperoxidase study using monoclonal antibodies to lymphocyte subsets’, British Journal of Dermatology, 111(5), pp. 587–595.

Matthews, J. B. et al. (1985) ‘Macrophages in oral lichen planus.’, Journal of oral pathology, 14(7), pp. 553–558.

Mattila, R. et al. (2012) ‘Human papillomavirus in oral atrophic lichen planus lesions’, Oral Oncology, 48(10), pp. 980–984.

Mattila, R., Ahlfors, E. and Syrjanen, S. (2011) ‘CD27 and CD38 lymphocytes are detected in oral lichen planus lesions.’, Oral surgery, oral medicine, oral pathology, oral radiology, and endodontics, 111(2), pp. 211–217.

Mattsson, T. et al. (1992) ‘A comparative immunological analysis of the oral mucosa in chronic graft-versus-host disease and oral lichen planus.’, Archives of oral biology, 37(7), pp. 539–547.

Mattsson, U. et al. (1994) ‘Computer analysis in oral lichenoid reactions.’, Acta odontologica Scandinavica, 52(2), pp. 86–92.

Mattsson, U. et al. (1995) ‘Use of computer-assisted image analysis for noninvasive evaluation of oral lichenoid reactions and oral leukoplakia.’, Oral surgery, oral medicine, oral pathology, oral radiology, and endodontics, 79(2), pp. 199–206.

McCaughey, C. et al. (2011) ‘Pimecrolimus 1% cream for oral erosive lichen planus: A 6-week randomized, double-blind, vehicle-controlled study with a 6-week open-label extension to assess efficacy and safety’, Journal of the European Academy of Dermatology and Venereology, 25(9), pp. 1061–1067.

McClatchey, K. D. et al. (1975) ‘Studies on oral lichen planus. III. Clinical and histologic correlations in 213 patients’, Oral Surgery Oral Medicine and Oral Pathology, 39(1), pp. 122–129.

McGrath, C. et al. (2003) ‘Patient-centred outcome measures for oral mucosal disease are sensitive to treatment’, International Journal of Oral and Maxillofacial Surgery, 32(3), pp. 334–336.

McParland, H., & Warnakulasuriya, S. (2021). Lichenoid morphology could be an early feature of oral proliferative verrucous leukoplakia. Journal of oral pathology & medicine: official publication of the International Association of Oral Pathologists and the American Academy of Oral Pathology, 50(2), 229–235.

Mehdipour, M. et al. (2014) ‘Evaluation of serum nitric oxide level in patients with oral lichen planus.’, Journal of dentistry (Shiraz, Iran), 15(2), pp. 48–51.

Mehdipour, M. et al. (2015) ‘Evaluation of the relationship between serum lipid profile and oral lichen planus.’, Journal of dental research, dental clinics, dental prospects, 9(4), pp. 261–266.

Mehdipour, M. et al. (2016) ‘The Relationship between Anger Expression and Its Indices and Oral Lichen Planus.’, Chonnam medical journal, 52(2), pp. 112–116.

Mehdipour, M., Taghavi Zenouz, A., Bahramian, A., et al. (2010) ‘Comparison of the Effect of Mouthwashes with and without Zinc and Fluocinolone on the Healing Process

of Erosive Oral Lichen Planus.', Journal of dental research, dental clinics, dental prospects, 4(1), pp. 25–28.

Mehdipour, M., Taghavi Zenouz, A., Hekmatfar, S., et al. (2010) 'Prevalence of Candida species in erosive oral lichen planus.', Journal of dental research, dental clinics, dental prospects, 4(1), pp. 14–16.

Mendes, G.-G., Servato, J.-P.-S., Borges, F.-C., Rosa, R.-R., Siqueira, C.-S., de Faria, P.-R., Loyola, A.-M., & Cardoso, S.-V. (2018). Differential metallothionein expression in oral lichen planus and amalgam-associated oral lichenoid lesions. Medicina Oral Patologia Oral y Cirugia Bucal, 23(3), e262--e268.

Mergoni, G. et al. (2019) 'Effects of oral healthcare motivation in patients with gingival oral lichen planus: A randomized controlled trial', Oral Diseases, 25(5), pp. 1335–1343.

Metwaly, H., Ebrahem, M. A.-M. and Saku, T. (2014) 'Vascular endothelial growth factor (VEGF) and inducible nitric oxide synthase (iNOS) in oral lichen planus: An immunohistochemical study for the correlation between vascular and inflammatory reactions', Journal of oral and maxillofacial surgery medicine and pathology, 26(3), pp. 390–396.

MFMS, C. et al. (2019) 'Cytokines levels and salivary microbiome play a potential role in oral lichen planus diagnosis.', Scientific reports, 9(1), p. 18137.

Mignogna, M. D. et al. (2005) 'Gingival involvement of oral lichen planus in a series of 700 patients', Journal of Clinical Periodontology, 32(10), pp. 1029–1033.

Mignogna, M. D. et al. (2007) 'Field cancerization in oral lichen planus', European Journal of Surgical Oncology, 33(3), pp. 383–389.

Miri-Moghaddam, M. and Kadeh, H. (2016) 'Immunohistochemical Expression of Stromelysin-2 (St-2) In Patients with Oral Lichen Planus and Its Clinical Significance.', Journal of dentistry (Shiraz, Iran), 17(3), pp. 250–255.

Miricescu, D. et al. (2011) 'The antioxidant potential of saliva: Clinical significance in oral diseases', Therapeutics, Pharmacology and Clinical Toxicology, 15(2), pp. 139–143.

Mirza, S. et al. (2018) 'Efficacy of photodynamic therapy or low level laser therapy against steroid therapy in the treatment of erosive-atrophic oral lichen planus.', Photodiagnosis and photodynamic therapy, 21, pp. 404–408.

Mittal, N., Shankari, G. M. and Palaskar, S. (2012) 'Role of angiogenesis in the pathogenesis of oral lichen planus.', Journal of oral and maxillofacial pathology, 16(1), pp. 45–48.

Mittermüller, P. et al. (2018) 'Five hundred patients reporting on adverse effects from dental materials: Frequencies, complaints, symptoms, allergies', Dental materials: official publication of the Academy of Dental Materials, 34(12), pp. 1756–1768.

Mizuki, H. et al. (2017) 'Immunohistochemical detection of Mycoplasma salivarium in oral lichen planus tissue.', Journal of oral pathology & medicine: official publication of

the International Association of Oral Pathologists and the American Academy of Oral Pathology, 46(8), pp. 649–656.

ML, Z. et al. (2019) ‘Severity of oral lichen planus and oral lichenoid lesions is associated with anxiety.’, Clinical oral investigations, 23(12), pp. 4441–4448.

Mohan, R. P. S. et al. (2017) ‘Incidence of Oral Lichen Planus in Perimenopausal Women: A Cross-sectional Study in Western Uttar Pradesh Population.’, Journal of mid-life health, 8(2), pp. 70–74.

Mollashahi, L. F. et al. (2016) ‘Evaluation of salivary total sialic acid in patients with oral erosive lichen planus’, Journal of Mashhad Dental School, 40(2), pp. 143–148.

Moncarz, V., Ulmansky, M. and Lustmann, J. (1993) ‘Lichen-planus - exploring its malignant potential’, Journal of the american dental association, 124(3), pp. 102--.

Montebugnoli, L. et al. (2006) ‘High proliferative activity and chromosomal instability in oral lichen planus’, International Journal of Oral and Maxillofacial Surgery, 35(12), pp. 1140–1144.

Montebugnoli, L. et al. (2011) ‘Immunohistochemical expression of p16(INK4A) protein in oral lichen planus.’, Oral surgery, oral medicine, oral pathology, oral radiology, and endodontics, 112(2), pp. 222–227.

Montebugnoli, L. et al. (2012) ‘Clinical and histologic healing of lichenoid oral lesions following amalgam removal: a prospective study.’, Oral surgery, oral medicine, oral pathology and oral radiology, 113(6), pp. 766–772.

Morandi, L. et al. (2017) ‘CpG location and methylation level are crucial factors for the early detection of oral squamous cell carcinoma in brushing samples using bisulfite sequencing of a 13-gene panel’, Clinical Epigenetics, 9(1), p. 85.

Morrison, L. et al. (2002) ‘An open trial of topical tacrolimus for erosive oral lichen planus’, Journal of the American Academy of Dermatology, 47(4), pp. 617–620.

Morruzzi, C. et al. (2009) ‘Four cases of photopheresis treatment for cutaneous lupus erythernatosus refractory to standard therapy’, Annales de dermatologie et de venereologie, 136(12), pp. 861–867.

Moshaverinia, M. et al. (2014) ‘The relationship between oral lichen planus and blood group antigens’, World Journal of Medical Sciences, 10(2), pp. 103–105.

Mostafa, B., & Zakaria, M. (2018). Evaluation of combined topical ozone and steroid therapy in management of oral lichen planus. Open Access Macedonian Journal of Medical Sciences, 6(5), 879–884.

Mostafa, D. et al. (2017) ‘Evaluation of photodynamic therapy in treatment of oral erosive lichen planus in comparison with topically applied corticosteroids’, Photodiagnosis and Photodynamic Therapy, 19, pp. 56–66.

Moule, I., Parsons, P. A. and Irvine, G. H. (1995) ‘Avoiding artefacts in oral biopsies: the punch biopsy versus the incisional biopsy.’, The British journal of oral & maxillofacial surgery, 33(4), pp. 244–247.

Mousavi, F. et al. (2009) ‘Ignatia in the treatment of oral lichen planus’, Homeopathy, 98(1), pp. 40–44.

Mozaffari, H. R., Mirbahari, S. and Sadeghi, M. (2018) ‘Histopathological Findings in Oral Lichen Planus: A Three-Year Report from Western Iran’, Journal of research in medical and dental science, 6(1), pp. 274–278.

MSK, A. et al. (2020) ‘Laminin-332 expression in oral lichen planus: Preliminary results of a cross-sectional study.’, Oral diseases.

Mucke, T. et al. (2015) ‘Clinical trial analyzing the impact of continuous defocused CO2 laser vaporisation on the malignant transformation of erosive oral lichen planus’, Journal of Cranio-Maxillofacial Surgery, 43(8), pp. 1567–1570.

Mukae, S. et al. (2009) ‘Detection of fascin and CCR-7 positive mature dendritic cells in oral lichen planus.’, Journal of oral pathology & medicine: official publication of the International Association of Oral Pathologists and the American Academy of Oral Pathology, 38(4), pp. 334–342.

Mulayim, M. K. and Uzun, P. O. S. (2016) ‘The Effectiveness of Topical Pimecrolimus in the Treatment of Oral Lichen Planus’, Turk dermatoloji dergisi-turkish journal of dermatology, 10(4), pp. 152–156.

Muñoz, A. A. et al. (2007) ‘Behavior of oral squamous cell carcinoma in subjects with prior lichen planus’, Otolaryngology - Head and Neck Surgery, 136(3), pp. 401–404.

Muraki, Y. et al. (1997) ‘Immunohistochemical detection of Fas antigen in oral epithelia.’, Journal of oral pathology & medicine: official publication of the International Association of Oral Pathologists and the American Academy of Oral Pathology, 26(2), pp. 57–62.

Muris, J. et al. (2015) ‘Sensitization to palladium and nickel in Europe and the relationship with oral disease and dental alloys.’, Contact dermatitis, 72(5), pp. 286–296.

Murti, P. R. et al. (1986) ‘Malignant potential of oral lichen planus: observations in 722 patients from India.’, Journal of oral pathology, 15(2), pp. 71–77.

MY, A. et al. (2020) ‘Expression of nucleotide-binding oligomerization domain 1 and 2 in oral lichen planus.’, Journal of dental sciences, 15(1), pp. 1–8.

Myoung, H. et al. (2007) ‘Chemiluminescence in diagnosis of oral lichen planus’, Journal of the korean association of oral and maxillofacial surgeons, 33(4), pp. 281–287.

N, G., A, E. R., et al. (2020) ‘Association of MAPK and its regulatory miRNAs (603, 4301, 8485, and 4731) with the malignant transformation of oral lichen planus.’, Molecular biology reports, 47(2), pp. 1223–1232.

N, G., F, P., et al. (2020) ‘Relationship between salivary vitamin D deficiency and oral lichen planus.’, Photodermatology, photoimmunology & photomedicine, 36(5), pp. 384–386.

N, G., M, A. R., et al. (2020) ‘Serum and salivary levels of lactate dehydrogenase in oral squamous cell carcinoma, oral lichen planus and oral lichenoid reaction.’, BMC oral health, 20(1), p. 314.

N, S. et al. (2020) ‘Treatment of oral lichen planus with mucoadhesive mycophenolate mofetil patch: A randomized clinical trial.’, Clinical and experimental dental research, 6(5), pp. 506–511.

N., C.-W. et al. (2007) ‘A randomized, placebo-controlled, double-blind clinical trial of curcuminoids in oral lichen planus’, Phytomedicine, 14(7), pp. 437–446.

N., C.-W. et al. (2008) ‘Validation of instruments to measure the symptoms and signs of oral lichen planus’, Oral Surgery, Oral Medicine, Oral Pathology, Oral Radiology, and Endodontics, 105(1), pp. 51–58.

N., C.-W. et al. (2012) ‘High-dose curcuminoids are efficacious in the reduction in symptoms and signs of oral lichen planus’, Journal of the American Academy of Dermatology, 66(5), pp. 752–760.

N., H. H. et al. (1986) ‘A semi-quantitative assessment of the histopathology of oral lichen planus.’, Journal of oral pathology, 15(5), pp. 268–272.

N., H. L. et al. (1999) ‘Oral lichen planus plaques and homogeneous leukoplasia: Comparative results of treatment with CO2 laser’, Acta Otorrinolaringologica Espanola, 50(7), pp. 543–547.

N., M. et al. (2015) ‘A case-control study to detect the extent of DNA damage in oral lichen planus and oral lichenoid reactions using comet assay’, Journal of Pharmacy and Bioallied Sciences, 7(6), pp. S451--S456.

N., T. et al. (2000) ‘Expression of apoptotic signaling proteins in leukoplakia and oral lichen planus: Quantitative and topographical studies’, Journal of Oral Pathology and Medicine, 29(8), pp. 385–393.

N.A., B. et al. (1993) ‘Oral cancer development in patients with oral lichen planus’, Journal of Oral Pathology and Medicine, 22(9), pp. 421–424.

N.G., E. L. et al. (1974) ‘Civatte bodies and the actively dividing epithelial cells in oral lichen planus’, British Journal of Dermatology, 90(1), pp. 13–23.

N.G., E.-L. (1982) ‘The nature of Langerhans cell granules: An ultrastructural study’, Histopathology, 6(3), pp. 317–325.

N.M., H. et al. (1987) ‘The expression of HLA-DR on keratinocytes in oral lichen planus.’, Journal of oral pathology, 16(1), pp. 31–35.

N.P., D. et al. (1997) ‘Apoptosis-associated markers in oral lichen planus’, Journal of Oral Pathology and Medicine, 26(4), pp. 170–175.

NA, A. et al. (2020) 'DNA integrity in diagnosis of premalignant lesions.', Medicina oral, patologia oral y cirugia bucal.

Nadendla, L. K. et al. (2014) 'Association of salivary cortisol and anxiety levels in lichen planus patients.', Journal of clinical and diagnostic research : JCDR, 8(12), pp. ZC01--3.

Nafarzadeh, S. et al. (2013) 'Comparative study of expression of smad3 in oral lichen planus and normal oral mucosa.', International journal of molecular and cellular medicine, 2(4), pp. 194–198.

Nafarzadeh, S., Jafari, S. and Bijani, A. (2013) 'Assessment of bax and bcl-2 immunoexpression in patients with oral lichen planus and oral squamous cell carcinoma.', International journal of molecular and cellular medicine, 2(3), pp. 136–142.

Nagao, Y. et al. (2000) 'Detection of hepatitis C virus RNA in oral lichen planus and oral cancer tissues', Journal of Oral Pathology and Medicine, 29(6), pp. 259–266.

Nagao, Y., Tomonari, R., et al. (2002) 'The possible intraspousal transmission of HCV in terms of lichen planus.', International journal of molecular medicine, 10(5), pp. 569–573.

Nagao, Y., Tsubone, K., et al. (2002) 'High prevalence of anticardiolipin antibodies in patients with HCV-associated oral lichen planus.', International journal of molecular medicine, 9(3), pp. 293–297.

Naik, S. R. et al. (2020) 'Reduced levels of serum vitamin B12 in symptomatic cases of oral lichen planus: A cross-sectional study.', Journal of oral biology and craniofacial research, 10(4), pp. 578–582.

Negi, D. et al. (2019) 'Assessment of Interleukin-18 gene polymorphism and serum levels in oral lichen planus in an Indian population', Journal of Oral Pathology and Medicine, 48(3), pp. 244–250.

Németh, C. G., Röcken, C., Siebert, R., Wiltfang, J., Ammerpohl, O., & Gassling, V. (2019). Recurrent chromosomal and epigenetic alterations in oral squamous cell carcinoma and its putative premalignant condition oral lichen planus. PLoS ONE, 14(4), e0215055–e0215055.

Neopane, P. et al. (2019) 'Immunohistochemical Localization of RNase 7 in Normal and Inflamed Oral Epithelia and Salivary Glands.', Acta histochemica et cytochemica, 52(2), pp. 35–43.

Neppelberg, E. et al. (2001) 'Apoptosis in oral lichen planus.', European journal of oral sciences, 109(5), pp. 361–364

Neppelberg, E. et al. (2007) 'Altered CD40 and E-cadherin expression--putative role in oral lichen planus.', Journal of oral pathology & medicine: official publication of the International Association of Oral Pathologists and the American Academy of Oral Pathology, 36(3), pp. 153–160.

Neppelberg, E., & Johannessen, A. C. (2007). DNA content, Cyclooxygenase-2 expression and loss of E-cadherin expression do not predict risk of malignant

transformation in oral lichen planus. European Archives of Oto-Rhino-Laryngology, 264(10), 1223–1230.

Neumann-Jensen, B., Holmstrup, P., & Pindborg, J. J. (1977). Smoking habits of 611 patients with oral lichen planus. Oral Surgery Oral Medicine and Oral Pathology, 43(3), 410–415.

Ni Riordain, R., Christou, J., et al. (2016) 'Cost of illness of oral lichen planus in a U.K. population--a pilot study.', Journal of oral pathology & medicine: official publication of the International Association of Oral Pathologists and the American Academy of Oral Pathology, 45(5), pp. 381–384.

Ni Riordain, R., Hodgson, T., et al. (2016) 'Validity and reliability of the Chronic Oral Mucosal Diseases Questionnaire in a UK population.', Journal of oral pathology & medicine: official publication of the International Association of Oral Pathologists and the American Academy of Oral Pathology, 45(8), pp. 613–616.

Ni Riordain, R., Meaney, S. and McCreary, C. (2011) 'A patient-centered approach to developing a quality-of-life questionnaire for chronic oral mucosal diseases.', Oral surgery, oral medicine, oral pathology, oral radiology, and endodontics, 111(5), pp. 578--86, 586.e1--2.

Nisa, S. U. and Saggu, T. K. (2016) 'To estimate the efficacy of 0.1% tacrolimus with Colgate Oraguard-B paste for the treatment of patients with symptomatic oral lichen planus.', Indian journal of dentistry, 7(1), pp. 23–27.

Nishimura, M. et al. (2003) 'Localization of human beta-defensin 3 mRNA in normal oral epithelium, leukoplakia, and lichen planus: an in situ hybridization study.', Medical electron microscopy : official journal of the Clinical Electron Microscopy Society of Japan, 36(2), pp. 94–97.

NL, C. et al. (2020) 'Evaluation of PD-L1, PD-L2, PD-1 and cytotoxic immune response in oral lichen planus.', Oral diseases.

Nolan, A. et al. (2009) 'The efficacy of topical hyaluronic acid in the management of oral lichen planus', Journal of Oral Pathology and Medicine, 38(3), pp. 299–303.

Nosratzehi, F., Nosratzehi, T., Alijani, E., & Rad, S. S. (2020). Salivary β2-microglobulin levels in patients with erosive oral lichen planus and squamous cell carcinoma. BMC Research Notes, 13(1), 294.

Nosratzehi, T. et al. (2017) 'Investigating the level of salivary endothelin-1 in premalignant and malignant lesions', Special care in dentistry: official publication of the American Association of Hospital Dentists, the Academy of Dentistry for the Handicapped, and the American Society for Geriatric Dentistry, 37(3), pp. 134–139.

Nosratzehi, T., Alijani, E. and Moodi, M. (2017) 'Salivary MMP-1, MMP-2, MMP-3 and MMP-13 Levels in Patients with Oral Lichen Planus and Squamous Cell Carcinoma', Asian Pacific journal of cancer prevention, 18(7), pp. 1947–1951.

NR, A., N, Y. and E, A. (2020) ‘The effect of smoking on clinical presentation and expression of TLR-2 and CD34 in Oral lichen Planus patients: clinical and immunohistochemical study.’, BMC oral health, 20(1), p. 129.

Nylander, E. et al. (2012) ‘Changes in miRNA expression in sera and correlation to duration of disease in patients with multifocal mucosal lichen planus.’, Journal of oral pathology & medicine: official publication of the International Association of Oral Pathologists and the American Academy of Oral Pathology, 41(1), pp. 86–89.

O, S., A, K., et al. (2020) ‘Expression of keratins 8, 18, and 19 in epithelia of atrophic oral lichen planus.’, European journal of oral sciences, 128(1), pp. 7–17.

O, S., MG, B., et al. (2020) ‘Composition of hemidesmosomes in basal keratinocytes of normal buccal mucosa and oral lichen planus.’, European journal of oral sciences, 128(5), pp. 369–378.

O., M., A., S. and G., L. (1981) ‘Clinical study of 175 patients with oral lichen planus’, Stomatologia, 38(5), pp. 283–288.

O., O. et al. (1985) ‘A histologic study of epithelial dysplasia in oral lichen planus’, Archives of Dermatology, 121(9), pp. 1132–1136.

O.F., R. et al. (2016) ‘Immunomorphology of oral lichen planus’, Stomatologiia, 95(2), pp. 4–7.

O’Flatharta, C. et al. (2001) ‘Telomerase activity detected in Oral Lichen Planus by RNA in-situ hybridisation’, Journal of pathology, 193, pp. 21A-21A.

O’Flatharta, C., Flint, S. R., Toner, M., Butler, D., & Mabruk, M. J. E. M. F. (2003). Investigation into a Possible Association between Oral Lichen Planus, the Human Herpesviruses, and the Human Papillomaviruses. Molecular Diagnosis, 7(2), 73–83.

O’Flatharta, C., Leader, M., Kay, E., Flint, S. R., Toner, M., Robertson, W., & Mabruk, M. J. E. M. F. (2002). Telomerase activity detected in oral lichen planus by RNA in situ hybridisation: Not a marker for malignant transformation. Journal of Clinical Pathology, 55(8), 602–607.

Ögmundsdóttir H. M., Björnsson J. & Holbrook W. P. (2009), "Role of TP53 in the progression of pre-malignant and malignant oral mucosal lesions. A follow-up study of 144 patients", Journal of Oral Pathology and Medicine. Vol. 38(7), pp. 565-571.

Ogmundsdottir, H. M. et al. (2002) ‘Oral lichen planus has a high rate of TP53 mutations. A study of oral mucosa in icelanD.’, European journal of oral sciences, 110(3), pp. 192–198.

Ohno, S. et al. (2011) ‘Enhanced expression of Toll-like receptor 2 in lesional tissues and peripheral blood monocytes of patients with oral lichen planus’, Journal of Dermatology, 38(4), pp. 324–333.

Oliveira Alves, M. et al. (2013) ‘Evaluation of the expression of p53, MDM2, and SUMO-1 in oral lichen planus’, Oral Diseases, 19(8), pp. 775–780.

Oluwadara, O. et al. (2009) 'LCK, survivin and PI-3K in the molecular biomarker profiling of oral lichen planus and oral squamous cell carcinoma.', Bioinformation, 4(6), pp. 249–257.

Onofre, M. A. et al. (1997) 'Potentially malignant epithelial oral lesions: discrepancies between clinical and histological diagnosis.', Oral diseases, 3(3), pp. 148–152.

Orakzai, G. S., Waqar-Un-Nisa, & Orakzai, S. H. (2015). Oral white lesions--histomorhological assessment and associated risk factors. Journal of Ayub Medical College, Abbottabad, 27(4), 865–868.

Osipoff, A., Carpenter, M. D., Noll, J. L., Valdez, J. A., Gormsen, M., & Brennan, M. T. (2020). Predictors of symptomatic oral lichen planus. Oral surgery, oral medicine, oral pathology and oral radiology, *129*(5), 468–477.

Ostman, P. O. et al. (1994) 'Oral lichen planus lesions in contact with amalgam fillings: a clinical, histologic, and immunohistochemical study.', Scandinavian journal of dental research, 102(3), pp. 172–179

Ostman, P. O., Anneroth, G., Johansson, I., et al. (1996) 'Life-style survey of patients with oral lichenoid reactions.', Acta odontologica Scandinavica, 54(2), pp. 96–101.

Ostman, P. O., Anneroth, G., Skoglund, A., et al. (1996) 'Amalgam-associated oral lichenoid reactions. Clinical and histologic changes after removal of amalgam fillings.', Oral surgery, oral medicine, oral pathology, oral radiology, and endodontics, 81(4), pp. 459–465.

Ostwald, C. et al. (2003) 'Human papillomavirus 6/11, 16 and 18 in oral carcinomas and benign oral lesions', Medical Microbiology and Immunology, 192(3), pp. 145–148.

Othman, N.-A. et al. (2016) 'The effect of diode laser and topical steroid on serum level of TNF-alpha in oral lichen planus patients.', Journal of clinical and experimental dentistry, 8(5), pp. e566--e570.

Özkur, E., Uğurer, E., & Altunay, İ. K. (2020). Dyslipidemia in Lichen Planus: A Case-control Study. Sisli Etfal Hastanesi Tip Bulteni, 54(1), 62–66.

P.-O., R. et al. (1994) 'Erosive oral lichen planus treated with clobetasol propionate and triamcinolone acetonide in Orabase: A double-blind clinical trial', Journal of Dermatological Treatment, 5(1), pp. 7–10.

P.-O., R. et al. (2004) 'Cancer and oral lichen planus in a Swedish population', Oral Oncology, 40(2), pp. 131–138.

P.-R., J. et al. (2018) 'Correlations between the T helper cell 17/regulatory T cells balance in peripheral blood of patients with oral lichen planus and clinical characteristics', Hua xi kou qiang yi xue za zhi = Huaxi kouqiang yixue zazhi = West China journal of stomatology, 36(4), pp. 384–388.

P., B. et al. (2006) 'Prevalence of vulval lichen planus in a cohort of women with oral lichen planus: An interdisciplinary study', British Journal of Dermatology, 155(5), pp. 994–998.

P., C. et al. (1999) ‘Oral lichen planus: An immunohistochemical study of heat shock proteins (HSPS) and cytokeratins (CKS) and a unifying hypothesis of pathogenesis’, Journal of Oral Pathology and Medicine, 28(5), pp. 210–215.

P., C. et al. (2005) ‘Nitrative and oxidative DNA damage in oral lichen planus in relation to human oral carcinogenesis’, Cancer Science, 96(9), pp. 553–559.

P., C. et al. (2008) ‘Alteration of the expression of CD4 isoforms in oral epithelia and saliva from patients with oral lichen planus’, Journal of Clinical Immunology, 28(1), pp. 26–34.

P., C. et al. (2018) ‘A prospective observational study to compare efficacy of topical triamcinolone acetonide 0.1% oral paste, oral methotrexate, and a combination of topical triamcinolone acetonide 0.1% and oral methotrexate in moderate to severe oral lichen planus’, Dermatologic Therapy, 31(1).

P., H. et al. (1990) ‘Effect of dental plaque control on gingival lichen planus’, Oral Surgery Oral Medicine and Oral Pathology, 69(5), pp. 585–590.

P., K. et al. (1999) ‘Oral lesions and symptoms related to metals used in dental restorations: A clinical, allergological, and histologic study’, Journal of the American Academy of Dermatology, 41(3), pp. 422–430.

P., K. et al. (2016) ‘Stress as an etiologic co-factor in recurrent aphthous ulcers and oral lichen planus’, Journal of oral science, 58(2), pp. 237–240.

Paderni, C. et al. (2011) ‘Direct visualization of oral-cavity tissue fluorescence as novel aid for early oral cancer diagnosis and potentially malignant disorders monitoring’, International journal of immunopathology and pharmacology, 24(2), pp. 121–128.

Pagano, S. et al. (2009) ‘Results evaluation of a recent pharmacological protocol for the treatment of lichen ruber planus [Valutazione di un protocollo farmacologico per il trattamento del lichen ruber planus]’, Dental Cadmos, 77(10), pp. 19–30.

Pakfetrat, A. et al. (2014) ‘Removal of refractory erosive-atrophic lichen planus by the CO2 laser.’, Oral health and dental management, 13(3), pp. 595–599.

Pakfetrat, A. et al. (2015) ‘The effect of pimecrolimus cream 1% compared with triamcinolone acetonide paste in treatment of atrophic-erosive oral lichen planus’, Iranian Journal of Otorhinolaryngology, 27(79), pp. 119–126.

Pakfetrat, A., Javadzadeh-Bolouri, A., Basir-Shabestari, S., & Falaki, F. (2009). Oral lichen planus: A retrospective study of 420 Iranian patients. Medicina Oral, Patologia Oral y Cirugia Bucal, 14(7), E315--E318.

Pan, Y.-X., Cai, Y. and Yu, H.-B. (2011) ‘[Expression of interleukin-12p40 and interferon-gamma in local lesions of human oral lichen planus].’, Hua xi kou qiang yi xue za zhi = Huaxi kouqiang yixue zazhi = West China journal of stomatology, 29(2), pp. 179–182.

Pandey, A., Setty, S., Rao, R., & Radhakrishnan, R. (2011). Assessment of Langerhans cells in oral lichen planus by ATPase histochemistry: a clinicopathologic correlation. Quintessence International (Berlin, Germany : 1985), 42(3), 225–234.

Pang, B. K. and Freeman, S. (1995) 'Oral lichenoid lesions caused by allergy to mercury in amalgam fillings.', Contact dermatitis, 33(6), pp. 423–427.

Parajuli, H. et al. (2017) 'Integrin alpha11 is overexpressed by tumour stroma of head and neck squamous cell carcinoma and correlates positively with alpha smooth muscle actin expression.', Journal of oral pathology & medicine: official publication of the International Association of Oral Pathologists and the American Academy of Oral Pathology, 46(4), pp. 267–275.

Park, S.-Y. et al. (2018) 'Factors affecting treatment outcomes in patients with oral lichen planus lesions: a retrospective study of 113 cases.', Journal of periodontal & implant science, 48(4), pp. 213–223.

Parlatescu, I., Tovaru, M., Nicolae, C. L., Sfeatcu, R., & Didilescu, A. C. (2020). Oral health-related quality of life in different clinical forms of oral lichen planus. Clinical Oral Investigations, 24(1), 301–308.

Passeron, T. et al. (2007) 'Treatment of oral erosive lichen planus with 1% pimecrolimus cream: a double-blind, randomized, prospective trial with measurement of pimecrolimus levels in the blood.', Archives of dermatology, 143(4), pp. 472–476.

Patel, K. J. et al. (2011) 'Concordance between clinical and histopathologic diagnoses of oral mucosal lesions.', Journal of oral and maxillofacial surgery: official journal of the American Association of Oral and Maxillofacial Surgeons, 69(1), pp. 125–133.

Patil, P. B., Bathi, R. and Chaudhari, S. (2013) 'Prevalence of oral mucosal lesions in dental patients with tobacco smoking, chewing, and mixed habits: A cross-sectional study in South India.', Journal of family & community medicine, 20(2), pp. 130–135.

Patil, S. et al. (2015) 'Lichenoid dysplasia revisited - evidence from a review of Indian archives', Journal of Oral Pathology and Medicine, 44(7), pp. 507–514.

Paul, M. and Shetty, D. C. (2013) 'Analysis of the changes in the basal cell region of oral lichen planus: An ultrastructural study.', Journal of oral and maxillofacial pathology, 17(1), pp. 10–16.

Paulusová, V. et al. (2010) 'Cytochrome P450 2D6 polymorphism and drug utilization in patients with oral lichen planus.', Acta odontologica Scandinavica, 68(4), pp. 193–198.

Paulusová, V. et al. (2012) 'Expression of matrix metalloproteinase 9 in patients with oral lichen planus.', Acta medica (Hradec Králové) / Universitas Carolina, Facultas Medica Hradec Králové, 55(1), pp. 23–26.

Pavic, I., Baličević, D., Vrdoljak, B., Filipović, M., Zekic, J., & Boras, V. V. (2017). The significance Of FHIT and BCL-2 in patients with oral lichen planus in comparison to the healthy oral mucosa and oral squamous cell cancer. Libri Oncologici, 45(1), 9–14.

Pekiner, F. N., Borahan, M. O. and Ozbayrak, S. (2014) 'Evaluation of levels of cortizol, anxiety and depression in patients with oral lichen planus (OLP)', Clinical and experimental health sciences, 4(1), pp. 24–28.

Pekiner, F. N., Demirel, Gulderen Y, et al. (2012) 'Evaluation of cytotoxic T-cell activation, chemokine receptors, and adhesion molecules in blood and serum in patients with oral lichen planus', Journal of Oral Pathology and Medicine, 41(6), pp. 484–489.

Pekiner, F. N., Demirel, Gulderen Yanikkaya, et al. (2012) 'Cytokine profiles in serum of patients with oral lichen planus', Cytokine, 60(3), pp. 701–706.

Peng H, Wang Y, Shen L and Zhou G (2020), "Reflectance confocal microscopy characteristics of oral lichen planus: An analysis of 47 cases in a Chinese cohort.", Experimental and therapeutic medicine. Vol. 20(5), pp. 6.

Perdigão, P. F. et al. (2007) 'Serotonin transporter gene polymorphism (5-HTTLPR) in patients with oral lichen planus', Archives of Oral Biology, 52(9), pp. 889–893.

Pereira, J. S. et al. (2012) 'FoxP3(+) T regulatory cells in oral lichen planus and its correlation with the distinct clinical appearance of the lesions.', International journal of experimental pathology, 93(4), pp. 287–294.

Pereira, T. et al. (2019) 'Quantitative and Qualitative Analysis of Mast Cells in Oral Lichen Planus and Its Effect on Basement Membrane Using Special Stains.', Indian dermatology online journal, 10(4), pp. 431–436.

Perez, M. A. et al. (2018) 'Different expression patterns of carbonic anhydrase IX in oral lichen planus and leukoplakia.', Acta odontologica latinoamericana : AOL, 31(2), pp. 77–81.

Persic, S. et al. (2008) 'Oral lesions in patients with lichen planus', Acta Clinica Croatica, 47(2), pp. 91–96.

Petruzzi, M. et al. (2004) 'Immune response in patients with oral lichen planus and HCV infection', International Journal of Immunopathology and Pharmacology, 17(1), pp. 93–98.

Pezelj-Ribaric, S. et al. (2004) 'Salivary levels of tumor necrosis factor-alpha in oral lichen planus.', Mediators of inflammation, 13(2), pp. 131–133.

Piattelli, A., Carinci, F., Iezzi, G., Perrotti, V., Goteri, G., Fioroni, M., & Rubini, C. (2007). Oral lichen planus treated with 13-cis-retinoic acid (isotretinoin): Effects on the apoptotic process. Clinical Oral Investigations, 11(3), 283–288.

Piccinni, M.-P. et al. (2014) 'Potential pathogenetic role of Th17, Th0, and Th2 cells in erosive and reticular oral lichen planus', Oral Diseases, 20(2), pp. 212–218.

Pigatti, F. M. et al. (2015) 'Immunohistochemical expression of Bcl-2 and Ki-67 in oral lichen planus and leukoplakia with different degrees of dysplasia', International Journal of Dermatology, 54(2), pp. 150–155.

Pihlman, K. et al. (1985) 'Immunologic findings of oral lichen planus.', Scandinavian journal of dental research, 93(4), pp. 336–342.

Pimenta, F. J. G. S., Pinheiro, M. das G. R. and Gomez, R. S. (2004) 'Expression of hMSH2 protein of the human DNA mismatch repair system in oral lichen planus.', International journal of medical sciences, 1(3), pp. 146–151.

Pimentel, V. N. et al. (2010) 'Perforin and granzyme B involvement in oral lesions of lichen planus and chronic GVHD', Journal of Oral Pathology and Medicine, 39(10), pp. 741–746.

Piñas, L. et al. (2018) 'Biomolecules in the treatment of lichen planus refractory to corticosteroid therapy: Clinical and histopathological assessment', Annals of Anatomy, 216, pp. 159–163.

Pirkic, A., Biocina-Lukenda, D., Cekic-Arambasin, A., Bukovic, D., Pavelic, L., et al. (2004) 'Changes in the Tissue expression of the C-erbB-2 oncogen in the oral lichen ruber', Collegium Antropologicum, 28(1), pp. 455–461.

Pirkic, A., Biocina-Lukenda, D., Cekic-Arambasin, A., Bukovic, D., Habek, M., et al. (2004) 'Tissue expression of proliferative antigens (PCNA and Ki-67) in oral lichen ruber related to clinical status.', Collegium antropologicum, 28(1), pp. 447–453.

PO, Q. et al. (2020) 'Selenium: A sole treatment for erosive oral lichen planus (Randomized controlled clinical trial).', Oral diseases, 26(4), pp. 789–804

Pol, C. A., Ghige, S. K., & Gosavi, S. R. (2015). Role of human papilloma virus-16 in the pathogenesis of oral lichen planus--an immunohistochemical study. International Dental Journal, 65(1), 11–14.

Polesello, V. et al. (2017) 'DEFB1 polymorphisms and salivary hBD-1 concentration in Oral Lichen Planus patients and healthy subjects', Archives of oral biology, 73, pp. 161–165.

Poomsawat, S. et al. (2011) 'Overexpression of cdk4 and p16 in oral lichen planus supports the concept of premalignancy', Journal of Oral Pathology and Medicine, 40(4), pp. 294–299.

Popovska, M et al. (2015) 'Salivary humoral changes in oral bullous lichen planus [Promene pljuvacke kod osoba sa buloznim lihen planusom]', Acta Stomatologica Naissi, 31(72), pp. 1493–1503.

Popovska, Mirjana et al. (2015) 'Etiopathogenetic Biochemical Mechanism Involved in Oral Lichen Planus', Revista de chimie, 66(11), pp. 1786–1790.

Potts, A. J. C., Hamburger, J. and Scully, C. (1987) 'The medication of patients with oral lichen-planus and the association of nonsteroidal antiinflammatory drugs with erosive lesions', Oral surgery oral medicine oral pathology oral radiology and endodontology, 64(5), pp. 541–543.

Pouralibaba, F. et al. (2013) 'Serum Level of Interleukin 17 in Patients with Erosive and Non erosive Oral Lichen Planus.', Journal of dental research, dental clinics, dental prospects, 7(2), pp. 91–94.

Pourshahidi, S. et al. (2012) 'Lack of association between Helicobacter pylori infection and oral lichen planus.', Asian Pacific journal of cancer prevention : APJCP, 13(5), pp. 1745–1747.

Pramod, R. et al. (2014) 'Immunohistochemical assessment of proliferating cell nuclear antigen protein expression in plaque, reticular and erosive types of oral lichen planus.', Annals of medical and health sciences research, 4(4), pp. 598–602.

Prasanna, S. W. et al. (2015) 'Photodynamic therapy of oral leukoplakia and oral lichen planus using methylene blue: A pilot study', Journal of innovative optical health sciences, 8(1).

Prodromidis, G., Nikitakis, N. G. and Sklavounou, A. (2013) 'Immunohistochemical Analysis of the Activation Status of the Akt/mTOR/pS6 Signaling Pathway in Oral Lichen Planus.', International journal of dentistry, 2013, p. 743456.

Prucktrakul, C. et al. (2015) 'Oral lichenoid lesions and serum antinuclear antibodies in Thai patients', Journal of Oral Pathology and Medicine, 44(6), pp. 468–474.

Pujar, A. et al. (2015) 'Comparing the efficacy of hematoxylin and eosin, periodic acid schiff and fluorescent periodic acid schiff-acriflavine techniques for demonstration of basement membrane in oral lichen planus: A histochemical study', Indian Journal of Dermatology, 60(5), pp. 450–456.

Q., P. et al. (2018) 'Differentially circulating exosomal microRNAs expression profiling in oral lichen planus', American Journal of Translational Research, 10(9), pp. 2848–2858.

Q., P. et al. (2019) 'Circulating exosomes regulate T-cell–mediated inflammatory response in oral lichen planus', Journal of Oral Pathology and Medicine, 48(2), pp. 143–150.

Qiao, B., Huang, J., Mei, Z., Lam, A. K., Zhao, J., & Ying, L. (2020). Analysis of immune microenvironment by multiplex immunohistochemistry staining in different oral diseases and oral squamous cell carcinoma. Frontiers in Oncology, 10, 555757.

R, C. et al. (2020) 'Photodynamic therapy in oral lichen planus: A prospective case-controlled pilot study.', Scientific reports, 10(1), p. 1667.

R, O. et al. (2020) 'Evidence for a significant role of B-cells in the pathogenesis of oral lichen planus: Preliminary results of a cross-sectional study.', Journal of cutaneous pathology, 47(3), pp. 310–313.

R, S. et al. (2020) 'Prevalence of oral premalignant lesions and conditions among the population of Kanpur City, India: A cross-sectional study.', Journal of family medicine and primary care, 9(2), pp. 1080–1085.

R., A. et al. (2017) 'Association of Oral Lichen Planus and Electrocardiographic P-Wave Dispersion - An Original Research', Brazilian dental journal, 28(6), pp. 699–703.

R., C. et al. (2014) 'Cytokines and tumor markers in potentially malignant disorders and oral squamous cell carcinoma: A pilot study', Oral Diseases, 20(5), pp. 477–481.

R., G.-G. and J.L., P.-C. (2004) 'Lichen planus and associated diseases: A clinical-epidemiological study', Actas Dermo-Sifiliograficas, 95(3), pp. 154–160.

R., L. et al. (2004) 'Oral lichen planus and allergy to dental amalgam restorations', Archives of Dermatology, 140(12), pp. 1434–1438.

R., L. et al. (2006) 'A comparison of treatment of oral lichen planus with topical tacrolimus and triamcinolone acetonide ointment', Acta Dermato-Venereologica, 86(3), pp. 227–229.

R., M. et al. (2004) 'DNA content as a prognostic marker of oral lichen planus with a risk of cancer development', Analytical and Quantitative Cytology and Histology, 26(5), pp. 278–284.

R., M. et al. (2007) 'Immunohistochemical study on topoisomerase II$α$, Ki-67 and cytokeratin-19 in oral lichen planus lesions', Archives of dermatological research, 298(8), pp. 381–388.

R., M. et al. (2008) 'Desmocollin expression in oral atrophic lichen planus correlates with clinical behavior and DNA content', Journal of Cutaneous Pathology, 35(9), pp. 832–838.

R., M. et al. (2010) 'Caspase cascade pathways in apoptosis of oral lichen planus', Oral Surgery, Oral Medicine, Oral Pathology, Oral Radiology and Endodontology, 110(5), pp. 618–623.

R., R. et al. (1999) 'Malignant transformation of oral lichen planus', European Journal of Surgical Oncology, 25(5), pp. 520–523.

R., Y. et al. (2016) 'Oral lichen planus patients exhibit consistent chromosomal numerical aberrations: A follow-up analysis', Head and Neck, 38, pp. E741--E746.

R.A., S. et al. (2010) 'Oral lichen planus shows higher expressions of tumor suppressor gene products of p53 and p21 compared to oral mucositis. An immunohistochemical study', Archives of Oral Biology, 55(6), pp. 454–461.

R.B., B. et al. (1979) 'Incidence rate of oral lichen planus among Indian villagers', Acta Dermato-Venereologica, 59(3), pp. 255–257.

R.B., U. et al. (2010) 'Oxidative stress and antioxidant defense in oral lichen planus and oral lichenoid reaction', Scandinavian Journal of Clinical and Laboratory Investigation, 70(4), pp. 225–228.

R.F., B. et al. (1990) 'Oral lichen planus: epidemiological observations in Argentina', Revista de la Asociación Odontológica Argentina, 78(1), pp. 23–26.

R.L., R. et al. (2012) 'Randomized trial of aloe vera gel vs triamcinolone acetonide ointment in the treatment of oral lichen planus.', Quintessence international (Berlin, Germany : 1985), 43(9), pp. 793–800.

R.M., A. H. et al. (2012) ‘DNA polymorphisms and tissue cyclooxygenase-2 expression in oral lichen planus: A case-control study’, Journal of the european academy of dermatology and venereology, 26(9), pp. 1122–1126.

R.R., H. et al. (2017) ‘Glucosamine as a novel adjunctive therapy in symptomatic oral lichen planus’, Journal of Pharmaceutical Sciences and Research, 9(6), pp. 941–946.

R.R., S. et al. (2016) ‘The efficacy of topical hyaluronic acid 0.2% in the management of symptomatic oral lichen planus’, Journal of Clinical and Diagnostic Research, 10(1), pp. ZC46--ZC50.

R.S., G. et al. (2017) ‘Evaluation of the diagnostic efficacy and spectrum of autofluorescence of benign, dysplastic and malignant lesions of the oral cavity using VELscope’, Oral Oncology, 75, pp. 67–74.

Rabinovich, O. F. et al. (2016) ‘Photodynamic therapy in treatment of severe oral lichen planus’, Stomatologiia, 95(4), pp. 31–33.

Rabinovich, O. F., Khanukova, L. M. and Khamidulina, K. F. (1999) ‘[The characteristics of the immune status of patients with lichen ruber planus].’, Stomatologiia, 78(5), pp. 20–23.

Radden, B. G., & Reade, P. C. (1966). Oral lichen planus. The Medical Journal of Australia, 1(11), 441–445.

Radwan-Oczko, M., Kozłowski, Z. and Kazanowska, M. (2011) ‘Pathological changes of the oral mucosa in patients attending outpatient clinic of periodontology and oral pathology - the analysis of years 2006-2009 [Choroby błony śluzowej jamy ustnej pacjentów Poradni Chorób Przyzębia i Błony Śluzowej Jamy Ustnej - an’, Journal of Stomatology, 64(3), pp. 186–199.

Rai, B. et al. (2008) ‘Salivary vitamin e and c in lichen planus’, gomal journal of medical sciences, 6(2), pp. 91–92.

Rai, B. et al. (2010) ‘Possible action mechanism for curcumin in pre-cancerous lesions based on serum and salivary markers of oxidative stress.’, Journal of oral science, 52(2), pp. 251–256.

Rai, N. P. et al. (2016) ‘Relation between periodontal status and pre-cancerous condition (Oral lichen planus): A pilot study’, Advances in Clinical and Experimental Medicine, 25(4), pp. 763–766.

Rajan, B., Ahmed, J., Shenoy, N., Denny, C., Ongole, R., & Binnal, A. (2014). Assessment of quality of life in patients with chronic oral mucosal diseases: a questionnaire-based study. The Permanente Journal, 18(1), e123--127.

Ramadas, A. A. et al. (2016) ‘Systemic absorption of 0.1% triamcinolone acetonide as topical application in management of oral lichen planus’, Indian journal of dental research, 27(3), pp. 230–235.

Ramalingam, S. et al. (2018) ‘Role of Mast Cells in Oral Lichen Planus and Oral Lichenoid Reactions’, Autoimmune Diseases, 2018, p. 7936564.

Ramirez-Amador, V. et al. (1996) ‘Altered interface adhesion molecules in oral lichen planus.’, Journal of dental research, 2(3), pp. 188–192.

Ramon-Fluixa, C., Bagan-Sebastian, J., Milian-Masanet, M., & Scully, C. (1999). Periodontal status in patients with oral lichen planus: A study of 90 cases. Oral Diseases, 5(4), 303–306.

Ramon, C. et al. (2000) ‘Quantitative analysis of saliva in patients with oral lichen planus: A study of 100 cases’, Medicina Oral, 5(3), pp. 187–192.

Ranjbar, M. et al. (2018) ‘Micronuclei comparison in lichen planus and oral lichenoid responses’, Journal of Babol University of Medical Sciences, 20(12), pp. 7–12.

Rao, D. S., Ali, I. M., & Annigeri, R. G. (2017). Evaluation of diagnostic value of AgNOR and PAP in early detection of dysplastic changes in leukoplakia and lichen planus – a preliminary case–control study. Journal of Oral Pathology and Medicine, 46(1), 56–60.

Raybaud H, Olivieri CV, Lupi-Pegurier L, Pagnotta S, Marsault R, Cardot-Leccia N and Doglio A (2018), "Epstein-Barr Virus-Infected Plasma Cells Infiltrate Erosive Oral Lichen Planus", Journal of dental research. Vol. 97(13), pp. 1494-1500.

Razavi, S. M. et al. (2009) ‘Human papilloma virus as a possible factor in the pathogenesis of oral lichen planus.’, Dental research journal, 6(2), pp. 82–86.

Redder, C. P. et al. (2014) ‘Comparative analysis of cell proliferation ratio in plaque and erosive oral lichen planus: An immunohistochemical study.’, Dental research journal, 11(3), pp. 316–320.

Reddy, D. S. et al. (2012) ‘Evaluation of mast cells, eosinophils, blood capillaries in oral lichen planus and oral lichenoid mucositis’, Indian Journal of Dental Research, 23(5), pp. 695–696.

Regezi, J. A. et al. (1985) ‘Immunohistochemical staining of Langerhans cells and macrophages in oral lichen planus’, Oral Surgery Oral Medicine and Oral Pathology, 60(4), pp. 396–402.

Regezi, J. A. et al. (1996) ‘Vascular adhesion molecules in oral lichen planus.’, Oral surgery, oral medicine, oral pathology, oral radiology, and endodontics, 81(6), pp. 682–690.

Regnault, M. M. et al. (2017) ‘No detection of Merkel cell polyomavirus in oral lichen planus: Results of a preliminary study in a French cohort of patients’, Journal of Medical Virology, 89(11), pp. 2055–2057.

Rekha, V. R., Sunil, S. and Rathy, R. (2017) ‘Evaluation of oxidative stress markers in oral lichen planus.’, Journal of oral and maxillofacial pathology, 21(3), pp. 387–393.

Rezazadeh, F. et al. (2018) ‘Plasma level of trace elements in patients with oral lichen planus’, Iranian Journal of Dermatology, 21(2), pp. 54–58.

Rezazadeh, F., Salehi, S. and Rezaee, M. (2019) 'Salivary Level of Trace Element in Oral Lichen Planus, A Premalignant Condition.', Asian Pacific journal of cancer prevention, 20(7), pp. 2009–2013.

Rezazadeh, F., Shahbazi, F. and Andisheh-Tadbir, A. (2017) 'Evaluation of salivary level of IL-10 in patients with oral lichen planus, a preliminary investigation', Comparative Clinical Pathology, 26(3), pp. 531–534.

Rhodus, N. L. et al. (2005) 'A comparison of the pro-inflammatory, NF-κB-dependent cytokines: TNF-alpha, IL-1-alpha, IL-6, and IL-8 in different oral fluids from oral lichen planus patients', Clinical Immunology, 114(3), pp. 278–283.

Rhodus, N. L. et al. (2006) 'Proinflammatory cytokine levels in saliva before and after treatment of (erosive) oral lichen planus with dexamethasone', Oral Diseases, 12(2), pp. 112–116.

Rhodus, N. L., Cheng, B. and Ondrey, F. (2007) 'Th1/Th2 cytokine ratio in tissue transudates from patients with oral lichen planus.', Mediators of inflammation, 2007, p. 19854.

Riaz, H. M. A. et al. (2017) 'Efficacy of pimecrolimus cream and triamcinolone acetonide paste in the treatment of symptomatic oral lichen planus', Medical Forum Monthly, 28(12), pp. 76–80.

Riggio, M. P. et al. (2000) 'Detection of Helicobacter pylori DNA in recurrent aphthous stomatitis tissue by PCR', Journal of Oral Pathology and Medicine, 29(10), pp. 507–513.

Rimkevičius, A. et al. (2017) 'Oral lichen planus: A 4-year clinical follow-up study', Turkish Journal of Medical Sciences, 47(2), pp. 514–522.

Rinaggio, J., Crossland, D. M. and Zeid, M. Y. (2007) 'A determination of the range of oral conditions submitted for microscopic and direct immunofluorescence analysis.', Journal of periodontology, 78(10), pp. 1904–1910.

Riordain, R. N. and McCreary, C. (2011) 'Validity and reliability of a newly developed quality of life questionnaire for patients with chronic oral mucosal diseases', Journal of oral pathology & medicine, 40(8), pp. 604–609.

Rivas-Tolosa, N. et al. (2016) 'Antimalarial Drugs for the Treatment of Oral Erosive Lichen Planus.', Dermatology (Basel, Switzerland), 232(1), pp. 86–90.

Robertson, W. D. et al. (1993) 'Immunohistochemical study of oral keratoses including lichen planus', Journal of Oral Pathology and Medicine, 22(4), pp. 180–182.

Robledo-Sierra, J. et al. (2015) 'Clinical characteristics of patients with concomitant oral lichen planus and thyroid disease', Oral surgery, oral medicine, oral pathology and oral radiology, 120(5), pp. 602–608.

Robledo-Sierra, J. et al. (2018) 'A mechanistic linkage between oral lichen planus and autoimmune thyroid disease', Oral Diseases, 24(6), pp. 1001–1011.

Rode, M. et al. (2002) ‘Malignant potential of the reticular form of oral lichen planus over a 25-year observation period in 55 patients from Slovenia.’, Journal of oral science, 44(2), pp. 109–111.

Rode, M. et al. (2006) ‘Image cytometric evaluation of nuclear texture features and DNA content of the reticular form of oral lichen planus’, Analytical and Quantitative Cytology and Histology, 28(5), pp. 262–268.

Rode, M. et al. (2008) ‘DNA ploidy and nuclear texture features of the reticular form of oral lichen planus’, Zdravniski vestnik-slovenian medical journal, 77(1), pp. 13–17.

Rodriguez-Nunez, I. et al. (2001) ‘Peripheral T-cell subsets in patients with reticular and atrophic-erosive oral lichen planus.’, Oral surgery, oral medicine, oral pathology, oral radiology, and endodontics, 91(2), pp. 180–188.

Rodstrom, P. O. et al. (2001) ‘Erosive oval lichen planus and salivary cortisol’, Journal of oral pathology & medicine, 30(5), pp. 257–263.

Rogers, R. S. & Van Hale, H. M. (1986) ‘Immunopathologic diagnosis of oral mucosal inflammatory diseases’, Australasian Journal of Dermatology, 27(2), pp. 51–57.

Roitberg-Tambur, A. et al. (1994) ‘Serologic and molecular analysis of the HLA system in Israeli Jewish patients with oral erosive lichen planus.’, Tissue antigens, 43(4), pp. 219–223.

Rokni, G. R. et al. (2017) ‘Evaluation of Serum Homocysteine Levels in Patients with Cutaneous-Oral Lichen Planus and Psoriasis Patients’, Galen medical journal, 6(3), pp. 226–232.

Romano, F. et al. (2019) ‘Effect of a structured plaque control on MMP-1 and MMP-9 crevicular levels in patients with desquamative gingivitis associated with oral lichen planus.’, Clinical oral investigations, 23(6), pp. 2651–2658.

Ron’, G. I., Akmalova, G. M., & Emel’yanova, I. V. (2015). Evaluation of the clinical efficacy of a new composition of tizol with triamcinolon in complex treatment of patients with erosive ulcerous form of lichen planus of the oral mucosa. Stomatologiia, 94(2), 13–15.

Roosaar, A. et al. (2006) ‘On the natural course of oral lichen lesions in a Swedish population-based sample.’, Journal of oral pathology & medicine: official publication of the International Association of Oral Pathologists and the American Academy of Oral Pathology, 35(5), pp. 257–261.

Rosa, E. A., Hurtado-Puerto, A. M., Falcao, D. P., Brietzke, A. P., De Almeida Prado Franceschi, L. E., Cavalcanti Neto, F. F., Tiziane, V., Carneiro, F. P., … Amorim, R. F. B. (2018). Oral lichen planus and malignant transformation: The role of p16, Ki-67, Bub-3 and SOX4 in assessing precancerous potential. Experimental and Therapeutic Medicine, 15(5), 4157–4166.

Rubaci, A. H., Kazancioglu, H. O., Olgac, V., & Ak, G. (2012). The roles of matrix metalloproteinases-2, -7, -10 and tissue inhibitor of metalloproteinase-1 in the

pathogenesis of oral lichen planus. Journal of Oral Pathology and Medicine, 41(9), 689–696.

Rusanen, P. et al. (2017) 'TLR1-10, NF-kappaB and p53 expression is increased in oral lichenoid disease.', PloS one, 12(7), pp. e0181361–e0181361.

S, B. et al. (2020) 'Comparative Evaluation of Mitochondrial Antioxidants in Oral Potentially Malignant Disorders.', The Kurume medical journal, 66(1), pp. 15–27.

S, G. et al. (2020) 'Prevalence of and related risk factors in oral mucosa diseases among residents in the Baoshan District of Shanghai, China.', PeerJ, 8, p. e8644.

S, M. et al. (2020) 'Cardiovascular and Metabolic Risk Assessment in Patients with Lichen Planus: A Tertiary Care Hospital-based Study from Northern India.', Indian dermatology online journal, 11(2), pp. 158–166.

S, N. et al. (2020) 'Dermoscopy of Lip Lichen Planus-A Descriptive Study.', Dermatology practical & conceptual, 10(4), p. e2020076.

S, P. et al. (2020) 'CD146 expression in oral lichen planus and oral cancer.', Clinical oral investigations, 24(1), pp. 325–332.

S, R. et al. (2021) 'Optical diagnosis of oral lichen planus: A clinical study on the use of autofluorescence spectroscopy combined with multivariate analysis.', Spectrochimica acta. Part A, Molecular and biomolecular spectroscopy, 248, p. 119240.

S, S. et al. (2020) 'Analysis of the Efficacy of Two Treatment Protocols for Patients with Symptomatic Oral Lichen Planus: A Randomized Clinical Trial.', International journal of environmental research and public health, 18(1).

S.-C., L. et al. (2012) 'Aberrant keratinization of reticular oral lichen planus is related to elastolysis', Oral Surgery, Oral Medicine, Oral Pathology and Oral Radiology, 113(6), pp. 808–816.

S.-H., J. et al. (2016) 'Topical sulfasalazine for unresponsive oral lichen planus', Quintessence international, 47(4), pp. 319–327.

S., A. et al. (2013) 'Comparative study of the therapeutic efficacy of the dapsone (alone), dapsone plus tacrolimus (topical) and dapsone plus triamcinolone (topical) in oral lichen planus', Indian Journal of Public Health Research and Development, 4(1), pp. 83–86.

S., A. et al. (2014) 'Psychological screening test results for stress, depression, and anxiety are variably associated with clinical severity of recurrent aphthous stomatitis and oral lichen planus', The journal of evidence-based dental practice, 14(4), pp. 206–208.

S., A. et al. (2016) 'Phenotypic variability and therapeutic implications of Candida species in patients with oral lichen planus', Biotechnic & histochemistry : official publication of the Biological Stain Commission, 91(4), pp. 237–241.

S., B. et al. (1995) 'Alteration of cytokeratin expression in oral lichen planus.', Oral surgery, oral medicine, oral pathology, oral radiology, and endodontics, 79(2), pp. 207–215.

S., B. et al. (2016) ‘Evaluation of Salivary Secretor Status of Blood Group Antigens in Patients with Oral Lichen Planus’, Medical Principles and Practice, 25(3), pp. 266–269.

S., B., J.B., T., et al. (2017) ‘Prevalence of oral lichen planus in Iranian children and adolescents: a 12-year retrospective study’, European archives of paediatric dentistry : official journal of the European Academy of Paediatric Dentistry, 18(6), pp. 419–422.

S., B., P., T., et al. (2017) ‘Assessment of Uric Acid Level in the Saliva of Patients with Oral Lichen Planus’, Medical Principles and Practice, 26(1), pp. 57–60.

S., B., S., A.-M., et al. (2017) ‘Comparing clinical effects of photodynamic therapy as a novel method with topical corticosteroid for treatment of Oral Lichen Planus’, Photodiagnosis and Photodynamic Therapy, 20, pp. 159–164.

S., E. et al. (2009) ‘Micronuclear and sister chromatid exchange analyses in peripheral lymphocytes of patients with oral lichen planus - A pilot study’, Oral Diseases, 15(7), pp. 499–504.

S., E. et al. (2011) ‘Evaluation of oxidative stress and antioxidant profile in patients with oral lichen planus’, Journal of Oral Pathology and Medicine, 40(4), pp. 286–293.

S., G. et al. (1989) ‘Role of direct immunofluorescence in the diagnosis of oral lichen planus’, Minerva stomatologica, 38(5), pp. 509–514.

S., G.-A. et al. (2019) ‘Human papillomavirus in premalignant oral lesions: No evidence of association in a Spanish cohort’, PLoS ONE, 14(1), pp. e0210070–e0210070.

S., I. et al. (2019) ‘Discrimination of oral squamous cell carcinoma from oral lichen planus by salivary metabolomics’, Oral diseases.

S., L. et al. (2008) ‘COX-2 expression in oral lichen planus’, Dermatology, 217(2), pp. 150–155.

S., M. et al. (2013) ‘Potentially malignant character of oral lichen planus and lichenoid lesions’, Revue de Stomatologie, de Chirurgie Maxillo-faciale et de Chirurgie Orale, 114(5), pp. 293–298.

S., M. et al. (2018) ‘Efficacy of photodynamic therapy or low level laser therapy against steroid therapy in the treatment of erosive-atrophic oral lichen planus’, Photodiagnosis and Photodynamic Therapy, 21, pp. 404–408.

S., N. et al. (2003) ‘Thermal sensation and pain in oral lichen planus and lichenoid reaction’, Journal of Oral Pathology and Medicine, 32(1), pp. 41–45.

S., R. et al. (2005) ‘Histological evidence for lichen planus in clinically normal perilesional tissue’, International Journal of Oral and Maxillofacial Surgery, 34(6), pp. 674–677.

S., R. et al. (2015) ‘Efficacy of topical tacrolimus for oral lichen planus: Real-life experience in a retrospective cohort of patients with a review of the literature’, Journal of the European Academy of Dermatology and Venereology, 29(6), pp. 1107–1113.

S., S. J. et al. (1985) 'A prospective follow-up study of 570 patients with oral lichen planus: persistence, remission, and malignant association', Oral Surgery Oral Medicine and Oral Pathology, 60(1), pp. 30–34.

S., T. et al. (2016) 'Oral lichen planus and its malignant transformation', Journal of Pharmaceutical Sciences and Research, 8(10), pp. 1226–1228.

S., U. et al. (2005) 'Formulation and efficacy of triamcinolone acetonide mouthwash for treating oral lichen planus', American Journal of Health-System Pharmacy, 62(5), pp. 485–491.

S., X. et al. (2012) 'Implications of Th1 and Th17 cells in pathogenesis of oral lichen planus', Journal of Huazhong University of Science and Technology - Medical Science, 32(3), pp. 451–457.

S., X. et al. (2014) 'Expressions of RORγT; and FOXP3 and clinical significance in patients with oral lichen planus', Shanghai kou qiang yi xue = Shanghai journal of stomatology, 23(4), pp. 472–476.

S.A., S. et al. (2014) 'Oral lichen planus: Different trends in treatment', Journal of Medical Sciences (Peshawar), 22(3), pp. 137–142.

S.C., G. et al. (1994) 'P53 and PCNA expression in carcinogenesis of the oropharyngeal mucosa', European Journal of Cancer Part B: Oral Oncology, 30(6), pp. 419–423.

S.I., K. et al. (2013) 'Photodynamic treatment of oral lichen planus', Oral Surgery, Oral Medicine, Oral Pathology and Oral Radiology, 115(1), pp. 62–70.

S.J., F. et al. (2017) 'Identification of AgNORs and cytopathological changes in oral lichen planus lesions', Acta Histochemica, 119(1), pp. 32–38.

S.J., S. et al. (2013) 'Cost-effectiveness of personalized plaque control for managing the gingival manifestations of oral lichen planus: A randomized controlled study', Journal of Clinical Periodontology, 40(9), pp. 859–867.

S.J., S. et al. (2015) 'The impact of structured plaque control for patients with gingival manifestations of oral lichen planus: a randomized controlled study', Journal of clinical periodontology, 42(4), pp. 356–362.

S.K.S., H. et al. (2015) 'Clinico-pathological study to evaluate oral lichen planus for the establishment of clinical and histopathological diagnostic criteria', Turk Patoloji Dergisi, 31(1), pp. 24–29.

S.M., A. et al. (2019) 'Evaluating the role of tissue microRNA-27b as a diagnostic marker for oral lichen planus and possible correlation with CD8', Journal of Oral Pathology and Medicine, 48(1), pp. 68–73.

S.M., S., G., N. and T.S., A. (2013) 'White lesions in the oral cavity: A clinicopathological study from a tertiary care dermatology centre in Kerala, India', Indian Journal of Dermatology, 58(4), pp. 269–274.

S.S., D. et al. (1996) ‘Cytogenetic damage in exfoliated oral mucosal cells and circulating lymphocytes of patients suffering from precancerous oral lesions’, Cancer Letters, 109(1), pp. 9–14.

S.S., I. et al. (2012) ‘Topical pimecrolimus effect on Fas inducing apoptosis in oral lichen planus: A clinical immunohistochemical study’, Journal of Oral Pathology and Medicine, 41(4), pp. 315–321.

S.S., P. et al. (1985) ‘The use of the lectin Ulex europeus to study epithelial cell differentiation in neoplastic and non-neoplastic oral white lesions’, Journal of Pathology, 147(3), pp. 173–179.

S.Y., L. et al. (1995) ‘Dramatic response to levamisole and low-dose prednisolone in 23 patients with oral lichen planus: a 6-year prospective follow-up study.’, Oral surgery, oral medicine, oral pathology, oral radiology, and endodontics, 80(6), pp. 705–709.

S.Y., L. et al. (1998) ‘Response to levamisole and low-dose prednisolone in 41 patients with chronic oral ulcers: a 3-year open clinical trial and follow-up study.’, Oral surgery, oral medicine, oral pathology, oral radiology, and endodontics, 86(4), pp. 438–445.

Saad, I. et al. (2018) ‘Evaluation of Serum Desmoglein 1 and Desmoglein 3 in Oral Erosive Lichen Planus before and after Topical Application of Tacrolimus’, The journal of contemporary dental practice, 19(10), pp. 1204–1213.

Saawarn, N. et al. (2011) ‘Lycopene in the management of oral lichen planus: A placebo-controlled study’, Indian Journal of Dental Research, 22(5), pp. 639–643.

Sadaksharam, J. et al. (2012) ‘Treatment of oral lichen planus with methylene blue mediated photodynamic therapy - a clinical study’, Photodermatology Photoimmunology and Photomedicine, 28(2), pp. 97–101.

Sadeghian, R. et al. (2019) ‘Comparison of therapeutic effect of mucoadhesive nano-triamcinolone gel and conventional triamcinolone gel on oral lichen planus’, Dental Research Journal, 16(5), pp. 277–282.

Saghravanian, N. et al. (2017) ‘The Epidemiological Pattern of Premalignant and Malignant Epithelial Lesions in Northeast of Iran: A 43-Year Evaluation’, International journal of cancer management, 10(8).

Sahebjamee, M. et al. (2007) ‘Presence of Epstein-Barr virus in oral lichen planus and normal oral mucosa’, Iranian journal of public health, 36(2), pp. 92–98.

Sahebjamiee, M. et al. (2015) ‘Prevalence of human papillomavirus in oral lichen planus in an Iranian cohort.’, Journal of oral and maxillofacial pathology, 19(2), pp. 170–174.

Salazar-Sánchez, N. et al. (2010) ‘Efficacy of topical Aloe vera in patients with oral lichen planus: A randomized double-blind study’, Journal of Oral Pathology and Medicine, 39(10), pp. 735–740.

Salem, A et al. (2017) ‘Histamine metabolism and transport are deranged in human keratinocytes in oral lichen planus’, British Journal of Dermatology, 176(5), pp. 1213–1223.

Salem, A. et al. (2015) ‘Histamine H4 receptor in oral lichen planus’, Oral Diseases, 21(3), pp. 378–385.

Salem, A., Almahmoudi, R., Vehvilainen, M., & Salo, T. (2018). Role of the high mobility group box 1 signalling axes via the receptor for advanced glycation end-products and toll-like receptor-4 in the immunopathology of oral lichen planus: a potential drug target? European Journal of Oral Sciences, 126(3), 244–248.

Salem, Abdelhakim et al. (2017) ‘Altered Expression of Toll-like Receptors in Human Oral Epithelium in Oral Lichenoid Reactions.’, The American Journal of dermatopathology, 39(11), pp. 811–818.

Sameera, A. et al. (2019). Molecular detection of human papillomavirus DNA in oral lichen planus patients. Journal of Clinical and Diagnostic Research, 13(1), ZC20--ZC24.

Sanchez-Siles, M. et al. (2011) ‘A novel application of the buccal micronucleus cytome assay in oral lichen planus: A pilot study’, Archives of Oral Biology, 56(10), pp. 1148–1153.

Sánchez-Siles, M. et al. (2019) ‘Genotyping of the C>T allele of rs16906252, predictor of O16-methylguanine-DNA methyltransferase (MGMT) promoter methylation status, in erosive atrophic lesions of oral lichen planus’, International Journal of Dermatology, 58(9), pp. 1078–1082.

Sanli H., Çetinkaya H., Tuȓsen U., Kaya M., Kuzu I, Gürle A. (2002), "Upper gastrointestinal findings in oral lichen planus", Turkish Journal of Gastroenterology. H. Çetinkaya, Ankara Universitesi Tip Fakultesi, Ibn-i Sina Hastanesi, Gastroenteroloji Klinigi, Ankara, Turkey Vol. 13(1), pp. 31-34.

Santarelli, A., Mascitti, M., Rubini, C., Bambini, F., Zizzi, A., Offidani, A., Ganzetti, G., Laino, L., Cicciu, M., & Lo Muzio, L. (2015). Active inflammatory biomarkers in oral lichen planus. International Journal of Immunopathology and Pharmacology, 28(4), 562–568.

Saraswathi, T. R. et al. (2006) ‘Prevalence of oral lesions in relation to habits: Cross-sectional study in South India.’, Indian journal of dental research: official publication of Indian Society for Dental Research, 17(3), pp. 121–125.

Saravi, Z. Z. et al. (2017) ‘VEGFR-3 expression in oral lichen planus’, Asian Pacific Journal of Cancer Prevention, 18(2), pp. 381–384.

Sardella, A., Demarosi, F., Oltolina, A., Rimondini, L., & Carrassi, A. (1998). Efficacy of topical mesalazine compared with clobetasol propionate in treatment of symptomatic oral lichen planus. Oral Diseases, 4(4), 255–259.

Sarper, D. A. et al. (2004) ‘HLA antigen distribution in erosive oral lichen planus’, Genes and immunity, 5(1), pp. S47–S47.

Saruhanoğlu, A., Ergun, S., Kaya, M., Warnakulasuriya, S., Erbağcı, M., Öztürk, Ş., Deniz, E., Özel, S., Çefle, K., Palanduz, Ş., & Tanyeri, H. (2014). Evaluation of micronuclear frequencies in both circulating lymphocytes and buccal epithelial cells of

patients with oral lichen planus and oral lichenoid contact reactions. Oral Diseases, 20(5), 521–527.

Saunders H, J.A.G. B, Cooper S, Hollowood K, Sherman V & Wojnarowska F (2010), "The period prevalence of oral lichen planus in a cohort of patients with vulvar lichen sclerosus", Journal of the European Academy of Dermatology and Venereology. Vol. 24(1), pp. 18-21.

Scalf, L. A. et al. (2001) ‘Dental metal allergy in patients with oral, cutaneous, and genital lechenoid reactions’, American Journal of Contact Dermatitis, 12(3), pp. 146–150.

Scardina, G. A. et al. (2006) ‘A randomized trial assessing the effectiveness of different concentrations of isotretinoin in the management of lichen planus’, International Journal of Oral and Maxillofacial Surgery, 35(1), pp. 67–71.

Scardina, G. A. et al. (2009) ‘Angiogenesis of oral lichen planus: A possible pathogenetic mechanism’, Medicina Oral, Patologia Oral y Cirugia Bucal, 14(11), pp. e558--e562.

Scardina, G. A., & Messina, P. (2009). Morphological characteristics of microcirculation in oral lichen planus involving the lateral border of the tongue. Journal of Oral Science, 51(2), 193–197.

Scheifele, C. et al. (2004) ‘The sensitivity and specificity of the OralCDx technique: Evaluation of 103 cases’, Oral Oncology, 40(8), pp. 824–828.

Schiodt, M. and M., S. (1984) ‘Oral discoid lupus erythematosus. III. A histopathologic study of sixty-six patients’, Oral Surgery Oral Medicine and Oral Pathology, 57(3), pp. 281–293.

Schiodt, M. et al. (1976) ‘Histologic differential diagnostic problems for oral discoid lupus erythematosus’, International Journal of Oral Surgery, 5(5), pp. 250–252.

Schirner, E., Hornstein, O. P., & Simon, M. J. (1981). Etiology and prognosis of oral lichen planus; preliminary results of a controlled prospective study. Deutsche Zahnärztliche Zeitschrift, 36(3), 130–135.

Schreurs, O., Karatsaidis, A. and Schenck, K. (2016) ‘Phenotypically non-suppressive cells predominate among FoxP3-positive cells in oral lichen planus.’, Journal of oral pathology & medicine: official publication of the International Association of Oral Pathologists and the American Academy of Oral Pathology, 45(10), pp. 766–773.

Schuurs, A. H., & van Joost, T. (2000). Orale lichen planus, amalgaam en andere vulmaterialen [Oral lichen planus, amalgam and other restorative materials]. Nederlands Tijdschrift Voor Tandheelkunde, 107(5), 198–202.

Scrobota, I. et al. (2011) ‘Histopathological aspects and local implications of oxidative stress in patients with oral lichen planus’, Romanian Journal of Morphology and Embryology, 52(4), pp. 1305–1309.

Seckin Ertugrul, A. et al. (2013) ‘Periodontopathogen profile of healthy and oral lichen planus patients with gingivitis or periodontitis’, International Journal of Oral Science, 5(2), pp. 92–97.

Sekine, R. et al. (2015) ‘A study on the intrapapillary capillary loop detected by narrow band imaging system in early oral squamous cell carcinoma’, Journal of oral and maxillofacial surgery medicine and pathology, 27(5), pp. 624–630.

Sethi Ahuja, U. et al. (2020) ‘Comparative evaluation of effectiveness of autologous platelet rich plasma and intralesional corticosteroids in the management of erosive oral Lichen planus- a clinical study.’, Journal of oral biology and craniofacial research, 10(4), pp. 714–718.

Seyedmajidi, M. et al. (2011) ‘Expression of P53 and P63 in oral licen planus and oral licenoid lesions’, Journal of Babol University of Medical Sciences, 13(4), pp. 7–13.

Seyedmajidi, M. et al. (2013) ‘VCAM1 and ICAM1 expression in oral lichen planus.’, International journal of molecular and cellular medicine, 2(1), pp. 34–40.

Seyedmajidi, M. et al. (2016) ‘Investigating Immunohistochemical Expression of cyclooxygenase-2 in oral lichen planus’, International journal of advanced biotechnology and research, 7(3), pp. 2046–2054.

Seyhan, M. et al. (2007) ‘High prevalence of glucose metabolism disturbance in patients with lichen planus.’, Diabetes research and clinical practice, 77(2), pp. 198–202.

Sha, Y. Q. (1990) ‘[Candida albicans infection in oral lichen planus].’, Zhonghua kou qiang yi xue za zhi = Zhonghua kouqiang yixue zazhi = Chinese journal of stomatology, 25(5), pp. 297–298.

Shahidi-Dadras, M. et al. (2016) ‘Evaluation of secretory state in patients with oral lichen planus: A case-control study’, Archives of Iranian Medicine, 19(1), pp. 21–24.

Shahidi, M. et al. (2017) ‘Predictive value of salivary microRNA-320a, vascular endothelial growth factor receptor 2, CRP and IL-6 in Oral lichen planus progression’, Inflammopharmacology, 25(5), pp. 577–583.

Shailaja, G. et al. (2015) ‘Estimation of malignant transformation rate in cases of oral epithelial dysplasia and lichen planus using immunohistochemical expression of Ki-67, p53, BCL-2, and BAX markers.’, Dental research journal, 12(3), pp. 235–242.

Shan, J. et al. (2013) ‘Proliferation and apoptosis of peripheral blood mononuclear cells in patients with oral lichen planus’, Inflammation, 36(2), pp. 419–425.

Shariati, M. et al. (2018) ‘Association between oral lichen planus and Epstein–Barr virus in Iranian patients’, Journal of Research in Medical Sciences, 23(3), p. 24.

Sharma, R. et al. (2011) ‘Role of mast cells in pathogenesis of oral lichen planus.’, Journal of oral and maxillofacial pathology, 15(3), pp. 267–271.

Sheelam, S. et al. (2018) ‘Role of cell proliferation and vascularity in malignant transformation of potentially malignant disorders.’, Journal of oral and maxillofacial pathology, 22(2), p. 281.

Shen H., Liu Q., Huang P, Fan H, Zang F., Liu M., Zhuo L., Wu J., Wu G., Yu R. and Yang J. (2020), "Vitamin D receptor genetic polymorphisms are associated with oral

lichen planus susceptibility in a Chinese Han population.", BMC oral health. Vol. 20(1), pp. 26.

Shen, Z. et al. (2014) ‘Expression of Foxp3 and interleukin-17 in lichen planus lesions with emphasis on difference in oral and cutaneous variants.’, Archives of dermatological research, 306(5), pp. 441–446.

Shen, Z. et al. (2016) ‘Aberrant expression of interleukin-22 and its targeting microRNAs in oral lichen planus: a preliminary study’, Journal of Oral Pathology and Medicine, 45(7), pp. 523–527.

Shen, Z. et al. (2016) ‘Altered expression of interleukin-17A and its targeting microRNAs in oral lichen planus: a pilot study.’, Oral surgery, oral medicine, oral pathology and oral radiology, 122(5), pp. 619--624.e1.

Shi, P. et al. (2010) ‘Podoplanin and ABCG2: Malignant transformation risk markers for oral lichen planus’, Cancer Epidemiology Biomarkers and Prevention, 19(3), pp. 844–849.

Shibahara, T. et al. (2014) ‘Narrow-band imaging system with magnifying endoscopy for early oral cancer.’, The Bulletin of Tokyo Dental College, 55(2), pp. 87–94.

Shimada, K. et al. (2018) ‘Phenotypic alteration of basal cells in oral lichen planus; switching keratin 19 and desmoglein 1 expression’, Journal of oral science, 60(4), pp. 507–513.

Shin, J. A., Seo, J. M., Oh, S., Cho, S. D., & Lee, K. E. (2016) ‘Myeloid cell leukemia-1 is a molecular indicator for malignant transformation of oral lichen planus’, Oncology Letters, 11(2), pp. 1603–1607.

Shirazian, S. et al. (2017) ‘Comparison of interleukin 17 and 22 in saliva of oral lichen planus patients with healthy people’, Bioscience biotechnology research communications, 10(3), pp. 587–591.

Shirol, P. D. et al. (2015) ‘Fibrinogen Demonstration in Oral Lichen Planus: An Immunofluorescence Study on Archival Tissues’, The journal of contemporary dental practice, 16(10), pp. 824–828.

Shirzad, A. et al. (2014) ‘Salivary total antioxidant capacity and lipid peroxidation in patients with erosive oral lichen planus.’, Journal of dental research, dental clinics, dental prospects, 8(1), pp. 35–39.

Shirzad, A., Bijani, A., Mehryari, M., Motallebnejad, M., & Mohsenitavakoli, S. (2018). Validity and reliability of the persian version of the chronic oral mucosal diseases questionnaire. Caspian Journal of Internal Medicine, 9(2), 127–133.

Shiva, A et al. (2020). Serum and Salivary Level of Nitric Oxide (NOx) and CRP in Oral Lichen Planus (OLP) Patients. Journal of Dentistry (Shiraz, Iran), 21(1), 6–11.

Shiva, A. et al. (2018) ‘Immunohistochemical Study of p53 Expression in Patients with Erosive and Non-Erosive Oral Lichen Planus.’, Journal of dentistry (Shiraz, Iran), 19(2), pp. 118–123.

Shukurova, U. A. and Bekjanova, O. E. (2016) 'Oral Lichen Planus and Features in the Short Chain Fatty Acid Pattern Produced by Colonic Fermentation', International journal of biomedicine, 6(1), pp. 74–77.

Siar, C. H. et al. (2013) 'Identifying factors associated with diagnostic concordance/discordance in oral lichen planus', Journal of Oral and Maxillofacial Surgery, 71(10), pp. 1688–1693.

Siar, C. H., Mah, M. C., & Gill, P. P. (2011). Risk of the contralateral mucosa in patients with oral potentially malignant disorders. Asian Pacific Journal of Cancer Prevention, 12(3), 631–635

Siar, C. H., Mah, M. C., & Gill, P. P. (2012). Prevalence of bilateral "mirror-image" lesions in patients with oral potentially malignant epithelial lesions. European Archives of Otorhino-Laryngology, 269(3), 999–1004.

Sidikov, A et al. (2020). The new differential diagnostic test for the lichenoid drug eruption. Dermatologic Therapy, 33(6), e13784.

Sieg, P. et al. (1995) 'Topical cyclosporin in oral lichen planus: A controlled, randomized, prospective trial', British Journal of Dermatology, 132(5), pp. 790–794.

Silverman, S. J. et al. (1974) 'Studies on oral lichen planus. II. Follow up on 200 patients, clinical characteristics, and associated malignancy', Oral Surgery Oral Medicine and Oral Pathology, 37(5), pp. 705–710.

Simark-Mattsson, C. and Eklund, C. (2013) 'Reduced immune responses to purified protein derivative and Candida albicans in oral lichen planus.', Journal of oral pathology & medicine: official publication of the International Association of Oral Pathologists and the American Academy of Oral Pathology, 42(9), pp. 691–697.

Simark-Mattsson, C. et al. (1999) 'Distribution of interleukin-2, -4, -10, tumour necrosis factor-alpha and transforming growth factor-beta mRNAs in oral lichen planus.', Archives of oral biology, 44(6), pp. 499–507.

Singh, A. R. et al. (2017) 'Efficacy of steroidal vs non-steroidal agents in oral lichen planus: A randomised, open-label study', Journal of Laryngology and Otology, 131(1), pp. 69–76.

Singh, P. et al. (2017) 'Elucidating the role of Cyclooxygenase-2 in the pathogenesis of oral lichen planus – an immunohistochemical study with supportive histochemical analysis', Journal of Oral Pathology and Medicine, 46(5), pp. 381–386.

Sinon, S. H. et al. (2016) 'Downregulation of toll-like receptor-mediated signalling pathways in oral lichen planus', Journal of Oral Pathology and Medicine, 45(1), pp. 28–34.

Siponen, M. et al. (2012) 'TLR4 and TLR9 are induced in oral lichen planus.', Journal of oral pathology & medicine: official publication of the International Association of Oral Pathologists and the American Academy of Oral Pathology, 41(10), pp. 741–747.

Siponen, M. et al. (2016) ‘Cathepsin K expression is increased in oral lichen planus’, Journal of Oral Pathology and Medicine, 45(10), pp. 758–765.

Sivaraman, S. et al. (2016) ‘A randomized triple-blind clinical trial to compare the effectiveness of topical triamcinolone acetonate (0.1%), clobetasol propionate (0.05%), and tacrolimus orabase (0.03%) in the management of oral lichen planus’, Journal of Pharmacy and Bioallied Sciences, 8, pp. S86--S89.

SJ, K. et al. (2020) ‘Comparison of oral Nano-Curcumin with oral prednisolone on oral lichen planus: a randomized double-blinded clinical trial.’, BMC complementary medicine and therapies, 20(1), p. 328.

Sklavounou-Andrikopoulou, A., Chrysomali, E., Iakovou, M., Garinis, G. A., & Karameris, A. (2004). Elevated serum levels of the apoptosis related molecules TNF-α, Fas/Apo-1 and Bcl-2 in oral lichen planus. Journal of Oral Pathology and Medicine, 33(7), 386–390.

Sklavounou, A. D., Laskaris, G., & Angelopoulos, A. P. (1983). Serum immunoglobulins and complement (C’3) in oral lichen planus. Oral Surgery Oral Medicine and Oral Pathology, 55(1), 47–51.

Sklavounou, A., Chrysomali, E., Scorilas, A., & Karameris, A. (2000). TNF-α expression and apoptosis-regulating proteins in oral lichen planus: A comparative immunohistochemical evaluation. Journal of Oral Pathology and Medicine, 29(8), 370–375.

Sklavounou, A., Laskaris, G. and Angelopoulos, A. P. (1981) ‘Serum immunoglobulins and complement (c-3) in oral lichen planus’, Journal of oral pathology & medicine, 10(5), p. 362.

Skoglund, A. (1994) ‘Value of epicutaneous patch testing in patients with oral, mucosal lesions of lichenoid character.’, Scandinavian journal of dental research, 102(4), pp. 216–222.

Skrinjar, I. et al. (2019) ‘Salivary Cortisol Levels in Patients with Oral Lichen Planus-A Pilot Case-Control Study.’, Dentistry journal, 7(2).

Sloberg, K. et al. (1979) ‘Topical tretinoin therapy and oral lichen planus’, Archives of Dermatology, 115(6), pp. 716–718.

Sloberg, K. et al. (1983) ‘Severe oral lichen planus: remission and maintenance with vitamin A analogues’, Journal of Oral Pathology, 12(6), pp. 473–477.

Sloberg, K. et al. (1984) ‘Assessment of Langerhans’ cells in oral lichen planus using monoclonal antibodies’, Journal of Oral Pathology, 13(5), pp. 516–524.

Sobaniec, S. et al. (2013) ‘Clinical assessment of the efficacy of photodynamic therapy in the treatment of oral lichen planus’, Lasers in Medical Science, 28(1), pp. 311–316.

Sonthalia, S. et al. (2012) ‘Comparative efficacy of tacrolimus 0.1% ointment and clobetasol propionate 0.05% ointment in oral lichen planus: A randomized double-blind trial’, International Journal of Dermatology, 51(11), pp. 1371–1378.

Souza, M. M. et al. (2018) ‘Salivary proteomics in lichen planus: A relationship with pathogenesis?’, Oral Diseases, 24(5), pp. 784–792.

Spadari, F. et al. (2015) ‘Low basal salivary flow and Burning Mouth Syndrome: New evidence in this enigmatic pathology’, Journal of Oral Pathology and Medicine, 44(3), pp. 229–233.

Splieth, C. H. et al. (2007) ‘Prevalence of oral mucosal lesions in a representative population.’, Quintessence international (Berlin, Germany : 1985), 38(1), pp. 23–29.

Squarzanti, D. F., Cena, T., Sorrentino, R., Migliario, M., Chiocchetti, A., Rimondini, L., Azzimonti, B., & Valente, G. (2019). Implications on pathogenesis and risk of oral lichen planus neoplastic transformation: an ex-vivo retrospective immunohistochemical study. Histology and Histopathology, 34(9), 1015–1024.

Sridevi, U. et al. (2015) ‘Expression of E-cadherin in normal oral mucosa, in oral precancerous lesions and in oral carcinomas.’, European journal of dentistry, 9(3), pp. 364–372.

Srinivasan, M. et al. (2008) ‘Soluble CD14 and toll-like receptor-2 are potential salivary biomarkers for oral lichen planus and burning mouth syndrome’, Clinical Immunology, 126(1), pp. 31–37.

Stanimirovic, D. et al. (2013) ‘TLR2, TLR3, TLR4 and CD14 gene polymorphisms associated with oral lichen planus risk’, European Journal of Oral Sciences, 121(5), pp. 421–426.

Strempel H., Klein G., Rubesamen-Vokuhl A. and K.-D. B (1982), "Treatment of oral lichen planus with topical retinoic acid in mucosal adhesive base", Aktuelle Dermatologie. Vol. 8(4), pp. 116-118.

Suganya, G. et al. (2016) ‘Survivin expression in oral lichen planus: Role in malignant transformation.’, Journal of oral and maxillofacial pathology, 20(2), pp. 234–238.

Sugerman, P. B. et al. (1992) ‘Phenotypic and functional analysis of peripheral blood lymphocytes in oral lichen planus’, Journal of Oral Pathology and Medicine, 21(10), pp. 445–450.

Sugerman, P. B. et al. (1993) ‘Clonal expansion of lymphocytes from oral lichen planus lesions’, Journal of Oral Pathology and Medicine, 22(3), pp. 126–131.

Sugerman, P. B. et al. (1994) ‘Phenotype and suppressor activity of T-lymphocyte clones extracted from lesions of oral lichen planus’, British Journal of Dermatology, 131(3), pp. 319–324.

Sugerman, P. B. et al. (1995) ‘Heat shock protein expression in oral lichen planus’, Journal of Oral Pathology and Medicine, 24(1), pp. 1–8.

Sugerman, P. B. et al. (1996) ‘A quantitative cytological study of lesional and non-lesional mucosa in oral lichen planus.’, Archives of oral biology, 41(1), pp. 117–120.

Sugerman, P. B., Savage, N. W., & Seymour, G. J. (1992) ‘Suppressor cell function in oral lichen planus.’, Journal of dental research, 71(12), pp. 1916–1919.

Sugiyama, T. et al. (2015) ‘Detection of trace metallic elements in oral lichenoid contact lesions using SR-XRF, PIXE, and XAFS’, Scientific reports, 5, p. 10672.

Sulewska, M. et al. (2019) ‘A clinical evaluation of efficacy of photodynamic therapy in treatment of reticular oral lichen planus: A case series.’, Photodiagnosis and photodynamic therapy, 25, pp. 50–57.

Sun, A. et al. (1996) ‘Human cytomegalovirus as a potential etiologic agent in recurrent aphthous ulcers and Behcet’s disease.’, Journal of oral pathology & medicine: official publication of the International Association of Oral Pathologists and the American Academy of Oral Pathology, 25(5), pp. 212–218.

Sun, A. et al. (1998) ‘Preliminary evidence for an association of Epstein-Barr virus with pre-ulcerative oral lesions in patients with recurrent aphthous ulcers or Behcet’s disease.’, Journal of oral pathology & medicine, 27(4), pp. 168–175.

Sun, A. et al. (2002) ‘Serum interleukin-6 level is a useful marker in evaluating therapeutic effects of levamisole and Chinese medicinal herbs on patients with oral lichen planus.’, Journal of oral pathology & medicine: official publication of the International Association of Oral Pathologists and the American Academy of Oral Pathology, 31(4), pp. 196–203.

Sun, A. et al. (2008) ‘``Tien-Hsien{’’} liquid modulates antigen-stimulated cytokine production by T-cells from patients with erosive oral lichen planus’, Journal of dental sciences, 3(3), pp. 159–166.

Sun, A., & Chiang, C. P. (2001). Levamisole and/or Chinese medicinal herbs can modulate the serum level of squamous cell carcinoma associated antigen in patients with erosive oral lichen planus. Journal of Oral Pathology and Medicine, 30(9), 542–548.

Sun, A., Wang, J. T., Chia, J. S., & Chiang, C. P. (2005). Serum interleukin-8 level is a more sensitive marker than serum interleukin-6 level in monitoring the disease activity of oral lichen planus. British Journal of Dermatology, 152(6), 1187–1192.

Sun, A., Wu, Y. C., Liang, L. C., & Kwan, H. W. (1986). Serum immunoglobulins, complements and circulating immune complexes in oral lichen planus. Chinese Journal of Microbiology and Immunology, 19(1), 46–51.

Sun, M. et al. (2014) ‘In situ expression and serum level of thymic stromal lymphopoietin in oral lichen planus’, Journal of Oral Pathology and Medicine, 43(10), pp. 740–745.

Sun, Y. et al. (2016) ‘Immunosuppression Induced by Chronic Inflammation and the Progression to Oral Squamous Cell Carcinoma.’, Mediators of inflammation, 2016, p. 5715719.

Sunitha, M. and Shanmugam, S. (2006) ‘Evaluation of salivary nitric oxide levels in oral mucosal diseases: A controlled clinical trial.’, Indian journal of dental research: official publication of Indian Society for Dental Research, 17(3), pp. 117–120.

Supic, G. et al. (2015) ‘HMGB1 genetic polymorphisms in oral squamous cell carcinoma and oral lichen planus patients’, Oral Diseases, 21(4), pp. 536–543.

Suvarna, C., Chaitanya, N. C., Ameer, S., Mannava, H., Bontala, P., Alyami, J. S., Samreen, H., & Kondapaneni, J. (2020). A Comparative Evaluation on the Effect of Oral Zinc 50 mg with or without 0.1% Triamcinolone Orabase on Oral Lichen Planus. International Journal of Applied & Basic Medical Research, 10(1), 54–58.

Swan, R. H. and R.H., S. (1982) ‘Oral lichen planus with associated nail changes’, Journal of Oral Medicine, 37(1), pp. 23–26.

Swift, J. C. et al. (2005) ‘The effectiveness of 1% pimecrolimus cream in the treatment of oral erosive lichen planus.’, Journal of periodontology, 76(4), pp. 627–635.

T, A. et al. (2020) ‘Keratin 17-positive Civatte bodies in oral lichen planus-distribution variety, diagnostic significance and histopathogenesis.’, Scientific reports, 10(1), p. 14586.

T, C. et al. (2020) ‘Analysis of DNA methylation of E-cadherin and p16(ink4a) in oral lichen planus/oral lichenoid lesions.’, Clinical and experimental dental research.

T., A. et al. (1987) ‘Oral lichen planus--a demographic study.’, Community dentistry and oral epidemiology, 15(1), pp. 52–56.

T., F.-S. et al. (2012) ‘DNMT3B (C46359T) polymorphisms and immunoexpression of DNMT3b and DNMT1 proteins in oral lichen planus’, Pathobiology, 79(1), pp. 18–23.

T., I. and Ishii, T. (1987) ‘Immunohistochemical demonstration of T cell subsets and accessory cells in oral lichen planus.’, Journal of oral pathology, 16(7), pp. 356–361.

T., N. et al. (2001) ‘Serum antioxidant micronutrient levels in oral lichen planus’, Journal of Oral Pathology and Medicine, 30(5), pp. 264–267.

T., N. et al. (2018) ‘Comparison of the Effects of Curcumin Mucoadhesive Paste and Local Corticosteroid on the Treatment of Erosive Oral Lichen Planus Lesions’, Journal of the National Medical Association, 110(1), pp. 92–97.

T., S. et al. (2014) ‘Study of tape A behavior in patients with oral lichen planus’, Shanghai kou qiang yi xue = Shanghai journal of stomatology, 23(1), pp. 91–94.

T., Y. et al. (1994) ‘Serum cytokines, interleukin-2 receptor, and soluble intercellular adhesion molecule-1 in oral disorders.’, Oral surgery, oral medicine, and oral pathology, 78(6), pp. 727–735.

T., Y. et al. (1995) ‘Characteristic cytokines generated by keratinocytes and mononuclear infiltrates in oral lichen planus’, Journal of Investigative Dermatology, 104(5), pp. 784–788.

T.A., K. et al. (2019) ‘Expression of cluster of differentiation 1a-positive langerhans cells in oral lichen planus’, Indian Journal of Dermatology, 64(1), pp. 41–46.

T.E., B. et al. (1995) ‘Heat shock (stress) proteins and gamma delta T lymphocytes in oral lichen planus.’, Oral surgery, oral medicine, oral pathology, oral radiology, and endodontics, 80(6), pp. 698–704.

Tabatabaei, S. H. et al. (2018) ‘Evaluation of polymorphism of P53 protein codon 72 in oral lichen planus by PCR technique.’, Journal of dental research, dental clinics, dental prospects, 12(4), pp. 245–251.

Tabolli, S. et al. (2009) ‘Quality of life and psychological problems of patients with oral mucosal disease in dermatological practice’, Dermatology, 218(4), pp. 314–320.

Tadakamadla, J. et al. (2017) ‘Qualitative analysis of the impact of Oral Potentially Malignant Disorders on daily life activities’, PLoS ONE, 12(4), pp. e0175531–e0175531.

Taghavi Zenouz, A. et al. (2012) ‘Evaluation of Serum TNF-alpha and TGF-beta in Patients with Oral Lichen Planus.’, Journal of dental research, dental clinics, dental prospects, 6(4), pp. 143–147.

Tak, M. M. and Chalkoo, A. H. (2015) ‘Demographic, Clinical Profile of Oral Lichen Planus and its Possible Correlation with Thyroid Disorders: A Case-Control Study’, International journal of scientific study, 3(8), pp. 19–23.

Tak, M. M. and Chalkoo, A. H. (2017) ‘Vitamin d deficiency - a possible contributing factor in the aetiopathogenesis of oral lichen planus’, Journal of evolution of medical and dental sciences, 6(66), pp. 4769–4772.

Takeuchi, Y. et al. (1988) ‘Immunohistochemical analysis of cells in mucosal lesions of oral lichen planus.’, Journal of oral pathology, 17(8), pp. 367–373.

Talat, H. et al. (2012) ‘Cutaneous drug reactions to antihypertensive agents: A report from a single center in Pakistan’, Iranian Journal of Dermatology, 15(62), pp. 122–126.

Talungchit, S. et al. (2018) ‘Putative salivary protein biomarkers for the diagnosis of oral lichen planus: a case-control study’, BMC oral health, 18(1), p. 42.

Tan, Y.-Q. et al. (2016) ‘Altered Autophagy-Associated Genes Expression in T Cells of Oral Lichen Planus Correlated with Clinical Features’, Mediators of Inflammation, 2016, p. 4867368.

Tan, Y.-Q. et al. (2017) ‘Increased circulating CXCR5(+) CD4(+) T follicular helper-like cells in oral lichen planus.’, Journal of oral pathology & medicine: official publication of the International Association of Oral Pathologists and the American Academy of Oral Pathology, 46(9), pp. 803–809.

Taniguchi, Y. et al. (2002) ‘Epithelial cell proliferation in oral lichen planus’, Cell Proliferation, 35, pp. 103–109.

Tao, X. et al. (2007) ‘Assessment of local angiogenesis and vascular endothelial growth factor in the patients with atrophic-erosive and reticular oral lichen planus’, Oral Surgery, Oral Medicine, Oral Pathology, Oral Radiology, and Endodontics, 103(5), pp. 661–669.

Tao, X. et al. (2008) 'Simultaneous detection of IFN-gamma and IL-4 in lesional tissues and whole unstimulated saliva from patients with oral lichen planus', Journal of Oral Pathology and Medicine, 37(2), pp. 83–87.

Tao, X.-A. et al. (2009) 'Differential gene expression profiles of whole lesions from patients with oral lichen planus', Journal of Oral Pathology and Medicine, 38(5), pp. 427–433.

Tatullo, M. et al. (2015) 'Bioimpedance detection of Oral Lichen Planus used as preneoplastic model', Journal of Cancer, 6(10), pp. 976–983.

Tavangar, A. et al. (2016) 'Serum level of Interleukin-8 in subjects with diabetes, diabetes plus oral lichen planus, and oral lichen planus: A biochemical study.', Dental research journal, 13(5), pp. 413–418.

Tavangar, A. et al. (2017) 'Salivary levels of interleukin-8 in oral lichen planus and diabetic patients: A biochemical study.', Dental research journal, 14(3), pp. 209–214.

Tavassol, F. et al. (2008) 'Heat-shock protein expression and topical treatment with tacrolimus in oral lichen planus: an immunohistochemical study', International Journal of Oral and Maxillofacial Surgery, 37(1), pp. 66–69.

Teja, C. S. R. M., Devy, A. S. M., Nirmal, R. M. M., Sunil, P. M. M. P., & Deepasree, M. M. (2014). Cytomorphometric analysis of exfoliated cells in oral lichen planus. CytoJournal, 11(1), 3.

Thanyavuthi, A., Boonchai, W., & Kasemsarn, P. (2016). Amalgam Contact Allergy in Oral Lichenoid Lesions. Dermatitis, 27(4), 215–221.

Thi Do, T. et al. (2018) 'New evidence of connections between increased O-GlcNAcylation and inflammasome in the oral mucosa of patients with oral lichen planus', Clinical and experimental immunology, 192(1), pp. 129–137.

Thomas, A. E., Varma, B., Kurup, S., Jose, R., Chandy, M. L., Kumar, S. P., Aravind, M. S., & Ramadas, A. A. (2017). Evaluation of efficacy of 1% curcuminoids as local application in management of oral lichen planus - interventional study. Journal of Clinical and Diagnostic Research, 11(4), ZC89--ZC93.

Thomopouloudoukoudakis, A., Squier, C. A. and Hill, M. W. (1983) 'Distribution of abo blood-group substances in various types of oral lichen planus', Journal of oral pathology and medicine, 12(1), pp. 47–56.

Thongprasom, K. et al. (1992) 'Relative efficacy of fluocinolone acetonide compared with triamcinolone acetonide in treatment of oral lichen planus', Journal of oral pathology and medicine, 21(10), pp. 456–458.

Thongprasom, K. et al. (1998) 'Telomerase activity in oral lichen planus', Journal of Oral Pathology and Medicine, 27(8), pp. 395–398.

Thongprasom, K. et al. (2001) 'Folate and vitamin B12 levels in patients with oral lichen planus, stomatitis or glossitis.', The Southeast Asian journal of tropical medicine and public health, 32(3), pp. 643–647.

Thongprasom, K. et al. (2006) ‘Expression of TNF-alpha in oral lichen planus treated with fluocinolone acetonide 0.1%.’, Journal of oral pathology & medicine: official publication of the International Association of Oral Pathologists and the American Academy of Oral Pathology, 35(3), pp. 161–166.

Thongprasom, K. et al. (2007) ‘A randomized-controlled trial to compare topical cyclosporin with triamcinolone acetonide for the treatment of oral lichen planus’, Journal of Oral Pathology and Medicine, 36(3), pp. 142–146.

Tiitta, O. et al. (1995) ‘Tenascin expression in mucocutaneous diseases and related lesions of human oral mucosa.’, Archives of oral biology, 40(11), pp. 1039–1045.

Tikkhanarak, K. et al. (2019) ‘Correlation of clinicopathological characteristics and direct immunofluorescence studies in oral lichenoid lesion in Thai patients’, Journal of investigative and clinical dentistry, pp. e12433–e12433.

Tishkov, D. S., Peretyagina, I. N., & Brusentsova, A. E. (2016). Inclusion of immunotropic drugs into the pharmacological therapy of oral lichen planus. Journal of Global Pharma Technology, 8(8), 1–3.

Titarenko, M. A. et al. (2018) ‘The role of gastrointestinal pathology in the development and severity of oral lichen planus’, Byulleten sibirskoy meditsiny, 17(3), pp. 151–156.

TN, U. M., MS, N. and P, R. (2020) ‘Expression profile of salivary micro RNA-21 and 31 in oral potentially malignant disorders.’, Brazilian oral research, 34, p. e002.

Tobon-Arroyave, S. I. et al. (2004) ‘Expression of caspase-3 and structural changes associated with apoptotic cell death of keratinocytes in oral lichen planus’, Oral Diseases, 10(3), pp. 173–178.

Totan, A. et al. (2015) ‘Possible salivary and serum biomarkers for oral lichen planus’, Biotechnic & histochemistry : official publication of the Biological Stain Commission, 90(7), pp. 552–558.

Toto, P. D. et al. (1987) ‘An immunohistochemical study of oral lichen planus’, Oral Surgery Oral Medicine and Oral Pathology, 63(1), pp. 60–67.

Tsai, L. L. et al. (2009) ‘Concomitant upregulation of matrix metalloproteinase-2 in lesions and circulating plasma of oral lichen planus’, Journal of dental sciences, 4(1), pp. 7–12.

Tsounias, B. et al. (1986) ‘Topographical distribution and morphology of mast cells in oral lichen planus.’, Annals of dentistry, 45(2), pp. 37-39,49.

Tunali-Akbay, T. et al. (2017) ‘Salivary tissue factor concentration and activity in patients with oral lichen planus’, Oral science international, 14(1), pp. 13–17.

Tvarijonaviciute, A. et al. (2018) ‘Salivary Antioxidant Status in Patients with Oral Lichen Planus: Correlation with Clinical Signs and Evolution during Treatment with Chamaemelum nobile’, BioMed Research International, 2018, p. 5187549.

Tvarijonaviciute, A., Aznar-Cayuela, C., Rubio, C. P., Ceron, J. J., & Lopez-Jornet, P. (2017). Evaluation of salivary oxidate stress biomarkers, nitric oxide and C-reactive protein in patients with oral lichen planus and burning mouth syndrome. Journal of Oral Pathology and Medicine, 46(5), 387–392.

Tyagi, N., Shetty, D. C. and Urs, A. B. (2012) 'Altered expression of HSP70 in oral lichen planus.', Journal of oral and maxillofacial pathology, 16(2), pp. 189–194.

Ueta, E., Umazume, M., Yamamoto, T., & Osaki, T. (1993). Leukocyte dysfunction in oral mucous membrane diseases. Journal of Oral Pathology and Medicine, 22(3), 120–125.

Ujwala, N. et al. (2016) 'Colposcopy in pre-malignant lesions and oral squamous cell carcinoma: Linking threads of clinical, histopathological and colposcopic inferences', Journal of Cancer Research and Therapeutics, 12(1), pp. 295–301.

UM, O. et al. (2020) 'Prevalence of oral mucosal normal variations and lesions in a middle-aged population: a Northern Finland Birth Cohort 1966 study.', BMC oral health, 20(1), p. 357.

Urbizo Vélez, J. (2013) 'Oral lichen planus and epithelial dysplasia [Liquen plano bucal y displasia epitelial]', Revista Cubana de Estomatologia, 50(2), pp. 162–170.

Urbizo-Velez, J. et al. (1990) 'Comparative histopathological studies in oral lichen planus', Acta Morphologica Hungarica, 38(1), pp. 71–81.

Urek, M. M. et al. (2014) 'Electric potential difference and salivary ph in patients with erosive and non-erosive oral lichen planus [Razlika električnog potencijala i ph sline u pacijenata s erozivnim i neerozivnim oralnim lihen planusom]', Medicina Fluminensis, 50(3), pp. 317–324.

V., O. et al. (2002) 'Treatment of chronic erosive oral lichen planus with low concentrations of topical tacrolimus: An open prospective study', Archives of Dermatology, 138(10), pp. 1335–1338.

V., P. et al. (2019) 'Mannose-Binding Lectin 2 (MBL2) combined genotypes deficiency is associated with susceptibility for Oral Lichen Planus', Genetics and molecular biology, 42(1), pp. 9–14.

V.C., C. et al. (2011) 'Prevalence and risk indicators of oral mucosal lesions in an urban population from South Brazil', Oral Diseases, 17(2), pp. 171–179.

V.G., S. et al. (2017) 'Gustatory function and taste perception in patients with oral lichen planus and tongue involvement', Clinical oral investigations, 21(3), pp. 957–964.

V.G.A., S. et al. (2016) 'The role of patch testing in the management of oral lichenoid reactions', Journal of Oral Pathology and Medicine, 45(1), pp. 48–57.

V.N., S. (1974) 'Natural history of oral lichen planus', Indian J. Derm. Venereol., 40(5), pp. 204–207.

V.N., S. and V.L., R. (1974) 'Lichen planus: an appraisal of 147 cases', Indian J.Derm.venereol., 40(3), pp. 104–107.

V.N., S. et al. (1973) 'An evaluation of 55 lichen planus cases', International Journal of Dermatology, 12(6), pp. 358–365.

Vahide, L. et al. (2017) 'Autoantibodies to desmogleins 1 and 3 in patients with lichen planus', Archives of Dermatological Research, 309(7), pp. 579–583.

Vaish, R. P. et al. (1985) 'Prevalence of oral lichen planus among dental patients in southern Orissa.', Journal of the Indian Dental Association, 57(4), pp. 139–141.

Valente, G., Pagano, M., Carrozzo, M., Carbone, M., Bobba, V., Palestro, G., & Gandolfo, S. (2001). Sequential immunohistochemical p53 expression in biopsies of oral lichen planus undergoing malignant evolution. Journal of Oral Pathology and Medicine, 30(3), 135–140.

van der Hem, P. S. et al. (2008) 'CO2 laser evaporation of oral lichen planus', International Journal of Oral and Maxillofacial Surgery, 37(7), pp. 630–633.

van der Meij, E. H. et al. (1999) 'Interobserver and intraobserver variability in the histologic assessment of oral lichen planus', Journal of Oral Pathology and Medicine, 28(6), pp. 274–277.

van der Meij, E. H. et al. (2002) 'Cost-effectiveness of screening for the possible development of cancer in patients with oral lichen planus.', Community dentistry and oral epidemiology, 30(5), pp. 342–351

van der Meij, E. H., Mast, H., & van der Waal, I. (2007). The possible premalignant character of oral lichen planus and oral lichenoid lesions: A prospective five-year follow-up study of 192 patients. Oral Oncology, 43(8), 742–748.

van der Meij, E. H., Schepman, K.-P., & van der Waal, I. (2003). The possible premalignant character of oral lichen planus and oral lichenoid lesions: A prospective study. Oral Surgery, Oral Medicine, Oral Pathology, Oral Radiology, and Endodontics, 96(2), 164–171.

Vankadara, S. et al. (2018) 'Evaluation of Serum C-Reactive Protein Levels in Oral Premalignancies and Malignancies: A Comparative Study.', Journal of dentistry (Tehran, Iran), 15(6), pp. 358–364.

Velez, I., Spielholz, N. I., Siegel, M. A., & Gonzalez, T. (2014). MuGard, an oral mucoadhesive hydrogel, reduces the signs and symptoms of oral mucositis in patients with lichen planus: a double-blind, randomized, placebo-controlled pilot study. Oral Surgery, Oral Medicine, Oral Pathology and Oral Radiology, 118(6), 657–664.

Veneri, F., Bardellini, E., Amadori, F., Conti, G., & Majorana, A. (2020). Efficacy of ozonized water for the treatment of erosive oral lichen planus: a randomized controlled study. Medicina Oral, Patologia Oral y Cirugia Bucal, 25(5), e675–e682

Venkatesiah, S. S. et al. (2013) ‘Histomorphometric analysis of nuclear and cellular volumetric alterations in oral lichen planus, lichenoid lesions and normal oral mucosa using image analysis software’, Indian journal of dental research, 24(2), p. 277.

Vesper, M. et al. (1997) ‘[Detection of human papillomavirus (HVP)-DNA in oral manifestation of lichen planus].’, Mund-, Kiefer- und Gesichtschirurgie : MKG, 1(3), pp. 146–149.

Vidyalakshmi, S. et al. (2016) ‘Buccal micronuclei assay as a tool for biomonitoring DNA damage in oral lichen planus’, Journal of Clinical and Diagnostic Research, 10(7), pp. 5–7.

Vieira, R. da R. et al. (2016) ‘Detection of Epstein-Barr virus in different sources of materials from patients with oral lichen planus: A case-control study’, Journal of Clinical Pathology, 69(4), pp. 358–363.

Viguier, M. et al. (2015) ‘Peripheral and local human papillomavirus 16-specific CD8+ T-cell expansions characterize erosive oral lichen planus.’, The Journal of investigative dermatology, 135(2), pp. 418–424.

Villa, A., Sankar, V., Bassani, G., Johnson, L. B., & Sroussi, H. (2020). Dexamethasone solution and dexamethasone in Mucolox for the treatment of oral lichen planus: a preliminary study. Oral Surgery, Oral Medicine, Oral Pathology and Oral Radiology, 129(6), 585–590.

Vohra, S. et al. (2016) ‘Clinical and serological efficacy of topical calcineurin inhibitors in oral lichen planus: A prospective randomized controlled trial’, International Journal of Dermatology, 55(1), pp. 101–105.

Volodina, E. V, Maksimovskii, I. M. and Lebedev, K. A. (1997) ‘[The combined treatment of lichen ruber planus of the mouth mucosa].’, Stomatologiia, 76(2), pp. 28–32.

Voute, A. B. et al. (1994) ‘Cyclosporin A in an adhesive base for treatment of recalcitrant oral lichen planus. An open trial.’, Oral surgery, oral medicine, and oral pathology, 78(4), pp. 437–441.

Voute, A. B., de Jong, W. F., Schulten, E. A., Snow, G. B., & van der Waal, I. (1992). Possible premalignant character of oral lichen planus. The Amsterdam experience. Journal of Oral Pathology and Medicine, 21(7), 326–329.

Voute, A. B., Schulten, E. A., Langendijk, P. N., Kostense, P. J., & van der Waal, I. (1993). Fluocinonide in an adhesive base for treatment of oral lichen planus: A double-blind, placebo-controlled clinical study. Oral Surgery Oral Medicine and Oral Pathology, 75(2), 181–185.

Vucicevic-Boras, V. et al. (2007) ‘Salivary IgA and IgG Subclass Levels in Patients with Oral Lichen Planus - A Pilot Study’, Acta stomatologica croatica, 41(1), pp. 13–22.

W, S. et al. (2020) ‘Could photodynamic therapy be utilized as a treatment modality for oral lichen planus?’, Photodiagnosis and photodynamic therapy, 30, p. 101677.

W, W. et al. (2020) ‘Enhanced T-cell proliferation and IL-6 secretion mediated by overexpression of TRIM21 in oral lesions of patients with oral lichen planus.’, Journal of oral pathology & medicine: official publication of the International Association of Oral Pathologists and the American Academy of Oral Pathology, 49(4), pp. 350–356.

W.-B., L. et al. (2017) ‘Treatment of oral lichen planus using 308-nm excimer laser’, Dermatologic Therapy, 30(5).

W.-D., L. et al. (2019) ‘Clinical evaluation of modified Xiaoyao pill on the treatment of oral lichen planus patients with anxiety or depression’, Shanghai kou qiang yi xue = Shanghai journal of stomatology, 28(3), pp. 312–316.

W.-Z., L. et al. (2014) ‘Interferon-γ and interleukin-4 detected in serum and saliva from patients with oral lichen planus’, International Journal of Oral Science, 6(1), pp. 22–26.

W., B. et al. (1997) ‘Efficacy of topical retinoic acid compared with topical fluocinolone acetonide in the treatment of oral lichen planus.’, Oral surgery, oral medicine, oral pathology, oral radiology, and endodontics, 83(1), pp. 21–25.

W., B. et al. (2000) ‘Efficacy of fluocinolone acetonide gel in the treatment of oral lichen planus.’, Oral surgery, oral medicine, oral pathology, oral radiology, and endodontics, 89(1), pp. 42–45.

W., B. et al. (2015) ‘Direct immunofluorescence in oral lichen planus’, Journal of Clinical and Diagnostic Research, 9(8), pp. ZC34--ZC37.

W., L. et al. (2009) ‘IFN-gamma and IL-4 in saliva of patients with oral lichen planus: A study in an ethnic chinese population’, Inflammation, 32(3), pp. 176–181.

W., W. et al. (2006) ‘Role of microchimerism in the pathogenesis of oral lichen planus’, Experimental Dermatology, 15(2), pp. 125–129.

W., Z. et al. (2012) ‘Oral health-related quality of life in patients with oral lichen planus’, Hua xi kou qiang yi xue za zhi = Huaxi kouqiang yixue zazhi = West China journal of stomatology, 30(1), pp. 40–44.

W., Z. et al. (2013) ‘A randomized single-blind controlled clinical trial of tacrolimus mouth rinse on erosive oral lichen planus’, Shanghai kou qiang yi xue = Shanghai journal of stomatology, 22(6), pp. 708–710.

W.F.B., D. J. et al. (1984) ‘Epithelial dysplasia in oral lichen planus. A preliminary report of a Dutch-Hungarian study of 100 cases’, International Journal of Oral Surgery, 13(3), pp. 221–225.

W.R., T. et al. (1977) ‘Betamethasone valerate aerosol in the treatment of oral lichen planus’, British Journal of Dermatology, 96(6), pp. 659–662.

Wang H. H., Jiang Y., Wang H., Luo Z, Wang Y. and Guan X. (2019), "IL-25 promotes Th2-type reactions and correlates with disease severity in the pathogenesis of oral lichen planus", Archives of oral biology. Vol. 98, pp. 115-121.

Wang H., He F., Xu C., Fang C. & Peng J. (2018), "Clinical analysis for oral mucosal disease in 21 972 cases", Zhong nan da xue xue bao. Yi xue ban = Journal of Central South University. Medical sciences. Vol. 43(7), pp. 779-783.

Wang, C. J., Li, Y. J., Xue, J. N., Ci, H. S., Li, L. P., & Li, L. (2016). *Shanghai kou qiang yi xue = Shanghai journal of stomatology*, *25*(4), 438–442.

Wang, F., Zhang, J., & Zhou, G. (2019). Deregulated phospholipase D2/mammalian target of rapamycin/hypoxia-inducible factor 1 alpha in peripheral T lymphocytes of oral lichen planus correlated with disease severity. Archives of Oral Biology, 98, 26–31.

Wang, H. et al. (2013) 'Interaction between oral lichen planus and chronic periodontitis with Th17-associated cytokines in serum', Inflammation, 36(3), pp. 696–704.

Wang, H. et al. (2014) 'Oral lichen planus may enhance the expression of Th17-associated cytokines in local lesions of chronic periodontitis', Clinical oral investigations, 18(6), pp. 1647–1654.

Wang, H. H. et al. (2017) 'Overexpression and varied clinical significance of Th9 versus Th17 cells in distinct subtypes of oral lichen planus', Archives of oral biology, 80, pp. 110–116.

Wang, J. et al. (2018) 'Forkhead box p3 controls progression of oral lichen planus by regulating microRNA-146a', Journal of Cellular Biochemistry, 119(11), pp. 8862–8871.

Wang, J. et al. (2019) 'Long non-coding RNA DQ786243 modulates the induction and function of CD4(+) Treg cells through Foxp3-miR-146a-NF-kappa B axis: Implications for alleviating oral lichen planus', International immunopharmacology, 75, p. 105761.

Wang, K. et al. (2015) 'Analysis of oral microbial community and Th17-associated cytokines in saliva of patients with oral lichen planus', Microbiology and Immunology, 59(3), pp. 105–113.

Wang, L.-L. et al. (2018) 'Expression of keratinocyte growth factor and its receptor in oral lichen planus', International journal of clinical and experimental pathology, 11(2), pp. 757–764.

Wang, T.-Y. et al. (2018) 'Malignant transformation of Taiwanese patients with oral leukoplakia: A nationwide population-based retrospective cohort study', Journal of the Formosan Medical Association, 117(5), pp. 374–380.

Wang, X.-X. et al. (2017) 'Hypoxia-inducible factor-1 alpha and glucose transporter 1 in the malignant transformation of oral lichen planus', International journal of clinical and experimental pathology, 10(8), pp. 8369–8376.

Wang, Y. et al. (2015) 'A Study of Association Between Oral Lichen Planus and Immune Balance of Th1/Th2 Cells', Inflammation, 38(5), pp. 1874–1879.

Wang, Y.-Y. et al. (2014) 'Malignant transformation in 5071 southern Taiwanese patients with potentially malignant oral mucosal disorders.', BMC oral health, 14, p. 99.

Wang, Yanni et al. (2016) ‘Total glucosides of paeony (TGP) inhibits the production of inflammatory cytokines in oral lichen planus by suppressing the NF-kappaB signaling pathway.’, International immunopharmacology, 36, pp. 67–72.

Warnakulasuriya, K. A. and Johnson, N. W. (1996) ‘Sensitivity and specificity of OraScan (R) toluidine blue mouthrinse in the detection of oral cancer and precancer.’, Journal of oral pathology & medicine : official publication of the International Association of Oral Pathologists and the American Academy of Oral Pathology, 25(3), pp. 97–103.

Warnakulasuriya, S. et al. (2011) ‘Factors predicting malignant transformation in oral potentially malignant disorders among patients accrued over a 10-year period in South East England.’, Journal of oral pathology & medicine: official publication of the International Association of Oral Pathologists and the American Academy of Oral Pathology, 40(9), pp. 677–683.

Weber, B., Schlapbach, C., Stuck, M., Simon, H.-U., Borradori, L., Beltraminelli, H., & Simon, D. (2017). Distinct interferon-gamma and interleukin-9 expression in cutaneous and oral lichen planus. Journal of the European Academy of Dermatology and Venereology, 31(5), 880–886.

Wei, B.-J. et al. (2009) ‘Expression of osteopontin in oral mucosal epithelium in patients with oral lichen planus’, Journal of Shanghai Jiaotong University (Medical Science), 29(3), pp. 316–318.

Wei, W. et al. (2018) ‘Mixed and inhomogeneous expression profile of Th1/Th2 related cytokines detected by cytometric bead array in the saliva of patients with oral lichen planus’, Oral surgery, oral medicine, oral pathology and oral radiology, 126(2), pp. 142–151.

Williams, A. et al. (2019) ‘Immunohistochemical Expression Patterns of Inflammatory Cells Involved in Chronic Hyperplastic Candidosis.’, Pathogens (Basel, Switzerland), 8(4).

Wiriyakijja, P. (2020) ‘Meaningful improvement thresholds in measures of pain and quality of life in oral lichen planus.’, Oral diseases, 26(7), pp. 1464–1473.

Wiriyakijja, P. (2020) ‘Validation of the HADS and PSS-10 and psychological status in patients with oral lichen planus.’, Oral diseases, 26(1), pp. 96–110.

Won, T. H. et al. (2009) ‘Levamisole monotherapy for oral lichen planus.’, Annals of dermatology, 21(3), pp. 250–254.

Wong, L. et al. (2003) ‘Oral lichenoid lesions (OLL) and mercury in amalgam fillings’, Contact Dermatitis, 48(2), pp. 74–79.

Wongwatana, S. et al. (2005) ‘Oxpentifylline is not effective for symptomatic oral lichen planus’, Journal of Oral Pathology and Medicine, 34(2), pp. 106–108.

Worle, B., Wollenberg, A., Schaller, M., Kunzelmann, K. H., Plewig, G., & Meurer, M. (1997). Chronic ulcerative stomatitis. British Journal of Dermatology, 137(2), 262–265.

Wu, D. et al. (2013) ‘CIITA rs4774 and rs6498122 polymorphisms are associated with oral lichen planus in Chinese people: A case-control study’, European Journal of Oral Sciences, 121(2), pp. 69–75.

Wu, D., Chen, X., Dong, C., Liu, Q., Yang, Y., He, C., Wang, J., Sun, M., & Wu, Y. (2015). Corrigendum to Association of single nucleotide polymorphisms in MPO and COX genes with oral lichen planus. International Journal of Immunogenetics, 42(3), 161–167.

Wu, D., Cheng, S., Chen, X., Sun, M., Wang, G., Fu, S., Dong, G., Wang, L., & Wu, Y. (2014). Mitochondrial haplogroup B4 may be a protective factor to oral lichen planus susceptibility in Chinese. Oral Diseases, 20(1), 62–68.

Wu, Y. et al. (2000) ‘Study on regulatory effect of composite taixian tablet on immune function of red blood cell in patients with oral lichen planus’, Zhongguo Zhong xi yi jie he za zhi Zhongguo Zhongxiyi jiehe zazhi = Chinese journal of integrated traditional and Western medicine / Zhongguo Zhong xi yi jie he xue hui, Zhongguo Zhong yi yan jiu yuan zhu ban, 20(4), pp. 261–263.

X, Y. et al. (2020) ‘Profiling risk factors of micro-invasive carcinoma within oral potentially malignant disorders: a cross-sectional study.’, Clinical oral investigations, 24(10), pp. 3715–3720.

X.-A., T. et al. (2010) ‘FOXP3+ T regulatory cells in lesions of oral lichen planus correlated with disease activity’, Oral Diseases, 16(1), pp. 76–82.

X.-C., L. et al. (2014) ‘Low expression of glucocorticoid receptor α in oral lichen planus correlates with activation of nuclear factor κB: A preliminary study’, Journal of Oral Pathology and Medicine, 43(8), pp. 600–605.

X.-Y., Y. et al. (2017) ‘Analysis of human serum metabolome for potential biomarkers identification of erosive oral lichen planus’, Clinica Chimica Acta, 468, pp. 46–50.

X.-Z., L. et al. (2019) ‘Serum-based metabolomics characterization of patients with reticular oral lichen planus’, Archives of oral biology, 99, pp. 183–189.

X., Z. et al. (2005) ‘Adhesion to buccal epithelial cells of Candida albicans isolates from oral lichen planus’, Hua xi kou qiang yi xue za zhi = Huaxi kouqiang yixue zazhi = West China journal of stomatology, 23(6), pp. 537–538.

X., Z. et al. (2008) ‘Genotypic profiles and virulence attributes of Candida albicans isolates from patients with oral lichen planus’, APMIS, 116(4), pp. 284–291.

X.J., Z. et al. (2002) ‘Intra-epithelial CD8+ T cells and basement membrane disruption in oral lichen planus.’, Journal of oral pathology & medicine : official publication of the International Association of Oral Pathologists and the American Academy of Oral Pathology, 31(1), pp. 23–27

X.L., Z. (1984) ‘Histopathologic analysis of oral lichen planus’, Zhonghua kou qiang ke za zhi [Chinese journal of stomatology], 19(1), pp. 9–12.

X.L., Z. and L.F., Z. (1984) ‘Ultrastructural observations on the basement membrane in oral lichen planus’, Zhonghua yi xue za zhi, 64(12), pp. 738-740,792.

Xavier, G. M. et al. (2007) ‘Investigation of functional gene polymorphisms interleukin-1 beta, interleukin-6, interleukin-10 and tumor necrosis factor in individuals with oral lichen planus’, Journal of Oral Pathology and Medicine, 36(8), pp. 476–481.

Xiao, Y. (1990) ‘[Primary investigation of multiple factors in hemorrheology of oral lichen planus].’, Zhonghua kou qiang yi xue za zhi = Zhonghua kouqiang yixue zazhi = Chinese journal of stomatology, 25(5), pp. 291–293.

Xiao, Y. and Y., X. (1993) ‘Multiple comprehensive analyses of immune regulate factors in patients with oral lichen planus’, Zhonghua kou qiang yi xue za zhi = Zhonghua kouqiang yixue zazhi = Chinese journal of stomatology, 28(1), pp. 38–40.

Xiong, C., Li, Q., Lin, M., Li, X., Meng, W., Wu, Y., Zeng, X., Zhou, H., & Zhou, G. (2009). The efficacy of topical intralesional BCG-PSN injection in the treatment of erosive oral lichen planus: A randomized controlled trial. Journal of Oral Pathology and Medicine, 38(7), 551–558.

XS, W. et al. (2020) ‘Potential Metabolic Biomarkers for Early Detection of Oral Lichen Planus, a Precancerous Lesion.’, Frontiers in pharmacology, 11, p. 603899.

Xu, J. et al. (2019) ‘Increased granulysin in the peripheral blood and tissues of patients with oral lichen planus’, International journal of clinical and experimental pathology, 12(5), pp. 1634–1641.

Xu, N. et al. (2019) ‘Role of mammary serine protease inhibitor on the inflammatory response in oral lichen planus’, Oral Diseases, 25(4), pp. 1091–1099.

Xu, Z. et al. (2013) ‘Aldehyde dehydrogenase 1 expression correlated with malignant potential of oral lichen planus’, Annals of Diagnostic Pathology, 17(5), pp. 408–411.

XY, Y., XZ, L. and SN, Z. (2020) ‘Urinary metabolomic signatures in reticular oral lichen planus.’, Heliyon, 6(5), p. e04041.

Y, L. et al. (2020) ‘Alteration of Streptococcus salivarius in Buccal Mucosa of Oral Lichen Planus and Controlled Clinical Trial in OLP Treatment.’, Probiotics and antimicrobial proteins, 12(4), pp. 1340–1348.

Y.-C., W. et al. (2014) ‘Oral manifestations and blood profile in patients with iron deficiency anemia’, Journal of the Formosan Medical Association, 113(2), pp. 83–87.

Y.-C., W. et al. (2018) ‘Gastric parietal cell and thyroid autoantibodies in recurrent aphthous stomatitis patients with concomitant oral lichen planus’, Journal of the Formosan Medical Association, 117(11), pp. 987–993.

Y., C. et al. (2008) ‘MMPs, TIMP-2, AND TGF-β1 in the cancerization of oral lichen planus’, Head and Neck, 30(9), pp. 1237–1245.

Y., H. et al. (2017) ‘Dysbiosis of oral buccal mucosa microbiota in patients with oral lichen planus’, Oral Diseases, 23(5), pp. 674–682.

Y., K. et al. (2018) ‘Diagnosis of oral lichen planus from analysis of saliva samples using terahertz time-domain spectroscopy and chemometrics’, Journal of biomedical optics, 23(4), pp. 1–8.

Y., N. et al. (1996) ‘Effectiveness of glycyrrhizin for oral lichen planus in patients with chronic HCV infection’, Journal of gastroenterology, 31(5), pp. 691–695.

Y., N. et al. (2000) ‘Histopathological and immunohistochemical study of oral lichen planus- associated HCV infection’, European Journal of Internal Medicine, 11(5), pp. 277–282.

Y., W. et al. (2010) ‘A randomized double-blind, positive-control trial of topical thalidomide in erosive oral lichen planus’, Oral Surgery, Oral Medicine, Oral Pathology, Oral Radiology and Endodontology, 110(2), pp. 188–195.

Y., W. et al. (2016) ‘The expression and clinical significance of IL-35 in periodontitis and oral lichen planus’, Shanghai kou qiang yi xue = Shanghai journal of stomatology, 25(4), pp. 443–447.

Y., W. et al. (2018) ‘Increased infiltration of CD11 c+/CD123+ dendritic cell subsets and upregulation of TLR/IFN-α signaling participate in pathogenesis of oral lichen planus’, Oral surgery, oral medicine, oral pathology and oral radiology, 125(5), pp. 459–467.

Y., Z. et al. (2008) ‘NF-κB-dependent cytokines in saliva and serum from patients with oral lichen planus: A study in an ethnic Chinese population’, Cytokine, 41(2), pp. 144–149.

Y., Z. et al. (2014) ‘Hydroxychloroquine decreases the upregulated frequencies of Tregs in patients with oral lichen planus’, Clinical oral investigations, 18(8), pp. 1903–1911.

Yaacob, H. B. (1981) ‘Oral lichen planus--a study of fifty-four cases.’, The Medical journal of Malaysia, 36(4), pp. 239–242.

Yamamoto, T. et al. (1994) ‘Cytokine production by keratinocytes and mononuclear infiltrates in oral lichen planus’, Journal of Oral Pathology and Medicine, 23(7), pp. 309–315.

Yamanaka, Y. et al. (2018) ‘Direct Immunofluorescence as a Helpful Tool for the Differential Diagnosis of Oral Lichen Planus and Oral Lichenoid Lesions’, American Journal of Dermatopathology, 40(7), pp. 491–497.

Yan, S.-K. et al. (2008) ‘A metabonomic approach to the diagnosis of oral squamous cell carcinoma, oral lichen planus and oral leukoplakia’, Oral Oncology, 44(5), pp. 477–483.

Yanatatsaneeji, P. et al. (2010) ‘Codon72 polymorphism in the p53 tumor suppressor gene in oral lichen planus lesions in a Thai population’, Asian Pacific Journal of Cancer Prevention, 11(4), pp. 1137–1141.

Yang, J.-G. et al. (2016) ‘Different Expression of MicroRNA-146a in Peripheral Blood CD4+ T Cells and Lesions of Oral Lichen Planus’, Inflammation, 39(2), pp. 860–866.

Yang, X.-Y. et al. (2018) 'Metabolomics analysis of oral mucosa reveals profile perturbation in reticular oral lichen planus', Clinica Chimica Acta, 487, pp. 28–32.

Yao, H. and Wu, B. X. (1998) '[The content of estradiol receptors in oral mucosa precancerous lesions and its effect]', Shanghai kou qiang yi xue = Shanghai journal of stomatology, 7(4), pp. 204–206.

Yarom, N. et al. (2009) 'Chromosomal numerical aberrations in oral lichen planus.', Journal of dental research, 88(5), pp. 427–432.

Yen, A. M.-F. et al. (2008) 'The effect of betel quid and cigarette on multistate progression of oral pre-malignancy.', Journal of oral pathology & medicine, 37(7), pp. 417–422.

Yeshurun, A. et al. (2019) 'Hydroxychloroquine sulphate therapy of erosive oral lichen planus', Australasian Journal of Dermatology, 60(2), pp. e109--e112.

Yiannias, J. A. et al. (2000) 'Relevant contact sensitivities in patients with the diagnosis of oral lichen planus', Journal of the American Academy of Dermatology, 42(2), pp. 177–182.

Yiemstan, S., Krisdapong, S., & Piboonratanakit, P. (2020) 'Association between Clinical Signs of Oral Lichen Planus and Oral Health-Related Quality of Life: A Preliminary Study.', Dentistry journal, 8(4).

Yildirim, B. et al. (2011) 'Prevalence of herpes simplex, Epstein Barr and human Papilloma viruses in oral lichen planus', Medicina Oral, Patologia Oral y Cirugia Bucal, 16(2), pp. e170--e174.

Yoke, P.C. et al. (2006) 'A randomized controlled trial to compare steroid with cyclosporine for the topical treatment of oral lichen planus', Oral Surgery, Oral Medicine, Oral Pathology, Oral Radiology, and Endodontics, 102(1), pp. 47–55.

Yoshida H., Imamura Y., Yoshimura H. & Kobayashi M. (2020), "Induction of High Endothelial Venule-like Vessels in Oral and Cutaneous Lichen Planus: A Comparative Study.", The journal of histochemistry and cytochemistry : official journal of the Histochemistry Society. Vol. 68(5), pp. 343-350.

Yosipovitch, G. et al. (2001) 'Distribution of mucosal pH on the bucca, tongue, lips and palate: A study in healthy volunteers and patients with lichen planus, Behçet's disease and burning mouth syndrome', Acta Dermato-Venereologica, 81(3), pp. 178–180.

Youngnak-Piboonratanakit, P. et al. (2004) 'The expression of B7-H1 on keratinocytes in chronic inflammatory mucocutaneous disease and its regulatory role.', Immunology letters, 94(3), pp. 215–222.

Youngnak-Piboonratanakit, P. et al. (2009) 'Expression of IFN-gamma before and after treatment of oral lichen planus with 0.1% fluocinolone acetonide in orabase.', Journal of oral pathology & medicine, 38(9), pp. 689–694.

Yu, F. et al. (2019) 'Anti-inflammatory effect of paeoniflorin combined with baicalin in oral inflammatory diseases.', Oral diseases.

Z., P. et al. (2018) ‘The effectiveness of the combined use of a polysaccharide film with photodynamic action in complex therapy of oral lichen planus in the oral cavity’, Drug Invention Today, 10(12), pp. 2553–2559.

Zagorodnyaya, E. B. et al. (2013) ‘Biopolymeric film containing bioactive naphthoquinone (shikonin) in combined therapy of inflammatory destructive lesions in the buccal mucosa.’, Bulletin of experimental biology and medicine, 156(2), pp. 232–235.

Zain, R. B. et al. (1997) ‘A national epidemiological survey of oral mucosal lesions in Malaysia.’, Community dentistry and oral epidemiology, 25(5), pp. 377–383.

Zarei, M. R. et al. (2007) ‘Detection of human papillomavirus DNA sequences in oral lesions using polymerase chain reaction’, Acta Medica Iranica, 45(3), pp. 177–182.

Zargaran, M., Baghaei, F. and Moghimbeigi, A. (2018) ‘Comparative study of beta-catenin and CD44 immunoexpression in oral lichen planus and squamous cell carcinoma.’, International journal of dermatology, 57(7), pp. 794–798.

Zaslansky, R. et al. (2018) ‘Topical application of morphine for wound healing and analgesia in patients with oral lichen planus: a randomized, double-blind, placebo-controlled study’, Clinical oral investigations, 22(1), pp. 305–311.

Zenouz, A. T. et al. (2014) ‘Evaluation of relationship between salivary cortisol levels and stress intensity in oral lichen planus patients’, Der Pharmacia Lettre, 6(6), pp. 459–461.

Zhan, F., Wang, H., & Wang, Z. (1996). Fibronectin in tissues and saliva of patients with oral lichen planus. Zhonghua Kou Qiang Yi Xue Za Zhi = Zhonghua Kouqiang Yixue Zazhi = Chinese Journal of Stomatology, 31(4), 235–237.

Zhang, D. et al. (2015) ‘The Activation of NF-kappaB in Infiltrated Mononuclear Cells Negatively Correlates with Treg Cell Frequency in Oral Lichen Planus.’, Inflammation, 38(4), pp. 1683–1689.

Zhang, J. et al. (2013) ‘Expression and significance of retinoid-related orphan receptor gamma t and 4-1BB/4-1BBL in oral lichen planus’, Hua xi kou qiang yi xue za zhi = Huaxi kouqiang yixue zazhi = West China journal of stomatology, 31(4), pp. 408–411.

Zhang, J. et al. (2016) ‘Declined hTERT expression of peripheral blood CD4(+) T cells in oral lichen planus correlated with clinical parameter.’, Journal of oral pathology & medicine, 45(7), pp. 516–522.

Zhang, L. et al. (1996) ‘p53 overexpression in oral lichen planus’, Oncology Reports, 3(6), pp. 1145–1148.

Zhang, N. et al. (2017) ‘Activated Akt/mTOR-autophagy in local T cells of oral lichen planus’, International Immunopharmacology, 48, pp. 84–90.

Zhang, W.-Y. et al. (2012) ‘Altered microRNA expression profile with miR-27b down-regulation correlated with disease activity of oral lichen planus’, Oral Diseases, 18(3), pp. 265–270.

Zhang, Y. et al. (2012) 'Salivary and serum interleukin-18 in patients with oral lichen planus: A study in an ethnic Chinese population', Inflammation, 35(2), pp. 399–404.

Zhang, Z. et al. (2015) 'Interferon-γ regulates the function of mesenchymal stem cells from oral lichen planus via indoleamine 2,3-dioxygenase activity', Journal of Oral Pathology and Medicine, 44(1), pp. 15–27.

Zhang, Z.-R. et al. (2018) 'Expression and clinical significance of periostin in oral lichen planus', Experimental and Therapeutic Medicine, 15(6), pp. 5141–5147.

Zhao, B. et al. (2018) 'LPS-induced Vitamin D Receptor Decrease in Oral Keratinocytes Is Associated With Oral Lichen Planus', Scientific reports, 8(1), p. 763.

Zhao, L. (2018) 'Clinical effect of initial periodontal therapy combined with topical medication therapy on erosive oral lichen planus', International journal of clinical and experimental medicine, 11(7), pp. 7346–7351.

Zhao, Z. et al. (2018) 'Total glucosides of paeony improves the immunomodulatory capacity of MSCs partially via the miR-124/STAT3 pathway in oral lichen planus.', Biomedicine & pharmacotherapy = Biomedecine & pharmacotherapie, 105, pp. 151–158.

Zhao, Z. Z. et al. (1997) 'Immunohistochemical localization of mast cells and mast cell-nerve interactions in oral lichen planus', Oral Diseases, 3(2), pp. 71–76.

Zhao, Z. Z. et al. (1998) 'Associations between mast cells and laminin in oral lichen planus', Journal of Oral Pathology and Medicine, 27(4), pp. 163–167.

Zhou, G. et al. (2009) 'Activation of nuclear factor-kappa B correlates with tumor necrosis factor-alpha in oral lichen planus: a clinicopathologic study in atrophic-erosive and reticular form.', Journal of oral pathology & medicine: official publication of the International Association of Oral Pathologists and the American Academy of Oral Pathology, 38(7), pp. 559–564.

Zhou, G. et al. (2012) 'Increased B7-H1 expression on peripheral blood T cells in oral lichen planus correlated with disease severity', Journal of Clinical Immunology, 32(4), pp. 794–801.

Zhou, G., Fan, M., & Liu, J. (2005). [Effects of glucocorticoids on T helper cells balance in oral lichen planus]. Zhonghua Kou Qiang Yi Xue Za Zhi = Zhonghua Kouqiang Yixue Zazhi = Chinese Journal of Stomatology, 40(2), 98–101.

Zhou, L. et al. (2016) 'Clinical observation on the treatment of oral lichen planus with total glucosides of paeony capsule combined with corticosteroids', International Immunopharmacology, 36, pp. 106–110.

Zhou, Z.-T., Wei, B.-J. and Shi, P. (2008) 'Osteopontin expression in oral lichen planus.', Journal of oral pathology & medicine: official publication of the International Association of Oral Pathologists and the American Academy of Oral Pathology, 37(2), pp. 94–98.

Zhu, J. H. et al. (2015) 'Interferon-gamma and interleukin-10 levels in serum and saliva are related to different types of oral lichen planus', Chinese Journal of Tissue Engineering Research, 19(2), pp. 236–240.

Zunt, S. L. et al. (2009) ‘Soluble forms of Toll-like receptor 4 are present in human saliva and modulate tumour necrosis factor-α secretion by macrophage-like cells’, Clinical and Experimental Immunology, 156(2), pp. 285–293.

11.2 List S4. Overlapping population (n=2).

Cigic, L., Gavic, L., Simunic, M., Ardalic, Z., Biocina-Lukenda, D., L. (2015). Increased prevalence of celiac disease in patients with oral lichen planus. Clinical Oral Investigations, 19(3), 627–635.

Wiriyakijja, P., Porter, S., Fedele, S., Hodgson, T., McMillan, R., Shephard, M., & Ni Riordain, R. (2020). Health-related quality of life and its associated predictors in patients with oral lichen planus: a cross-sectional study. International Dental Journal. Advance online publication.
